# Extracellular matrix stiffness directs region-specific lung epithelial differentiation revealed by hPSC-derived lung organoids

**DOI:** 10.1038/s41467-026-75663-9

**Published:** 2026-07-17

**Authors:** Zhiying Liao, Hao Meng, Junjie Lv, Dong Wang, Hengrui Zhang, Runxi Jiang, Ruihao Lan, Yu Chen, Jiaqi Sun, Zonghong Li, Xiangping Yang, Xuepeng Chen, Jincun Zhao, Tao Xu, Huisheng Liu

**Affiliations:** 1 School of Biomedical Engineering, Guangzhou National Laboratory, Guangzhou Medical University, Guangzhou, China; 2 College of Biological Science, China Agricultural University, Beijing, China; 3 GMU-GIBH Joint School of Life Sciences, Guangzhou Medical University, Guangzhou, China; 4 School of Basic Medical Sciences, Guangdong Medical University, Dongguan, China; 5 School of Biomedical Sciences and Engineering, South China University of Technology, Guangzhou, China; 6 Guangdong Key Provincial Laboratory of Allergy & Clinical Immunology, The Second Affiliated Hospital, Guangzhou Medical University, Guangzhou, China; 7 The Guangdong-Hong Kong-Macao Joint Laboratory for Cell Fate Regulation and Diseases, Guangzhou National Laboratory, GMU-GIBH Joint School of Life Sciences, Guangzhou Medical University, Guangzhou, China; 8 State Key Laboratory of Respiratory Disease, National Clinical Research Center for Respiratory Disease, Guangzhou Institute of Respiratory Health, The First Affiliated Hospital of Guangzhou Medical University, Guangzhou, China

**Keywords:** Stem-cell differentiation, Biomaterials - cells, Stem-cell research

## Abstract

Regional epithelial lineages of the human respiratory system reside within an extracellular matrix (ECM) whose mechanics vary along the airway–alveolar axis, yet how ECM stiffness directs epithelial fates remains unclear. Here, utilizing human pluripotent stem cell-derived lung organoids embedded in stiffness-tunable hydrogels as an in vitro model, we show ECM stiffness governs region-specific epithelial differentiation. Stepwise softening of ECM stiffness yields airway organoids with proximal-to-distal airway epithelial compositions and biomimetic physiological functions. During alveolar differentiation, increased stiffness promotes alveolar type 2 (AT2) and type 1 (AT1) maturation and drives AT2-to-AT1 transition. Furthermore, RNA sequencing reveals ECM stiffness regulates epithelial fates primarily through mechanotransduction pathways. Finally, these organoids reproduce the infection tropisms of SARS-CoV-2 variants. Together, this research elucidates ECM stiffness as a critical determinant of epithelial cell fate specification and region-specific lung organoid generation, which offers a valuable in vitro model for studying region-specific lung development, diseases pathogenesis, and drug screening.

## Abstract (web summary)

Using human pluripotent stem cell-derived lung organoids, the authors show that extracellular matrix stiffness directs region-specific lung differentiation, revealing a key mechanical cue that guides human regional lung specification.

## Introduction

The physical properties of the extracellular environment, particularly its mechanical stiffness^1^, play crucial roles in guiding tissue morphogenesis and determining cell fate^2,3^. Extracellular matrix (ECM) stiffness varies significantly across different tissues, ranging from soft, pliable brain tissue with an elastic modulus in the subkilopascal (kPa) range to rigid, mineralized bone with stiffness values reaching hundreds of megapascal (MPa)^2^. These diverse mechanical environments shape cells to adapt and function optimally within their native microenvironment. Stem cell-derived organoids serve as ideal models for studying the impact of the microenvironment on cell lineage specification. For example, mesenchymal stem cells differentiate into neurogenic, myogenic, or osteogenic lineages depending on the mechanical stiffness of the culture substrate^4^. Similarly, the modulation of matrix stiffness impacts brain organoid growth, cellular differentiation, and spatial organization^5^. Collectively, these findings underscore the importance of ECM mechanics in directing tissue-specific development.

The human respiratory tract is a highly dynamic, multicellular system composed of epithelial, endothelial, mesenchymal, and immune cells that coordinate to maintain structural integrity and respiratory function^6^. Although these multicellular interactions are essential, epithelial cells are central to executing key respiratory functions and are frequently implicated in the pathogenesis of lung diseases^7^. Early stages of lung development, including morphogenesis, vasculogenesis, and alveolarization, are closely associated with regional changes in ECM composition and stiffness^8^, suggesting that ECM mechanics may instruct spatially patterned epithelial differentiation. Epithelial subtypes exhibit region-specific, proximal-to-distal distributions within the respiratory tract. For example, the airway epithelium comprises a variety of cell types, with goblet and ciliated cells enriched in the proximal airway^9^, whereas club cells (SCGB1A1^+^) and SCGB3A2^+^ secretory cells are predominant in the distal regions^10,11^. Within the alveolar space, alveolar epithelial type 2 (AT2) and type 1 (AT1) cells constitute the major epithelial populations. However, the underlying mechanism governing epithelial cell fate decisions and their spatial organization remains largely unelucidated.

The ECM constitutes the immediate microenvironment in which respiratory epithelial cells reside^12^. In the proximal respiratory airways, increased ECM stiffness is essential for maintaining the tubular structure necessary for air conduction. The conducting airway is a hierarchical, tree-like branching structure that extends from the proximal trachea to the distal bronchioles along the proximal-to-distal axis^13^. Mechanical rigidity of trachea and bronchi is reinforced by cartilage rings and smooth muscle layers, resulting in a thick and stiff ECM^13^. As the airway tree branches from larger conducting airways to smaller distal bronchioles, the ECM gradually becomes thinner and softer, accompanied by the loss of cartilage support^14^. Therefore, the stiffness of the ECM along the air-conducting airway exhibits a gradient from stiff proximally to soft distally. The ECM surrounding alveolar epithelia is both soft and highly elastic^15^. Alveolar epithelial development begins at the late canalicular stage (~24 weeks of gestation) and continues until early childhood (~8 years of age)^16^. During the saccular and alveolar stages, myofibroblasts deposit fibronectin, elastin and collagen IV, forming a compliant but highly elastic ECM that supports epithelial morphogenesis^17^. After birth, biomechanical forces generated by respiratory motion activate the Hippo-YAP/TAZ signaling pathway for maintaining AT1 cell identity, whereas the loss of biophysical forces can drive AT1 reprogramming toward AT2^18,19^. An appropriate level of ECM elasticity is also critical for secondary septation (the formation of mature alveoli) and coordination with respiratory motion through regulation of downstream pathways, among which YAP/TAZ mediated mechanical tension induced pulmonary alveolar regeneration^20^. Overexpression of activated nuclear YAP is sufficient to drive transcriptomic shift of iAT2 to iAT1-like cells, without ECM mechanical forces involvement^21^. Although mechanical force, including ECM stiffness, elasticity, and loading, all of these may act differently, the underlying mechanisms were cross over e.g., focal adhesion kinase (FAK), Hippo, integrin signaling pathways]^22^. In summary, the mechanical property of alveolar ECM is soft and elastic; proper mechanical force is critical for mature alveolar formation and cooperative respiratory movement. Excessive hydrogel stiffness suppresses embryonic lung bud (E10.5–E12.5) morphogenesis, emphasizing the critical role of mechanical cues in guiding lung patterning and morphogenesis^23^. However, whether spatially heterogeneous ECM stiffness suggests that localized mechanical gradients guide region-specific lung development and its underlying molecular mechanisms remains unclear.

Human pluripotent stem cell (hPSC)-derived lung organoids have been widely used to investigate lung development^24^. These organoids are typically embedded in Matrigel, which provides 3D structural support^25^. However, Matrigel is a basement membrane extract from Engelbreth–Holm–Swarm mouse sarcoma and offers limited mechanical tunability^26^, which constrains its ability to replicate the complex mechanical properties of the native respiratory microenvironment. Available data indicate that region-specific lung stiffness typically ranges from 0.5 to 15 kPa^27^. Mechanically tunable hydrogels enable precise modulation of ECM stiffness, thereby facilitating controlled organoid differentiation^5^. Gelatin methacryloyl (GelMA), a mechanically tunable alternative to Matrigel, has been successfully applied in a variety of organoid systems, including hPSC-derived kidney organoids^28^, intrahepatic cholangiocyte organoids^26^ and patient-derived breast cancer organoids^29^. Its tunable stiffness range (0.2–40 kPa) effectively spans the physiological stiffness spectrum of the respiratory system^30^. Moreover, hPSC-derived lung organoids frequently exhibit heterogeneous and disorganized epithelial compositions, with significant batch-to-batch variability^31^. This cellular disorganization fails to recapitulate the proximodistal epithelial patterning observed in the native lung. Previous studies have shown that manipulating the extracellular microenvironment through the addition of small molecules^32^, the incorporation of specific ECM components^33^, or coculture with region-specific fibroblasts^34^ can influence epithelial cell fate. These findings underscore that developing lung epithelial cells are capable of sensing and responding to extracellular cues, thereby undergoing fate transitions in accordance with their local microenvironment.

In this work, we utilized a stiffness-tunable GelMA hydrogel system to provide a 3D culture environment for hPSC-derived lung organoids, aiming to investigate the role of ECM stiffness in region-specific respiratory epithelial cell development. Our results reveal that stiffness directs lung epithelial cell fate and elucidate its underlying mechanism. Region-specific infection assays with SARS-CoV-2 variants (Omicron and Delta) further recapitulated proximal-to-distal epithelial patterning. This study highlights the impact of ECM stiffness on lung epithelial cell fate, offering a platform to generate region-specific lung organoids from the proximal-to-distal direction to closely mimic the lung in vivo proximal-to-distal respiratory system.

## Results

### High extracellular stiffness promotes LPC specification

Three different stiffnesses of GelMA hydrogels were prepared with different degrees of methacrylation (MA, 30%, 60%, and 90%) and consistent gelatin concentration (8% w/v) in the presence of lithium phenyl-2,4,6-trimethylbenzoylphosphinate (LAP, 0.25%) and a constant ultraviolet (UV) light exposure time of 30 s (Supplementary Fig. 1a, denoted as G30, G60, and G90, respectively), as described in previous reports^35^. Rheological tests (Supplementary Fig. 1b) and compression testing confirmed that the stiffness gradually increased from G30 to G90, yielding compressive moduli of 2.99 ± 0.24, 6.22 ± 0.65, and 11.31 ± 0.12 kPa, respectively (Supplementary Fig. 1c, d). Swelling ratio and scanning electron microscopy further revealed reduced water restrained property (227 ± 1.77%, 158 ± 3.95% and 116 ± 1.34%, *P* < 0.001, Supplementary Fig. 1e) and pore size (304 ± 46.3 μm^2^, 169 ± 34.2 μm^2^, and 119 ± 23.1 μm^2^, *P* < 0.001, Supplementary Fig. 1f, g) with increasing stiffness. Enzymatic degradation assays using collagenase II demonstrated stiffness-dependent stability in which G30 exhibited the fastest degradation, and G90 exhibited the slowest (Supplementary Fig. 1h, i). Collectively, these data establish a well-controlled GelMA series that provides a robust stiffness gradient (2.99–11.31 kPa) with coordinated swelling, pore architecture, and degradability, well-suited for probing how extracellular stiffness regulates hPSC-derived lung progenitor cells (LPCs) and human lung organoids (HLOs) differentiation.

**Fig. 1:**
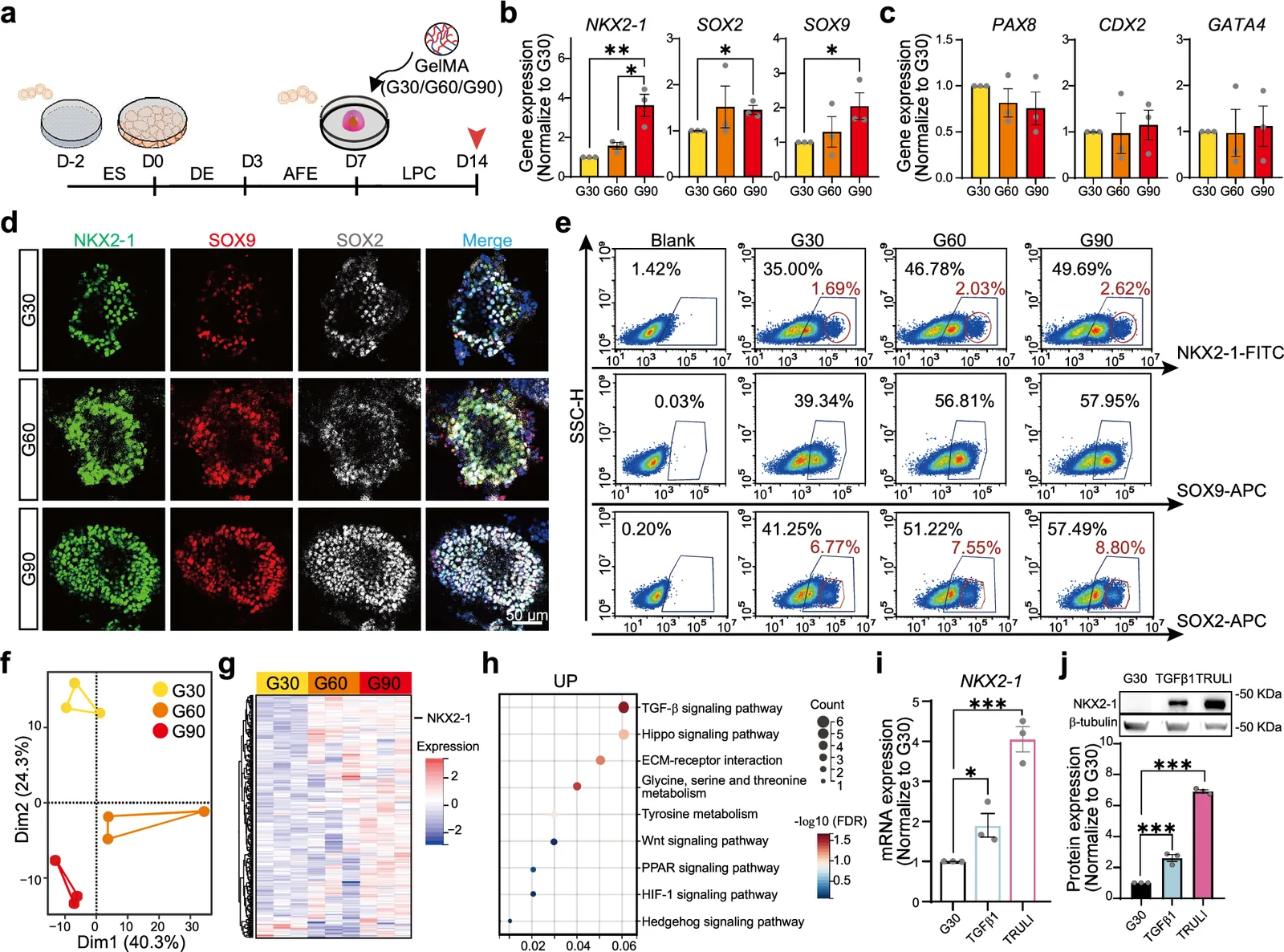
High stiffness promotes LPCs generation by activating the TGF-β and Hippo pathways. **a** Schematic of the directed differentiation protocol from hESCs to LPCs. Self-assembled AFE spheroids were collected on day 7 and encapsulated in GelMA droplets. ES, embryonic stem cells; DE, definitive endoderm; AFE, anterior foregut endoderm; LPCs, lung progenitor cells. Red arrowhead indicates the samples collection time. G30/G60/G90, GelMA hydrogels with low to high stiffness. **b** Fold changes in mRNA expression of lung lineage-related genes relative to G30: *NKX2-1* (lung progenitor), *p* = 0.0041, 0.0132; *SOX2* (airway progenitor), *p* = 0.0113; and *SOX9* (alveolar progenitor), *p* = 0.0298. **c** Fold changes in mRNA expression of non-lung lineage genes relative to G30: *PAX8* (thyroid marker), *CDX2* (intestinal marker), *GATA4* (neuronal marker). **d** Representative immunofluorescence (IF) whole-mount images of lung lineage markers: NKX2-1 (green), SOX9 (red), and SOX2 (white) in LPCs differentiated in G30, G60, and G90. **e** Representative flow cytometry profiles of NKX2-1, SOX2 and SOX9 positive cells in LPCs under varying stiffness. Blue polygon indicates positive cell population; red circle represents the strongly positive cells. **f** Principal component analysis (PCA) of bulk RNA sequencing data from LPCs cultured in G30 (yellow), G60 (orange) and G90 (red). **g** Heatmap of significantly upregulated genes, highlighting increased *NKX2-1* expression with gradient hydrogel stiffness. **h** KEGG pathway enrichment analysis of upregulated genes. **i** *NKX2-1* gene expression in LPCs treated with TGFβ1 (a TGF-β pathway agonist) and TRULI (a nuclear YAP activator), normalized to G30, *p* = 0.0382 and *P* < 0.001. **j** WB analysis of NKX2-1 protein levels treated with TGFβ1 and TRULI, *P* < 0.001, <0.001. For all statistical plots, data are presented as mean ± SEM, with distinct dots representing individual values from three independent biological experiments. *p* values were calculated using *t*-test (middle and right graph from **b**; **i**), one-way ANOVA with Tukey’s multiple comparison test (left graph from **b**; **c**) or Dunnett’s multiple comparison test (**j**). *P < 0.05, **P < 0.01, ***P < 0.001.

Efficient hPSC differentiation into NKX2-1^+^ LPCs (also known as thyroid transcription factor 1, TTF1) is essential for subsequent high-quality generation of human airway organoids (hAWOs) and alveolar organoids (hALOs)^11^. Early SOX2 and SOX9 expression orchestrates branching morphogenesis, with SOX2 maintaining proximal multipotent progenitors and SOX9 predominant in the distal lung^36^. In this study, hPSCs were differentiated into definitive endoderm (DE) and anterior foregut endoderm (AFE) spheroids and then encapsulated AFE spheroids in GelMA hydrogels of defined stiffness (G30, low; G60, intermediate; G90, high) to investigate stiffness-mediated LPC specification (Fig. 1a; Supplementary Fig. 2a). Self-assembled AFE spheroids remained viable and generated luminal, multicellular organoids across all stiffness hydrogels (Supplementary Fig. 2b, c). However, direct encapsulation of single cells in G90 did not support survival or organoid formation (Supplementary Fig. 2d). In contrast, in G90, self-assembled AFE spheroids grew with time and progressively formed multicellular structures (Supplementary Fig. 2d). Consistent with prior work^37^, once multicellular aggregates are established under permissive conditions, subsequent culture in stiffer matrices can modulate cell fate without compromising survival. Moreover, the extracts from Matrigel and GelMA (G30, G60 and G90) were applied to LPCs embedded in Matrigel to assess viability and differentiation (Supplementary Fig. 2e). CCK8 assays and *NKX2-1* gene expression analysis showed the extracts did not affect LPC viability or *NKX2-1* levels (Supplementary Fig. 2f, g), indicating that residual unreacted components (if any remained) do not impact LPC viability or differentiation capacity.

Hydrogel stiffness significantly increased *NKX2-1, SOX2*, and *SOX9* expression (Fig. 1b) but did not upregulate the expression of nonlung lineage markers such as *PAX8* (thyroid), *CDX2* (intestine), and *GATA4* (neuron) (Fig. 1c). Immunofluorescence (IF) confirmed increased NKX2-1 expression in G90 LPCs with strong colocalization of SOX2 and SOX9 (Fig. 1d). Flow cytometry revealed a stiffness-dependent increase in the number of NKX2-1^+^ LPCs, which increased from 35.00% in G30 to 46.78% in G60 and 49.69% in G90 (Fig. 1e). Similarly, the percentage of SOX9^+^ LPCs increased from 39.34% in G30 to 57.95% in G90, whereas the percentage of SOX2^+^ LPCs increased from 41.25 to 57.49%. Notably, compared with G30 (1.69% and 6.77%, respectively), G90 contained a significantly greater percentage of strongly NKX2-1^+^ (2.62%) and SOX2^+^ (8.80%) cells (Fig. 1e; highlighted in red circle). LPC growth, proliferation, and apoptosis were not affected by increased stiffness (Supplementary Fig. 3a–h). The stiffness of the GelMA hydrogels was tuned exclusively by varying the degree of methacryloyl (MA) substitution, but keeping the GelMA concentration the same. Previous studies similarly reported no significant effect of stiffness on proliferation when the GelMA concentration remains the same^38^.

Bulk RNA sequencing (RNA-seq) of LPCs cultured under different stiffnesses revealed distinct transcriptional profiles (Fig. 1f), with *NKX2-1* expression increasing consistently with stiffness (Fig. 1g). Cell cycle and replication genes were comparable across G30, G60, and G90 (Supplementary Fig. 3i), indicating minimal effects of varying stiffness on cell proliferation status. Pathway enriched in upregulated genes from G60 vs. G30, G90 vs. G60, and G90 vs. G30 were primarily associated with LPC differentiation [transforming growth factor beta (TGF-β), wingless/int-1 (Wnt), sonic hedgehog (SHH)], ECM mechanics [Hippo, peroxisome proliferator-activated receptor (PPAR) and hypoxia] and metabolism (glycine, serine, threonine and tyrosine) (Fig. 1h). The functional activation of TGF-β with TGFβ1 and nuclear Yes-associated protein (YAP) with TRULI in soft hydrogels (G30) increased NKX2-1 expression (Fig. 1i, j), recapitulating the high-stiffness phenotype. These functional rescue experiments demonstrated that the stiffness-driven increase in NKX2-1^+^ LPCs is mediated, at least in part, by TGF-β and Hippo signaling. Taken together, these data support a model in which increased ECM stiffness promotes LPC specification through modulation of the TGF-β and Hippo pathways, effects that are reproducible by pharmacological activation under low-stiffness conditions.

### Physiological validation of proximal-to-distal hAWOs specified in stiff-to-soft hydrogels

**Fig. 2:**
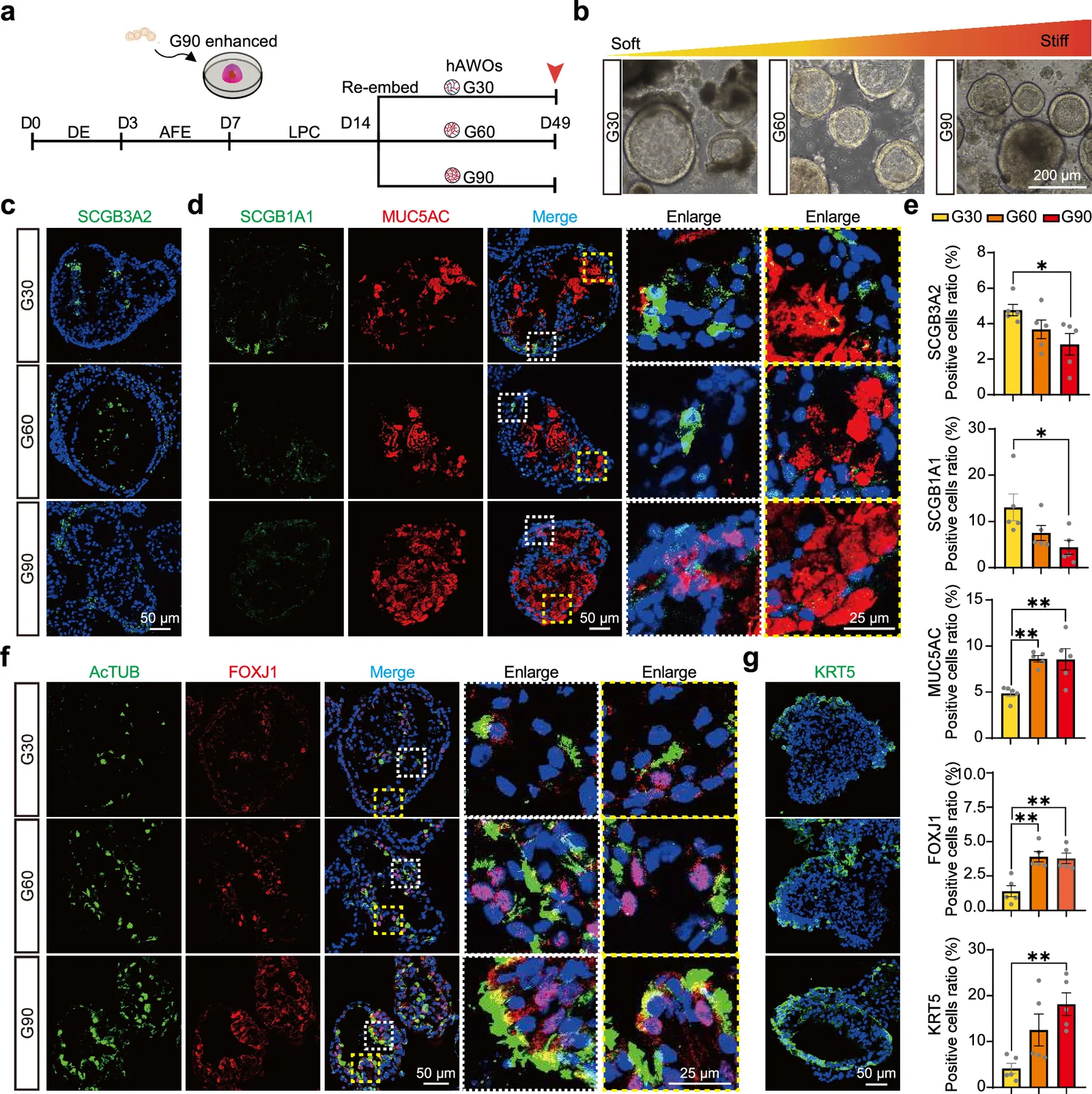
ECM stiffness directs proximal-to-distal specification in hAWOs. **a** Schematic of the directed differentiation protocol for hAWOs modulated by ECM stiffness. AFE spheroids were embedded in G90 to enhance NKX2-1 expression. On day 14, LPCs were re-embedded in hydrogels with varying stiffness (G30, G60, G90) and analyzed on day 49. Red arrow indicates the time to collect the samples. **b** Representative bright-field images of hAWOs cultured in G30, G60 and G90 (*n* = 3). **c** Representative IF staining for SCGB3A2 (green) in hAWOs cultured under G30, G60 and G90. **d** Representative IF staining for SCGB1A1 (green), MUC5AC (goblet cell, red) in G30, G60, and G90 hAWOs. Merged images highlight regional colocalization. White dotted box highlighting SCGB1A1, yellow dotted box highlighting MUC5AC. **e** Quantification of positive cells expressing SCGB3A2, *p* = 0.0453; SCGB1A1, *p* = 0.0329; MUC5AC, *p* = 0.0087, 0.0097; FOXJ1 (ciliated cell), *p* = 0.0017, 0.0025; and KRT5 (basal cell), *p* = 0.0057, under varying stiffness (G30, G60, G90) (*n* = 5 organoids from one biological replicates; data from two additional biological replicates are provided in the Source data file). **f** Representative IF staining for AcTUB (multiciliated cell, green) and FOXJ1 (red) in G30, G60, and G90 (*n* = 3). White dotted box highlighting AcTUB, yellow dotted box frame highlighting FOXJ1. **g** Representative IF staining for KRT5 (green) in hAWOs cultured under G30, G60, and G90 (*n* = 3). Data are presented as mean ± SEM. *n* = 3 independent biological replicates. Statistical analyses were performed using one-way ANOVA with Tukey’s multiple comparison test (**e**). *P < 0.05, **P < 0.01, ***P < 0.001.

HAWOs derived from LPCs typically display heterogeneous, randomly mixed epithelial populations^39^, unlike the organized proximal-to-distal patterning of airway epithelial cells observed in vivo^9^. Growth factor modulation is insufficient to control the cell composition, spatial arrangement and heterogeneity of hAWOs^40^. Notably, the native respiratory airway exhibits a stiff-to-soft ECM gradient across regions^41^, inspiring us to explore whether stiffness cues direct airway epithelial cell fate and spatial distribution. To this end, LPCs were encapsulated in GelMA hydrogels of defined stiffness (G30, G60, and G90) and differentiated into hAWOs (Fig. 2a). By Day 49, lumenized multicellular organoids had formed and were collected for further analysis (Fig. 2b). IF and flow cytometry revealed that stiffness produced a distinct stiffness-dependent cell fate pattern. SCGB1A1^+^ secretory cells and SCGB3A2^+^ secretory cells were enriched in soft G30 hydrogel (Fig. 2c–e; Supplementary Fig. 4a). In contrast, goblet cells (MUC5AC^+^) were more abundant in the stiff G90 group (Fig. 2d, e; Supplementary Fig. 4b). Ciliated cells (AcTUB^+^ and FOXJ1^+^) were most prominent in the G60 and G90 hydrogels (Fig. 2e, f; Supplementary Fig. 4c). Basal cells (KRT5^+^) were enriched in G90 (Fig. 2e, g; Supplementary Fig. 4d). The cellular composition of hAWOs derived under high-to-low stiffness indicated G90-hAWOs contained more ciliated, goblet and basal cells, but fewer SCGB3A2^+^ and SCGB1A1^+^ secretory cells. Whereas G30-hAWOs contained more SCGB3A2^+^ and SCGB1A1^+^ secretory cells but fewer ciliated, goblet and basal cells. These data revealed that tuning ECM stiffness biased epithelial composition of hAWOs toward region-specific epithelial distributions, yielding proximal-like hAWOs in G90, bronchiolar-like organoids in G60, and distal-airway-like organoids in G30.

To validate the physiological relevance of the stiffness-guided, region-specific hAWOs, we performed established functional assays for mucociliary defense^42,43^ and epithelial ion and fluid transport capacity^9,44^. Since the major differences among G30-, G60-, and G90-hAWOs were the distribution of ciliated cells and secretory lineages (e.g., goblet- and ciliated-enriched features in G90-hAWOs versus SCGB1A1/SCGB3A2-secretory cells enriched in G30-hAWOs), we assessed ciliary motility and mucociliary transport to evaluate cilia-related function and forskolin-induced swelling (FIS), which is related to secretory cell function.

**Fig. 3:**
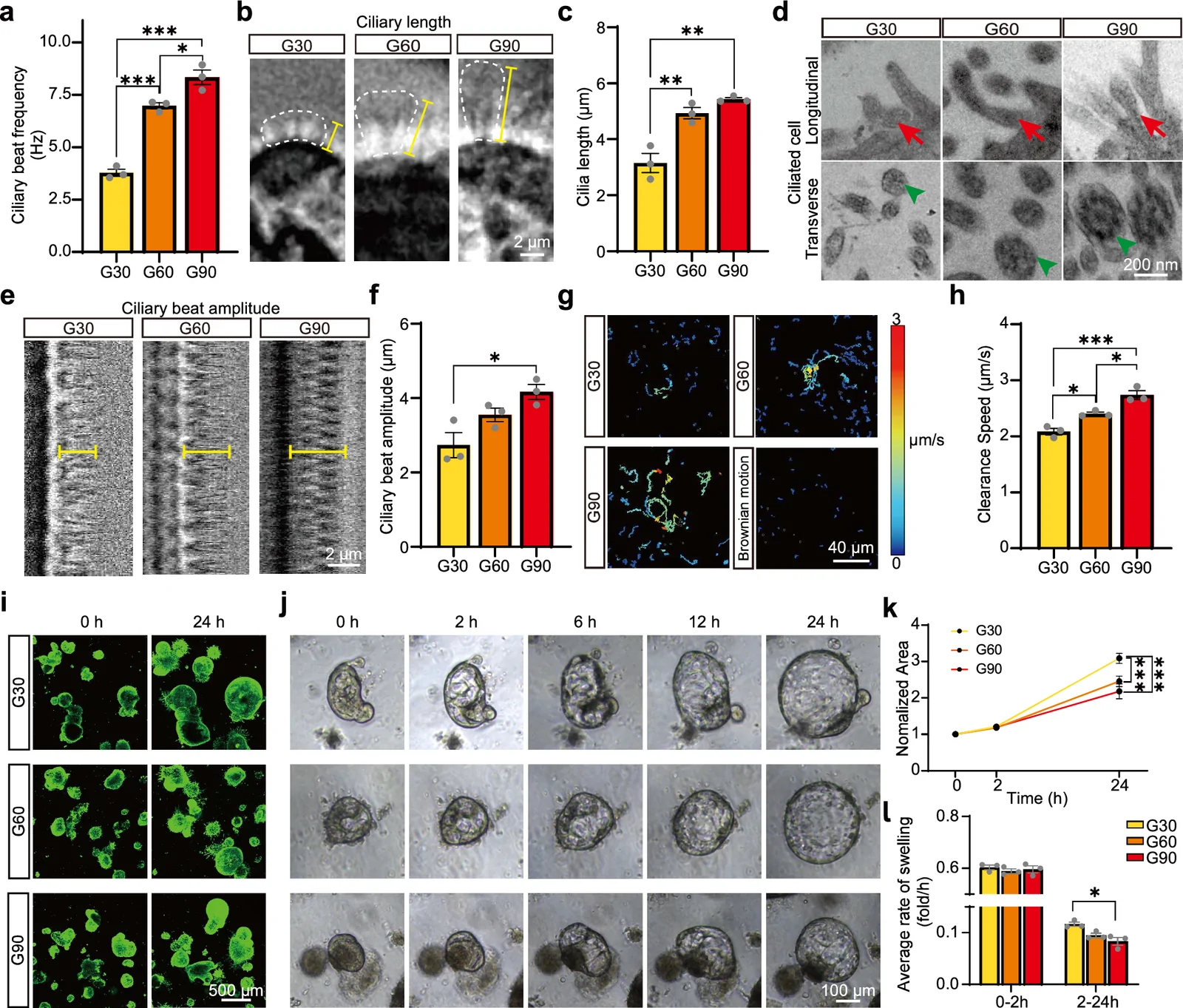
Region-specific differences in ciliary function and forskolin-induced swelling of hAWOs differentiated under varying ECM stiffness. **a** Quantification of average ciliary beat frequency (CBF) in hAWOs (*n* = 3), *p* = 0.0021, <0.001, <0.001. **b** Representative bright-field images of cilia in hAWOs. White dashed outlines denote the ciliary region, and yellow lines indicate representative ciliary length measurements. (Scale bar: 2 μm). **c** Quantification of ciliary lengths of G30-, G60- and G90-hAWOs (*n* = 3), *p* = 0.0011, 0.0041. **d** TEM images of ciliated cells under G30, G60, and G90 in longitudinal sections (red arrow) and in transverse sections (green triangle). **e** Analysis of ciliary beat amplitude (CBA) based on kymograph span. Yellow lines indicate the lateral span of ciliary movement used to quantify CBA. (Scale bar: 2 μm). **f** Quantification of CBA of G30-, G60- and G90-hAWOs (*n* = 3), *p* = 0.0155. **g** Stacked images of the fluorescent beads placed in the culture medium with hAWOs and Brownian motion of the beads without exposure to the hAWOs. The movies were recorded for 20 s. Color spectrum reflected the time course. **h** The clearance speed of hAWOs calculated from at least 30 trajectories of the fluorescent beads per sample in each hAWOs (*n* = 3), *p* = 0.0304, 0.0417, <0.001. **i** Fluorescence microscopy of live hAWOs (calcein green staining) at 0 h and 24 h after forskolin treatment. (Scale bar: 500 μm). **j** Live imaging of hAWOs recorded at 0, 2, 6, 12, and 24 h post-forskolin treatment. **k** Quantification of normalized organoid swelling area at 0, 2, and 24 h post-forskolin treatment. The bright-field area of each well at 0 h was set to 1 (*n* = 3), *P* < 0.001, <0.001. **l** The average rate of swelling was calculated for the first 2 h and substituted 22 h to reflect instant and long-term response (*n* = 3), *p* = 0.0416. Data are presented as mean ± SEM. Fields of view were recorded from three biological replicates. Statistical analyses were performed using one-way ANOVA (**a**, **c**, **f**, **h**) or two-way ANOVA (**k**, **l**) with Tukey’s multiple comparison test. *P < 0.05, **P < 0.01, ***P < 0.001.

Physiologically, motile cilia provide a critical airway defense barrier by rhythmically beating to propel mucus and trapped pathogens toward the pharynx. Our results indicated ciliary beat frequency (CBF) (Supplementary Movies 1–3 and Fig. 3a) was significantly increased in G90-AWOs (8.34 ± 0.35 Hz) compared with G30 (3.78 ± 0.16 Hz) and G60-AWOs (6.97 ± 0.15 Hz). The cilia on G90-hAWOs (5.43 ± 0.06 µm) and G60-hAWOs (4.93 ± 0.20 µm) were also significantly longer than those in G30 (3.15 ± 0.35 µm) (Fig. 3b, c). Transmission electron microscopy (TEM) analysis was used to examine intracellular ultrastructural features of organelles. Ultrastructure of the cilia in G90 appeared longer and denser in longitudinal sections (red arrow, Fig. 3d), and exhibited a canonical 9 + 2 axonemal architecture (nine doublets surrounding a central pair), indicating the presence of motile, functionally mature cilia (green triangle, Fig. 3d). With increasing stiffness, the goblet cells in G90 displayed more numerous and larger mucus vesicles (blue triangle, Supplementary Fig. 4e). Together, cilia and goblet cells contribute to functional mucociliary clearance. Additionally, G90-hAWOs exhibited the largest ciliary beat amplitude (CBA) (4.17 ± 0.20 µm; Fig. 3e, f), likely reflecting their higher CBF and longer cilia (Fig. 3a–d). To measure mucociliary transport, fluorescent beads were tracked (Supplementary Movies 4–6 and Fig. 3g). Functionally, G90-hAWOs showed the highest particle clearance speed (2.74 ± 0.076) compared with G60-hAWOs (2.40 ± 0.031) and G30-hAWOs (2.06 ± 0.058) (Fig. 3h). Together, these data indicate that region-specific hAWOs demonstrate a more robust mucociliary clearance phenotype in G90-hAWOs which show greater coverage of motile cilia and goblet cells mimicking proximal airways, compared with the lower cilia coverage mimicking distal airways in G60- and G30-hAWOs.

FIS is widely used to assess cAMP-dependent anion or fluid transport and functional maturation of airway secretory epithelium. The hAWOs from G30, G60, and G90 all showed significant swelling in response to forskolin treatment after 24 h (Fig. 3i, j) with 3.09-, 2.45- and 2.18-fold increases, respectively (Fig. 3k). The average swelling rates of G30-, G60- and G90-hAWOs were 0.603, 0.589, and 0.596-fold/h, respectively, in the first 2 h, and 0.116, 0.095, 0.083-fold/h, respectively, over the subsequent 22 h (Fig. 3l), indicating functional epithelial fluid secretion capacity. Although the early responses were comparable among the three groups, swelling divergence became more evident over time, with G90-hAWOs displaying significantly less swelling in the normalized area than G30-hAWOs. Taken together, by modulating ECM stiffness, we can obtain hAWOs with different airway epithelial cell compositions with proximal-like mucociliary defense properties versus more distal-small airway-like FIS response, thereby providing region-specific-mimic hAWOs for modeling regional-specific airway physiology and disease.

### ECM stiffness directs hAWOs cell fate via the Hippo/hypoxia/Wnt

**Fig. 4:**
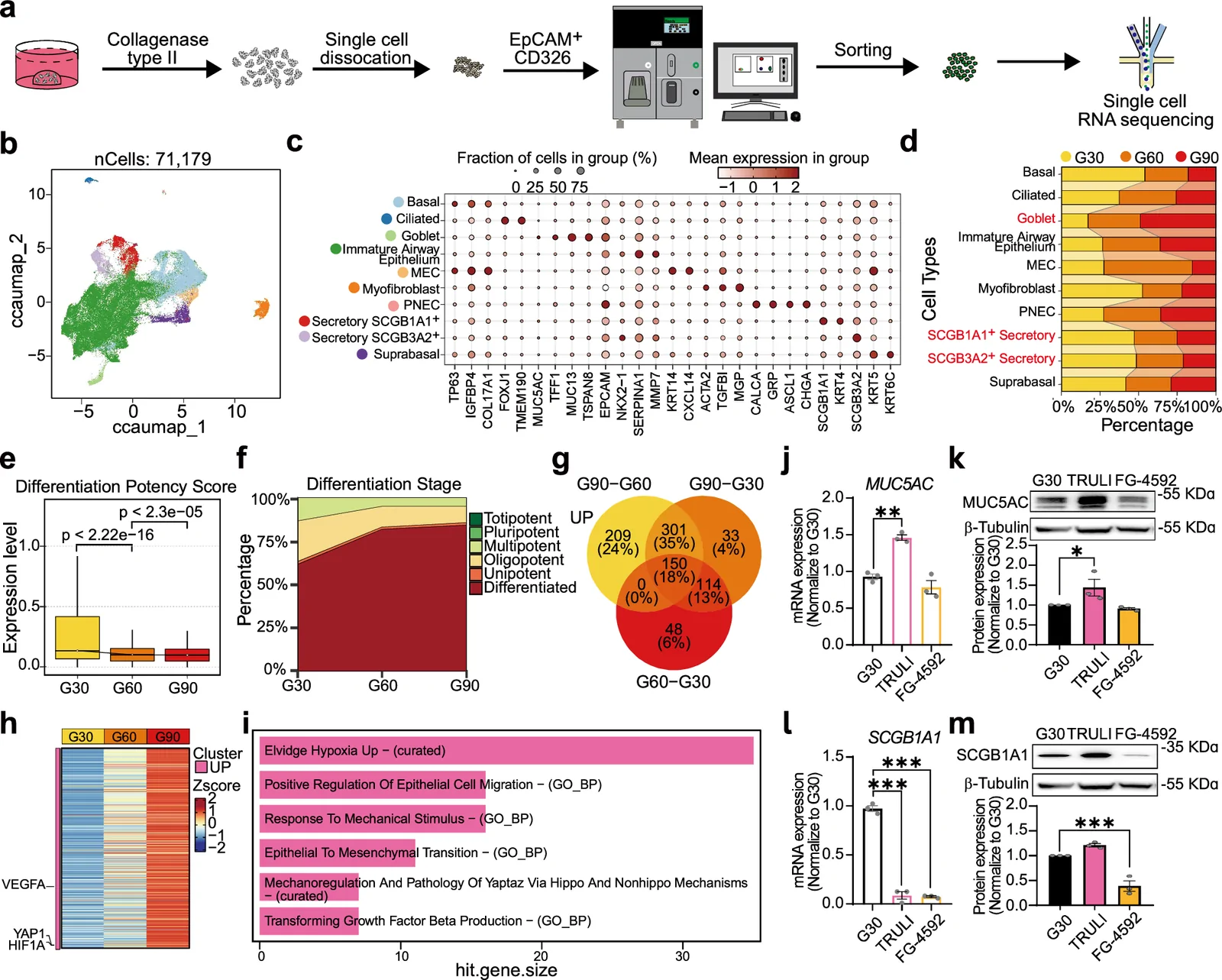
scRNA-seq reveals ECM stiffness directs airway epithelial cell fate via regulating YAP1 and HIF1A. **a** Workflow for scRNA-seq analysis of hAWOs. Cells were released from GelMA using collagenase type II, then dissociated into single cells with TrypLE, enriched via EpCAM^+^ sorting, and subjected to scRNA-seq. **b** Uniform Manifold Approximation and Projection (UMAP) visualization of 71,179 cells from hAWOs differentiated under G30, G60 and G90 across three biological replicates grouped into 10 distinct clusters based on curated cell type markers. **c** Dot plot showing marker gene expressions across 10 cell clusters. The size of the dots indicates the fraction of cells expressing the gene, and the color of the dots represents the mean gene expression. **d** Stacked bar chart displaying the distribution of cell types across various stiffnesses of G30, G60, and G90. **e** Differentiation potency scores calculated using CytoTRACE2, indicating reduced differentiation potency with increased stiffness. The center line indicates the median, box bounds indicate the 25th and 75th percentiles, and whiskers indicate the minimum and maximum values. Statistical significance was determined by pairwise comparison (*n* = 71,179 cells). Exact *P* values are shown in the figure. **f** Predicted absolute differentiation stages based on scRNA-seq data, categorized as Totipotent, Pluripotent, Multipotent, Oligopotent, Unipotent, and Differentiated. **g** Venn diagram showing upregulated genes shared across low to high stiffness. Overlapping regions highlight genes shared between every two groups. **h** Heatmap showing gene expression profiles of key genes regulated by higher stiffness. **i** Pathway enrichment analysis of upregulated genes using GO-BP and curated databases, highlighting pathways such as hypoxia, TGF-β, and Hippo signaling. MUC5AC expression analysis via qPCR (**j**) (*p* = 0.0016) and WB (**k**) (*p* = 0.0414) in G30 organoids treated with TRULI and FG-4592 (HIF1A stabilizer). SCGB1A1 expression analysis via qPCR (**l**) (*P* < 0.001, <0.001) and WB (**m**) (*P* < 0.001) in G30 organoids treated with TRULI and FG-4592. Data are presented as mean ± SEM. *n* = 3 independent biological replicates. Statistical significance was determined by one-way ANOVA with Dunnett’s multiple comparisons (**j**–**m**). *P < 0.05, **P < 0.01, ***P < 0.001.

To elucidate how ECM stiffness influences epithelial differentiation in hAWOs, single-cell RNA sequencing (scRNA-seq) was performed on hAWOs with varying stiffnesses (Fig. 4a). To enrich for epithelium, epithelial cell adhesion molecule positive (EpCAM^+^) cells were prospectively sorted prior to sequencing (Supplementary Fig. 5). Three biological replicates from each stiffness group (G30, G60 and G90) were analyzed, and the datasets were integrated for scRNA-seq analysis and figure generation (Supplementary Fig. 6a). The cells were categorized into epithelial and mesenchymal lineages (Fig. 4b; Supplementary Fig. 6b, c), with 97.1% epithelial cells. A small mesenchymal admixture (2.9%) remained, likely due to mesenchymal‒epithelial interactions during lung development. Previous studies with hiPSC-HLOs have also detected residual mesenchyme from scRNA-seq datasets^25,45^. The cells from three biological replicates were classified into 10 transcriptionally distinct subtypes (Fig. 4c; Supplementary Fig. 6d, e). Goblet cells were predominantly enriched in G90, whereas SCGB1A1^+^ and SCGB3A2^+^ secretory cells were more abundant in G30 (Fig. 4d; Supplementary Fig. 7a–f), which is consistent with the IF results (Fig. 2).

To determine the anatomical relevance of our hAWOs cultured under various stiffness, we performed a reference-based mapping and annotation analysis, referencing the datasets of the developing human airway atlas spanning the trachea, small airway and distal lung (Supplementary Fig. 8a)^46^. The reference map generated from trachea, small airway and distal lung regions anatomically defined fetal epithelial populations (Supplementary Fig. 8b). We then mapped our scRNA-seq data from hAWOs with different stiffness onto the referenced map. The integrated map of our stiffness-guided hAWOs indicated that the majority of cells correspond to epithelial populations of the trachea (35,273 cells) and small airways (29,457 cells), with only a minor fraction mapping to distal lung lineages (4403 cells) (Supplementary Fig. 8c). Finally, we quantitatively compared the predicted tissue position labels of organoids across different stiffness groups which also indicated more trachea-like G90-hAWOs, more small airway-like-G30-hAWOs (Supplementary Fig. 8d). Together, these results indicate that our ECM-stiffness-guided hAWOs primarily model the conducting airway epithelium spanning the trachea to small-airway regions.

Differentiation potency analysis revealed the lowest scores in G90 (Fig. 4e), with approximately 70% of cells classified as terminally differentiated, indicating advanced maturation under high-stiffness conditions (Fig. 4f). Under stiffness conditions, 565 overlapping upregulated genes were identified, 150 of which were commonly upregulated, including *VEGFA*, *YAP1* and *HIF1A* (Fig. 4g, h). Enriched pathways included hypoxia, epithelial migration, mechanical stiffness response, epithelial-to-mesenchymal transition (EMT), the TGF-β, and Hippo signaling pathways (Fig. 4i), whereas 230 overlapping downregulated genes were associated with cell metabolism and Wnt signaling pathways (Supplementary Fig. 7g–i). Consistent with a differentiation effect rather than a proliferation effect, no significant differences in the S Score, G2M Score, *MKI67*, or *TOP2A* were noted across G30, G60 and G90 (Supplementary Fig. 7j).

To functionally investigate hypoxia and Hippo signaling, we treated G30 hAWOs with FG-4592 (a HIF1A stabilizer) and TRULI (a nuclear YAP activator). TRULI significantly increased MUC5AC expression by 1.5-fold, whereas FG-4592 alone had no effect (Fig. 4j, k). Interestingly, the combination of TRULI and FG-4592 significantly increased MUC5AC expression by 4-fold (*P* < 0.001) (Supplementary Fig. 7k). Both TRULI and FG-4592 suppressed *SCGB1A1* and *SCGB3A2* expression (*P* < 0.001) by 5- and 10-fold, respectively, whereas the combination resulted in more pronounced 50- and 25-fold decreases, respectively (Fig. 4l; Supplementary Fig. 7k, l). Western blot (WB) analysis confirmed that FG-4592 reduced SCGB1A1 protein levels (Fig. 4m). These data indicate that the synergistic action of Hippo signaling and hypoxia signaling more effectively directs hAWOs epithelial cells toward the proximal airway lineage. Wnt pathway activation with CHIR-99021 in G30 cells suppressed *MUC5AC* (*P* < 0.01) but increased *SCGB3A2* (*P* < 0.001) expression (Supplementary Fig. 7m). In summary, ECM stiffness regulates epithelial cell fate in hAWOs predominantly via the Hippo, hypoxia, and Wnt signaling pathways, enabling stiffness-guided generation of region-specific hAWOs.

### Increased stiffness promotes hALO functional maturation and epithelial integrity

**Fig. 5:**
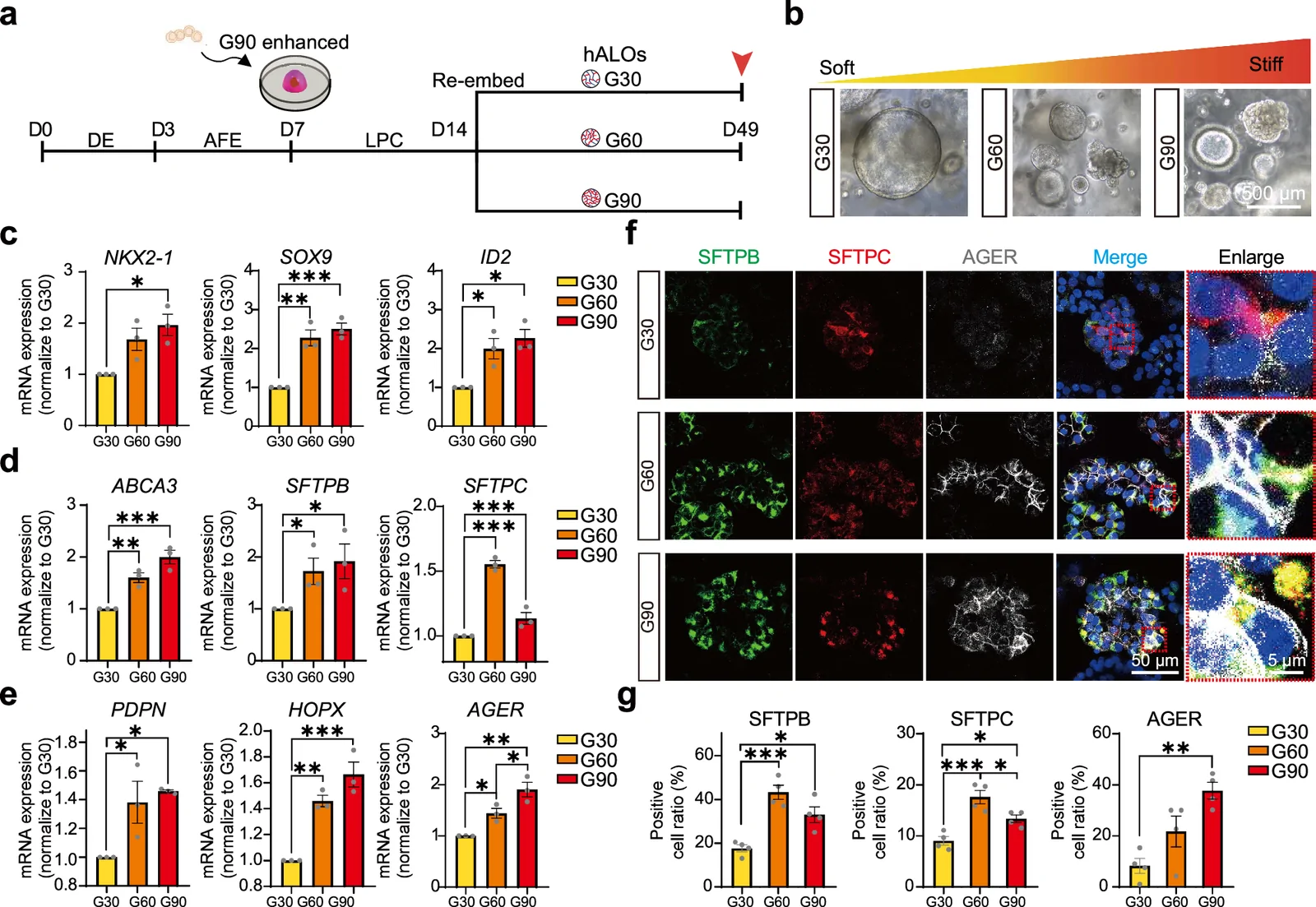
ECM stiffness promotes alveolar epithelial cells differentiation and maturation in hALOs. **a** Schematic illustrating stiffness-regulated differentiation of hALOs from LPCs, red arrowhead indicates samples collection time. **b** Representative bright-field images of hALOs differentiated in G30, G60 and G90 (*n* = 3). Organoids cultured in GelMA hydrogels displayed normal epithelial morphology. qPCR analysis of mRNA expression for **c** progenitor markers (*n* = 3) [*NKX2-1* (*p* = 0.0221), *SOX9* (*p* = 0.0021, <0.001) and *ID2* (*p* = 0.0102, 0.0292)], **d** AT2 markers (*n* = 3) [*ABCA3* (*p* = 0.0089, <0.001), *SFTPB* (*p* = 0.0258, 0.0459), and *SFTPC* (*P* < 0.001, <0.001)], and (**e**) AT1 markers (*n* = 3) [*PDPN* (*p* = 0.0201, 0.0429), *HOPX* (*P* < 0.001, <0.001), and *AGER* (*p* = 0.0016, 0.0374 0.0453)], with distinct dots biological representing individual values from three independent experiments. **f** Representative IF images of SFTPB (green), SFTPC (red), and AGER (white) in hALOs. Dashed red boxes highlight the magnified region from merged images. **g** Quantification of (**f**) reveals the percentage of positive cells expressing SFTPB (*p* = 0.0122, <0.001), SFTPC (*p* = 0.0339, 0.036, <0.001), and AGER (*p* = 0.0025) relative to total cell nuclei (*n* = 4 organoids from one biological replicate; data from two additional biological replicates are provided in the Source data file). Data are presented as mean ± SEM. *n* = 3 independent biological replicates. Statistical analyses were performed using a *t*-test (middle graph from **d**) or one-way ANOVA with Tukey’s multiple comparison test (**c**, left and right graph from **d**; **e**, **g**). *P < 0.05, **P < 0.01, ***P < 0.001.

The role of ECM stiffness in alveolar epithelial cell differentiation and maturation remains unclear. Herein, LPCs were encapsulated in GelMA hydrogels with different stiffnesses and differentiated into hALOs (Fig. 5a). By Day 35, the hALOs exhibited rounded and lumenized epithelial structures (Fig. 5b). The expression of the alveolar progenitor genes *NKX2-1*, *SOX9*, and *ID2* was upregulated in G90 (Fig. 5c). The expression of AT2 markers (*ABCA3*, *SFTPB*, and *SFTPC*) was increased in G60. With further increases in stiffness (G90), *ABCA3* and *SFTPB* expression levels were maintained, whereas *SFTPC* expression decreased (Fig. 5d). Early AT1 markers (*PDPN*, *HOPX)* were significantly upregulated in both G60 and G90, whereas the late-stage AT1 marker *AGER* was highest in G90 (Fig. 5e). Consistent with these findings, IF staining revealed greater numbers of SFTPB^+^ and SFTPC^+^ cells in both G60 (43.27% and 17.61%, respectively) and G90 (33.09% and 13.35%, respectively) than in G30 (17.67% and 9.04%, respectively). However, compared with G60 (21.75%) and G30 (8.27%), AGER^+^ cells were most abundant in G90 (37.74%) (Fig. 5f, g). These results indicate that increased stiffness promotes AT1 maturation, whereas intermediate stiffness supports AT2 cell specification.

**Fig. 6:**
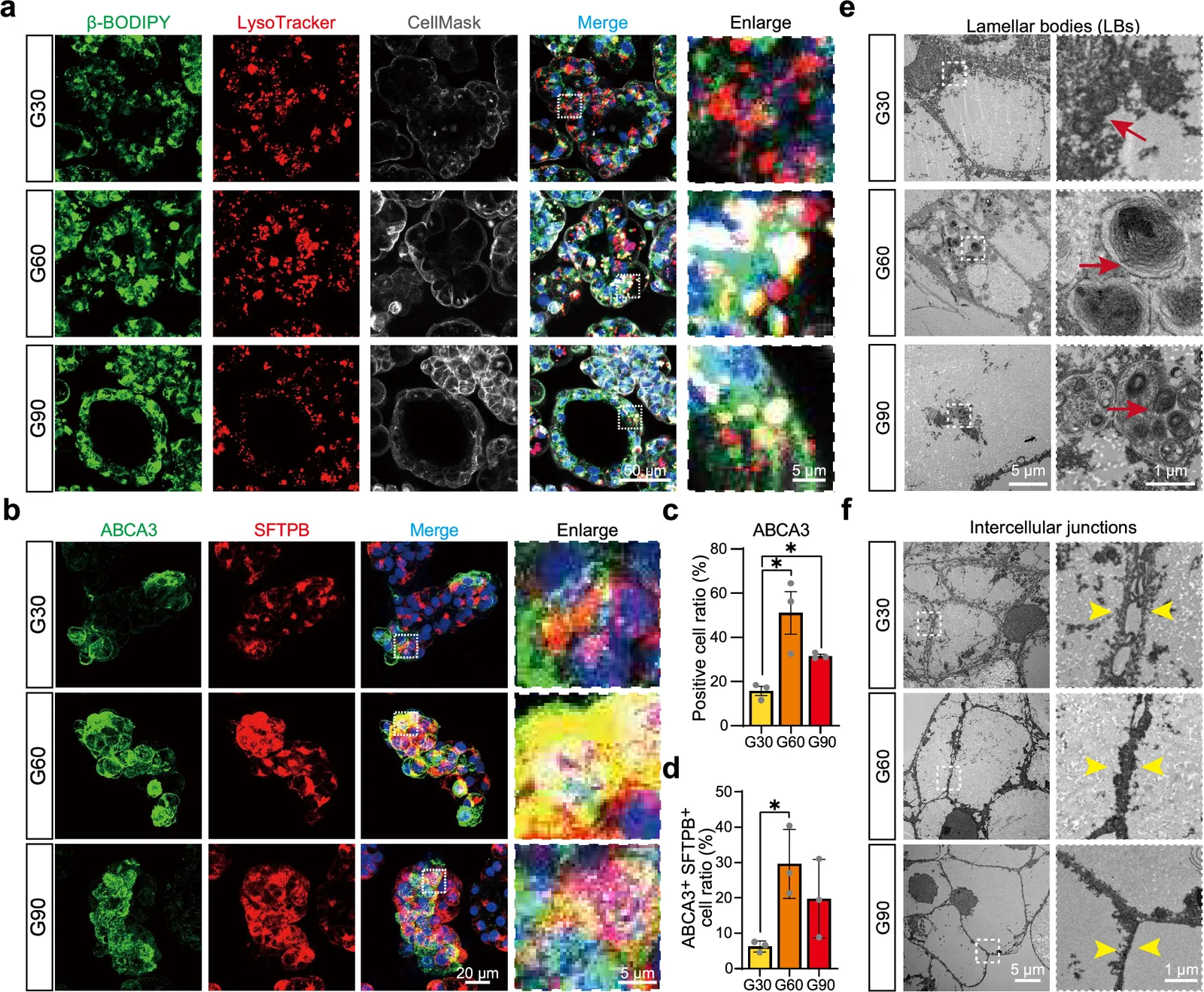
Functional maturation and epithelial integrity of hALOs under varying stiffness. **a** Representative live-cell confocal imaging of lipid metabolism and surfactant activity in hALOs cultured in G30, G60, and G90 hydrogels (*n* = 3). β-BODIPY (green) labels neutral lipids, LysoTracker (red) indicates acidic organelles, and CellMask (white) labels the cell membrane. Merged images and magnified insets highlight colocalization of lipid droplets and lysosomes, indicating active lipid metabolism in AT2 cells under intermediate stiffness conditions. **b** IF staining of SFTPB and ABCA3, magnified insets highlight colocalization of ABCA3^+^SFTPB^+^. **c**, **d** Quantification of ABCA3^+^ (*p* = 0.0108, 0.0306) and ABCA3^+^SFTPB^+^ cell ratios (*p* = 0.0361), respectively. **e** Representative TEM images demonstrating the presence of lamellar bodies (LBs) (red arrow) in AT2 cells, LBs with organized layers appear in G60 and G90 (*n* = 3). **f** Representative TEM images illustrating the intercellular junctions (yellow arrowhead) in AT1 cells, the intercellular space becomes smaller with increased stiffness (*n* = 3). Data are presented as mean ± SEM. *n* = 3 independent biological replicates. Statistical analyses were performed using *t*-test (**c**) or one-way ANOVA with Tukey’s multiple comparisons test (**c**, **d**). *P < 0.05.

Given that the key physiological function of the alveolus is surfactant production and respiration, we next performed established functional assays to evaluate AT2 surfactant maturation and AT1 epithelial integrity. Surfactant biogenesis in AT2 cells is tightly coupled to lipid handling and the endolysosomal-lamellar body (LB) axis, in which large amounts of phospholipids (e.g., DPPC) are synthesized, trafficked through acidic vesicular compartments, and packaged into LBs for secretion^47,48^. β-BODIPY (neutral lipid) and LysoTracker (acidic endolysosomal compartments) staining are powerful for studying lipid droplets and lysosomal activity. We found that AT2 cells in G60 exhibited increased colocalization of β-BODIPY and LysoTracker, indicating enhanced surfactant metabolism (Fig. 6a). Furthermore, we validated LB maturation using ABCA3, a transmembrane phospholipid transporter and a well-established marker of mature LBs. IF quantification demonstrated a higher proportion of ABCA3^+^ cells in G60 (51.04 ± 9.56%) and G90 (31.57 ± 0.76%) compared with G30 (15.75 ± 2.10%) (Fig. 6b, c). Moreover, ABCA3 and SFTPB co-staining revealed an increased fraction of ABCA3^+^SFTPB^+^ double-positive cells in G60, indicating more mature AT2 surfactant-associated function under intermediate-to-high stiffness (Fig. 6d).

TEM imaging was performed to visualize intracellular ultrastructural features of organelles. LBs store and secrete pulmonary surfactant, which is the key structure involved in AT2 maturation. TEM analysis revealed smaller and disorganized immature LBs in G30 but larger, highly organized mature LBs in G60 and G90 (Fig. 6e, red arrow). Microvilli (MVs) on AT2 cells primarily increased the surface area and maintained alveolar homeostasis, which was observed in all three stiffness groups (Supplementary Fig. 9a, green arrow). Notably, tubular myelin (TM), which is essential for surfactant storage and secretion in mature AT2 cells, was present in G60 and G90, which is a hallmark of mature AT2 cells (Supplementary Fig. 9b, purple arrow). Although ultrastructural features do not alone confirm functionality, the presence of mature LBs, MVs and TMs is considered a hallmark of functionally mature AT2 cells, because they are closely associated with the ability to synthesize, store, and secrete pulmonary surfactant^49^. The integrity of the alveolar epithelial barrier is primarily maintained by intercellular junctions (e.g., tight junctions) between alveolar epithelial cells^50^. AT1 in G90 displayed more elongated shapes and more tightly integrated intercellular junctions than those in G30 and G60 did (Fig. 6f, yellow arrow). The presence of elongated AT1-like morphology and well-formed intercellular junctions is morphologically consistent with functional barrier-forming alveolar epithelium.

Collectively, these results demonstrate that stiffness tuning enhanced surfactant biogenesis and LB maturation in AT2-enriched hALOs (G60) and strengthened AT1-associated epithelial integrity in AT1-dominant hALOs (G90), supporting the generation of more in vivo-like mature alveolar organoids under higher mechanical cues. If the goal is to obtain more alveolar stem cells for transplantation or seed cell banking, G60 is the ideal stiffness, whereas if the model aims to study AT1 integrity or AT2-to-AT1 transdifferentiation, G90 would be a better choice.

### ECM stiffness determines alveolar fates via the Hippo and Wnt

**Fig. 7:**
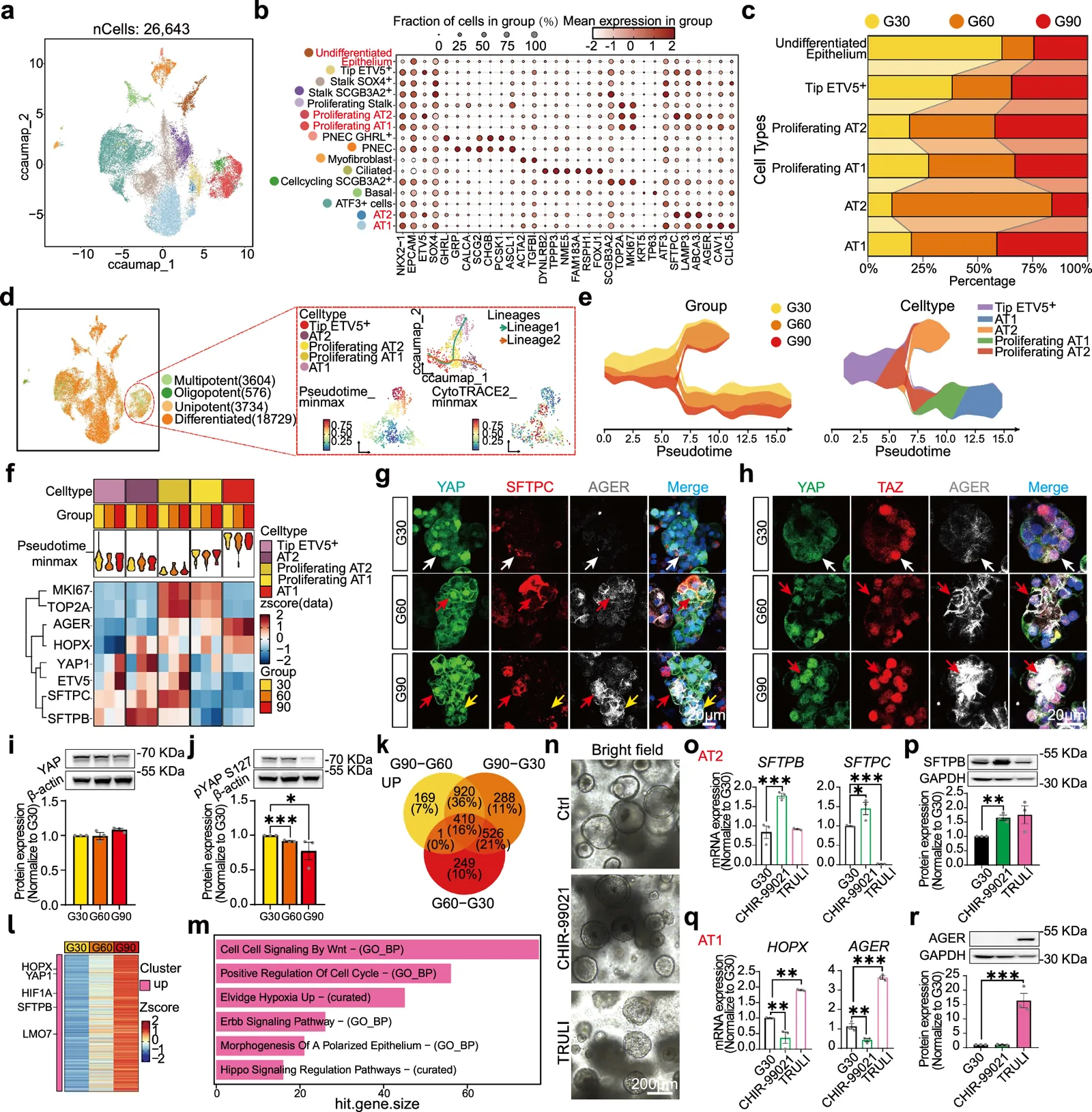
ECM stiffness regulates differentiation and maturation of hALOs via Hippo and Wnt signaling pathways. **a** UMAP of 26,643 hALOs cells differentiated under G30, G60 and G90 from three independent biological replicates. **b** Dot plot showing scaled marker gene expressions across 16 cell types, with dot size representing the fraction of expression cells and color representing mean gene expression. **c** Cell-types distribution under different stiffness conditions. **d** Differentiation potency and pseudotime analysis of alveolar lineage cells. **e** STREAM trajectories showing differentiation dynamics across stiffness conditions. **f** Heatmap showing dynamic gene expression patterns along differentiation trajectories. **g** Representative IF staining for YAP (green), SFTPC (red) and AGER (white) G30, G60, and G90, Scale bar: 20 μm (*n* = 3). White, red and yellow arrows indicate cytoplasmic, cytoplasm-to-nuclear and nuclear YAP, respectively. **h** Representative IF staining of YAP (green), TAZ (red), and AGER (white), Scale bar: 20 μm, (*n* = 3). White arrows indicate cytoplasmic YAP with nuclear TAZ; red arrows indicate nuclear YAP and nuclear TAZ. **i**, **j** Western blots and quantification of total YAP and pYAP S127 across all stiffness, *p* = 0.0496, <0.001. **k** Venn diagram showing upregulated differential expressed genes shared with G60 vs G30, G90 vs G60 and G90 vs G30. **l** Heatmap of stiffness-upregulated genes across low to high stiffness. **m** Pathway enrichment analysis of upregulated genes. **n** Bright-field images of hALOs treated with CHIR-99021 and TRULI. AT2 markers [SFTPB (*p* = 0.001) and SFTPC (*p* = 0.0228, <0.001)] measured by qPCR (**o**) and WB (**p**) (*p* = 0.002) after CHIR and TRULI treatment. AT1 markers [HOPX (*p* = 0.0015, 0.0083), AGER (*p* = 0.0059 and *P* < 0.0001)] measured by qPCR (**q**) and WB (**r**) (*p* = 0.0006) after CHIR and TRULI treatment. Data are mean ± SEM. *n* = 3 independent biological replicates. Statistical analyses were performed using *t*-test (**j**, **q**) or one-way ANOVA with Tukey’s multiple comparison test (**i**) or Dunnett’s multiple comparisons (**o**, **p**, **r**). *P < 0.01, ***P < 0.001.

In vitro experiments revealed that extracellular stiffness differentially regulates AT2 and AT1 cell fates, with intermediate stiffness favoring AT2 and higher stiffness promoting AT1 maturation (Figs. 5 and 6). To elucidate the underlying mechanisms, we performed scRNA-seq on hALOs generated at G30, G60, and G90. EpCAM^+^ cells were prospectively enriched prior to sequencing (Supplementary Fig. 10). Among the total 26,643 cells profiled from three independent biological replicates for each stiffness group (G30, G60 and G90), 99.3% epithelial and 0.7% mesenchymal lineages were further annotated into undifferentiated epithelium, tip ETV5^+^, AT2, AT1, and proliferating AT2 and AT1 cells (Fig. 7a, b; Supplementary Fig. 11a–e). AT2 cells were most abundant in G60, whereas AT1 cells were predominant in G90 (Fig. 7c), which is consistent with the experimental observations (Fig. 5). Differentiation potency and pseudotime (Slingshot) analysis resolved two trajectories from tip ETV5^+^ progenitors to mature AT1 and AT2 (Fig. 7d, e; Supplementary Fig. 12a, b). As shown in Fig. 7b, a proliferating AT1 cluster coexpressed mature AT1 (*CLIC5*, *CAV1*, and *AGER*) and AT2 (*ABCA3*, *LAMP3*, and *SFTPC*) markers, suggesting a transitional state between proliferating AT1 and AT2.

YAP is widely recognized for its critical role in alveolar epithelial cell differentiation and regeneration^51^. Therefore, we investigated its expression in different alveolar epithelial cells across stiffness groups. Unsupervised heatmaps of key markers are clustered into groups, e.g., *AGER* and *HOPX* for AT1, *MKI67* and *TOP2A* for proliferating cells, *SFTPB* and *SFTPC* for AT2, *ETV5* for tip ETV5^+^, and *YAP1* for AT1 fate determination (Fig. 7f). *YAP1* expression increased with stiffness in tip ETV5^+^, AT2, proliferating AT2 and AT1 cells. Although both YAP and TAZ are mechanosensitive Hippo effectors, their specific contributions to alveolar epithelial differentiation are still debated^18,52^. IF staining showed that YAP signaling translocated from the cytoplasm to the nucleus with increasing ECM stiffness (Fig. 7g, h). At lower stiffness (G30), the YAP signal was predominantly cytoplasmic and colocalized with SFTPC^+^ AT2 cells (Fig. 7g, white arrow). With the increasing stiffness, YAP gradually translocated into the nucleus and colocalized with AGER^+^ AT1 (Fig. 7g, red arrow) with or without SFTPC^+^ AT2 (Fig. 7g, yellow arrow). In contrast, TAZ exhibited strong nuclear localization in AGER^+^ AT1 cells or AGER^+^SFTPB^+^ double positive cells across all stiffnesses (Fig. 7h; Supplementary Fig. 13). At low stiffness (G30), the nuclei of AGER^+^ AT1 cells were TAZ^+^ but YAP^-^, indicating that nuclear TAZ, rather than YAP, directly specified AT1 fate (Fig. 7h, white arrow). At higher stiffness (G60 and G90), YAP and TAZ colocalized in AT1 nuclei (Fig. 7h, red arrow). Our results indicate that TAZ acts as a primary determinant of AT1 identity, whilst cytoplasmic-to-nuclear translocation of YAP contributes to AT2-to-AT1 transition under increased stiffness. WB revealed that total YAP protein levels remained relatively constant throughout the stiffness of the hydrogel, whereas phosphorylation at YAP Ser127 (pYAP S127), a readout of Hippo activity and cytoplasmic retention of YAP^53^, decreased as stiffness increased (Fig. 7i, j). These findings are consistent with the enhanced nuclear localization of YAP at G90 (Fig. 7h). Together, these findings suggest that ECM stiffness modulates Hippo-YAP signaling primarily through posttranslational control of pYAP S127 to regulate YAP subcellular localization in hALOs rather than through changes in total YAP abundance.

Among the differentially expressed genes (DEGs) identified, 410 genes were consistently upregulated, and 85 were downregulated with increasing stiffness (Fig. 7k; Supplementary Fig. 12c). Gradient-variant genes were enriched for pathways associated with alveolar differentiation, such as the Wnt, hypoxia, and Hippo signaling pathways, along with cell cycle regulation and ErbB signaling pathway (Fig. 7l, m). The downregulated DEGs were predominantly enriched in cellular stress and oxidative phosphorylation pathways, suggesting that the organoids were under stress conditions without sufficient ECM support (Supplementary Fig. 12d, e). Cell cycle and replication genes (*MKI67*, *TOP2A*) were comparable across G30, G60, and G90 (Supplementary Fig. 12f), indicating minimal effects of varying stiffness on cell proliferation status. Using the identified 25 significantly upregulated genes (*EGFLAM*, *CD163L1*, *TRIM43*, etc.) in idiopathic pulmonary fibrosis (IPF) patient-derived organoids^54^, we proved there were no canonical fibrotic programs in G90 (Supplementary Fig. 12g), supporting a non-pathological interpretation. Thus, G90 serves as a high-physiological boundary stiffness rather than a model of fibrosis, enabling us to interrogate stiffness-dependent epithelial fate decisions across a physiologically relevant gradient.

Functional validation with the CHIR-99021 treatment of hALOs in G30 indicated that the organoids maintained the lumen structure (Fig. 7n) and significantly upregulated AT2 expression (Fig. 7o, p). In contrast, the addition of TRULI induced the formation of aggregated clumps of cells lacking a visible lumen (Fig. 7n), a morphology previously associated with AT1^21^. Quantitative polymerase chain reaction (qPCR) and WB confirmed that TRULI predominantly promoted the expression of AGER (4-fold at the gene level and 15-fold at the protein level) (Fig. 7q, r). In summary, ECM stiffness promoted hALOs maturation through Hippo and Wnt signaling. Wnt upregulation enhances AT2 identity while restraining AT1 differentiation, whereas nuclear YAP activation promoted AT1 maturation at the expense of AT2 expression.

### SARS-CoV-2 variants recapitulate physiological infection tropism in hHLOs

**Fig. 8:**
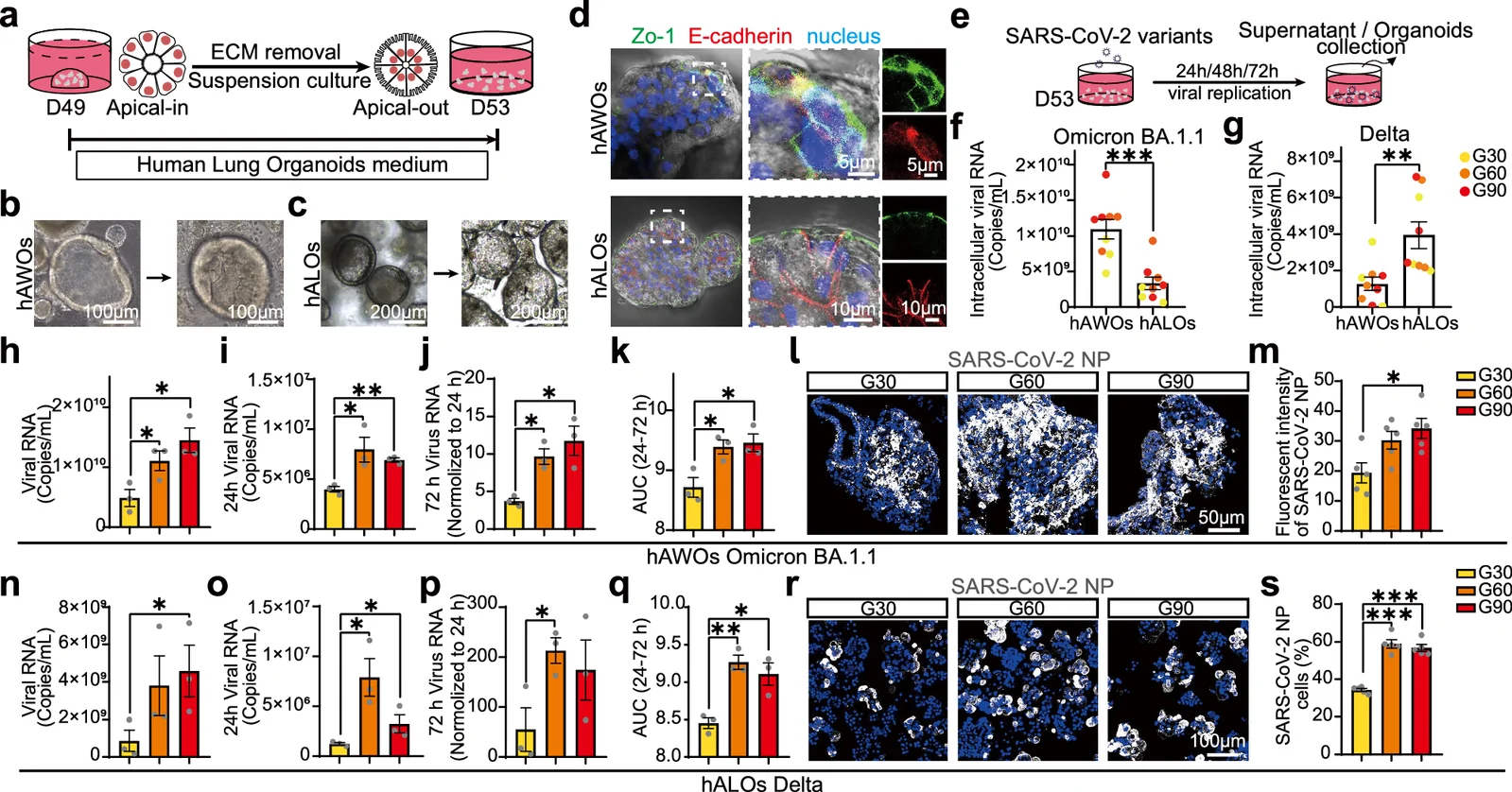
Omicron BA.1.1 and Delta display region-specific infection tropism in hAWOs and hALOs. **a** Schematic of apical-out organoid generation. Representative bright-field images showing the transition from apical-in to apical-out orientation in hAWOs (**b**) and hALOs (**c**) (*n* = 3). **d** Representative IF images of ZO-1 (green) and E-cadherin (red) (*n* = 3). **e** Schematic of SARS-CoV-2 infection. Intracellular viral RNA in hAWOs and hALOs infected with Omicron BA.1.1 (**f**, *P* < 0.001) or Delta (**g**, *p* = 0.0046). Yellow, orange, and red dots represent G30, G60, and G90, respectively (*n* = 3). qPCR analysis of Omicron BA.1.1 infection in hAWOs: **h** intracellular viral RNA at 72 hpi, *p* = 0.0178, 0.046; **i** viral RNA in supernatant at 24 hpi, *p* = 0.0012, 0.035; **j** fold change of viral RNA in supernatant from 24 to 72 hpi, *p* = 0.0107, 0.0394; and **k** area under the curve (AUC) analysis of viral titer between 24 and 72 hpi, *p* = 0.0246, 0.0368 (*n* = 3). **l**, **m** Representative IF staining and quantification of SARS-CoV-2 nucleocapsid protein (NP, white) in Omicron BA.1.1-infected hAWOs. (*n* = 5 organoids from one biological replicate; data from two additional biological replicates are provided in the Source data file, *p* = 0.0168). qPCR analysis of Delta infection in hALOs: **n** intracellular viral RNA at 72 hpi, *p* = 0.0333; **o** viral RNA in supernatant at 24 hpi, *p* = 0.0191, 0.0482; **p** fold change of viral RNA in supernatant from 24 to 72 hpi, *p* = 0.035; and **q** AUC analysis of viral titer between 24 and 72 hpi, *p* = 0.0045, 0.0125 (*n* = 3). **r**, **s** Representative IF staining and quantification of NP^+^ cells in Delta-infected hALOs. (*n* = 5 organoids from one biological replicate; data from two additional biological replicates are provided in the Source data file; *p* < 0.001, <0.001). Data are presented as mean ± SEM. Statistical significance was determined using *t*-test (**f**–**i**, **n**, **o**, **p**), one-way with Tukey’s multiple comparisons (**h**–**k**, **m**, **o**, **q**, **s**). *P < 0.05, **P < 0.01, ***P < 0.001.

Previous studies have used SARS-CoV-2 variants to validate the physiological relevance of adult stem cell (ASC)-derived hAWOs and hALOs^55^. Omicron BA.1.1 and delta SARS-CoV-2 variants exhibit distinct infection tropisms in the human respiratory system, with Omicron preferentially infecting the proximal airway epithelium and Delta showing stronger tropism for the alveolus^56^. Accordingly, to establish physiological relevance, we challenged stiffness-guided, region-specific lung organoids with Delta and Omicron to test whether our organoids recapitulate region-specific epithelial properties. To facilitate viral apical entry, the organoids were released from the GelMA hydrogels to create an apical-out configuration (Fig. 8a). The epithelial layer of the organoids was inverted to an apical-out structure (Fig. 8b, c), with ZO-1 located primarily on the exterior and E-cadherin localized internally (Fig. 8d). HAWOs and hALOs were then infected with the SARS-CoV-2 variants and samples were collected at 24, 48 and 72 h post infection (hpi) (Fig. 8e). At 72 hpi, Omicron BA.1.1 resulted in a higher infection titer in hAWOs than in hALOs (Fig. 8f), whereas Delta infection occurred more efficiently in hALOs than in hAWOs (Fig. 8g). These results align with those of previous studies^57^ showing the strong tropism of Delta for alveolar tissue and the enhanced replication of Omicron in airway epithelial cells.

HAWOs differentiated in high-, intermediate- and low-stiffness hydrogel models proximal to the distal airway. Omicron BA.1.1 infection in hAWOs differentiated under G90 resulted in significantly higher viral loads and infection efficiency than in those under soft G30 (Fig. 8h–k), which aligns with the tropism of Omicron for proximal airways. IF staining and quantification further confirmed that the Omicron infection ratio was significantly greater in G90 (Fig. 8l, m). With respect to hALOs, Delta infection was more efficient in high-stiffness organoids, corresponding to enhanced AT2 and AT1 cell maturation (Fig. 8n–q). IF staining and quantification further confirmed that the Delta infection ratio was significantly greater in the G60 and G90 groups (Fig. 8r, s). In conclusion, region-specific lung organoids generated in hydrogels with tunable stiffness faithfully recapitulate SARS-CoV-2 infection tropism in vivo. Omicron BA.1.1 preferentially infects proximal airway organoids, whereas Delta targets alveolar organoids. These findings provide a valuable region-specific respiratory epithelium in vitro platform for studying SARS-CoV-2 pathogenesis and variant-specific tropisms.

## Discussion

In this study, a stiffness-tunable GelMA hydrogel model was employed to provide a 3D culture environment for hPSC-derived respiratory organoids, enabling the investigation of how ECM stiffness affects region-specific lung development. The results revealed that increased ECM stiffness enhanced LPC differentiation, proximal airway epithelial cell generation, alveolar maturation, and AT2/AT1 cell transition primarily through the interaction among the Hippo, hypoxia and TGF-β pathways. However, lower ECM stiffness favored the differentiation of distal bronchial epithelial cells (e.g., SCGB1A1^+^ and SCGB3A2^+^ secretory cells) by upregulating the Wnt pathway (Supplementary Fig. 14). Extracellular stiffness gradients can influence region-specific lung epithelial cell fate by modulating the Hippo, hypoxia, Wnt, and TGF-β pathways. These organoids recapitulate SARS-CoV-2 variants’ region-specific infection tropism, with Omicron BA.1.1 targeting the proximal airway and Delta targeting the alveolar compartment. Thus, we generated region-specific lung organoids by modulating 3D hydrogel stiffness. This study provides an in vitro stiffness-tunable ECM model to elucidate the mechanism of ECM stiffness in determining lung epithelial cell fate, facilitating the generation of region-specific lung organoids that recapitulate key aspects of in vivo lung epithelial differentiation, and providing a platform to study extracellular stiffness in region-specific lung generation and disease modeling.

The ECM is not an inert and universal scaffold; rather, it provides both biochemical (e.g., ECM components, soluble factors, and ligand‒receptor interactions) and biophysical (stiffness, topography, and ECM stress) cues with region-specific compositions and mechanics that guide tissues’ morphogenesis and function^58^. Although mimetic ECM stiffness along the proximal-to-distal axis biases hAWOs toward distinct proximal-to-small-airway identities, modulation of ECM stiffness alone is not sufficient to fully replicate an in vivo airway segment. Along the respiratory tree, the proximal airways are enriched for goblet and ciliated cells, whereas distal regions contain SCGB1A1^+^ and SCGB3A2^+^ secretory cells and alveolar epithelia^59^, which are embedded in regionally heterogeneous ECM components^60^ with dynamic mechanical properties^61^. Decellularized ECM (dECM) scaffolds and hydrogels provide biomimetic 3D culture environments that preserve region-specific instructive cues^62^. hPSC-derived lung epithelial cells exposed to alveolar region-enriched dECM adopt distal or alveolar phenotypes, while the nonregional lung dECM exhibits enhanced lung progenitor formation and maturation^63^. In contrast, trachea-derived dECM scaffolds or hydrogels support the proliferation of bronchial epithelial cells and the formation of a confluent epithelial layer^64^. Together, these observations support the notion that region-matched dECM directs epithelial fate in vitro. Hyaluronic acid (HA) is a major constituent of the lung interstitium and alveolar space. The incorporation of HA into polyethylene glycol (PEG)-Matrigel systems significantly increased viscoelasticity and triggered mechanotransduction, which promoted alveolar progenitor cell differentiation^33^. These studies verify that the ECM composition influences epithelial fate^60^.

In the current study, we focused specifically on how extracellular stiffness influences lung epithelial cell fate. To minimize confounding effects from region-specific ECM components, we used gelatin-based GelMA hydrogels without the introduction of exogenous biochemical signals (Supplementary Fig. 1). This design allowed us to determine the effect of stiffness on respiratory epithelial patterning. Physical forces generated by ECM are sensed by cells and transduced into intracellular signals through several primary pathways, including Hippo, mechanotransduction cascades, and FAK signaling pathways^22^. These pathways ultimately converge to influence gene expression and determine cell fate decisions^65^. Our study indicates that ECM stiffness modulates lung development by regulating the Hippo pathway, which in turn regulates the specification of lung progenitors and the differentiation of airway and alveolar epithelial cell fates (Figs. 1, 4 and 7). Mechanistically, stiffness-tunable GelMA hydrogels direct lung epithelial fates predominantly through mechanical signaling (e.g., Hippo) rather than cell-ECM adhesion-mediated FAK signaling. Future work incorporating defined, region-specific biochemical gradients in a stiffness-tunable hydrogel system will help clarify how mechanical and biochemical signals coordinate lung epithelial development. In addition to stiffness and composition, other microenvironmental properties, such as ECM remodeling, matrix topography and viscoelasticity, critically regulate lung epithelial fate and regeneration by altering stress relaxation, mechanotransduction, matrix topography and niche plasticity^66,67^. Integrating tunable viscoelasticity and controlled degradability into hydrogels, together with 3D-printing and micropatterning, would more comprehensively recapitulate the native lung microenvironment.

To recapitulate the proximal-to-distal mechanical gradient of the respiratory system, we employed three GelMA hydrogels (G30, G60 and G90) with stiffness ranging from 2.99 to 11.31 kPa (Supplementary Fig. 1c), covering reported lung regional ECM values (0.5–15 kPa) and fetal lung stiffness (3.68–5 kPa)^27,68^. In hAWOs, goblet, basal and ciliated cells preferentially developed in high stiffness hydrogels, whereas club and SCGB3A2^+^ secretory cells were enriched in lower stiffness hydrogels (Fig. 2). These high-to-low stiffness-derived hAWOs resemble in vivo lung conducting airway epithelium from trachea to distal bronchioles. Surprisingly, the distal alveolar cell did not favor soft hydrogel as we proposed. Instead, intermediate-to-high stiffness (6.22–11.31 kPa) enhanced alveolar cells maturation and AT2-to-AT1 transition (Fig. 5). A previous study reported that cortical organoids developed better in relatively stiffer scaffolds (7–9 kPa) than in softer scaffolds (0.1–3 kPa). The physical constraints imposed by the developing skull with orders of magnitude higher stiffness (1–50 MPa) may provide tissue-level constraints beyond the local ECM^69^. Similarly, the lung parenchyma is surrounded by the visceral pleura, whose tensile mechanics are orders of magnitude higher than those of the local epithelial ECM^41^. Therefore, lung epithelial development may occur under multiscale mechanical influences, including tissue-level constraints beyond the local basement membrane and interstitial ECM. Luque et al.^70^ used AFM to measure the local stiffness of lung ECM in different regions, including alveolar wall segments, alveolar wall junctions, and pleura regions, with the stiffness increasing from 5.59 to 15.76 kPa. Our results indicated that alveolar epithelial cells differentiated better under intermediate-to-high stiffness, which falls within the stiffness range reported for alveolar regions in vivo. Taken together, airway development (conducting function) strictly follows a proximal-to-distal high-to-low stiffness gradient. However, the development and maturation of the alveolar epithelium, which requires external mechanical force, seems to play an essential role in signaling pathways to specify epithelial fates.

Both hPSC- and lung tissue-specific ASC-derived respiratory organoids recapitulate key structural and functional properties of the respiratory epithelium^55^. Their cellular compositions are broadly similar, comprising four major airway epithelial cells (ciliated, goblet, club and basal cells) and two principal alveolar epithelial cell types (AT2 and AT1)^71^. ASC-derived respiratory organoids yield more mature and homogeneous epithelial cells with minimal off-target differentiation but are limited by tissue access. In contrast, hPSC-derived respiratory organoids provide an essentially unlimited cell source but are often immature with high heterogeneity and long differentiation timelines. ECM stiffness is a central pathological feature of IPF and chronic obstructive pulmonary disease (COPD), in which matrix remodeling alters tissue mechanics and actively drives epithelial cell behaviors^72^. In future studies, combining patient-derived ASC organoids with stiffness-tunable hydrogels will enable personalized in vitro models to explore how extracellular mechanics contribute to epithelial plasticity, injury responses and fibrosis progression in diseases such as IPF and COPD.

This study explored the effect of ECM stiffness on the generation of region-specific lung organoids and the underlying orchestrated pathways, providing evidence that ECM is capable of determining lung epithelial cell differentiation fates and generating distinct region-specific organoids from proximal to distal regions. This in vitro model recapitulates in vivo lung ECM stiffness and epithelial formation and therefore holds great potential for studying region-specific lung development and ECM-induced lung diseases.

## Methods

For Supplementary Figs. 1–14, Supplementary Tables 1–3 (including the primers and gene lists used for scRNA-seq annotation), and Supplementary Methods, are provided in the Supplementary Information. Antibodies are provided in the Supplementary Data 1, and upregulated gene lists used for bulk RNA-seq annotation are provided in the Source data.

### Ethics statements

All work involving human embryonic stem cell line H1 (WiCell Research Institute) was reviewed and approved by the Institutional Review Board of Guangzhou National Laboratory (2026-0019). All work involving live SARS-CoV-2 was performed at the Biosafety Level 3 Laboratories of Guangzhou Customs District Technology Center (Bioland, Guangzhou, China) and approved by the ethics committee under protocol number IQTC2024008.

### GelMA hydrogel preparation and characteristics

GelMA hydrogels with varying stiffness levels were prepared by altering the methacrylation substitution degree while maintaining a constant precursor concentration (8% w/v) and crosslinking time (30 s). GelMA kits with substitution degrees of 30%, 60%, and 90% were obtained from Engineering for Life Co. (EFL, Cat#EFL-GM-30, Cat#EFL-GM-60, Cat#EFL-GM-90). Hydrogel was prepared following the manufacturer’s protocol. Protocols for hydrogel preparation, rheology, compression testing, swelling ratio analysis, scanning electron microscopy, and degradation testing are detailed and depicted in the Supporting information.

### hPSCs differentiation into LPCs and HLOs

#### Maintenance of hPSCs

The hPSCs line H1 was maintained on Matrigel (Biocoat, Cat#354277) coated 6-well culture plates in mTeSR1 medium (StemCell Technologies, Cat#85850). Cells were passaged every 4 to 6 days using 0.5 mM EDTA (Invitrogen, Cat#AM9261) at split ratios of 1:20 to 1:40. For differentiation experiments, hESCs were dissociated into single cells with Accutase (StemCell Technologies, Cat#07922) and replated at a density of 1.2 × 10^5^ cells/well in 24-well culture plates.

#### Differentiation of hPSCs into AFE spheroids

Differentiation into AFE spheroids was carried out following previously published protocols with slight modifications. Briefly, H1 cells at ~80% confluence were treated with DE medium for 3 days, followed by incubation in AFE medium. Spheroids were formed and floated in the culture after 3–5 days with treatment of AFE medium.

#### Encapsulation of AFE spheroids in GelMA hydrogel and differentiation into LPCs

AFE spheroids were encapsulated into GelMA hydrogel to support the 3D culture using a previously established protocol with modifications described below^25^. GelMA was purchased from Engineering for Life Co., and the hydrogel precursor was prepared following the user manual, with details provided in the Supplementary Materials. Approximately 200 AFE spheroids were mixed with 25 µL GelMA precursor and placed at the center of one well of a 24-well plate. The 24-well plate was gently inverted to avoid the precipitation of spheroids and immediately crosslinked with 405 nm ultraviolet light (25 mW/cm^2^, Curing surface light, EFL, Cat#EFL-LS1602) for 30 s. Solidified GelMA domes were overlaid with LPCs basal medium to replace the uncrosslinked residual polymer, photoinitiator, and PBS. After 10 min, the medium was refreshed and replaced every other day for 7 days.

#### Differentiation of LPCs into human lung organoids (hAWOs or hALOs)

LPCs were released from GelMA hydrogels using 100 μg/mL Collagenase Type II (Sigma-Aldrich, Cat#C2-28-100 mg) and re-encapsulated (200 organoids) following the same GelMA encapsulation protocol. LPCs were incubated in hAWOs or hALOs medium to obtain hAWOs and hALOs individually. The compositions of hAWOs and hALOs media were provided in the Supporting Information.

For all experiments, cells or organoids were randomly allocated into the corresponding experimental groups. All differentiation procedures, culture conditions and downstream analyses were performed in parallel using the same protocols to minimize potential confounding variables.

### RNA extraction and qPCR analysis

The total RNA extraction of LPCs and HLOs, cDNA synthesis, and qPCR reactions were performed according to the manufacturer’s protocol (Qiagen, Cat#74106; Thermo Fisher Scientific, Cat#EP0753). The *GAPDH* gene served as the housekeeping gene for normalization. Primer sequences are provided in Supplementary Table 1. Data analysis was conducted using Graphpad Prism Software 8.0.1, and normalization was performed against G30 using ΔΔCt or relative to GAPDH using ΔCt^11^.

### Immunofluorescence staining

IF staining was carried out following the published protocols. Briefly, hAWOs were released from GelMA and carried out following cryosection methods. Whilst the LPCs and hALOs with a smaller size were released from GelMA and performed whole-mounting staining. Antibodies information and dilutions were listed in Supplementary Data 1.

### Flow cytometry and fluorescence-activated cell sorting (FACS)

Flow cytometry was conducted using an Agilent NovoCyte Advanteon (Agilent Technologies), and FACS was performed with a Sony MA900 sorter (Sony, Cat#LE-MA900FP). LPCs or HLOs were enzymatically dissociated into single cell suspensions using TrypLE (Thermo Fisher, Cat#12605028) and stained with antibodies specific for flow cytometry or FACS. Details of antibodies are provided in Supplementary Data 1.

### Western Blot (WB)

Total protein was extracted from LPCs and HLOs using RIPA lysis buffer (Beyotime, Cat#P0013B) supplemented with a protease inhibitor cocktail (Beyotime, Cat#P1006). Protein concentrations were quantified using a BCA Protein Assay Kit (Beyotime, Cat#P0010). Equal amounts of total protein (30 μg) were loaded onto 4–20% precast polyacrylamide gels (GenScript, Cat#M42015C) for separation. Antibodies and their respective dilutions are listed in Supplementary Data 1. Protein molecular weight markers (GenScript, Cat#M00624-250; Invitrogen, Cat#26619) were added to determine the size of the protein bands. The intensity of WB bands was quantified using densitometry analysis. After acquiring the blot images, the grayscale values of the bands were measured using ImageJ software.

### Measurement of the ciliary beat frequency, ciliary beat amplitude and mucociliary transport

To analyze CBF, hAWOs (>30 days in hAWOs medium) were released from GelMA hydrogels (collagenase II as described in the “Differentiation of LPCs into human lung organoids (hAWOs or hALOs)” section) and transferred to glass-bottom confocal dishes. For measuring the CBF and CBA, 1366 frames of bright-field images of the beating cilia were acquired at 130 frames per second (fps) on a high-speed confocal imaging system (Andor Dragonfly 200). CBF was calculated using a fast Fourier transform (FFT) based analysis^42^. Briefly, the regions of interest (ROIs) (2 × 2 μm) of each frame were selected using the ImageJ software program (National Institutes of Health). Then, the average brightness of each ROI and frame was calculated, followed by exporting and processing in R (v4.5.2) for FFT analysis. The ROIs of beating cilia by thresholding the CBF (3 and 35 Hz) were selected to minimize the effect of noise. At least three fields of view (FOVs) were recorded from each batch.

Ciliary length was measured using the freehand line tool in ImageJ by tracing individual cilia with clearly identifiable base and tip points^43^. For each batch, at least three randomly selected FOVs were analyzed, and at least 10 individual cilia per FOV were measured. The average value was used for statistical analysis.

For the measurement of CBA, individual cilia with clearly visible basal bodies and tips were first identified using ImageJ. Kymographs were then generated to visualize the spatiotemporal movement of the cilia. The displacement range of each beating cilium was manually traced on the kymographs, and the CBA was defined as the maximum excursion distance between the base and tip positions during the beating cycle.

To assess mucociliary transport, fluorescent microspheres (Fluoresbrite®, 1 μm; Polysciences; 1:1000 dilution) were added to the culture medium. Fluorescence images were acquired using a high-speed spinning-disk confocal imaging system (Andor Dragonfly 200) equipped with a 20× oil-immersion objective. Movies were recorded at 20 frames per second (fps), with 400 frames captured per acquisition. The trajectories and displacement of fluorescent beads driven by ciliary beating were automatically tracked using TrackMate in ImageJ (Fiji). Particle transport velocity was calculated based on the displacement of individual bead trajectories over time. Because ciliated regions in airway organoids are spatially localized rather than uniformly distributed across the entire FOV, particle trajectories were analyzed specifically within regions exhibiting coordinated ciliary beating. This approach ensured that the measured particle movement reflected active mucociliary transport rather than passive diffusion. Particles with extremely low velocities were considered to represent random Brownian diffusion and were excluded from the analysis. Brownian motion was used as a negative control for mucociliary transport analysis following previously described approaches^43^. For each experiment, more than five particle trajectories per organoid were analyzed to calculate the mucociliary transport index. During imaging, the samples were maintained in an incubator (37 °C, 5% CO₂) equipped with the microscope.

### Forskolin-induced swelling of organoids

One day prior to the experiment, organoids were released from GelMA hydrogels as described above, resuspended in 20 μL of growth factor-reduced Matrigel, and seeded into 48-well plates. After Matrigel solidification, 100 μL of AWOs culture medium (without 8Br-cAMP and IBMX) was added. For the swelling analysis, the organoids were incubated with 10 μM forskolin (Aladdin, Cat#66575-29-9) and 2 μM Calcein AM (Solarbio, Cat#CA1630) for 0–24 h at 37 °C, 5% CO₂. Images were acquired at 2 h, 6 h, 12 h, and 24 h using a bright-field microscope (Nikon, ECLIPSE Ts2-FL). The area of organoids was analyzed using ImageJ software, and normalized relative to the average area at 0 h (before treatment). Images were again analyzed at 2 and 24 h post-forskolin addition, and the ratio of the post-swelling area to the original area was calculated. At least 5 individual organoids were measured for each sample with 3 replicates.

### Transmission electron microscopy (TEM)

TEM was used to analyze the intracellular morphological ultrastructures of organelles in hAWOs and hALOs (e.g., lamellar bodies, microvilli, intercellular junctions, 9 + 2 structure) associated with functional maturation. HLOs were collected and processed for TEM analysis following previously described methods. Ultra-thin sections (approximately 90 nm) were cut on an Ultra-microtome (Leica EM UC7), mounted on grids and stained with uranyl acetate and lead citrate. Images were capture during a digital camera integrated with a TEM (Thermo Fisher Scientific, Talos L120C G2).

### Bulk RNA-seq

LPCs from G30, G60 and G90 groups (three biological replicates per group) were lysed with TRIzol^TM^ reagent (Thermo Fisher, Cat#15596018) and flash frozen in liquid nitrogen for bulk RNA-seq. Quality testing, database construction and RNA-seq were subsequently performed by Annoroad Gene Technology (Beijing, China). A gene expression matrix of 60,625 genes was generated. The initial exploratory analysis included heatmaps and bubble plots, which were performed using the SangerBox online platform [http://www.sangerbox.com/tool]^73^. Differential expression analysis identified significantly upregulated genes with FDR < 0.05 and |log_2_(FC)| > 1. Bubble plots highlighted key pathways enriched in Kyoto Encyclopedia of Genes and Genomes (KEGG) and other pathways of interest.

### scRNA-seq and analysis

For scRNA-seq, three biological replicates were included for each stiffness sample (G30, G60, G90). Pre-sequencing qPCR and IF were used to verify marker-expression trends. Single-cell suspensions were prepared from HLOs differentiated under varying stiffness. Flow cytometry was used to sort EpCAM^+^ cells for further processing. Quality control, RNA sequencing, and library construction for hAWOs were performed using the C4 platform provided by Geneplus Gene Technology. The raw sequencing reads for hAWOs (three biological replicates for each stiffness) were aligned to the GRCh38 genome, and a total of 55,480 genes were detected in 71,179 cells. Stressed cells from organoids were filtered using Gruffi (v1.5.5). Downstream analyses were performed using Seurat (v5.1.0). A total of 10 unique cell types were annotated for hAWOs. The gene markers for each cell type were provided in Supplementary Table 2. For hALOs, these processes were carried out using the 10x Genomics platform provided by Annoroad Gene Technology. Similarly, raw reads for hALOs (three biological replicates for each stiffness) were mapped to the GRCh38 genome as well, resulting in 67,481 genes from 26,643 cells, and 16 unique cell types were identified for hALOs. The gene markers used for annotation hALOs were provided in Supplementary Table 3.

Initial exploratory analyses included Venn diagrams, heatmaps, and pathway enrichment visualizations based on Gene Ontology-Biological Process (GO-BP) and curated databases. CytoTRACE2 (v1.0.0) was employed to predict cell potency categories and absolute developmental potential from organoids’ scRNA-seq data. For alveolar trajectory inference, Slingshot (v2.12.0), STREAM (v1.1) and Partition-based Graph Abstraction (PAGA) were simultaneously applied to verify consistent trajectories based on the gene expression matrix. All statistical analyses for violin and box plots were conducted using the “wilcox.test (alternative = ‘two-sided’)” function in the R package “stats.”

### Validate transcriptomic pathways with small molecules

#### LPCs

To evaluate the role of the TGF-β and Hippo signaling pathways in NKX2-1^+^ LPC generation, LPCs were treated on the first day of the LPCs differentiation stage with 10 µg/mL TGFβ1 (Procell, Cat#PCK091) or 10 µM TRULI (MCE, Cat#HY-138489), while LPCs cultured in standard LPC medium served as the control. Samples were collected after 7 days for further analysis.

#### HLOs

To examine the regulatory effects of hypoxia, Hippo, and Wnt signaling pathways on the directional differentiation and maturation of HLOs, day 49 organoids were treated with one of the following: 50 µM FG-4592 (MCE, Cat#HY-13426); 3 µM CHIR-99021 (MCE, Cat#HY-10182); 10 µM TRULI; or combinations of these conditions. G30 lung organoids cultured in standard differentiation medium served as the control group. Treated organoids were collected after 7–14 days for subsequent analysis.

### SARS-CoV-2 infection

SARS-CoV-2 (Omicron BA.1.1 and Delta variants) was isolated from COVID-19 patients in Guangdong, China, by the Guangdong Provincial Center for Disease Control and Prevention. All SARS-CoV-2-related experiments were conducted in Biosafety Level 3 (BSL-3) laboratories at the Guangzhou Customs District Technology Center (Guangzhou International Bio Island, Guangzhou, China). To generate apical-out organoids for viral infection assays, D49 apical-in organoids were transferred to suspension culture following ECM removal and maintained for 3 days to reverse epithelial polarity^57^. SARS-CoV-2 infection was added in HLOs medium at a multiplicity of infection (MOI) = 1 for 2 h at 37 °C. Then, the organoids were washed three times with DPBS. Fresh HLO culture medium was added and incubated at 37 °C for 72 h. Supernatant was collected at specified time points (24, 48, 72 hpi) for viral titer determination. After 72 h, the organoids were collected for qPCR and IF staining.

### Viral RNA isolation and quantification of viral titers

Viral RNA was isolated from both the organoids and supernatant of infected organoids following previously published methods with slight modifications^74^. The pcDNA3.1 plasmid containing *SARS-CoV-2 N* gene fragments (4049 bp) was synthesized and provided by Dr. Jincun Zhao’s Laboratory (Guangzhou National Laboratory, Guangdong, China) and served as a positive control for viral quantification. Purified plasmid DNA was quantified, and DNA copy numbers were calculated based on the virus genome equivalents formula^75^. A stock solution of 10^10^ copies/μL was prepared, and 10-fold serial dilutions were performed to obtain 10^−2^ to 10^−6^ copies/μL working solutions. Standard qPCR reactions were performed using primers specific to SARS-CoV-2 (listed in Supplementary Table 1). ΔCt values obtained from the qPCR of serial dilutions were plotted to construct the standard curve. The viral titer in each sample was then calculated by inputting its ΔCt value into the equation derived from the standard curve. Area under the curve (AUC) was calculated from the viral titer from different time points indicated on the *y*-axis.

### Image analysis

A bright field microscope (Nikon, ECLIPSE Ts2-FL) was used to capture the bright field of the organoids. The size of the organoids was measured by ImageJ. Confocal imaging was conducted with a Carl Zeiss LSM 980 inverted confocal laser scanning microscope, and Zen 2.6 software was used to generate 3D Z-stack images. Image processing and analysis were performed using ImageJ software. The cellular fluorescence intensity was calculated with the following formula: Fluorescence intensity = mean fluorescence of selected cell ÷ mean fluorescence of background reading. For each sample, 9 representative FOVs (top-left, top-center, top-right; center-left, center, center-right; bottom-left, bottom-center, bottom-right) were captured using a Carl Zeiss LSM 980 confocal microscope. Images to be analyzed were selected randomly from non-overlapping regions to avoid sampling bias. Approximately 500 cells or 4–5 randomly selected images were analyzed per sample. The percentage of positive cells was calculated as the number of positive cells divided by the number of nuclei. Image analysis and quantification of intracellular mucin content in the airway, based on published literature^76^. The area and length of the stain were applied to the volume density to translate into a 3D estimate of mucin volume per square millimeter of airway epithelium.

### Statistics and reproducibility

At least three droplets for each group were observed. All representative experiments were based on three independent biological experiments. All additional images are provided in the Source data. The data are presented as the means ± standard error of mean (SEM). All comparisons were statistically analyzed by *t*-test, one-way ANOVA or two-way ANOVA with Tukey’s or Dunnett’s multiple comparison test. All of the statistical analyses in this study were performed with GraphPad Prism 8.0.2 software. A *p* value less than 0.05 is considered to indicate statistical significance.

### Reporting summary

Further information on research design is available in the Nature Portfolio Reporting Summary linked to this article.

## Supplementary information

Supplementary Information

Description of Additional Supplementary Files

Supplementary Data 1

Supplementary Movie 1

Supplementary Movie 2

Supplementary Movie 3

Supplementary Movie 4

Supplementary Movie 5

Supplementary Movie 6

Reporting Summary

Transparent Peer Review file

## Source data

Source data

## Data Availability

The scRNA-seq datasets generated in this study have been deposited in the NCBI Gene Expression Omnibus (GEO) database under accession codes GSE293170 (hAWOs) and GSE292932 (hALOs). Processed bulk RNA-seq results generated in this study are provided in the Source data file. Source data are provided with this paper.

## Code availability

All code used to generate the data in this manuscript is adapted from base R, Seurat, or other publicly available packages. The code is publicly available and has been deposited in GitHub at [https://github.com/EddieLv/1-Extracellular-stiffness-regulates-site-specific-lung-development]. The specific version of the code associated with this publication is archived in Zenodo and is accessible via [10.5281/zenodo.20507306]^77^.
