# Supplementary material for: Extracellular matrix stiffness directs region-specific lung epithelial differentiation revealed by hPSC-derived lung organoids
Source: Nat Commun. 2026 Jul 17;17:8757. doi: 10.1038/s41467-026-75663-9 (PMC13493954; doi:10.1038/s41467-026-75663-9)
Supplement: Supplementary file 1 [file 41467_2026_75663_MOESM10_ESM.pdf]

Corresponding author(s): Huisheng Liu, Hao Meng, Tao Xu, Juncun Zhao and Xuepeng Chen

Last updated by author(s): Jun 10, 2026

Reporting Summary

Nature Portfolio wishes to improve the reproducibility of the work that we publish. This form provides structure for consistency and transparency in reporting. For further information on Nature Portfolio policies, see our Editorial Policies and the Editorial Policy Checklist.

Statistics

For all statistical analyses, confirm that the following items are present in the figure legend, table legend, main text, or Methods section.

n/a	Confirmed	
☐	☒	The exact sample size (*n*) for each experimental group/condition, given as a discrete number and unit of measurement
☐	☒	A statement on whether measurements were taken from distinct samples or whether the same sample was measured repeatedly
☐	☒	The statistical test(s) used AND whether they are one- or two-sided<br>*Only common tests should be described solely by name; describe more complex techniques in the Methods section.*
☒	☐	A description of all covariates tested
☐	☒	A description of any assumptions or corrections, such as tests of normality and adjustment for multiple comparisons
☐	☒	A full description of the statistical parameters including central tendency (e.g. means) or other basic estimates (e.g. regression coefficient) AND variation (e.g. standard deviation) or associated estimates of uncertainty (e.g. confidence intervals)
☐	☒	For null hypothesis testing, the test statistic (e.g. *F*, *t*, *r*) with confidence intervals, effect sizes, degrees of freedom and *P* value noted<br>*Give P values as exact values whenever suitable.*
☒	☐	For Bayesian analysis, information on the choice of priors and Markov chain Monte Carlo settings
☒	☐	For hierarchical and complex designs, identification of the appropriate level for tests and full reporting of outcomes
☒	☐	Estimates of effect sizes (e.g. Cohen's *d*, Pearson's *r*), indicating how they were calculated

Our web collection on statistics for biologists contains articles on many of the points above.

Software and code

Policy information about availability of computer code

Data collection

The data were collected by using:
1. CFX96 Touch Real-Time PCR Detection System (Bio-Rad)
2. Car Zeiss Fluorescent Microscope (LSM980)
3. Nikon Bright Field Microscope (ECLIPSE Ts2-FL)
4. Agilent Novocyte Advanteon (Agilent Technologies)
5. Transmission Electron Microscope (TEM; Thermo Fisher Scientific, Talos L120C G2)
6. Sony MA900 sorter (Sony, Cat#LE-MA900CF)
7. CuntessTM 3 Automated Cell Counter (Life Technologies)
8. DNBSEQ-T7 Sequencer
9. Rheometer (Anton Paar, Cat#MCR 302e)
10. Biological Force Instrument (EFL, Cat#T5600)
11. Field-emission Scanning Electron Microscope (FE-SEM; Zeiss GeminiSEM 500)
12. Spectrophotometer (Thermoscientific 51119670)
13. Spinning-Disk Confocal Imaging System (Andor Dragonfly 200)

Data analysis

The data were analysed using ImageJ, GraphPad Prism v8.0.2, Carl Zeiss Zen 2011 SP6 (blue), FlowJo_V10, NovoExpress (Agilent Novocyte Advanteon), DNBelabTM C Series Single-cell RNA Analysis Report v2.1.1, CellRanger v8.0.0, DoubletFinden v2.0.4, Doublet Detection v4.2, scDblFinder v1.18.0, Scrublet v0.2.3, Gruffi v1.5.5, R, CytoTRACE2 v1.0.0, STREAM v1.1, MShot Image Analysis System v1.1.4, Imaris v9.9.0, Fusion 2.2.0.49.

For manuscripts utilizing custom algorithms or software that are central to the research but not yet described in published literature, software must be made available to editors and reviewers. We strongly encourage code deposition in a community repository (e.g. GitHub). See the Nature Portfolio guidelines for submitting code & software for further information.

Data

Policy information about availability of data

All manuscripts must include a data availability statement. This statement should provide the following information, where applicable:

- Accession codes, unique identifiers, or web links for publicly available datasets
- A description of any restrictions on data availability
- For clinical datasets or third party data, please ensure that the statement adheres to our policy

The scRNA-seq datasets generated in this study have been deposited in the NCBI Gene Expression Omnibus (GEO) database under accession codes GSE293170 (hAWOs) and GSE292932 (hALOs). Processed bulk RNA-seq results generated in this study are provided in Source Data file. All code used to generate the data in this manuscript is adapted from base R, Seurat, or other publicly available packages. The code is publicly available and has been deposited in GitHub at [https://github.com/EddieLv/1-Extracellular-stiffness-regulates-site-specific-lung-development]. The specific version of the code associated with this publication is archived in Zenodo and is accessible via [https://doi.org/10.5281/zenodo.20507306]

Research involving human participants, their data, or biological material

Policy information about studies with human participants or human data. See also policy information about sex, gender (identity/presentation), and sexual orientation and race, ethnicity and racism.

Reporting on sex and gender	N/A
Reporting on race, ethnicity, or other socially relevant groupings	N/A
Population characteristics	N/A
Recruitment	N/A
Ethics oversight	N/A

Note that full information on the approval of the study protocol must also be provided in the manuscript.

Field-specific reporting

Please select the one below that is the best fit for your research. If you are not sure, read the appropriate sections before making your selection.

☒ Life sciences ☐ Behavioural & social sciences ☐ Ecological, evolutionary & environmental sciences

For a reference copy of the document with all sections, see nature.com/documents/nr-reporting-summary-flat.pdf

Life sciences study design

All studies must disclose on these points even when the disclosure is negative.

Sample size	Sample size are ranging from 6-9 per group for most RT-qPCR experiments.
Data exclusions	No data was excluded.
Replication	Each replicate was performed with 2, 3, or more technical repeats or was done in biological triplicate according to or exceeding standards in the field. In all cases,representative figure displays were appropriately indicated.
Randomization	Randomization was not a relevant feature as we were differentiating cells batch by batch, data were collected from all batches.
Blinding	Blinding was not used for this study.

Reporting for specific materials, systems and methods

We require information from authors about some types of materials, experimental systems and methods used in many studies. Here, indicate whether each material, system or method listed is relevant to your study. If you are not sure if a list item applies to your research, read the appropriate section before selecting a response.

Materials & experimental systems

n/a	Involved in the study
☐	☒ Antibodies
☐	☒ Eukaryotic cell lines
☒	☐ Palaeontology and archaeology
☒	☐ Animals and other organisms
☒	☐ Clinical data
☒	☐ Dual use research of concern
☒	☐ Plants

Methods

n/a	Involved in the study
☒	☐ ChIP-seq
☐	☒ Flow cytometry
☒	☐ MRI-based neuroimaging

Antibodies

Antibodies used

Primary antibodies
Anti-TTF1/Nkx2-1 antibody [EP1584Y], Abcam (Ab76013)
Anti-TTF1/Nkx2-1 antibody [SP141], Abcam (Ab227652)
Anti-SOX9 antibody [3C10] - BSA and Azide free, Abcam (Ab76997)
Human SOX9 Antibody, R&D Systems (AF3075)
Anti-SOX2 antibody, Abcam (Ab239218)
Sox-2 Antibody (E-4), Santa Cruz Biotechnology (Sc-365823)
Human UGRP1/SCGB3A2 Antibody, R&D Systems(AF3545)
Uteroglobin/SCGB1A1/CC10 Antibody (E-11), Santa Cruz Biotechnology(Sc-365992)
Anti-Mucin 5AC antibody [EPR16904], Abcam (Ab198294)
Anti-Mucin 5AC antibody [45M1], Abcam (Ab3649)
MUCSAC Mouse Monoclonal Antibody [G6-H8], HUABIO (EM1707-36)
Monoclonal Anti-Acetylated Tubulin, Antibody produced in mouse, Sigma-Aldrich (T7451)
FOXJ1 Monoclonal Antibody (2A5), eBioscience/invitrogen (14-9965-82)
Cytokeratin 5 Antibody (RCK103), Santa Cruz Biotechnology (Sc-32721)
SP-B Antibody (F-2), Santa Cruz Biotechnology (Sc-133143)
Anti-Pro-SP-C - Rabbit, n-terminal, Seven Hills Bioreagents (WRAB-9337)
Human/Mouse/Rat RAGE/AGER Antibody, R&D Systems (AF1145)
ABCA3 Polyclonal Antibody, Proteintech (27450-1-AP)
YAP (63.7), Santa Cruz Biotechnology (Sc-101199)
Anti-WWTR1, antibody produced in rabbit, Sigma-Aldrich (HPA007415)
ZO-1 Monoclonal Antibody (ZO1-1A12), Invitrogen (33-9100)
Purified Mouse Anti-E-Cadherin 36/E-Cadherin, BD Transduction Laboratories (610181)
Human/Mouse E-Cadherin Antibody,QQ R&D Systems (AF748-SP)
SARS-CoV/SARS-CoV-2 Nucleocapsid Antibody, Rabbit PAb, Antigen Affinity Purified 40143, SinoBiological (40143-T62)
Ki-67 Alexa 488 B56 100Tst, BD Transduction Laboratories (558616)
Phospho-YAP (Ser127) Antibody, Cell signaling (4911)
β-Tubulin Mouse mAb (HRP Conjugated), Beyotime (AF2839)
GAPDH Mouse Monoclonal Antibody, Beyotime (AF5009)

Secondary antibodies
Donkey anti-Rabbit IgG (H+L) Highly Cross-Adsorbed Secondary Antibody, Alexa Fluor 488, Invitrogen (A-21206)
Donkey anti-Goat IgG (H+L) Cross-Adsorbed Secondary Antibody, Alexa Fluor 488, Invitrogen (A-11055)
Donkey anti-Mouse IgG (H+L) Highly Cross-Adsorbed Secondary Antibody, Alexa Fluor 488, Invitrogen (A-21202)
Donkey anti-Mouse IgG (H+L) Highly Cross-Adsorbed Secondary Antibody, Alexa Fluor 546, Invitrogen (A-10036)
Donkey anti-Rabbit IgG (H+L) Highly Cross-Adsorbed Secondary Antibody, Alexa Fluor546, Invitrogen (A-10040)
Donkey anti-Goat IgG (H+L) Cross-Adsorbed Secondary Antibody, Alexa Fluor 546, Invitrogen (A-11056)
Donkey anti-Mouse IgG (H+L) Highly Cross-Adsorbed Secondary Antibody, Alexa Fluor 647, Invitrogen (A-31571)
Donkey Anti-Rabbit IgG H&L (Alexa Fluor 647), Abcam (Ab150075)
Donkey Anti-Goat IgG H&L (Alexa Fluor 647) preadsorbed, Abcam (Ab150135)
HRP-Donkey Anti-Goat IgG (H+L), proteintech (SA00001-3)
HRP-conjugated Affinipure Goat Anti-Mouse IgG (H+L), proteintech (SA00001-1)
HRP-conjugated Affinipure Goat Anti-Rabbit IgG (H+L), proteintech (SAO0001-2)

Validation

Anti-TTF1/Nkx2-1 antibody [EP1584Y], Abcam (Ab76013), Human SOX9 Antibody, R&D Systems (AF3075), Monoclonal Anti-Acetylated Tubulin, Antibody produced in mouse, Sigma-Aldrich (T7451), have been verified in Han et al., STAR Protocols 2022.
Anti-TTF1/Nkx2-1 antibody [SP141], Abcam (Ab227652) has been verified in Hauer C et al., Physiol Rep 2025.
Anti-SOX9 antibody [3C10] - BSA and Azide free, Abcam (Ab76997) has been verified in Nakamura K et al., Int J Mol Sci 2025.
Anti-SOX2 antibody, Abcam (Ab239218) has been verified in Zhang H et al., STAR Protocols 2025.
Sox-2 Antibody (E-4), Santa Cruz Biotechnology (Sc-365823) has been verified in Sano E et al., iScience 2021.
Human UGRP1/SCGB3A2 Antibody, R&D Systems(AF3545), FOXJ1 Monoclonal Antibody (2A5), eBioscience/invitrogen (14-9965-82), Human/Mouse/Rat RAGE/AGER Antibody, R&D Systems (AF1145) have been verified in Kadur Lakshminarasimha Murthy P et al., Nature 2022.
Uteroglobin/SCGB1A1/CC10 Antibody (E-11), Santa Cruz Biotechnology(Sc-365992) has been verified in Jeon HY et al., The EMBO Journal 2022.
Anti-Mucin 5AC antibody [EPR16904], Abcam (Ab198294) has been verified in Ekanger CT et al., Front Immunol 2025.
Anti-Mucin 5AC antibody [45M1], Abcam (Ab3649) has been verified in Geiger RM et al., Front Pharmacol 2025.
MUCSAC Mouse Monoclonal Antibody [G6-H8], HUABIO (EM1707-36) has been verified in Martin et al., Biochemical Journal 2026.

Cytokeratin 5 Antibody (RCK103), Santa Cruz Biotechnology (Sc-32721) has been verified in White SR et al., Respir Res 2013.
SP-B Antibody (F-2), Santa Cruz Biotechnology (Sc-133143) has been verified in Magro-Lopez E et al., Stem Cell Res Ther 2018.
Anti-Pro-SP-C - Rabbit, n-terminal, Seven Hills Bioreagents (WRAB-9337), Purified Mouse Anti-E-Cadherin 36/E-Cadherin, BD Transduction Laboratories (610181) have been verified in Miller AJ et al., Nat Protoc 2019.
YAP (63.7), Santa Cruz Biotechnology (Sc-101199), Phospho-YAP (Ser127) Antibody, Cell signaling (4911) have been verified in Nantie LB et al., Development 2018.
Anti-WWTR1, antibody produced in rabbit, Sigma-Aldrich (HPA007415) has been verified in Hicks-Berthet J et al., Cell Reports 2021.
ZO-1 Monoclonal Antibody (ZO1-1A12), Invitrogen (33-9100) has been verified in Co JY et al., Cell Rep 2019.
Human/Mouse E-Cadherin Antibody,QQ R&D Systems (AF748-SP) has been verified in Pei R et al., Protein Cell 2021.
SARS-CoV/SARS-CoV-2 Nucleocapsid Antibody, Rabbit PAb, Antigen Affinity Purified 40143, SinoBiological (40143-T62) has been verified in Becker ME et al., Nature Communications 2024.
GAPDH Mouse Monoclonal Antibody, Beyotime (AF5009) has been verified in Zeng C et al., Phytother Res 2023.

Eukaryotic cell lines

Policy information about cell lines and Sex and Gender in Research

Cell line source(s)	H1 (WiCell Research Institute)
Authentication	None of the cells used were authenticated
Mycoplasma contamination	Cell lines were tested for mycoplasma contamination
Commonly misidentified lines (See ICLAC register)	No commonly misidentified cell lines were used in the study

Plants

Seed stocks	*Report on the source of all seed stocks or other plant material used. If applicable, state the seed stock centre and catalogue number. If plant specimens were collected from the field, describe the collection location, date and sampling procedures.*
Novel plant genotypes	*Describe the methods by which all novel plant genotypes were produced. This includes those generated by transgenic approaches, gene editing, chemical/radiation-based mutagenesis and hybridization. For transgenic lines, describe the transformation method, the number of independent lines analyzed and the generation upon which experiments were performed. For gene-edited lines, describe the editor used, the endogenous sequence targeted for editing, the targeting guide RNA sequence (if applicable) and how the editor was applied.*
Authentication	*Describe any authentication procedures for each seed stock used or novel genotype generated. Describe any experiments used to assess the effect of a mutation and, where applicable, how potential secondary effects (e.g. second site T-DNA insertions, mosiacism, off-target gene editing) were examined.*

Flow Cytometry

Plots

Confirm that:

☒ The axis labels state the marker and fluorochrome used (e.g. CD4-FITC).

☒ The axis scales are clearly visible. Include numbers along axes only for bottom left plot of group (a 'group' is an analysis of identical markers).

☒ All plots are contour plots with outliers or pseudocolor plots.

☒ A numerical value for number of cells or percentage (with statistics) is provided.

Methodology

Sample preparation

Organoids were dissociated into single cells using TrypLE (Thermo Fisher, Cat12605028) at 37°C for 5-15 minutes, withgentle pipetting every 6 minutes to minimize cell clumping. An equal volume of DMEM/F-12 medium supplemented with 10uM Y-27632 was added to terminate the digestion. Single cells were centrifuged at 300x g for 3 minutes.

For flow cytometry analysis, singal cells were fixed in 4% PFA for 30 minutes. Cells were permeabilized, blocked, and stainedwith primary and secondary antibodies according to the IF staining protocol. Stained cells were resuspended in sorting buffer(1 nM EDTA+ 1% BSA in PBS) and passed through a 70um cell stainer (BD Falcon, Cat#352235) into FACS tubes andprotecting from light. The stained single cells were ran and analyzed using Agilent Novocyte Advanteon (AgilentTechnologies).

For FACS the dissociation protocol of organoids was the same as above except all steps were performed on ice to preserve cell viabilily. After digestion, cells were incubated with EPCAM antibodies (Bioegend, Cat369810) on ice for 30 minutes.Single cells with EPCAM positive were sorted using Sony MA900 sorter (Cat#LE-MA900FP) and collected in sorting buffer withY-27632. Data were processed with NovoExpress (Agilent Novocyte Advanteon) and Flowlo_V10 software.

Instrument

Cell stainer (BD Falcon, Cat#352235)

Instrument	Agilent Novocyte Advanteon (Aglent Technologies)<br>Sony MA900 sorter (Cat#LE-MA900FP)
Software	NovoExpress (Agilent Novocyte Advanteon) and Flowlo_V10 software
Cell population abundance	At least 10,000 cells
Gating strategy	Using the FSC/SSC gating, debris was removed by gating on the main cell population.Positivity threshold for cells at each stage was defined on the basis of isotype control stained sample (same stages). Identicalpositivity threshold was applied to all samples with the same stage.

☒ Tick this box to confirm that a figure exemplifying the gating strategy is provided in the Supplementary Information.
