# Supplementary material for: Extracellular matrix stiffness directs region-specific lung epithelial differentiation revealed by hPSC-derived lung organoids
Source: Nat Commun. 2026 Jul 17;17:8757. doi: 10.1038/s41467-026-75663-9 (PMC13493954; doi:10.1038/s41467-026-75663-9)
Supplement: Supplementary file 2 [file 41467_2026_75663_MOESM11_ESM.pdf]

nature portfolio

Peer Review File

Extracellular matrix stiffness directs region-specific lung epithelial differentiation revealed by hPSC-derived lung organoids

Corresponding Author: Professor Huisheng Liu

This file contains all reviewer reports in order by version, followed by all author rebuttals in order by version.

Version 0:

Reviewer comments:

Reviewer #1

(Remarks to the Author)
This study explores how extracellular matrix (ECM) stiffness influences region-specific lung development, using human embryonic stem cell-derived lung organoids as a model. Using stiffness tunable hydrogels they show that ECM stiffness is a key factor in regulating lung epithelial cell fate and region-specific lung development. Stiffer ECM promotes lung progenitor cells and affects the proximal-to-distal specification and alveolar maturation. Proximal airway cells emerge in stiffer ECM whereas distal airway cells favor a softer matrix. Alveolar cell maturation (e.g., AT2 to AT1 transition) is enhanced with intermediate to high stiffness. ECM stiffness mediates cell differentiation through pathways such as Hippo, TGF-β, Wnt, and hypoxia signaling. Finally the authors show that stiffness-tunable organoids effectively modeled region-specific infection, with Omicron BA.1.1 targeting proximal airways and Delta variant infecting alveolar tissues. The study offers an in vitro platform for understanding lung development, disease pathogenesis, and drug screening. Overall, this is an interesting study. However, it is unclear whether the anti-Yap1 antibody used is specific for Yap1 or whether it also detects Taz. This is rather important as Yap and Taz play somewhat opposite roles in the lung and Taz but not Yap has been demonstrated to drive AT1 cell differentiation in mouse (https://pubmed.ncbi.nlm.nih.gov/39639058/). We therefore would implore the authors to investigate Taz as well using (rabbit anti-WWTR1 Taz clone E115179; HPA007415; RRID:AB_1080602; Prestige Antibodies) and expand Fig.6G&H.

(Remarks on code availability)

Reviewer #2

(Remarks to the Author)
The manuscript by Zhiying Liao et al. titled "Extracellular Matrix Stiffness Regulates Region-Specific Lung Development" presents interesting insights into how ECM stiffness, modulated through GelMA hydrogels, influences lung epithelial cell fate and organoid patterning. However, there are several areas, both conceptual and technical, that require clarification and improvement to strengthen the manuscript's conclusions.

Major Comments
1. The title implies that this study focusses on processes related to lung development, however this focus does not come across in the presentation of the data or the selected models for experimental approaches. Thus, there is no clear theme or story told by the data presentation in this manuscript.
2. While the SARS-CoV-2 infection exploration with the model adds translational interest, it appears somewhat disconnected from the manuscript’s core developmental focus, and therefore is a distraction within the data story.
3. The selection of G30, G60, and G90 hydrogels needs justification. Specifically, G90 (~18–20 kPa) approaches the stiffness of fibrotic lung tissue, which may model pathological rather than physiological conditions. Please clarify whether these values reflect in vivo gradients across healthy airways and alveolar regions, especially during development.

4. How many organoids were observed across the different GelMA stiffness conditions? Given that G90 lacks the mechanical characteristics (viscoelasticity) typically necessary for supporting cell viability and organoid formation, how do you explain its ability to sustain organoid development in the presented study?
5. Many previous studies have shown that hydrogel stiffness often influences the cell proliferation rate, however, your results showed differently. Could you clarify this and provide a possible explanation for why your findings differ from earlier reports?
6. It is surprising that distal alveolar cells didn't favor soft ECM conditions, as this contradicts typical in vivo lung biology and is inconsistent with the airway data presented. Could you clarify the physiological relevance of this finding and what it suggests about the role of ECM stiffness in regulating alveolar cell fate?
7. Experimental Details:
Please provide more detailed methodological information, particularly regarding:
o Time point of live/dead staining
o Number of images captured per sample, how the images to be analyzed were chosen?
o Number of cells/images used for each quantification
o Number of biological replicates that were included per group for the sequencing analysis?
o How many reads/cell were aimed for for the sequencing?
o In Figure 1h, could you clarify which group comparisons were used to generate the enriched pathways shown?
8. The identification of neuronal populations within the organoid system raises some concern. If the cells were sorted to be EpCAM+ prior to sequencing then how were mesenchymal and neuronal cells captured in the sequencing population? How pure was the cell sorting? What neuronal markers were used to support this annotation? Additional validation or clarification is necessary.
9. Beyond YAP1 localization by staining, were any reporter assays or phosphorylation markers used to confirm Hippo pathway activation?
10. In line 149, the statement of "stiffness-driven mechanisms" requires a stronger link to the data. Could you elaborate on how this conclusion can be supported?
11. More information is needed about your data integration steps in the single-cell RNA-seq analysis, as all subsequent downstream analyses depend on this. It is not clear what the authors refer to as a "Biological relevant feature" or a "noisy cluster". The authors should be more concrete about this.
12. Further , I would be very hesitant to annotate neurons in an organoid culture, and also did not find any evidence presented by the authors that this could be the case. It is not clear is being defined as neuronal markers.
13. The authors state they use gene lists for annotation, but do not specify which gene lists were used for annotation. The lists used need to be provided for the reader to be able to understand the annotation and whether this makes sense.
14. A supplemental figure with umaps colored by cell-specific markers would help here, but maybe also a dot plot with the authors markers per cell type would be really helpful for the reader to understand how the authors have processed these data.
15. The conclusion in lines 385–387 appears to overstate the strength of the findings. This reviewer recommends rephrasing this to better match the data presented.
16. Given that GelMA was crosslinked for 30 seconds, some uncrosslinked material may remain. Could this influence cell behavior beyond stiffness?

Minor Comments
1. The lung environment involves more than ECM and epithelial cells (e.g., endothelial cells, stroma cells, immune components). It would be helpful to acknowledge this complexity in the Introduction.
2. The use of terms like "pleura" may cause confusion since the model focuses on airway and alveolar structures. Please clarify and ensure terminology is used accurately throughout.
3. Please ensure all abbreviations (e.g., ECM, AFE, AT2) are fully defined at first use.
4. Multiple grammatical errors and incomplete sentences are present throughout the manuscript (e.g., lines 371–373, 375–376). A thorough language edit is recommended.
5. TEM imaging alone cannot confirm functionality. Please clarify how function was inferred from this result. Please clarify if SEM or TEM imaging was used (and when) in the study.
6. The statement on line 325 overstates the interpretation of the presented data. Consider softening the conclusion to align with the evidence presented, and consider the physiological relevance.
7. You mention that mesenchymal-secreted ECM proteins alter lung ECM mechanics (Lines 330–332). Were any of these components included in your culture conditions?
8. The claim that decellularization disrupts ECM structure and stiffness is not entirely accurate (Line 349). This statement should be supported with evidence. Reference to prior work in this area should be made.
9. The mention of hyaluronic acid (HA) (Lines 350–351) promoting bronchiolar progenitors seems out of context. Consider removing or better integrating this information.
10. Please revise incomplete sentences in lines 371–373 and 375–376 to ensure clarity.

(Remarks on code availability)

Reviewer #3

(Remarks to the Author)
I co-reviewed this manuscript with one of the reviewers who provided the listed reports. This is part of the Nature

Communications initiative to facilitate training in peer review and to provide appropriate recognition for Early Career Researchers who co-review manuscripts.

(Remarks on code availability)

Reviewer #4

(Remarks to the Author)
Liao et al investigated how extracellular matrix (ECM) stiffness regulates region-specific differentiation of human pluripotent stem cell (hPSC)-derived lung organoids. By establishing a stiffness-tunable GelMA hydrogel platform, the authors provide a valuable model system to dissect how mechanical cues influence proximal-to-distal airway and alveolar epithelial fate decisions. The integration of mechanical modeling with transcriptomic and single-cell analyses offers new insights into the ECM-related mechanisms underlying lung development, particularly involving the Hippo, Wnt, TGF-β, and hypoxia pathways. The data are clearly delivered and fully support the conclusion. Herein, several issues may have to be addressed to improve the scientific vigor of the manuscript.

1. The authors mention that GelMA stiffness ranges from 3 to 15 kPa to mimic physiological lung stiffness. It would be helpful to more explicitly discuss how these stiffness levels compare to the in vivo stiffness ranges of the proximal airways, bronchioles, and alveoli. This would enhance the physiological relevance of the model.

2. The authors may have to be cautious using “lung”. E.g., line 81, consider replacing “the lung” with “the human respiratory tract”. Line 85, revise “the lung epithelium” as “the airway epithelium”; and delete “lung” line 111. The authors are suggested to check the whole manuscript to ensure the correct usage of “respiratory” and “lung”.

3. I am confused about the usage of “secretory cells” by the authors. In line 86, it may refer to goblet cell and club cell. In line 162, UGRP1 and uteroglobin are marker genes of club cells. please clarify.

4. While focusing on ECM stiffness as a key regulator of epithelial cell fate, the authors may have to acknowledge that other aspects of the microenvironment, e.g. ECM remodeling, matrix topography etc, also play critical roles. The authors are suggested to briefly discuss these factors in the manuscript (e.g., Jones et al., 2024, Science; Courbot et al., 2025, NPJ Regenerative Medicine).

5. The authors may discuss how hiPSC-derived region-specific lung organoids compare to adult stem cell-derived respiratory organoids in cellular composition and biological relevance. Suggested references: Chiu et al., 2022 Cell Discovery; Li et al., 2024 Nature Communications.

6. The demonstration of region-specific SARS-CoV-2 infection highlights the physiological relevance of the lung organoids. The authors may consider extending the discussion by linking ECM stiffness-related pulmonary diseases, such as IPF and COPD.

7. Minor issues
1) Line 54. This study discover.
2) Line 93. Please specify the stage for “budding morphogenesis in embryonic lungs.”
3) Please clarify figure legends (e.g., arrows in Figures 2i and 3h) and define abbreviations (e.g., “LBs,” “TJs”) at first mention.
4) Line 131. Exhibited induced?

(Remarks on code availability)

Version 1:

Reviewer comments:

Reviewer #1

(Remarks to the Author)
The authors have addressed my concerns.

(Remarks on code availability)

Reviewer #2

(Remarks to the Author)
Thank you for your extensive responses to the previous round of comments. We still have several remaining questions:
1. Title

The revised title is not a complete or precise phrase. “Extracellular stiffness” is ambiguous; it should read “Extracellular matrix stiffness.”
2. SARS-CoV-2 data as physiological validation
Using SARS-CoV-2 data to demonstrate physiological relevance remains unconvincing. Although the authors base this work on a finding that has been published, this approach is not widely adopted or well established for model validation. Please consider adding more physiologically relevant and established methods to substantiate the model.
3. RGD density across GelMA with different methacrylation
The statement that RGD density is the same in G30, G60, and G90 is inaccurate. While the sequence-level RGD count may be similar, differing degrees of methacrylation alter the GelMA network structure and thus the degree of bioavailable/accessible RGD for cells.
4. Thickness vs. stiffness inconsistency
You state thicker GelMA leads to higher stiffness; however, in the rebuttal Figure 1, panel i (2.5 mm) shows G90 at ~22–23 kPa, whereas panel l (5 mm) shows G90 at ~17–18 kPa. Please clarify this. It is unclear why the stiffness, an inherent property of a material, would change when measuring hydrogels of differing thicknesses. An explanation as to why these data changed is necessary.
5. Targeted airway region
Which small-airway compartment is the model intended to mimic? Note that cartilage is present in the trachea and bronchi but absent from bronchioles (small airways). Please clarify anatomical scope and relevance.
6. Mechanical cues beyond stiffness
In your response to Question 6 (p.13), you note that elasticity and loading contribute to in vivo mechanosignaling in alveoli. However, the experimental design in this study did not incorporate elasticity/loading but only adjusted stiffness. Therefore the relevance of this response is not immediately obvious. This is not convincing for the conclusion and discussion.
7. The discussion points relating to cortical organoids for assessing optimal neural development do not provide substantive support for the points related to this manuscript. Similarly, pleura-to-alveolar septa stiffness gradients refer to gradients within the alveolar compartment, and thus are difficult to align with the current thesis that there is an altered response to stiffness between the airway and alveolar compartments.
8. scRNA-seq replication
With only one biological replicate, the scRNA-seq evidence is insufficient to draw any conclusions.

Minor comments
1. In the Discussion, the sentence regarding fetal and neonatal lungs (“…alveoli continue to mature postnatally under respiratory forces…”) should be removed, as the study focuses on region-specific effects and this statement is not directly relevant.
2. Please remove or revise the sentence in the discussion:
“However, the development and maturation of the alveolar epithelium (air exchange under the respiratory system) are not simply better, and the external force seems to play an essential role in signaling pathway that determine epithelial fates.” As currently phrased, it is unclear and insufficiently relevant to the model used.

(Remarks on code availability)

Reviewer #3

(Remarks to the Author)
I co-reviewed this manuscript with one of the reviewers who provided the listed reports. This is part of the Nature Communications initiative to facilitate training in peer review and to provide appropriate recognition for Early Career Researchers who co-review manuscripts.

(Remarks on code availability)

Reviewer #4

(Remarks to the Author)
The authors have adequately addressed my questions and revised the manuscript accordingly.

(Remarks on code availability)

Version 2:

Reviewer comments:

Reviewer #5

(Remarks to the Author)
In my opinion, the authors have very thoroughly and comprehensively addressed Reviewer 2’s stated concerns, and should be commended for the diligence and care in conducting new experiments and revising the wording of the manuscript to

support the claims made.

Open Access This Peer Review File is licensed under a Creative Commons Attribution 4.0 International License, which permits use, sharing, adaptation, distribution and reproduction in any medium or format, as long as you give appropriate credit to the original author(s) and the source, provide a link to the Creative Commons license, and indicate if changes were made.
In cases where reviewers are anonymous, credit should be given to 'Anonymous Referee' and the source.
The images or other third party material in this Peer Review File are included in the article's Creative Commons license, unless indicated otherwise in a credit line to the material. If material is not included in the article's Creative Commons license and your intended use is not permitted by statutory regulation or exceeds the permitted use, you will need to obtain permission directly from the copyright holder.
To view a copy of this license, visit https://creativecommons.org/licenses/by/4.0/

We sincerely thank the reviewers for their insightful comments and valuable suggestions that have helped us to improve and refine our manuscript. As a result of addressing all the comments from the reviewers, we have modified a total of 24 figure panels (9 in the main figures and 15 in the supplementary figures), added a total of 15 new panels (2 to the main figures and 13 to the supplementary figures), added 2 new supplementary tables and 1 supplementary data 1, provided more detailed methods, reorganized the results and expanded the discussion.

We have addressed each of the concerns raised by the reviewers and have added new data, analysis, and we have modified the text to clarify specific issues as follows:

1) To justify whether Yap and Taz play opposite roles in the AT1 and AT2 cell fate determination, we utilized TAZ antibody co-stained with YAP and AT1 (AGER) or AT2 (SFTPB/SFTPC) (Fig. 5g, h; Supplementary Fig. 12). Our results indicate TAZ determines AT1 differentiation while cytoplasm-to-nuclear translocation of YAP accompanies the AT2-to-AT1 transition. Analysis of YAP phosphorylation further supports increased cytoplasm-to-nuclear translocation of YAP with increasing stiffness (Fig. 5i, j).

2) Regarding the concern of the title, we have changed the title by replacing "development" with "differentiation" and added organoid model, now the title is "Extracellular stiffness directs region-specific lung epithelial differentiation revealed by hPSC-derived lung organoids". To maintain the manuscript's core focus, we moved SARS-CoV-2 section to the end of the Results section.

3) To justify the selection of stiffness-tunable GelMA hydrogels: a) We detailed mechanical characterization of GelMA (Supplementary Fig. 1b-d) to demonstrate the physiologically relevant to the proximal to distal respiratory system; b) We added cell viability and differentiation assays with hydrogel extracts to show the hydrogel residual did not affect cell viability or lineage differentiation (Supplementary Fig. 2e-g); c) We analyzed expression of proliferation markers across stiffness conditions (G30, G60 and G90) in both bulk- and scRNA-seq datasets and found no significant differences in proliferation among these groups (Supplementary Figs. 3i, 7j, 11f).

4) To demonstrate the high-stiffness G90 is a necessary upper-stiffness boundary scale without generating pathological epithelial cells: a) We analyzed scRNA-seq datasets of hALOs and found that fibrosis-associated gene expression in G90 hALOs was comparable to levels in normal human lung epithelium and markedly lower than in fibrotic epithelium (Supplementary Fig. 11g); b) We also found G90 stiffness did not inhibit organoids' formation (Supplementary Fig. 2d).

5) To improve the quality of the transcriptomic analyses: a) We provided the gene lists for bulk RNA-seq and scRNA-seq to document marker selection and cell-type annotation (Supplementary Tables 3, 4; Supplementary Data 1); b) We added UMAP plots showing lineage-specific marker expression to clarify data processing and annotation (Supplementary Figs. 6d, 10d); c) We reclassified the neuronal population as pulmonary neuroendocrine cells because in our scRNA-seq dataset, these cells are EpCAM positive (Fig. 3c; Supplementary Fig. 6; Fig. 5b; Supplementary Fig. 10); d) We added flow cytometry (FACS) plots showing EpCAM gating strategy and post-sort purity with mesenchymal and epithelial cells ratio (Supplementary Figs. 5 and 9).

6) To enrich the background and discussion, we added more details in response to the reviewers' comments, including: a) The lung's complex multicellular composition; b) The composition, architecture, and mechanical properties of the respiratory extracellular matrix (ECM) and their roles in epithelial fate determination; c) A comparison of lung organoids derived from pluripotent stem cells versus adult stem cells; d) The relevance of ECM stiffness for modeling stiffness-related lung diseases (e.g. IPF and COPD).

7) The manuscript received professional English language editing from Springer Nature Author Services (SNAS).

A point-by-point response to each reviewer's comments is provided below. The references associated with this rebuttal letter can be found on p.33 of this document.

Reviewer #1 (Comments to the Author):

It is unclear whether the anti-Yap1 antibody used is specific for Yap1 or whether it also detects Taz. This is rather important as Yap and Taz play somewhat opposite roles in the lung and Taz but not Yap has been demonstrated to drive AT1 cell differentiation in mouse (https://pubmed.ncbi.nlm.nih.gov/39639058/). We therefore would implore the authors to investigate Taz as well using (rabbit anti-WWTR1 Taz clone E115179; HPA007415; RRID:AB_1080602; Prestige Antibodies) and expand Fig.6G&H.":

We thank the reviewer for this helpful suggestion. We performed co-immunofluorescent (IF) staining for YAP (mouse anti-YAP1 antibody, Santa Cruz Biotechnology, Sc-101199) and TAZ (rabbit anti-WWTR1/TAZ antibody, Sigma-Aldrich, HPA007415) alongside AGER or SFTPC or SFTPB, to verify antibody specificity and clarify their distinct roles of YAP and TAZ in alveolar epithelial differentiation across hydrogel stiffness. We found that:

1) YAP undergoes cytoplasm-to-nucleus translocation with increasing GelMA stiffness (Fig. 5g, h).

2) At G30, SFTPC$^+$ AT2 cells exhibit predominantly cytoplasmic YAP (Fig. 5g). At intermediate stiffness (G60), YAP translocated into the nucleus in SFTPC$^+$AGER$^+$ double-positive cells. At G90, more nuclear YAP enriched in AGER$^+$ cells that are SFTPC$^-$.

3) TAZ is strongly expressed in AGER$^+$ AT1 cells across all stiffness hydrogels, independent of YAP localization (Fig. 5h). With increasing stiffness, nuclear YAP more frequently colocalizes with TAZ, accompanied by stronger AGER expression.

4) These findings support YAP specific detection by the anti-YAP antibody. At lower stiffness, TAZ$^+$ nuclei lack nuclear YAP signal, and appreciable YAP/TAZ co-localization emerges only at intermediate-to-high stiffness, coincident with YAP cytoplasm-to-nuclear translocation.

We have expanded the figure accordingly (now Fig. 5g, h; original Fig. 6) and added a new supplementary figure (Supplementary Fig. 12) to present these data.

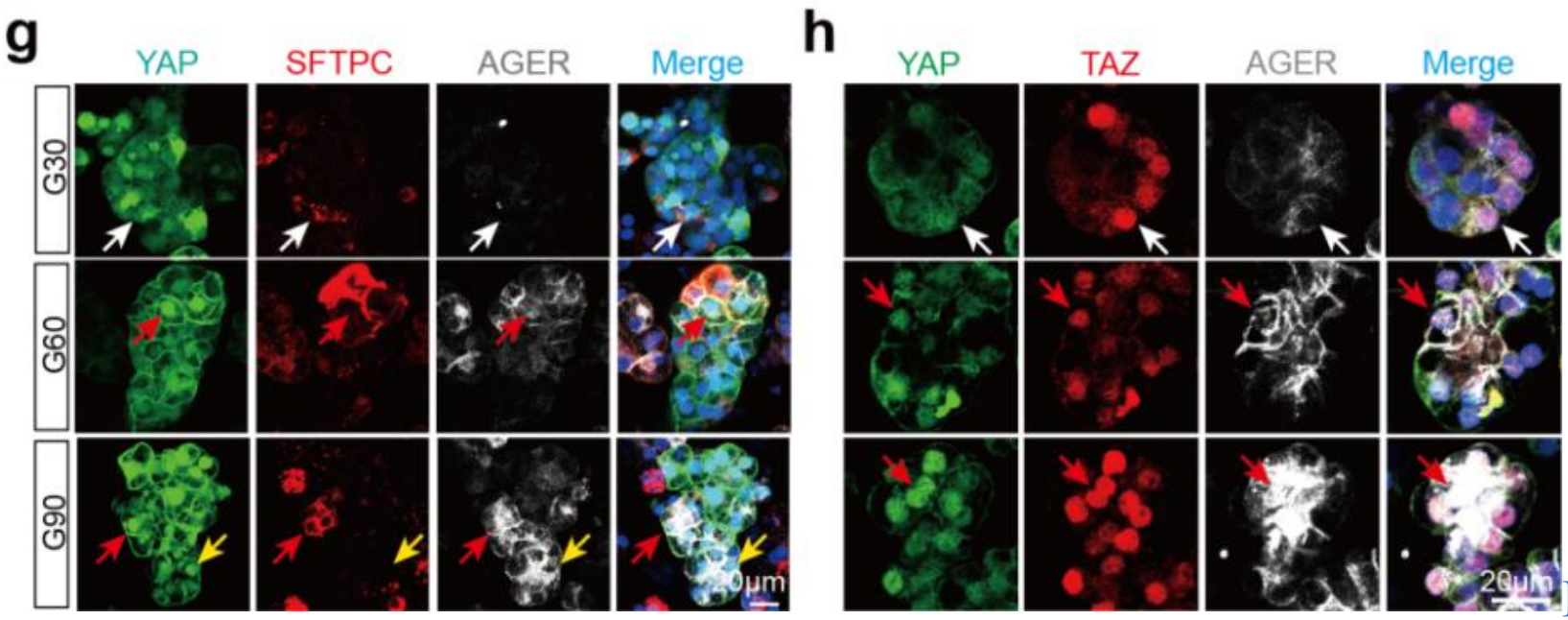

Fig. 5g&h (original Fig 6)

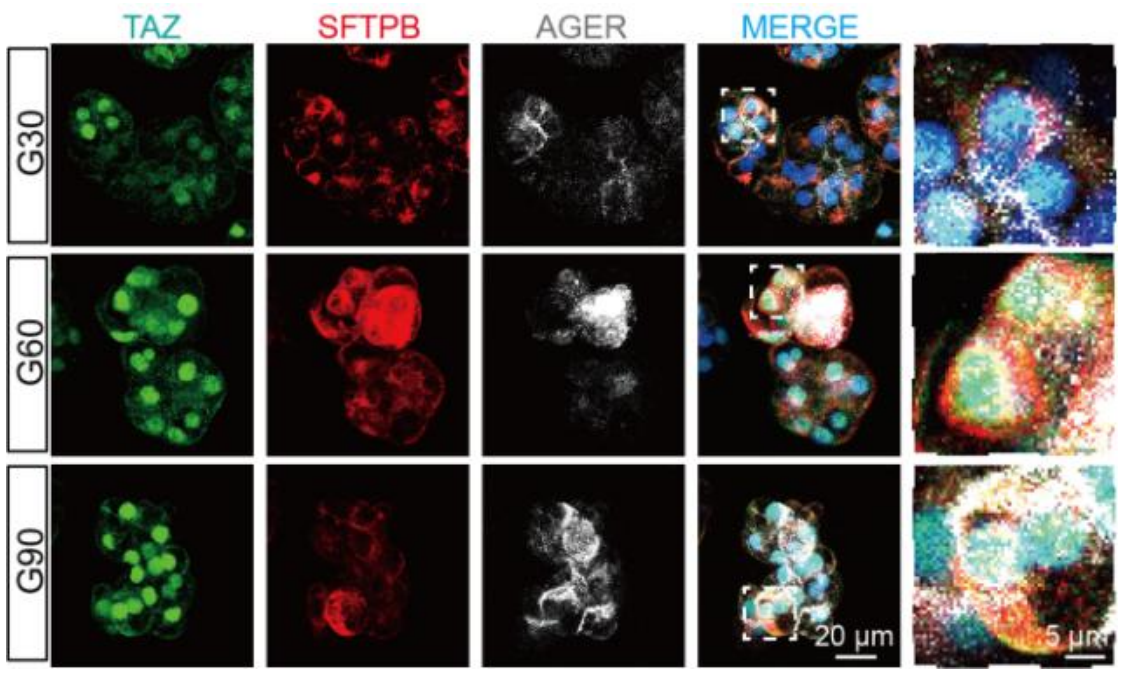

Supplementary Fig. 12

We now describe, in the "Results" section of the revised manuscript (page 11) that:

"Although both YAP and TAZ are mechanosensitive Hippo effectors, their specific contributions to alveolar epithelial differentiation are still debated[18, 43]. IF staining showed that YAP signaling translocated from the cytoplasm to the nucleus with increasing ECM stiffness (Fig. 5g, h). At lower stiffness (G30), YAP signal was predominantly cytoplasmic and colocalized with SFTPC+ AT2 cells (Fig. 5g, white arrow). With the increasing stiffness, YAP gradually translocated into the nucleus and colocalized with AGER+ AT1 (Fig. 5g, red arrow) with or without SFTPC+ AT2 (Fig. 5g, yellow arrow). In contrast, TAZ exhibited strong nuclear localization in AGER+ AT1 cells or AGER+SFTPB+ double positive cells across all stiffnesses (Fig. 5h; Supplementary Fig. 12). At low stiffness (G30), the nuclei of AGER+ AT1 cells were TAZ+ but YAP-, indicating that nuclear TAZ, rather than YAP, directly specified AT1 fate (Fig. 5h, white arrow). At higher stiffness (G60 and G90), YAP and TAZ colocalized in AT1 nuclei (Fig. 5h, red arrow). Our results indicate that TAZ acts as a primary determinant of AT1 identity whilst cytoplasmic-to-nuclear translocation of YAP contributes to AT2-to-AT1 transition under increased stiffness."

Reviewer #2 :

The manuscript by Zhiying Liao et al. titled "Extracellular Matrix Stiffness Regulates Region-Specific Lung Development" presents interesting insights into how ECM stiffness, modulated through GelMA hydrogels, influences lung epithelial cell fate and organoid patterning. However, there are several areas, both conceptual and technical, that require clarification and improvement to strengthen the manuscript's conclusions.

Major points:

1. The title implies that this study focusses on processes related to lung development, however this focus does not come across in the presentation of the data or the selected models for experimental approaches. Thus, there is no clear theme or story told by the data presentation in this manuscript.

We appreciate the reviewer's comment.

Our study utilizes a stiffness-tunable hydrogel in combination with human pluripotent stem cell-derived lung organoids as an *in vitro* biomimetic model to systematically interrogate how extracellular stiffness regulates early (lung progenitors formation) and late (airway epithelial patterning and alveolar fate specification and maturation) stages of lung generation. However, we acknowledge that our original title was overly broad and mislead to a less focused narrative. To make the scope explicit and align the title with the data and models used, we changed the title to:

"Extracellular stiffness directs region-specific lung epithelial differentiation revealed by hPSC-derived lung organoids."

2. While the SARS-CoV-2 infection exploration with the model adds translational interest, it appears somewhat disconnected from the manuscript's core developmental focus, and therefore is a distraction within the data story.

We greatly appreciate the reviewer's comment and concern.

1) To maintain a clear developmental trajectory throughout the manuscript, we have reorganized the Results section to first present the phenotypic and transcriptomic characterization of lung progenitors, airway and alveolar organoids and repositioned the SARS-CoV-2 infection section to the end. Now the "Results" section is ordered as below:

"2.1. High Extracellular Stiffness Promotes LPC Specification

2.2. Stiff to Soft Directs Proximal-to-distal hAWO Specification

2.3(2.5). ECM Stiffness Directs hAWOs Cell Fate via the Hippo/Hypoxia/Wnt

2.4(2.3). High ECM Stiffness Promotes hALO Differentiation and Maturation

2.5(2.6). ECM Stiffness Determines Alveolar Fates Via the Hippo and Wnt

2.6(2.4). SARS-CoV-2 Variants Recapitulate Physiological Infection Tropism in hHLOs"

2) We utilized the SARS-CoV-2 data function as evidence of physiological relevance that our region-specific organoids reproduce *in vivo* variant specific tropism. Chiu et al (Chiu et al., Cell Discov, 2022)[1] utilized wild type and Omicron to verify the physiological relevance of airway and alveolar organoids derived from adult stem cells, they stated that "Notably, the higher infectivity

and replicative fitness of the Omicron variant than an ancestral strain were accurately recapitulated in these optimized airway organoids." and "...alveolar organoids sustain a productive SARS-CoV-2 infection, albeit a lower replicative fitness was observed compared to that in airway organoids." We now describe, in the "Results" section of the revised manuscript (page 12) that:

"Previous studies have used SARS-CoV-2 variants to validate the physiological relevance of adult stem cell–derived hAWOs and hALOs[47]. ... Accordingly, to establish physiological relevance, we challenged stiffness-guided, region-specific lung organoids with Delta and Omicron to test whether our organoids recapitulate region-specific epithelial properties."

3. 1）The selection of G30, G60, and G90 hydrogels needs justification. 2）Specifically, G90 (~18–20 kPa) approaches the stiffness of fibrotic lung tissue, which may model pathological rather than physiological conditions. 3）Please clarify whether these values reflect in vivo gradients across healthy airways and alveolar regions, especially during development.

We appreciate the reviewer for raising these important points.

3.1 The selection of G30, G60, and G90 hydrogels needs justification.

1) Hydrogels were prepared by modulating the degree of methacrylation ratio

We prepared GelMA hydrogels of different stiffness by varying the degree of methacrylation while keeping the GelMA concentration (8% w/v), photoinitiator (LAP, 0.25%) and UV crosslinking time (30s) the same. This design ensures that stiffness is the only intentionally varied parameter, avoiding confounding effects from changes in gelatin/RGD content, photoinitiator concentration, or UV exposure time that could otherwise influence cell adhesion or viability. This approach is consistent with prior work (Li et al., Polymers, 2016)[2] and they stated that: "GelMA hydrogels with different stiffness and the same RGD density were fabricated by adjusting the degree of methacryloyl substitution of same concentration of gelatin. The Young's modulus increased with the degree of functionalization." To clarify the strategy of G30, G60 and G90 selection in the revised manuscript, we now describe in the "Results" section (page 5) that:

"Three different stiffnesses of GelMA hydrogels were prepared with different degrees of methacrylation (MA, 30%, 60%, and 90%) and consistent gelatin concentration (8% w/v) in the presence of lithium phenyl-2,4,6-trimethylbenzoylphosphinate (LAP, 0.25%) and a constant ultraviolet (UV) light exposure time of 30 s (Supplementary Fig. 1a, denoted as G30, G60, and G90, respectively), as described in previous reports[33]."

Further, in the following results (page 6), we specify that:

"The stiffness of the GelMA hydrogels was tuned exclusively by varying the degree of methacryloyl (MA) substitution without altering the Arg-Gly-Asp (RGD, a minimal tripeptide motif recognized by integrins and mediates cell adhesion) content; accordingly, the proliferation rate did not differ across the groups. Previous studies similarly reported no significant effect of stiffness on proliferation when the RGD density was unchanged[36]."

2) Optimizing hydrogel preparation to harmonize mechanical testing with culture conditions

We realized in our original setup, mechanical tests were performed on 5 mm thick cylindrical hydrogels crosslinked for 120 s referenced prior work (Chansoria et al., ACS Biomater Sci Eng, 2021)[3], they stated: "For the compression testing, cylindrical GelMA hydrogel constructs (∅ = 7.5 mm, thickness = 5 mm) were fabricated via casting in negative molds made of a flexible resin (Smooth Cast 300, Smooth-On Inc., Macungie, PA)."

Whereas 3D cell culture used ~ 25 µL hemispherical droplet crosslinked for 30s referenced previous publications (Dye et al., Elife, 2015)[4] (Hein et al., Development, 2022)[5] (Miller et al., Nat Protoc, 2019)[6].

Because the thickness and crosslinking time affect crosslink density and thus modulus (see Rebuttal Fig. 1), we harmonized the hydrogel used for mechanical testing with culture conditions by adopting the thickness of 2.5mm (assume 25 µL droplet is a hemisphere of radius about 2.5 mm) and 30 s UV crosslinking for mechanical characterization.

Therefore, we now describe the mechanical-testing hydrogel preparation in the "Supplementary Methods" of the revised "Supplementary Information" (page 22) that:

"For sample preparation, GelMA precursor solutions were injected into PDMS molds (2.5 mm thickness) sandwiched between glass coverslips. To facilitate detachment, coverslips were pretreated with an anti-adherence rinsing solution (STEMCELL, CAT#7010), then rinsed 3 times with DPBS and air-dried at room temperature. GelMA precursor was crosslinked under UV light for 30s. The hydrogel was carefully detached with a spatula, and punched into 10 mm diameter cylinders using a biopsy punch. The hydrogels were equilibrated in DPBS overnight prior to testing."

We have added the rheological and compression testing data to the "Results" section of the revised manuscript (page 5) that:

"Rheological tests (Supplementary Fig. 1b) and compression testing confirmed that the stiffness gradually increased from G30 to G90, yielding compressive moduli of 2.99 ± 0.24, 6.22 ± 0.65, and 11.31 ± 0.12 kPa, respectively (Supplementary Fig. 1c, d)."

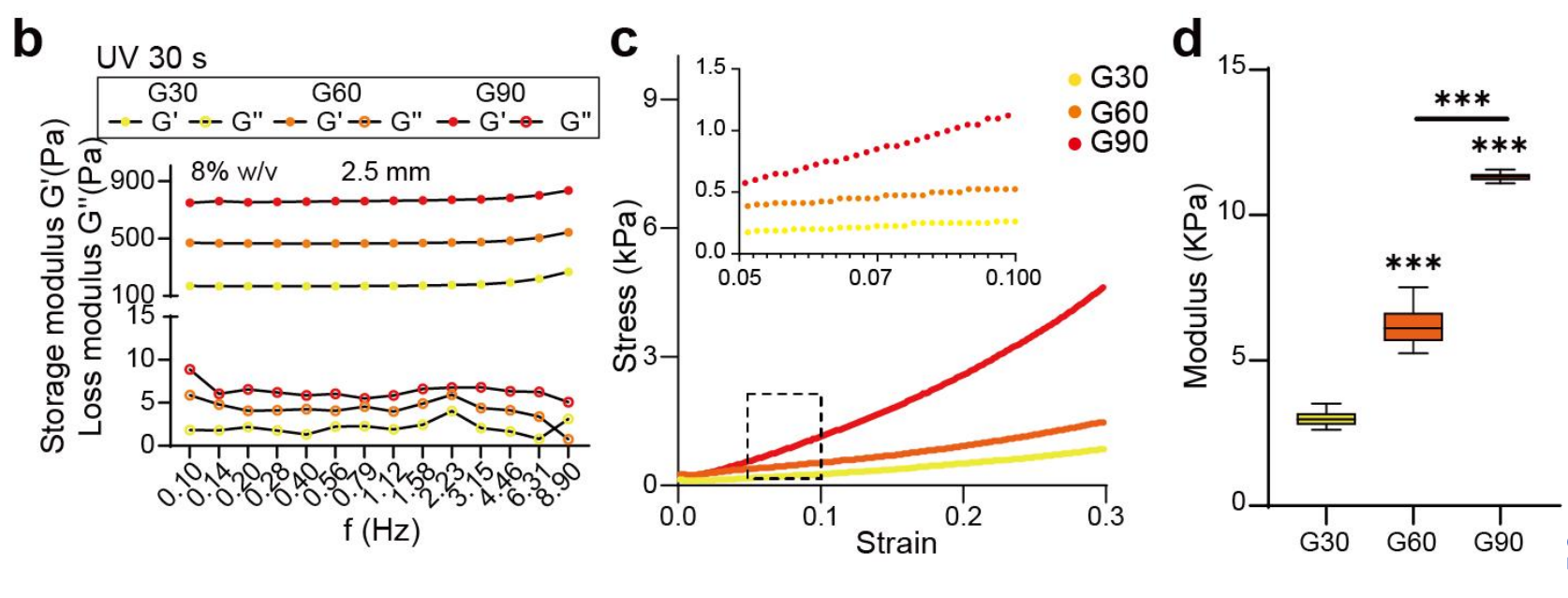

Supplementary Fig. 1b-d

We also performed rheology and unconfined compression tests on hydrogels crosslinked for 30 s, 60 s and 120 s, and with thickness 2.5 or 5 mm thickness (120 s). The results are included in the rebuttal letter for your reference. The hydrogel stiffness increased with both crosslinking time and specimen thickness.

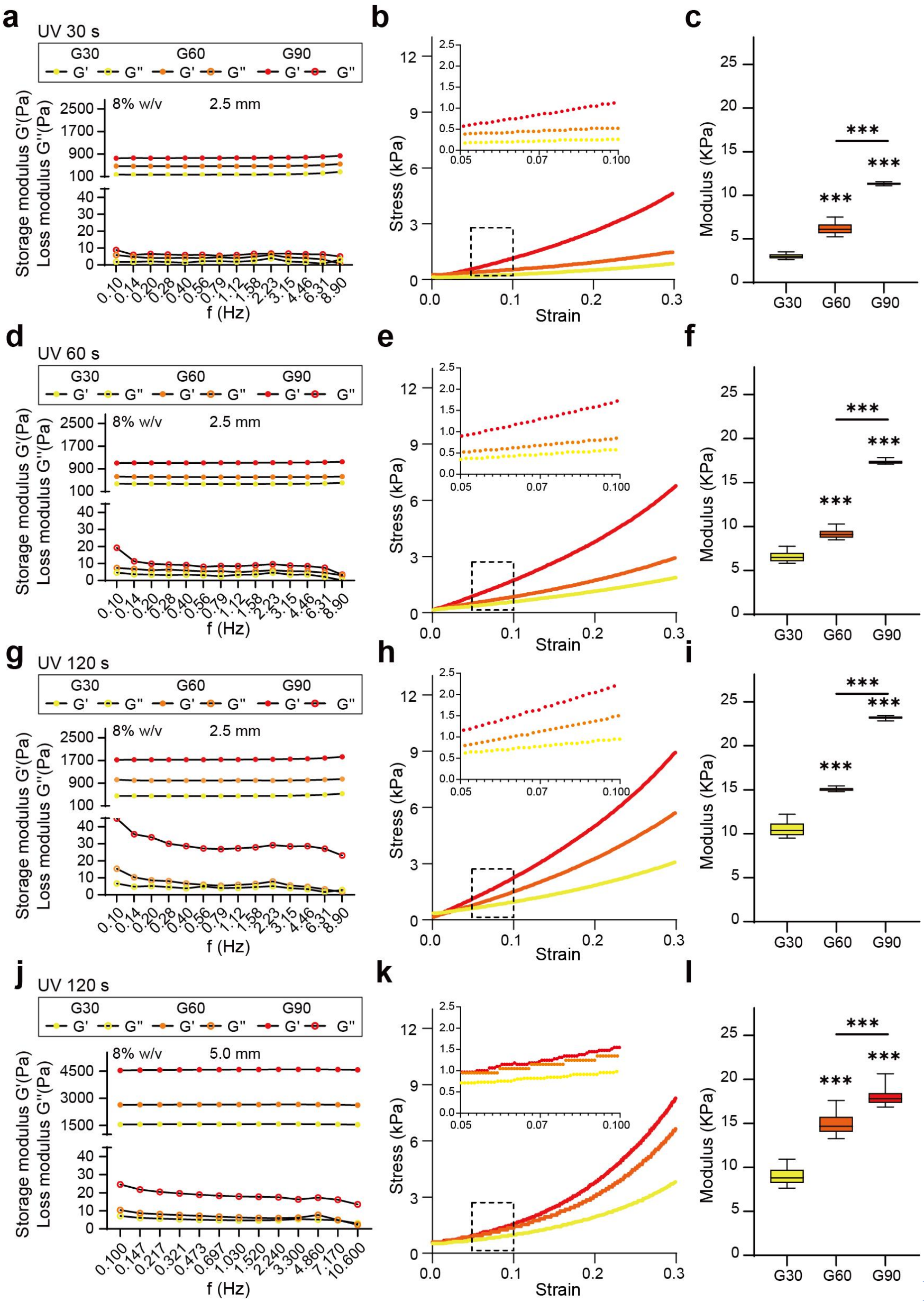

Rebuttal Fig. 1

3.2 Specifically, G90 (~18–20 kPa) approaches the stiffness of fibrotic lung tissue, which may model pathological rather than physiological conditions.

3) Updated modulus after harmonizing hydrogel preparation for mechanical testing

After optimizing hydrogel preparation by reducing the thickness from 5 mm to 2.5 mm and UV crosslinking time from 120s to 30s, the compressive modulus of G90 was 11.31 ± 0.12 kPa (Supplementary Fig. 1c, d), which falls in reported moduli values for healthy lungs (0.5-15 kPa) (Nallet et al., Eur Radiol, 2022)[7] (Guo et al., Compr Physiol, 2022)[8] (Baruffaldi et al., ACS Appl Bio Mater, 2021)[9] although very close to the upper boundary of physiological lung stiffness.

4) Rationale for using high-physiological stiffness

A recent study on human cortical organoids (Cadena et al., Adv Healthc Mater, 2024)[10] reported the optimal scaffold stiffness to promote neural development ranged from 7 to 9 kPa, which lies at

the higher end of the brain ECM physiological range. The authors stated: "the reason of this high stiffness preferential result may due to the developing skull (1 to 50 Mpa), which is orders of magnitude higher than the brain ECM provide force on brain cortical layer."

Similarly, pleura-chest wall coupling imposes cyclic external loads on the lung, the stiffer pleura relative to compliant alveolar septa creates a marked mechanical gradient can transiently elevate the effective mechanical cues sensed by alveolar epithelial during breathing (Luque et al., Acta Biomaterialia, 2013)[11]. Consistently, overly soft matrices impede alveolar lineage progression, whereas appropriate elasticity is required for secondary septation and coordination with respiratory motion (Li et al., Dev Cell, 2018)[12] (Shiraishi et al., Cell, 2023)[13]. Therefore, we now describe, in the "Discussion" section of the revised manuscript (page 15) that:

"Analogously, optimal neural development in human cortical organoids occurs at 7–9 kPa, with the much stiffer developing skull (1–50 MPa) adding a constraining force that promotes morphogenesis[63]. Similarly, a pleura-to-alveolar septa stiffness gradient (from MPa to kPa) could impose extra forces on alveolar epithelia, transiently elevating effective alveolar stiffness[64]. Previous work revealed that soft matrices impede alveolar lineage progression[18], whereas appropriate elasticity is critical for secondary septation and coordination with respiratory motion[65]. Taken together, airway development (conducting function) strictly follows a proximal-to-distal high-to-low stiffness gradient. However, the development and maturation of the alveolar epithelium (air exchange under the respiratory system) are not simply better, and the external force seems to play an essential role in signaling pathways that determine epithelial fates."

5) Transcriptomic IPF gene expression in our G90 hALOs

Bammert *et al*. (Bammert et al., Adv Sci (Weinh), 2025)[14] performed RNA profiling on IPF patient-derived alveolar organoids and identified 25 significantly upregulated genes (EGFLAM, CD163L1, TRIM43, etc.). Using this signature, we conducted a similarity analysis of our ALOs datasets and found no enrichment or systematic upregulation of canonical IPF-related programs under G90. Therefore, we now describe, in the "Results" section of our revised manuscript (page 12) that:

"Using identified 25 significantly upregulated genes (EGFLAM, CD163L1, TRIM43, etc.) in idiopathic pulmonary fibrosis (IPF) patient derived organoids[45], we proved there were no canonical fibrotic programs in G90 (Supplementary Fig. 11g), supporting a non-pathological interpretation. Thus G90 serves as a high-physiological boundary stiffness rather than a model of fibrosis, enabling us to interrogate stiffness-dependent epithelial fate decisions across a physiologically relevant gradient."

g

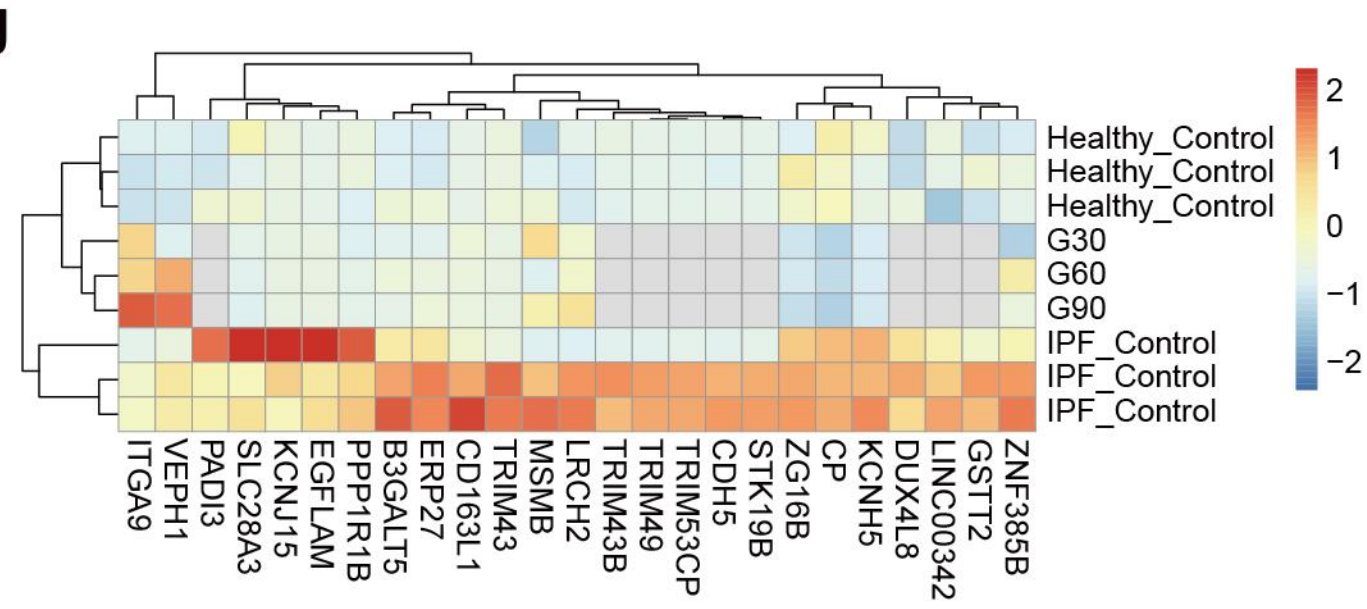

Supplementary Fig. 11g

3.3 Please clarify whether these values reflect in vivo gradients across healthy airways and alveolar regions, especially during development.

6) Evidence of lung stiffness *in vivo*

To the best of our knowledge, region-specific fetal lung stiffness data remain limited due to the scarcity of fetal samples and the challenges in direct measurement. Recent two-dimensional shear wave elastography (2D-SWE) provided fetal lung stiffness reference data, showing that fetal pulmonary elasticity values initially increase and then decrease during second and third trimesters with a range of 3.68-5.0 kPa from 20-39 weeks of gestation (Nallet et al., Eur Radiol, 2022)[7] (Liu et al., Quant Imaging Med Surg, 2024)[15].

In adults, multiple modalities indicate proximal-distal mechanical contrasts, cartilage-supported proximal airways and pleura present higher apparent stiffness, whereas alveolar septa are comparatively compliant, yielding physiological values on the order of 0.5 to 15 kPa across regions.

7) How our *in vitro* values related to these gradients

Guided by this literature and the known anatomical progression from trachea/bronchi to distal bronchioles to alveoli (Bennet et al., Cells, 2021)[16] (Schematic on the right). We sampled a physiologically relevant window using three GelMA formulations that form a gradient of 2.99 ± 0.24, 6.22 ± 0.65, and 11.31 ± 0.12 kPa (Supplementary Fig. 1c, d). These levels: (i) span the fetal/low stiffness range captured by 2D-SWE, and (ii) extend toward the upper boundary of healthy adult tissue, where proximal structures and pleural coupling can increase effective mechanical cues. Therefore, our stiffness set is intended to approximate replicate the *in vivo* proximal to distal mechanical landscape encountered by developing epithelia.

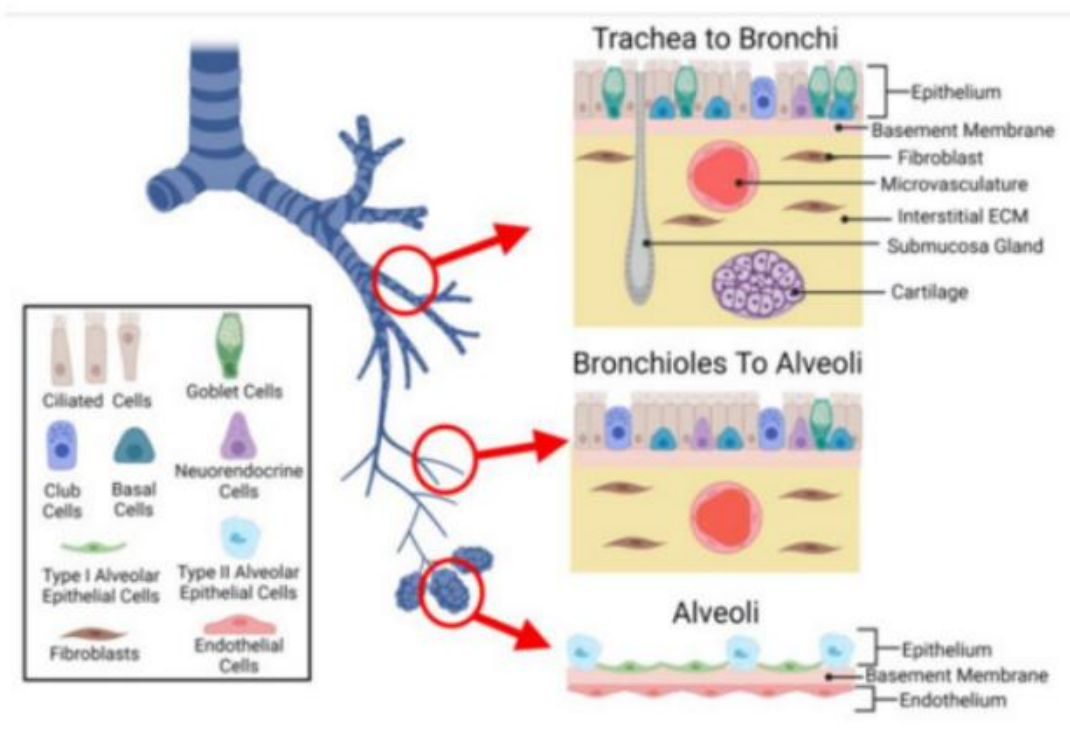

Bennet et al, cells, 2021

In summary of the findings from 6) and 7), we now describe, in the "Discussion" section of the revised manuscript (page 15) that:

"To recapitulate the proximal–distal mechanical gradient of the respiratory system, we employed three GelMA hydrogels (G30, G60 and G90) with stiffness ranging from 2.99 kPa to 11.31 kPa (Supplementary Fig. 1c), covering reported lung regional values (0.5-15 kPa) and fetal lung stiffness (3.68-5 kPa)[25, 61]. "

4. 1)How many organoids were observed across the different GelMA stiffness conditions? 2)Given that G90 lacks the mechanical characteristics (viscoelasticity) typically necessary for supporting cell viability and organoid formation, how do you explain its ability to sustain organoid development in the presented study?

We thank the reviewer for these important questions.

4.1) How many organoids were observed across the different GelMA stiffness condition?

As described in the "Methods" of the original manuscript (page 17): "Approximately 200 AFE spheroids were mixed with 25 µL GelMA precursor and placed at the center of one well of a 24-well plate." "LPCs were released from GelMA hydrogels using 100 µg/mL Collagenase Type II (Sigma-Aldrich, Cat#C2-28-100mg) and re-encapsulated (200 orgnoids) following the same GelMA encapsulation protocol."

Accordingly, ~200 spheroids/organoids per 25 µL droplet per well were used across all stiffness hydrogels (G30, G60 and G90) in each experiment. Representative bright field images for each stiffness are provided in Rebuttal Fig.2 for reviewers' reference.

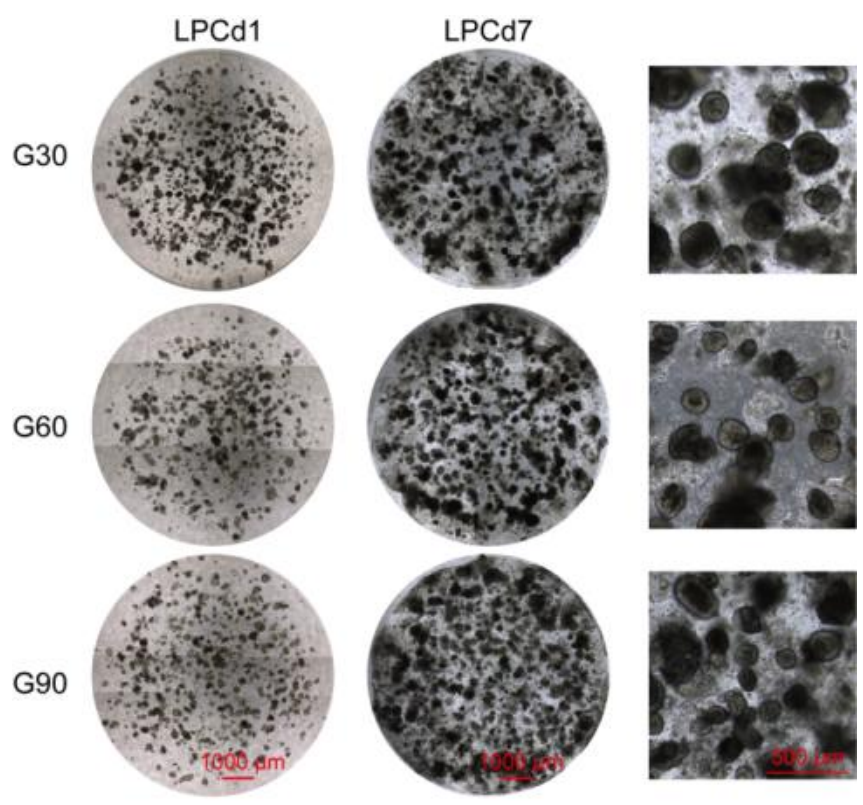

Rebuttal Fig. 2

4.2) Given that G90 lacks the mechanical characteristics (viscoelasticity) typically necessary for supporting cell viability and organoid formation, How do you explain its ability to sustain organoid development in the presented study?

1) As the reviewer noted and as shown in Supplementary Fig. 2b, directly encapsulated single cells in stiffer hydrogels compromised cell viability and prevented organoid formation, whereas self-assembled AFE spheroids remained viable and generated luminal, multicellular structures even in G90 (Supplementary Fig. 2b).

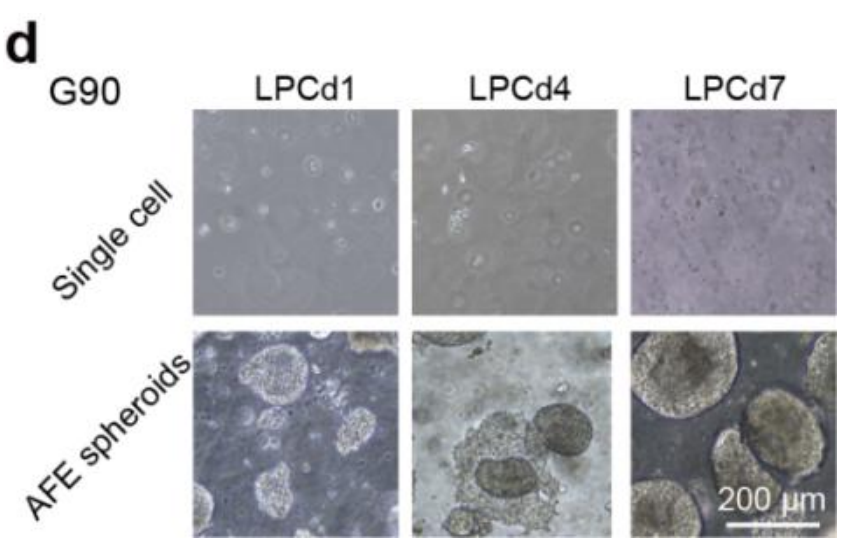

Supplementary Fig. 2b

2) In the current study, we embedded and passaged multicellular aggregates rather than single cells, following established protocols (Dye et al., Elife, 2015)[4] (Hein et al., Development, 2022)[5] (Miller et al., Nat Protoc, 2019)[6]. As stated in the "Discussion" and "Methods" sections of the original manuscript: (Lines 431-432) " ....we adapted Spence lab's method by encapsulating self-assembled AFE spheroids in stiffness-tunable hydrogels to differentiate LPC."(Lines 647-649) "5.4.3 Encapsulation of AFE spheroids in GelMA hydrogel and differentiation into LPC: AFE spheroids were encapsulated into GelMA hydrogel to support the 3D culture as described previously."(Lines 657-660) " ...LPCs were released from GelMA hydrogels using 100 µg/mL Collagenase Type II (Sigma-Aldrich, Cat#C2-28-100mg) and re-encapsulated following the same GelMA encapsulation protocol."

3) Consistent with our observations, Liu *et al.* (Liu et al., Adv Sci (Weinh), 2025)[17] stated that: "Single cells encapsulated directly into high-stiffness HA-ELP hydrogels (~6kPa) could not survive or form organoids. However, in the dynamic stiffening hydrogel that was initially soft (~1.5kpa) to allow single-cell assembly into spheroid, the subsequent increase in stiffness to 6 kPa did not impair the cell viability. Instead, it promoted further cell fate transdifferentiation under fibrotic mimic ECM stiffness."

Taken above together, AFE spheroids and LPC organoids with multicellular structure can survive and develop well in higher stiffness hydrogel. We now describe, in the "Results" section of the revised manuscript that (page 6):

"Self-assembled AFE spheroids remained viable and generated luminal, multicellular organoids across all stiffness hydrogels (Supplementary Fig. 2b, c). However, direct encapsulation of single cells in G90 did not support survival or organoid formation (Supplementary Fig. 2d). In contrast, in G90, self-assembled AFE spheroids grew with time and progressively formed multicellular structures (Supplementary Fig. 2d). Consistent with prior work[35], once multicellular aggregates are established under permissive conditions, subsequent culture in stiffer matrices can modulate cell fate without compromising survival."

5. Many previous studies have shown that hydrogel stiffness often influences the cell proliferation rate, however, your results showed differently. Could you clarify this and provide a possible explanation for why your findings differ from earlier reports?

We appreciate the reviewer's question.

1) We analyzed bulk- and sc-RNA seq data of LPC, hAWOs and hALO, which consistently indicates no difference in proliferation across G30, G60 and G90. We have added these depictions in the "Results" section of the revised manuscript that:

(page 7; LPC) "Cell cycle and replication genes were comparable across G30, G60, and G90 (Supplementary Fig. 3i), indicating minimal effects of varying stiffness on cell proliferation status."

i

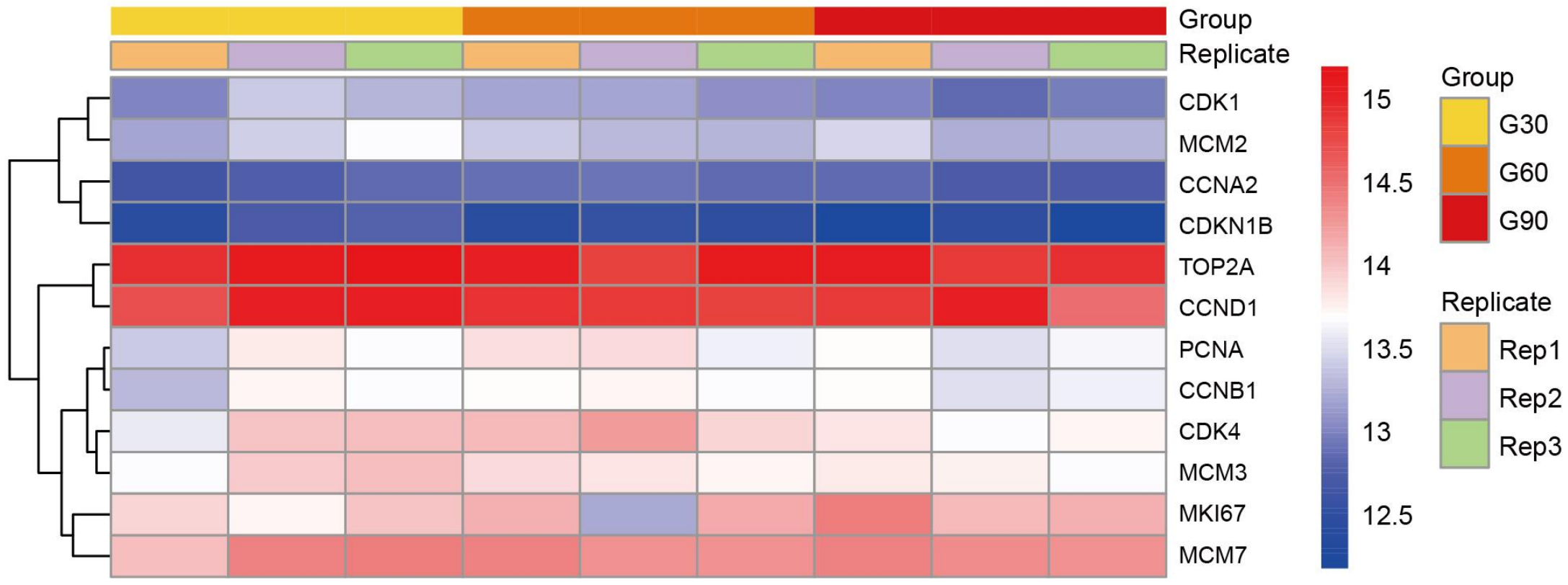

Supplementary Fig. 3i

(page 9; hAWOs) "Consistent with a differentiation effect rather than a proliferation effect, no significant differences in the S Score, G2M Score, *MKI67*, or *TOP2A* were noted across G30, G60 and G90 (Supplementary Fig. 7j)."

j

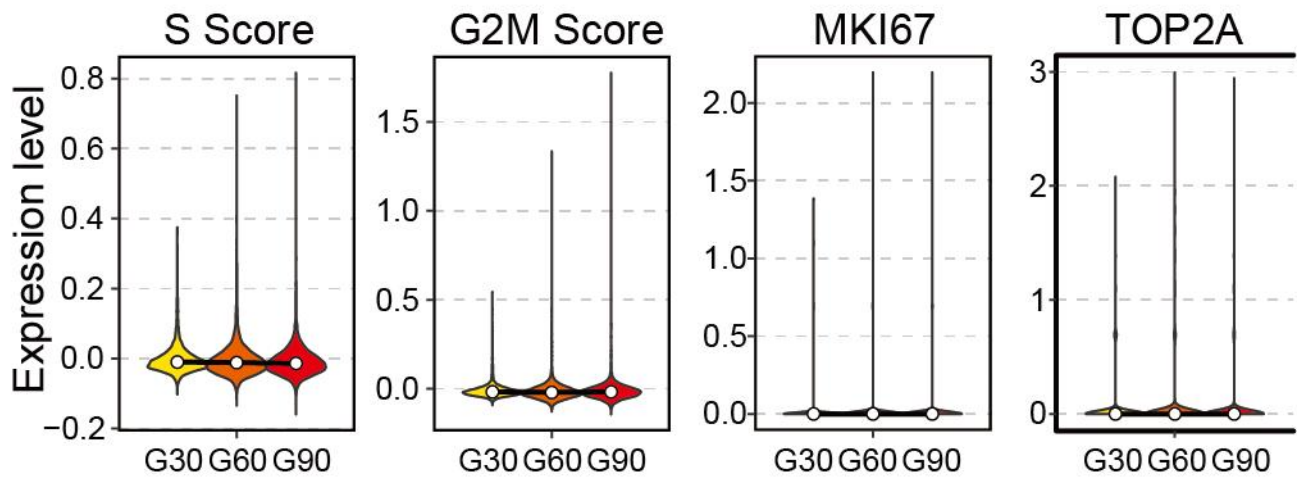

Supplementary Fig. 7j

(page 12, hALOs) "Cell cycle and replication genes (*MKI67*, *TOP2A*, *MCM2*, and *RFC4*) were comparable across G30, G60, and G90 (Supplementary Fig. 11f), indicating minimal effects of varying stiffness on cell proliferation status."

f

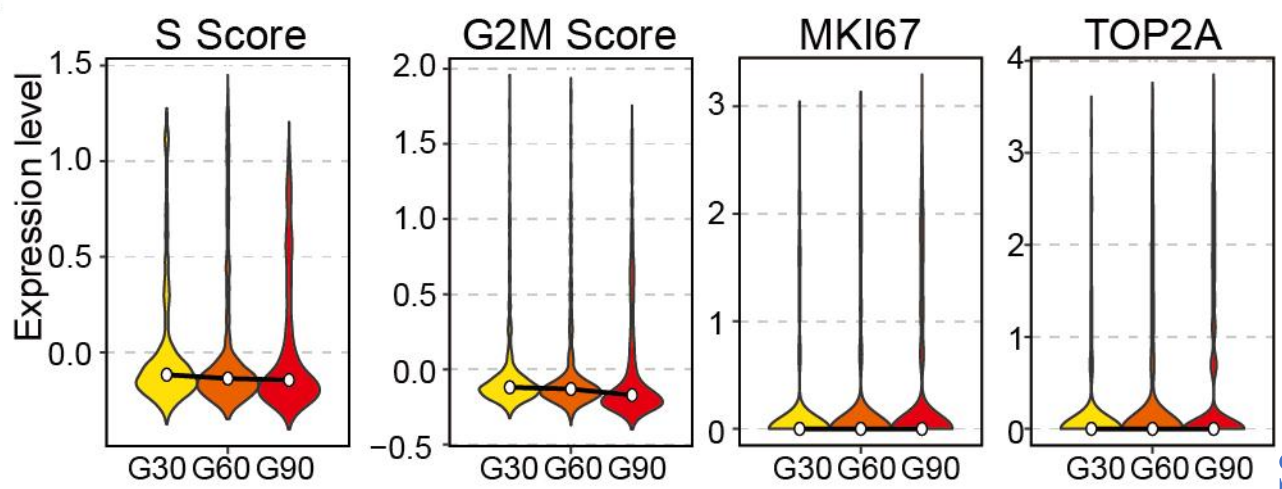

Supplementary Fig. 11f

2) While many studies report stiffness-dependent changes in cell proliferation (He et al., Gastroenterology, 2023)[18] (Cruz-Acuna et al., J Clin Invest, 2023)[19] (Nguyen et al., J Cell Sci, 2024)[20], others have shown no effect when adhesive ligand density (e.g. RGD concentration) is rigorously controlled (Brock et al., Cells, 2022)[21] (Blackford et al., Biomaterials, 2023)[22] (Conway et al., Proc Natl Acad Sci U S A, 2023)[23] (Indana et al., Adv Mater, 2021)[24] (Li et al., Polymers, 2016)[2]. For example, Indana *et al.* (Indana et al., Adv Mater, 2021)[24] stated that: "...these data show that hydrogel stiffness does not impact hiPSC viability, apoptosis, proliferation, pluripotency, and lumen formation. Higher RGD density and fast stress relaxation promote hiPSC viability, proliferation, and lumen formation, while slow stress relaxation at low RGD densities triggers hiPSC apoptosis" Li *et al.* (Li et al., Polymers, 2016)[21] stated that: "...prepared GelMA hydrogels with stiffness ranging from ~4 kPa to ~30 kPa while maintaining constant RGD density.....To

investigate whether the stiffness affects cell proliferation....The cells cultured in different GelMA hydrogels showed almost the same DNA amount, indicating the stiffness of hydrogels had no obvious influence on cell proliferation." Taken together, the cell proliferation rate directly corresponded to the RGD density.

Consistent with this design, our hydrogel preparation strategy maintains constant RGD density while tuning stiffness by varying the degree of MA substitution. We now describe, in the "Results" section of the revised manuscript (page 6) that:

" LPC growth, proliferation, and apoptosis were not affected by increased stiffness (Supplementary Fig. 3a-h). The stiffness of the GelMA hydrogels was tuned exclusively by varying the degree of methacryloyl (MA) substitution without altering the Arg-Gly-Asp (RGD, a minimal tripeptide motif recognized by integrins and mediates cell adhesion) content; accordingly, the proliferation rate did not differ across the groups. Previous studies similarly reported no significant effect of stiffness on proliferation when the RGD density was unchanged[36]."

6. It is surprising that distal alveolar cells didn't favor soft ECM conditions, as this contradicts typical in vivo lung biology and is inconsistent with the airway data presented. Could you clarify the physiological relevance of this finding and what it suggests about the role of ECM stiffness in regulating alveolar cell fate?

We appreciate the reviewer's questions.

1） Actually, the distal alveolar cells reside within a soft yet highly elastic ECM and feel the gradient pressure transmitted from surrounded tissue (e.g. pleura). Thus, the effective mechanical cues sensed by alveolar cells reflect not only matrix softness but also external forces.

2） In our system, intermediate-to-high stiffness (~6-11 kPa) favored alveolar maturation and AT2 to AT1 transition. We interpret this not as a contradiction of *in vivo* softness, but as evidence that appropriate elasticity together with loading, optimizes alveolar fate programs. In the absence of true respiratory motion in our *in vitro* system, a stiffer matrix can approximate the composite mechanical signal that alveoli experience *in vivo*. By contrast, airway development, which serves a conducting function and includes cartilage reinforced segments, follows a clearer proximal-to-distal decline in apparent stiffness.

To more clearly address our depiction of extracellular mechanical forces in alveolar development and maturation, emphasize the alveolar epithelial cells reside in a soft but highly elastic microenvironment, we now describe, in the "Introduction" section of the revised manuscript (page 4) that:

"The ECM surrounded alveolar epithelia is both soft and highly elastic[15]. Alveolar epithelial development begins at the late canalicular stage (~24 weeks of gestation) and continues until early childhood (~8 years of age)[16]. During the saccular and alveolar stages, myofibroblasts deposit fibronectin, elastin and collagen IV forming a compliant but highly elastic ECM that supports epithelial morphogenesis[17]. After birth, biomechanical forces generated by respiratory motion are crucial for maintaining AT1 cell identity, whereas the loss of biophysical forces can drive AT1

reprogramming toward AT2[18]. An appropriate level of ECM elasticity is also critical for secondary septation (the formation of mature alveoli)[19]. In summary, the alveolar ECM is soft and elastic, and the proper mechanical force is critical for mature alveolar formation and cooperative respiratory movement."

In the Discussion section of the original manuscript (page 15), we have discussed: "Surprisingly, distal alveolar cells did not favor soft hydrogel as we proposed. Instead, intermediate-to-high stiffness (6.22-11.31 kPa) enhanced alveolar cells maturation and AT2-to-AT1 transition (Fig. 4). In fetal and neonatal lungs, alveoli continue to mature postnatally under respiratory forces that promote alveolar expansion and differentiation."

To underscore the importance of stiffness in alveolar development and distinct between airway and alveolar ECM, we have added further citations to prior studies in the revised manuscript (page 15) that:

"Previous work revealed that soft matrices impede alveolar lineage progression[18], whereas appropriate elasticity is critical for secondary septation and coordination with respiratory motion[65]. Taken together, airway development (conducting function) strictly follows a proximal-to-distal high-to-low stiffness gradient. However, the development and maturation of the alveolar epithelium (air exchange under the respiratory system) are not simply better, and the external force seems to play an essential role in signaling pathways that determine epithelial fates."

7. Experimental Details:

Please provide more detailed methodological information, particularly regarding:

We appreciated the reviewer's comments regarding more detailed methodological information.

o Time point of live/dead staining

Live/dead staining was performed on day 7 LPCs. We have added the time point and detailed protocol in the "Supplementary Methods" of the revised Supplementary Information (page 25) that:

"Live/Dead staining was performed on day 7 LPCs following previous published protocol[8] with minor modification. Live cells were labeled with Calcein-AM and dead cells with PI. Briefly, LPCs were released from GelMA using collagenase Type II, then incubated with Calcein-AM (2 μM) and PI (4 μM) premixed in DPBS at 37 °C for 30 min, protected from light. After staining, samples were washed three times with DPBS, transferred to glass bottom cell culture dish (NST, Cat#801002) and imaged using an inverted fluorescence microscope (Carl Zeiss LSM 980)."

o Number of images captured per sample, how the images to be analyzed were chosen?

We acquired 9 non-overlapping fields of view per sample. The imaging area was partitioned into a 3x3 grid (top-left, top-center, top-right; center-left, center, center-right; bottom-left, bottom center, bottom-right). Images to be analyzed were selected randomly from non-overlapping regions to avoid sampling bias. We now describe, in the "Methods" of the revised manuscript (page 21) that:

"For each sample, 9 representative fields of view (top-left, top-center, top-right; center-left, center,

center-right; bottom-left, bottom center, bottom-right) were captured using a Carl Zeiss LSM 980 confocal microscope. Images to be analyzed were selected randomly to minimize sampling bias."

o Number of cells/images used for each quantification

At least 500 cells or 4-5 randomly selected images were analyzed per condition.

We have added this detail to the "Methods" of the revised manuscript (page 22) as follows:

"Approximately 500 cells or 4-5 randomly selected images were analyzed per sample."

o Number of biological replicates that were included per group for the sequencing analysis?

1) For the bulk RNA sequencing, we included 3 biological replicates per group. We have added this information after the description of G30, G60 and G90 sample collection in the "Methods" section of the revised manuscript (page 19) that:

"(3 biological replicates per group)"

2) For the scRNA-seq, we included one biological replicate per stiffness condition. We have added the following details to the "Methods" of the revised manuscript (page 19) that:

"For scRNA-seq, one biological replicate was included for each stiffness sample (G30, G60, G90). Pre-sequencing qPCR and IF were used to verify marker-expression trends."

o How many reads/cell were aimed for the sequencing?

We aimed for a sequencing depth of approximately 50,000–100,000 reads per cell, following standard practice. In the generated datasets, the mean reads per cell were 77,778 reads for hAWOs and 407,789 reads for hALOs. The detailed information about quality control of sequencing data has been depicted in the "Supplementary Methods" of the original Supplementary Information (page 28) that:"Reads were mapped to the GRCh38 genome for hAWOs (42,665 cells; 77,778 reads) and GRCh38 genome for hALOs (8,132 cells; 407,789 reads). Low-quality cells were excluded based on a cutoff of fewer than 500 genes or 1000 UMIs."

o In Figure 1h, could you clarify which group comparisons were used to generate the enriched pathways shown?

Pathway enrichment in Fig. 1h was performed on upregulated gene sets from the following pairwise contrasts: G60 vs G30, G90 vs G60, and G90 vs G30. The gene lists used for each contrast are provided in Supplementary Data 1. We now describe, in the "Results" section of the revised manuscript (page 7) that:

"Pathway enrichment in upregulated genes from G60 vs. G30, G90 vs. G60, and G90 vs. G30 (Supplementary Data 1) ...... (Figure 1h)."

8. 1)The identification of neuronal populations within the organoid system raises some concern. 2)If the cells were sorted to be EpCAM+ prior to sequencing then how were mesenchymal and

neuronal cells captured in the sequencing population? 3)How pure was the cell sorting? 4)What neuronal markers were used to support this annotation? Additional validation or clarification is necessary.

We appreciate the reviewer for these important questions.

8.1 The identification of neuronal populations within the organoid system raises some concern.

We apologize for mistakenly annotated pulmonary neuroendocrine cells (PNECs) as neuron in our original manuscript. They should be annotated PNECs rather than "neuron" because all the neuronal clusters were positively co-expressed EpCAM marker (Fig. 3c, Fig.5b).

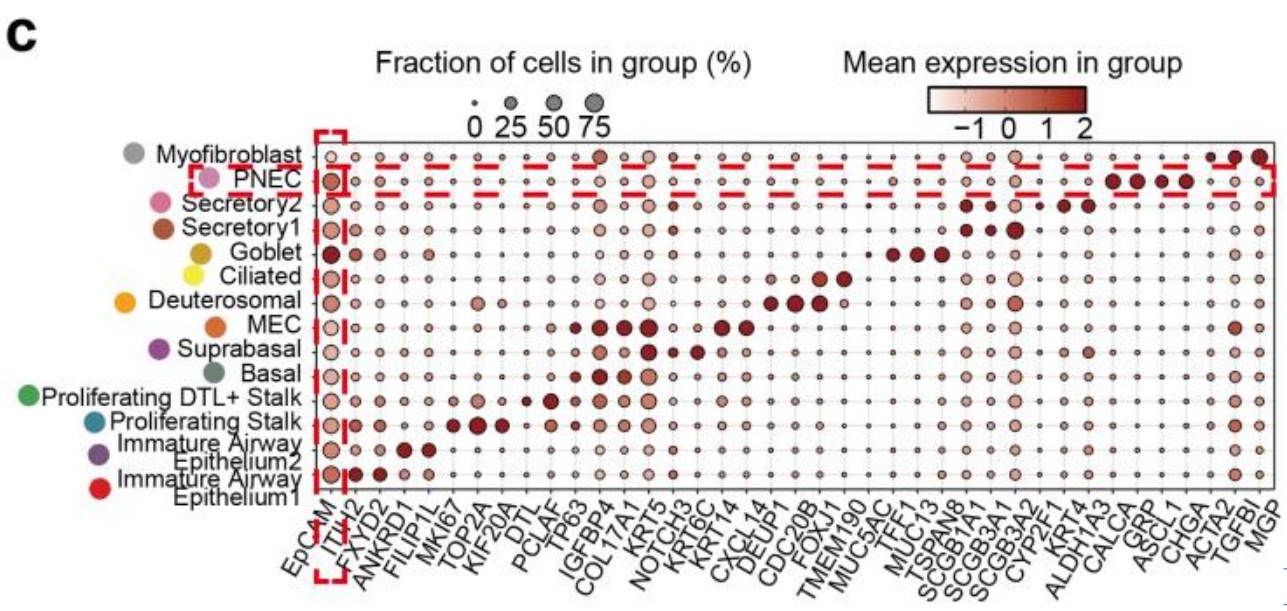

Fig. 3c

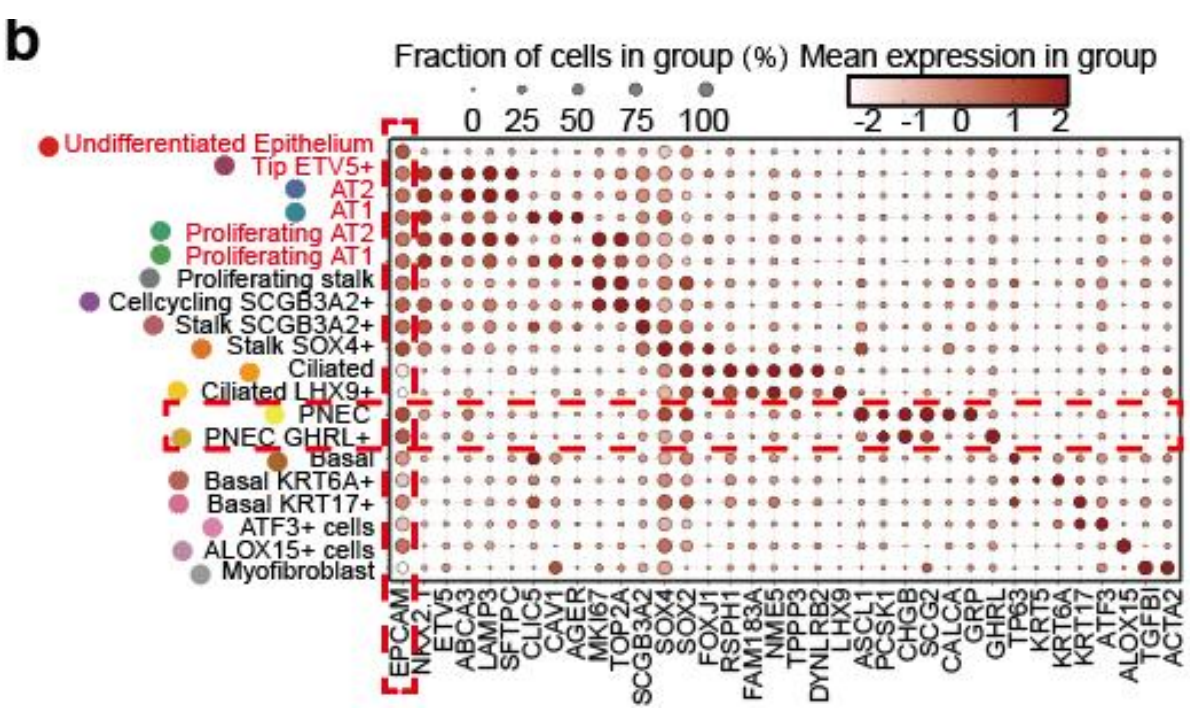

Fig. 5b

We have corrected the identity of the neural cluster to epithelial cluster in the broad cell type annotation accordingly in the revised figures and manuscript text (Supplementary Figs. 6a-b, 10a-b). The terms "neuron" and "neuroendocrine" have been replaced with PNECs (Figs. 3c, 5b).

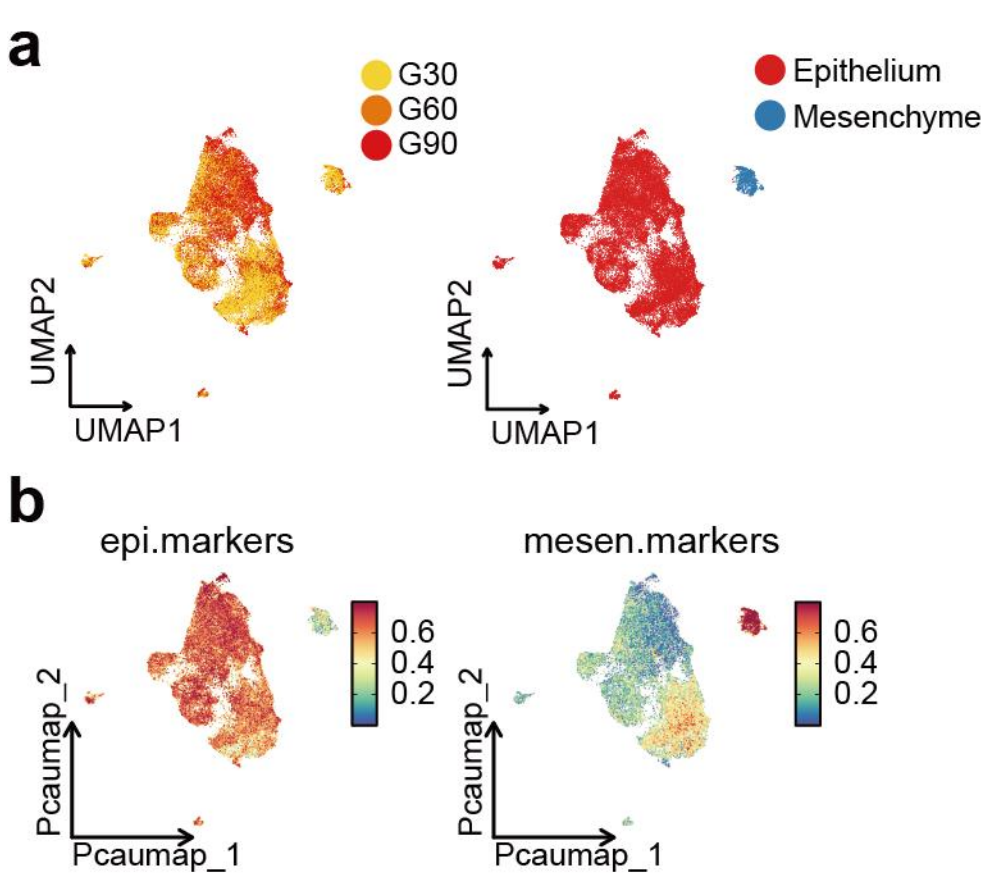

Supplementary Fig. 6a-b

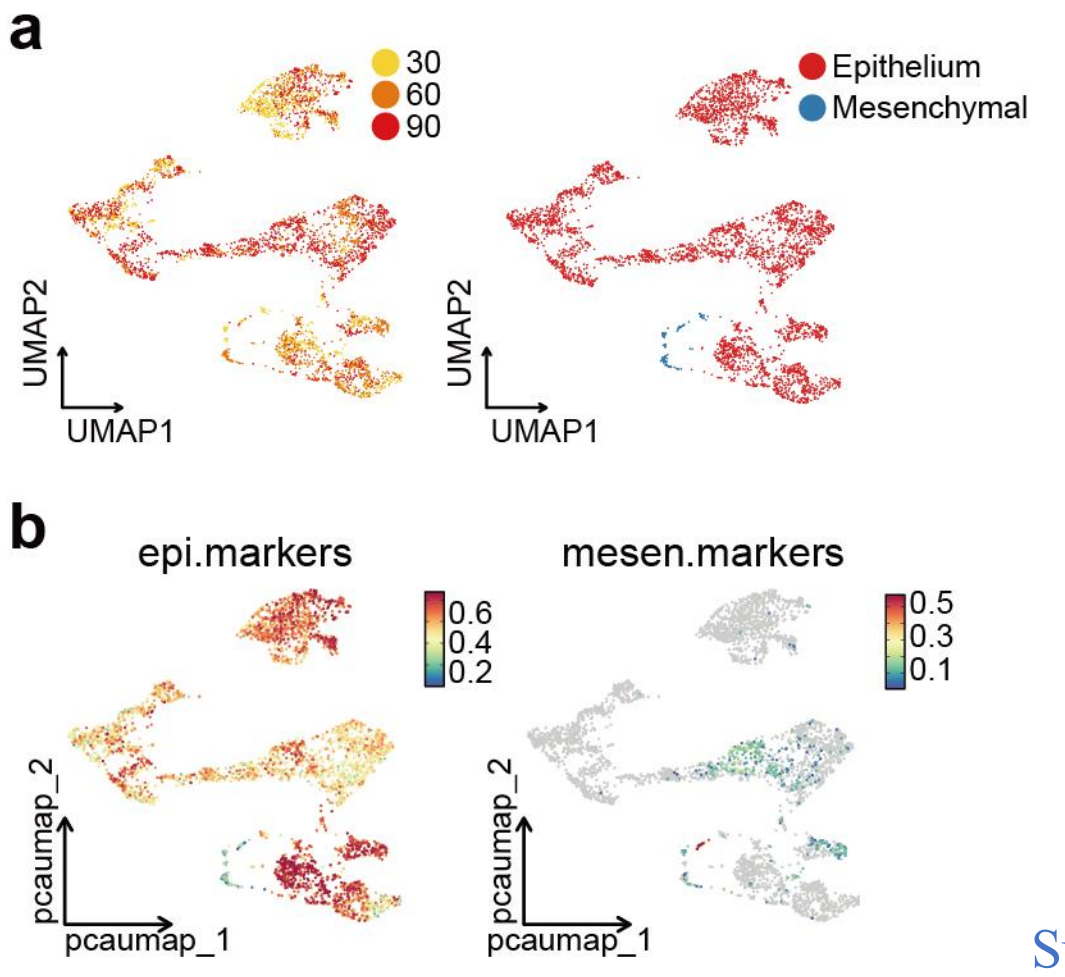

Supplementary Fig. 10a-b

8.2 If the cells were sorted to be EpCAM+ prior to sequencing then how were mesenchymal and neuronal cells captured in the sequencing population?

We have responded for the question 8.1 that all cells initially labeled "neuronal" or "neuron" were EpCAM+ PNECs rather than neurons. Although we prospectively enriched EpCAM+ cells prior to scRNA-seq, it is inevitable mixed a small amount of other lineages because the organoids are differentiated from pluripotent stem cells. Previous studies of hiPSC-HLO have reported residual mesenchymal clusters in scRNA-seq datasets (Miller et al., Nat Protoc, 2019)[6] (Budeus et al., Stem Cell Research & Therapy, 2024)[25]. Moreover, mesenchymal-epithelial interactions are indispensable during fetal lung development, providing a developmental rationale for detecting minor mesenchymal populations (Hawkins et al., J Clin Invest, 2017)[26] (Tamai et al., Cell Rep Methods, 2022)[27]. We now describe, in the "Results" of the revised manuscript that:

(page 8; hAWO): "The cells were categorized into epithelial and mesenchymal lineages (Supplementary Fig. 6a, b), with 96.2% epithelial cells. A small mesenchymal admixture (3.8%) remained, likely due to mesenchymal–epithelial interactions during lung development. Previous studies with hiPSC-HLOs have also detected residual mesenchyme from scRNA-seq datasets[23, 40]."

(page10; hALO): "Among the 4,030 cells profiled, 97.2% epithelial and 2.8% mesenchymal lineages were further annotated into undifferentiated epithelium, tip ETV5$^+$, AT2, AT1, and proliferating AT2 and AT1 cells (Fig. 5a, b; Supplementary Fig. 10a-d)."

8.3 How pure was the cell sorting?

We have depicted the details of FACS of EpCAM$^+$ sorting in "Supplementary Methods" of the original supplementary Information (page 17 and 19). Gates were defined based on the blank control processed without anti-EpCAM staining. Despite efforts to enrich EpCAM$^+$ cells, a small fraction of other lineage persisted. As quantified above (8.2), mesenchymal cells accounted for 3.8% (hAWOs) and 2.8% (hALOs), which is negligible compared to the epithelial majority. We now add the FACS results to our supplementary figures and describe in the "Results" section of the revised manuscript that:

(page 8; hAWOs): "To enrich for epithelium, epithelial cell adhesion molecule positive (EpCAM+) cells were prospectively sorted prior to sequencing (Supplementary Fig. 5)."

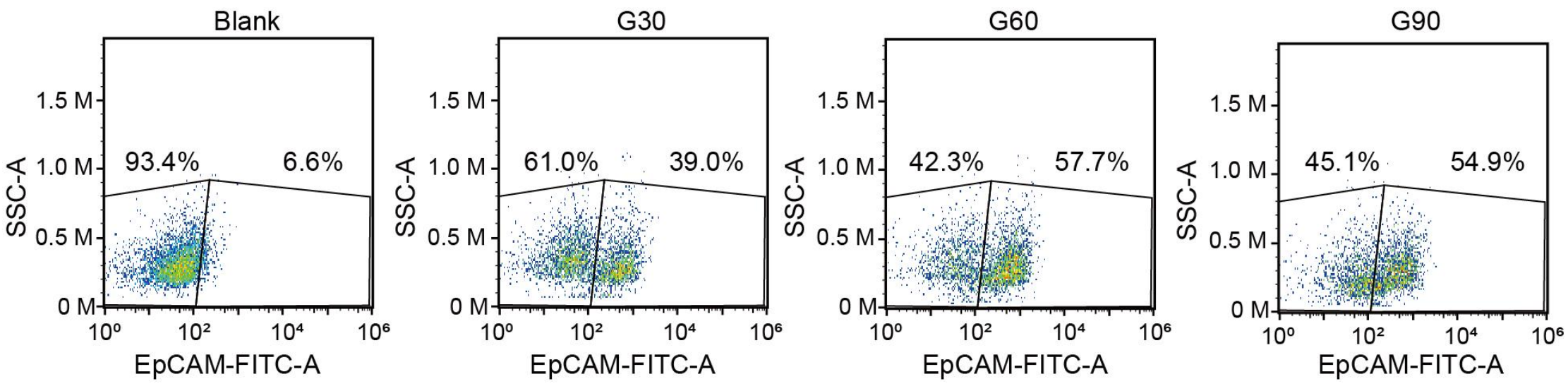

Supplementary Fig. 5

(page 10; hALOs): "EpCAM+ cells were prospectively enriched prior to sequencing (Supplementary Fig. 9)."

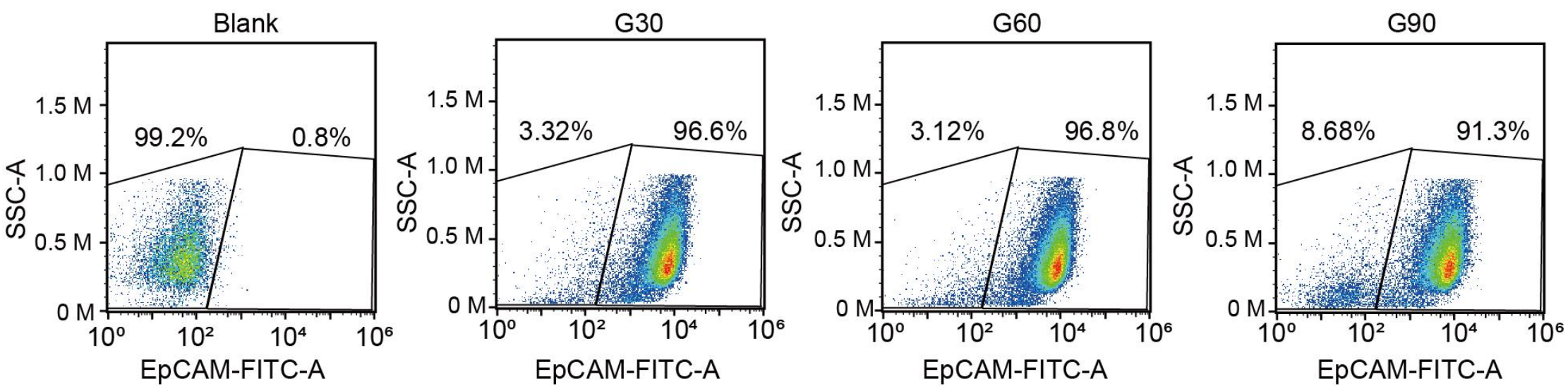

Supplementary Fig. 9

8.4 What neuronal markers were used to support neuron annotation?

The neuronal markers used to support neuron annotation included CALCA, GRP, ASCL1, CHGA, and EPCAM via "AddModuleScore". The gene lists were compiled from the two cited references (He et al., Cell, 2022)[28] (Basil et al., Nature, 2022)[29] and are provided in Supplementary Tables 3 and 4 referenced these papers.

9. Beyond YAP1 localization by staining, were any reporter assays or phosphorylation markers used to confirm Hippo pathway activation?

We appreciate the reviewer's suggestion. To assess Hippo pathway activation beyond YAP1 immunolocalization, we performed western blotting for phosphorylated YAP at serine 127 (p-YAP S127) to confirm Hippo pathway activation statues.

Phosphorylation of YAP by LATS kinases is a canonical readout of Hippo pathway activity, phosphorylation promotes YAP cytoplasmic retention, whereas dephosphorylation favors YAP nuclear translocation (Nguyen et al., Cell Rep, 2014)[30] (Chen et al., Kidney Int, 2024)[31]. To assess this, we performed Western blotting with both anti-YAP and anti-p-YAP S127 antibodies (Cell Signaling Technology, Cat#4911). Our results indicated the total YAP protein levels did not increase with stiffness, whereas p-YAP S127 decreased dramatically (Fig. 5j, k), consistent with enhanced nuclear YAP at higher stiffness (Fig. 5h). We now describe, in our "Results" section of the revised manuscript (page 11) that:

"WB revealed that total YAP protein levels remained relatively constant throughout the stiffness of the hydrogel, whereas phosphorylation at YAP Ser127 (p-YAP S127), a readout of Hippo activity and cytoplasmic retention of YAP[44], decreased as stiffness increased (Fig. 5i, j). These findings are consistent with the enhanced nuclear localization of YAP at G90 (Fig. 5h). Together, these findings

suggest that ECM stiffness modulates Hippo–YAP signaling primarily through posttranslational control of pYAP S127 to regulate YAP subcellular localization in hALOs rather than through changes in total YAP abundance.”

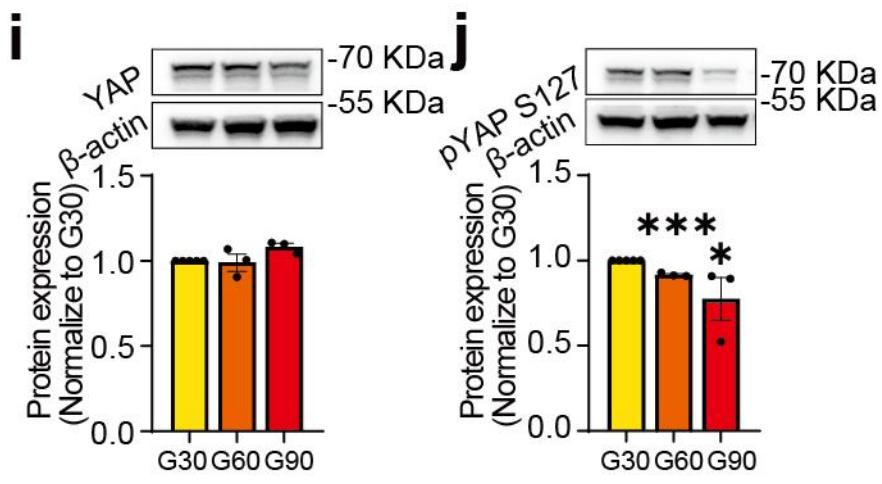

Fig. 5i, j

10. In line 149, the statement of "stiffness-driven mechanisms" requires a stronger link to the data. Could you elaborate on how this conclusion can be supported?

We appreciate the reviewer for this important comment. In the revised manuscript, we have clarified the mechanistic basis underlying the statement of “stiffness-driven mechanisms” specifically in the context of Figure 1 and the lung progenitor cell stage. We now describe, in the “Results” section of the revised manuscript (page 7) that:

“The functional activation of TGF-β with TGFβ1 and nuclear Yes-associated protein (YAP) with TRULI in soft hydrogels (G30) increased NKX2-1 expression (Fig 1i, j), recapitulating the high-stiffness phenotype. These functional rescue experiments demonstrated that the stiffness-driven increase in NKX2-1+ LPCs is mediated, at least in part, by TGF-β and Hippo signaling. Taken together, these data support a model in which increased ECM stiffness promotes LPC specification through modulation of the TGF-β and Hippo pathways, effects that are reproducible by pharmacological activation under low-stiffness conditions.”

11. 1)More information is needed about your data integration steps in the single-cell RNA-seq analysis, as all subsequent downstream analyses depend on this. 2)It is not clear what the authors refer to as a "Biological relevant feature" or a "noisy cluster". The authors should be more concrete about this.

We sincerely appreciate the reviewer’s suggestion, which helped us clarify our single-cell integration procedures.

1) We have substantially revised the relevant “Supplementary Methods” sections in the revised Supplementary Information (page 29) to provide a clearer and more detailed explanation of our integration strategy as below:

“In detail, we applied method = "glmGamPoi" to improve modeling of gene expression variability. All samples were integrated simultaneously, allowing Seurat to internally identify shared features and compute a unified model based on Pearson residuals.”

2) We apologized for the mis-described terms "biological relevant feature" and "noisy cluster" in our original manuscript. In fact, the term "biological-related features" refers to the marker genes used for cluster annotation, that is, the classic markers of known cell types. The term "noise clustering" refers to clustering closely related to technical factors such as sequencing depth, library complexity or high mitochondrial gene expression. To avoid confusions, we have removed these

informal terms from the revised manuscript and instead provided more precise descriptions of the quality control metrics and filtering steps used to identify and exclude such technical artifacts. We have modified the relevant section in the revised Supplementary Information (page 29) that:

"To avoid the potential influence of low quality cells and technical noise,"....... "canonical features"..... "clusters who have very low nUMIs/nGenes, or very high score of mitochondrial genes ....".

12. Further, I would be very hesitant to annotate neurons in an organoid culture, and also did not find any evidence presented by the authors that this could be the case. It is not clear is being defined as neuronal markers.

We sincerely thank the reviewer for helping us improve the biological accuracy of our annotations We mistakenly annotated the PNEC as neuron. We have answered the question regarding the neuron/PNEC annotation and provided neuronal markers in the response to question 8.1 and 8.4. Please find more details in question 8.1 and 8.4.

13. The authors state they use gene lists for annotation, but do not specify which gene lists were used for annotation. The lists used need to be provided for the reader to be able to understand the annotation and whether this makes sense.

We appreciate the reviewer's suggestion. We now compile and provide the full list of marker genes used for cell type annotation across all clusters (Supplementary Tables 3, 4), which includes the gene markers for each cell type identified in our analysis, with supporting references (He et al., Cell, 2022)[28] (Basil et al., Nature, 2022)[29].

We have added to the "Methods" section in the revised manuscript that:

(page 20) "The gene markers genes for each cell type were provided in Supplementary Table 3." "The gene markers used for annotation hALOs were provided in Supplementary Table 4."

The supporting references could be found in the "Supplementary Methods" (page 29) and "Supplementary References" (page 31) of revised Supplementary Information.

14. A supplemental figure with umaps colored by cell-specific markers would help here, but maybe also a dot plot with the authors markers per cell type would be really helpful for the reader to understand how the authors have processed these data.

We appreciate the reviewer's helpful suggestion. We prepared the UMAPs of the markers for all cell types in Supplementary Fig. 6d (hAWOs) and Supplementary Fig. 10d (hALOs). In addition, the dot plot panels are shown in Fig. 3c and Fig. 5b (see the figures attached in response to Comment 8.1). Together, these UMAPs and dot plots present the detailed cell type-specific markers used for cell annotation.

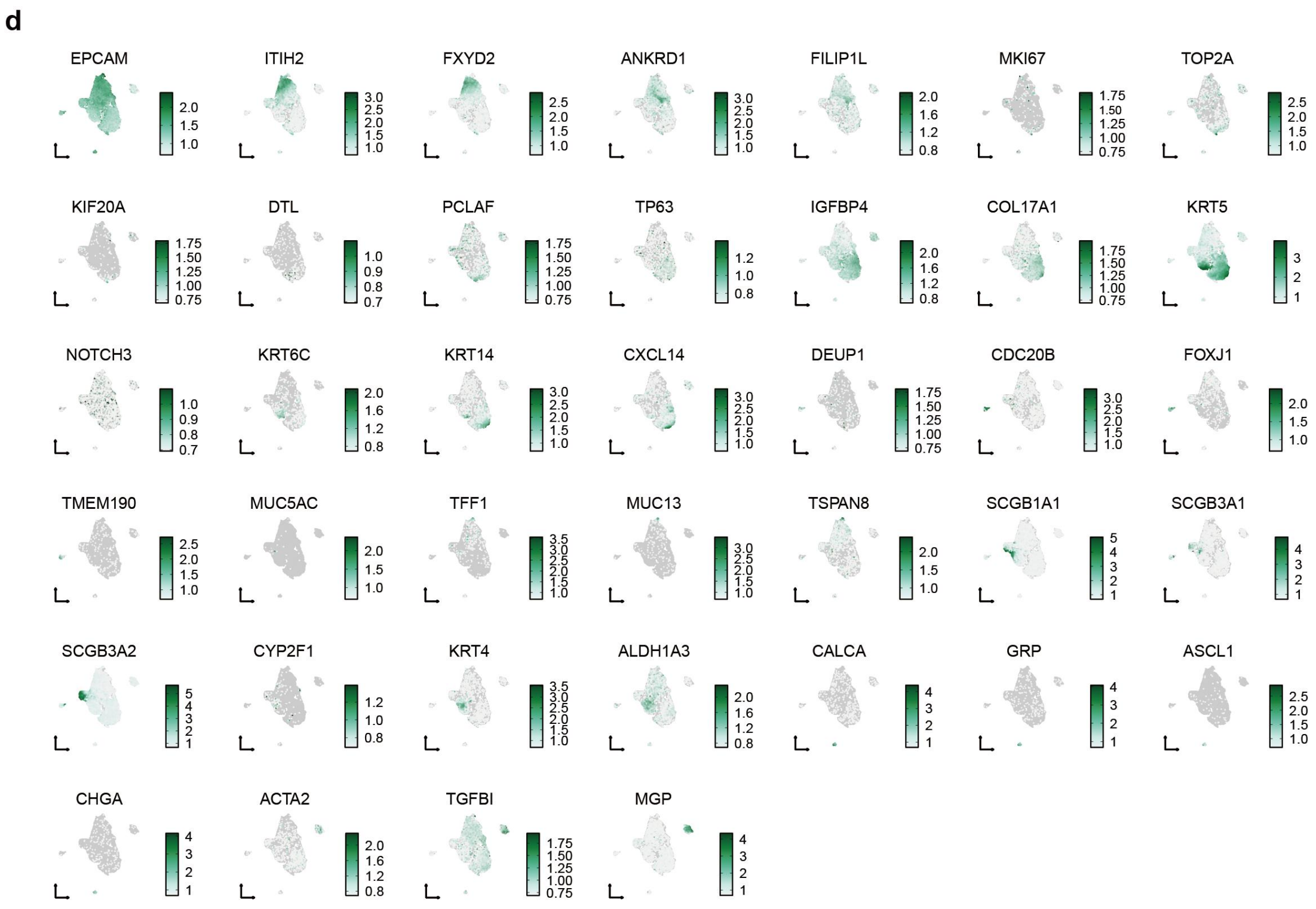

Supplementary Fig. 6d

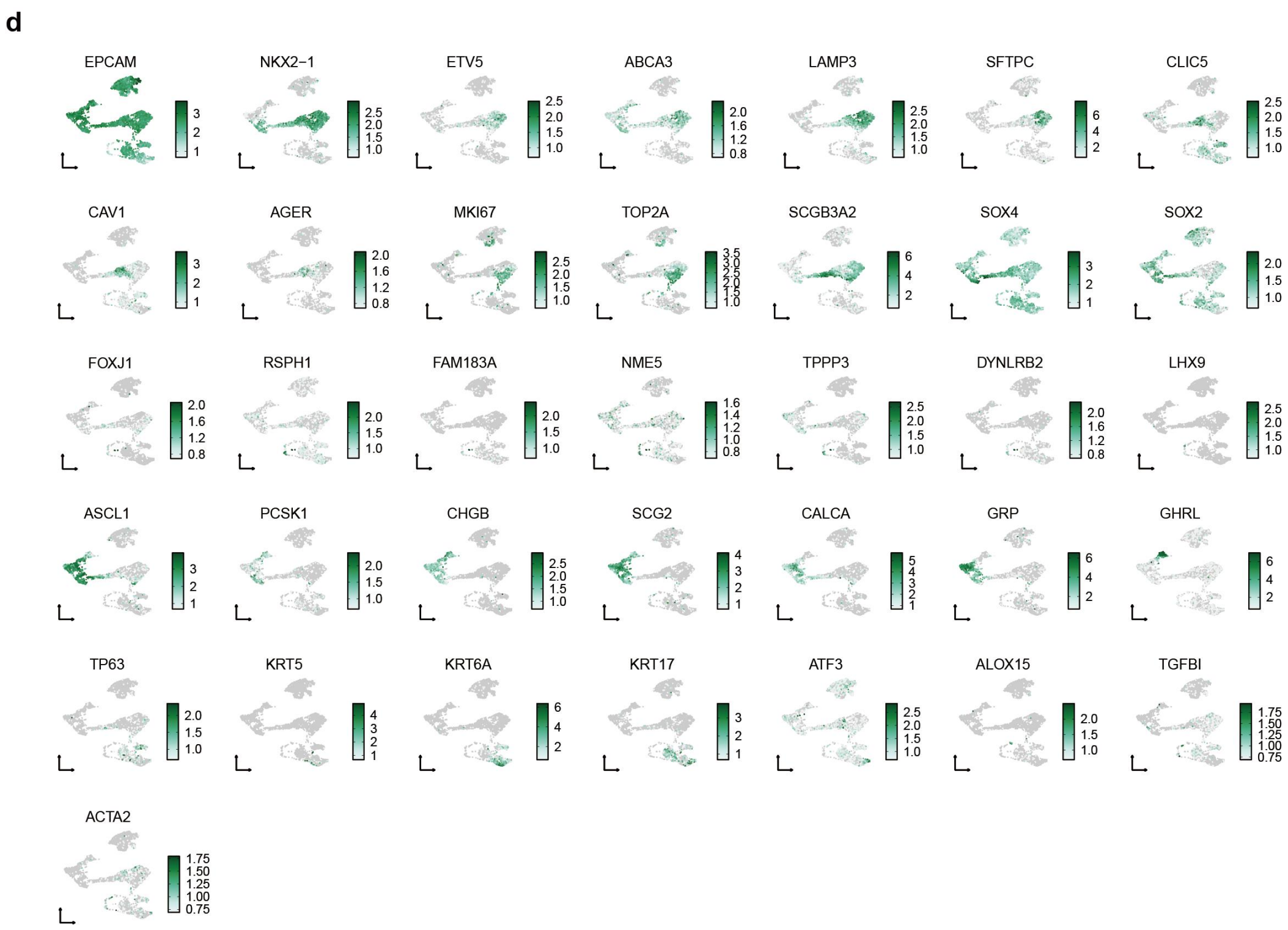

Supplementary Fig. 10d

15. The conclusion in lines 385–387 appears to overstate the strength of the findings. This reviewer recommends rephrasing this to better match the data presented.

We appreciate the reviewer's critical comment. We now rewrite this statement to better reflect the evidence (now page 13 in the revised manuscript):

"Extracellular stiffness gradients can influence region-specific lung epithelial cell fate by modulating the Hippo, hypoxia, Wnt, and TGF-β pathways."

16. Given that GelMA was crosslinked for 30 seconds, some uncrosslinked material may remain. Could this influence cell behavior beyond stiffness?

We appreciate the reviewer's question regarding potential effects of residual uncrosslinked GelMA components beyond stiffness on cell behavior.

1) Previous studies utilized GelMA to encapsulate cells or organoids incubated/washed the crosslinked hydrogels in cell culture medium/PBS and then replaced with the fresh culture medium to effectively remove the unreacted polymer, LAP and PBS (Spencer et al., ACS Appl Mater Interfaces, 2019)[32] (Hu et al., Cells, 2025)[33] (Tabatabaei et al., Journal of Oral Biology and Craniofacial Research, 2020)[34]. For example, Tabatabaei et al. stated that: "The resulting polymerized GelMA hydrogels were washed with PBS to remove unreacted residues, submerged in the regular medium..." Consistently, we have described in the "Methods" of the original manuscript that (now page 17 in the revised manuscript): "Solidified GelMA domes were overlaid with LPCs basal medium to replace the photoinitiator. After 10 minutes, the medium was refreshed and replaced every other day for 7 days." In the revision, we clarify that this step removed uncrosslinked residual polymer, photoinitiator and PBS, that:

"....to replace the uncrosslinked residual polymer, photoinitiator and PBS."

2) However, we acknowledge the possibility that residual uncrosslinked material may still exist and influence cell behavior. Therefore, we evaluated the cytotoxicity of biomaterial extracts in accordance with ISO 10993-5, a standard tests for *in vitro* cytotoxicity of biomaterials, to systematically assess the effects of GelMA hydrogel extracts on LPC viability and development.

We have added the detailed protocol in the "Supplementary Methods" of the revised Supplementary Information (page 24) that:

"3. Biocompatibility testing of GelMA extract according to ISO 10993-5

To determine whether the residual uncrosslinked hydrogel components could affect cell behavior independently of stiffness, the cell viability and differentiation efficiency exposed to hydrogel extracts were assessed following ISO 10993-5 (ISO 10993-5:2009 et al., 2009)[35] with minor modification. Briefly, 25 μL droplets of Materigel (control) and GelMA (G30, G60 and G90) were polymerized without cells (3 repeats per condition), overlaid with 550 μL LPC culture medium, and incubated at 37°Cfor 24h to generate conditioned medium (hydrogel "extracts"). The next day, AFE spheroids were embedded in growth factor reduced Matrigel (Corning, Cat#354230) and polymerized at 37°C for 10 min. The 24 h extracts (Matrigel, G30, G60 and G90) were applied to the Matrigel-embedded AFE spheroids. Media (extracts or control) were replaced every other day. After 7 days, 20 μL of CCK8 (MCE, CAT#HY-K0301) was added per well and incubated for 2 h at 37°C. The absorbance was measured at 450 nm using a microplate reader. Relative cell viability was calculated as below:

$$\text{Relative cell viability (\%)} = (As-Ab)/(Ac-Ab) \times 100\%$$

Where As is the samples absorbance, Ac is the control absorbance, Ab is the blank (without cells) absorbance.
In parallel, LPCs were lysed and total mRNA were extracted for NKX2.1 gene expression analysis (qPCR)."

The CCK8 test and NKX2.1 expression of LPCs indicated cell viability and development were not affected by GelMA extracts, we now describe, in the "Results" section of the revised manuscript (page 6) that:

"Moreover, the extracts from Matrigel and GelMA (G30, G60 and G90) were applied to LPCs embedded in Matrigel to assess viability and differentiation (Supplementary Fig. 2e). CCK8 assays and NKX2-1 gene expression analysis showed the extracts did not affect LPC viability or NKX2-1 levels (Supplementary Fig. 2f, g), indicating that residual unreacted components (if any remained) do not impact LPC viability or differentiation capacity."

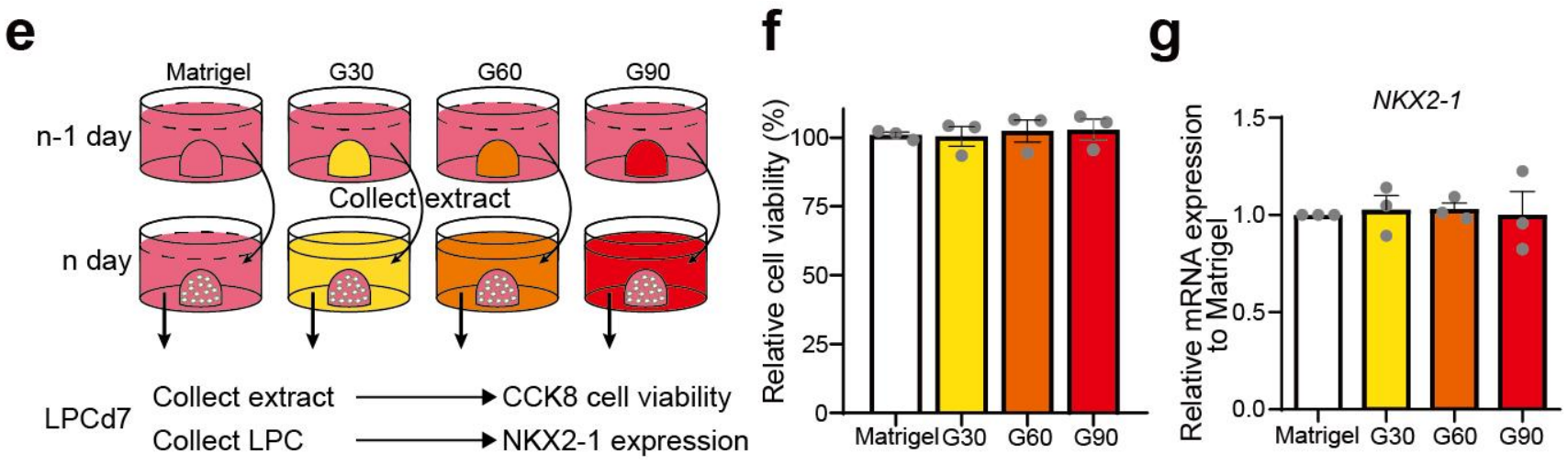

Supplementary Fig. 2

Minor points:

1. The lung environment involves more than ECM and epithelial cells (e.g., endothelial cells, stroma cells, immune components). It would be helpful to acknowledge this complexity in the Introduction.

We appreciate the reviewer's suggestion. We now describe, in the "Introduction" section of the revised manuscript (page 3) that:

"The human respiratory tract is a highly dynamic, multicellular system composed of epithelial, endothelial, mesenchymal, and immune cells that coordinate to maintain structural integrity and respiratory function[6]. Although these multicellular interactions are essential, epithelial cells are central to executing key respiratory functions and are frequently implicated in the pathogenesis of lung diseases[7]."

2. The use of terms like "pleura" may cause confusion since the model focuses on airway and alveolar structures. Please clarify and ensure terminology is used accurately throughout.

We appreciate the reviewer's comment and apologize for the confusion.

According to the reference (Luque et al., Acta Biomaterialia, 2013)[11], the stiffness of pleura is MPa compared to the kPa level close to epithelium which generated a gradient stiffness (MPa to kPa) from pleura to epithelial cells. This gradient stiffness from high to low provides a good explanation for why alveolar epithelium prefer intermediate-to-high stiffness instead of soft one. To make it

more clear without generate confusions, we have moved this sentence from the "Introduction" section to the "Discussion" section to explain the selection of higher stiffness G90. Herein, we now describe, in the "Discussion" section of the revised manuscript (page 15) that:

"Similarly, a pleura-to-alveolar septa stiffness gradient (from MPa to kPa) could impose extra forces on alveolar epithelia, transiently elevating effective alveolar stiffness[64]."

3. Please ensure all abbreviations (e.g., ECM, AFE, AT2) are fully defined at first use.

We apologize for not defining certain abbreviations at first use. We have carefully reviewed the manuscript and identified all abbreviations and revised the manuscript accordingly to ensure that each abbreviation is spelled out upon first use. The changes are summarized in the table below, the abbreviations not fully defined or not abbreviated were modified in our manuscript (Rebuttal Table 1):

Abbreviation	First Appearance (Page)	Full Definition
ECM	Page 3	extracellular matrix
kPa	Page 3	kilopascal
MPa	Page 3	megapascal
MSCs	Page 3	mesenchymal stem cells
AT2	Page 3	alveolar epithelial type 2 cells
AT1	Page 3	alveolar epithelial type 1 cells
hPSC	Page 4	human pluripotent stem cell
GelMA	Page 4	gelatin methacryloyl
LAP	Page 5	lithium phenyl-2,4,6-trimethylbenzoylphosphinate
UV	Page 5	ultraviolet
SEM	Page 5	scanning electron microscopy
LPCs	Page 5	lung progenitor cells
HLOs	Page 5	human lung organoids
TTF1	Page 6	thyroid transcription factor 1
hAWOs	Page 6	human airway organoids
hALOs	Page 6	human alveolar organoids
DE	Page 6	definitive endoderm
AFE	Page 6	anterior foregut endoderm
IF	Page 6	Immunofluorescence
MA	Page 6	methacryloyl
RGD	Page 6	Arg-Gly-Asp
bulk RNA-seq	Page 7	bulk RNA sequencing

TGF-β	Page 7	transforming growth factor beta
Wnt	Page 7	wingless/int-1
SHH	Page 7	sonic hedgehog
PPAR	Page 7	peroxisome proliferator-activated receptor
YAP	Page 7	Yes-associated protein
TEM	Page 8	transmission electron microscopy
scRNA-seq	Page 8	single-cell RNA sequencing
EpCAM	Page 8	epithelial cell adhesion molecule positive
qPCR	Page 8	quantitative polymerase chain reaction
EMT	Page 8	epithelial-to-mesenchymal transition
WB	Page 9	western blot
LBs	Page 10	lamellar bodies
MVs	Page 10	microvilli
TM	Page 10	tubular myelin
TJs	Page 10	tight junctions
DEGs	Page 11	differential expressed genes
IPF	Page 11	idiopathic pulmonary fibrosis
hpi	Page 11	hours post infection
dECM	Page 14	decellularized ECM
HA	Page 14	hyaluronic acid
PEG	Page 14	polyethylene glycol
FAK	Page 14	focal adhesion kinase
ASC	Page 15	adult stem cell
COPD	Page 15	chronic obstructive pulmonary disease
KEGG	Page 19	Kyoto Encyclopedia of Genes and Genomes
GO-BP	Page 19	Gene Ontology-Biological process
PAGA	Page 19	Partition-based Graph Abstraction
BSL-3	Page 20	Biosafety Level 3
MOI	Page 20	multiplicity of infection
AUC	Page 21	area under the curve
PCA	Page 28	Principle component analysis
UMAP	Page 28	Uniform Manifold Approximation and Projection

4. Multiple grammatical errors and incomplete sentences are present throughout the manuscript (e.g., lines 371–373, 375–376). A thorough language edit is recommended.

We appreciate the reviewer's comment. We have thoroughly revised the manuscript to correct grammatical errors and incomplete sentences. In addition, we engaged the Springer Nature Author Services (SNAS) for professional English-language editing. The editing certificate is appended at the end of this rebuttal letter.

5. TEM imaging alone cannot confirm functionality. 1)Please clarify how function was inferred from this result. 2) Please clarify if SEM or TEM imaging was used (and when) in the study.

1) We appreciate the reviewer's comment regarding the interpretation of TEM ultrastructural imaging. We agree that TEM alone cannot establish functionality. In revision, we clarify TEM was used for ultrastructural imaging of specific organelles in the cells, and the functional inference comes from combining these findings with immunofluorescence and live imaging records (e.g. cilia beating). To clarify how function was inferred from TEM results, we have added the depiction of TEM to the "Methods" of the revised manuscript (page 19) that:

"TEM was used to analysis of intracellular morphological ultrastructures of organelles in hAWOs and hALOs (e.g., lamellar bodies, microvilli, tight junction, 9+2 structure) associated with functional maturation." .

We also have revised the "Results" section (page 8) that:

"The ultrastructure of the cilia in G90 appeared longer and denser in longitudinal sections (red arrow, Fig. 2i), and exhibited a canonical 9+2 axonemal architecture (nine doublets surrounding a central pair), indicating the presence of motile, functionally mature cilia. This 9+2 structure was observed in G90 hAWOs but not in G30 or G60 hAWOs (green triangle, Fig. 2i). Cilia consistently exhibited noticeable beating after being released from G90 hydrogels (Supplementary Video 1). Together with the increased FOXJ1+ and AcTUB+ cells in G90 hAWOs, these data indicate that increased extracellular stiffness enhances cilia differentiation and functional maturation."

Furthermore, for the TEM imaging of hALOs, after systematically depicted the ultrastructure of LBs, MV, and TM, we have described in the "Results" section of the revised manuscript (page 10) that:

"Although ultrastructural features do not alone confirm functionality, the presence of mature LBs, MVs and TMs is considered a hallmark of functionally mature AT2 cells, because they are closely associated with the ability to synthesize, store, and secrete pulmonary surfactant[41]."

2) Only TEM was used to obtain ultrastructural images in the current study. To avoid confusion, we now add the depiction of "TEM analysis" before presenting the TEM data. In the "Results" section of the revised manuscript (pages 8 and 10), we state that:

HAWO: "Transmission electron microscopy (TEM) analysis was used to examine intracellular ultrastructures features of organelles."

HALO: "TEM imaging was performed to visualize intracellular ultrastructural features of organelles."

6. The statement on line 325 overstates the interpretation of the presented data. Consider softening the conclusion to align with the evidence presented, and consider the physiological relevance.

We appreciate reviewer's comment and suggestion. We have softened the conclusion and align with our observations. We now describe their physiological relevance in the "Discussion" section of the revised manuscript (page 13) as below:

"….facilitating the generation of region-specific lung organoids that recapitulate key aspects of *in vivo* lung epithelial differentiation, and providing a platform to study extracellular stiffness in region-specific lung generation and disease modeling."

7. You mention that mesenchymal-secreted ECM proteins alter lung ECM mechanics (Lines 330–332). Were any of these components included in your culture conditions?

We appreciate the reviewer's question.

No, our current culture system did not incorporate exogenous ECM proteins. Organoids were embedded in gelatin-based GelMA hydrogels. Gelatin inherently contains abundant RGD motifs that provide adhesive binding sites for embedded cells. This design was intentional to isolate the effects of hydrogel stiffness on epithelial patterning, independent of region-specific biochemical components derived from the ECM. We have expanded the "Discussion" section to emphasize the focus of this study is on ECM stiffness instead of ECM composition (page 14) that:

"In the current study, we focused specifically on how extracellular stiffness influences lung epithelial cell fate. To minimize confounding effects from region-specific ECM components, we used gelatin-based GelMA hydrogels without the introduction of exogenous biochemical signals (Supplementary Fig. 1). This design allowed us to determine the effect of stiffness on respiratory epithelial patterning."

8. The claim that decellularization disrupts ECM structure and stiffness is not entirely accurate (Line 349). This statement should be supported with evidence. Reference to prior work in this area should be made.

We thank the reviewer for this critical comment. We realized that we had inadvertently conflated decellularized ECM (dECM) hydrogels with dECM scaffolds. The key difference is that dECM scaffolds retain native structure and architecture, whereas dECM hydrogels are prepared from minced dECM powder (Hoffman et al., Biomaterials, 2023)[36] (Golebiowska et al., Bioactive Materials, 2024)[37] (Kuşoğlu et al., ACS Appl Bio Mater, 2023)[38]. To avoid confusion, we have removed the statement that "decellularization disrupts ECM structure and stiffness", instead, we summarize the applications of both dECM scaffolds and hydrogels in lung epithelial fate determination. We now describe, in the "Discussion" section of the revised manuscript (page 14) that:

"Decellularized ECM (dECM) scaffolds and hydrogels provide biomimetic 3D culture environments that preserve region-specific instructive cues[54]. hPSC-derived lung epithelial cells exposed to alveolar region-enriched dECM adopt distal or alveolar phenotypes, while the nonregional lung dECM exhibit enhanced lung progenitors formation and maturation[55]. In contrast, trachea-derived dECM scaffolds or hydrogels support proliferation of bronchial epithelial cells and

the formation of a confluent epithelial layer[56]. Together, these observations support the notion that region-matched dECM directs epithelial fate *in vitro*."

9. The mention of hyaluronic acid (HA) (Lines 350–351) promoting bronchiolar progenitors seems out of context. Consider removing or better integrating this information.

We appreciate the reviewer's suggestion.

We moved the "hyaluronic acid (HA) (original lines 350–351) promoting bronchiolar progenitors" to the section discussing ECM components that regulate lung epithelial cell fate (revised page 14), because HA is a major component of alveolar ECM. We now describe, in the "Discussion" section of the revised manuscript (page 14) that:

"Hyaluronic acid (HA) is a major constituent of the lung interstitium and alveolar space. The incorporation of HA into polyethylene glycol (PEG)-Matrigel systems significantly increased viscoelasticity and triggered mechanotransduction, which promoted alveolar progenitor cell differentiation[31]."

10. Please revise incomplete sentences in lines 371–373 and 375–376 to ensure clarity.

We thank the reviewer for pointing out these incomplete sentences. We have revised Lines 371-373 and 375-376 (Now page 14 in the revised manuscript) for clarity and grammatical correctness as follows:

Page 14 (original lines 371-373) "Physical forces generated by ECM are sensed by cells and transduced into intracellular signals through several primary pathways, including Hippo, mechanotransduction cascades, and focal adhesion kinase (FAK) signaling pathways[57]."

Page 14 (original lines 375-376) "Our study indicates that ECM stiffness modulates lung development by regulating the Hippo pathway, which in turn regulates the specification of lung progenitors and the differentiation of airway and alveolar epithelial cell fates (Fig. 1, 3 and 5)."

Reviewer #3 :

I co-reviewed this manuscript with one of the reviewers who provided the listed reports. This is part of the Nature Communications initiative to facilitate training in peer review and to provide appropriate recognition for Early Career Researchers who co-review manuscripts.

We sincerely thank the reviewer for the comments and suggestions. We appreciate the opportunity to benefit from both senior and early-career perspectives, which provided valuable insights into improving the quality of our study.

Reviewer #4 :

Liao et al investigated how extracellular matrix (ECM) stiffness regulates region-specific differentiation of human pluripotent stem cell (hPSC)-derived lung organoids. By establishing a stiffness-tunable GelMA hydrogel platform, the authors provide a valuable model system to dissect how mechanical cues influence proximal-to-distal airway and alveolar epithelial fate

decisions. The integration of mechanical modeling with transcriptomic and single-cell analyses offers new insights into the ECM-related mechanisms underlying lung development, particularly involving the Hippo, Wnt, TGF-β, and hypoxia pathways. The data are clearly delivered and fully support the conclusion. Herein, several issues may have to be addressed to improve the scientific vigor of the manuscript.

We thank the reviewer for reviewing our manuscript.

1. The authors mention that GelMA stiffness ranges from 3 to 15 kPa to mimic physiological lung stiffness. It would be helpful to more explicitly discuss how these stiffness levels compare to the in vivo stiffness ranges of the proximal airways, bronchioles, and alveoli. This would enhance the physiological relevance of the model.

We thank the reviewer for this insightful suggestion. We addressed this point in our response to Reviewer 2, Comment 3, in brief, we have stated in the "Discussion" section of the revised manuscript (page 15) that:

"To recapitulate the proximal–distal mechanical gradient of the respiratory system, we employed three GelMA hydrogels (G30, G60 and G90) with stiffness ranging from 2.99 kPa to 11.31 kPa (Supplementary Fig. 1c), covering reported lung regional values (0.5-15 kPa) and fetal lung stiffness (3.68-5 kPa) (Nallet et al., Eur Radiol, 2022)[7] (Guimarães et al., Nature Reviews Materials, 2020)[39] "

We respectfully refer Reviewer 4 to that section for full details.

2. The authors may have to be cautious using "lung". E.g., line 81, consider replacing "the lung" with "the human respiratory tract". Line 85, revise "the lung epithelium" as "the airway epithelium"; and delete "lung" line 111. The authors are suggested to check the whole manuscript to ensure the correct usage of "respiratory" and "lung".

We appreciate the reviewer's suggestion. We have revised the text throughout the manuscript to distinguish between "lung," "respiratory tract," "airway epithelium," and "alveolar epithelium" as appropriate (e.g., in the revised manuscript pages 2, 3, 4, 5, 7, 13, 14, 15), to ensure accurate anatomical references.

3. 1)I am confused about the usage of "secretory cells" by the authors. In line 86, it may refer to goblet cell and club cell. 2)In line 162, UGRP1 and uteroglobin are marker genes of club cells. please clarify.

We appreciate the reviewer's comment and apologize for the confusion.

1) Regarding the usage of "secretory cells"

We referenced the paper published by Basil et al (Basil et al., Nature, 2022)[29], which identified two secretory cell populations located in distinct regions of the human respiratory airways: SCGB1A1+ and SCGB3A2+ cells. They reported that: "Proximal airways lack SCGB3A2+ cells." "The secretory cells in the proximal airways were identified by expression of SCGB1A1. However, only rare cells in the proximal airways expressed SCGB3A2." "SCGB3A2+ cells lined the distal most

regions of these airways, whereas SCGB1A1+ cells were observed in the terminal bronchioles." Therefore, we used "secretory cells" to refer to cells expressing either SCGB1A1 or SCGB3A2.

However, as the reviewer pointed out, proximal secretory cells include both club cells and mucus-secreting goblet cells. To improve clarity, we have revised the terminology through the manuscript. The secretory cells are now specifically referred to as SCGB1A1+ secretory cells and SCGB3A2+ secretory cells.

In our scRNA-seq results (Fig. 3c), secretory1 corresponds to the SCGB3A2+ cluster, whereas secretory 2 corresponds to SCGB1A1+ cluster. Therefore, we renamed these clusters SCGB1A1+ secretory cells (secretory 2) and SCGB3A2+ secretory cells (secretory 1), following Basil's annotation.

We now annotated three secretory-cell clusters, they are goblet cells (MUC5AC+), SCGB1A1+ secretory cells and SCGB3A2+ secretory cells. The manuscript text and figure labels have been thoroughly revised and corrected to apply this nomenclature consistently.

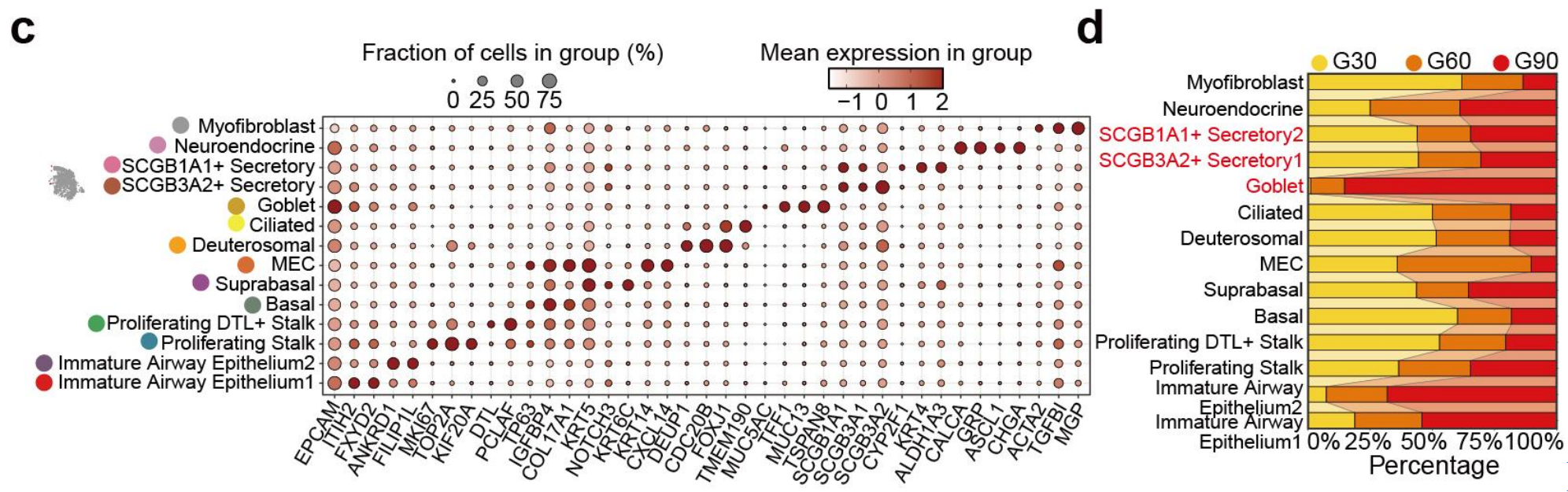

Fig. 3c, d

2) Regarding whether the UGRP1 and uteroglobin are the marker genes for club cells

The UGRP1 antibody (goat anti-UGRP1/SCGB3A2, R&D systems, AF3545) we used in current study specifically detects SCGB3A2. This antibody has been validated in prior studies [e.g. (Kadur Lakshminarasimha Murthy et al., Nature, 2022)[40] (Basil et al., Nature, 2022)[29]] to identify SCGB3A2+ secretory cells in human distal airway.

The uteroglobin antibody (mouse anti-uteroglobin/SCGB1A1, Santa Cruz Biotechnology, Sc-365992) specifically detects SCGB1A1 (Tindle et al., eLife, 2021)[41].

To avoid confusion, we have revised the manuscript text and figures to replace "UGRP1" and "uteroglobin" with SCGB3A2 and SCGB1A1 (Fig. 2c, d, e), respectively.

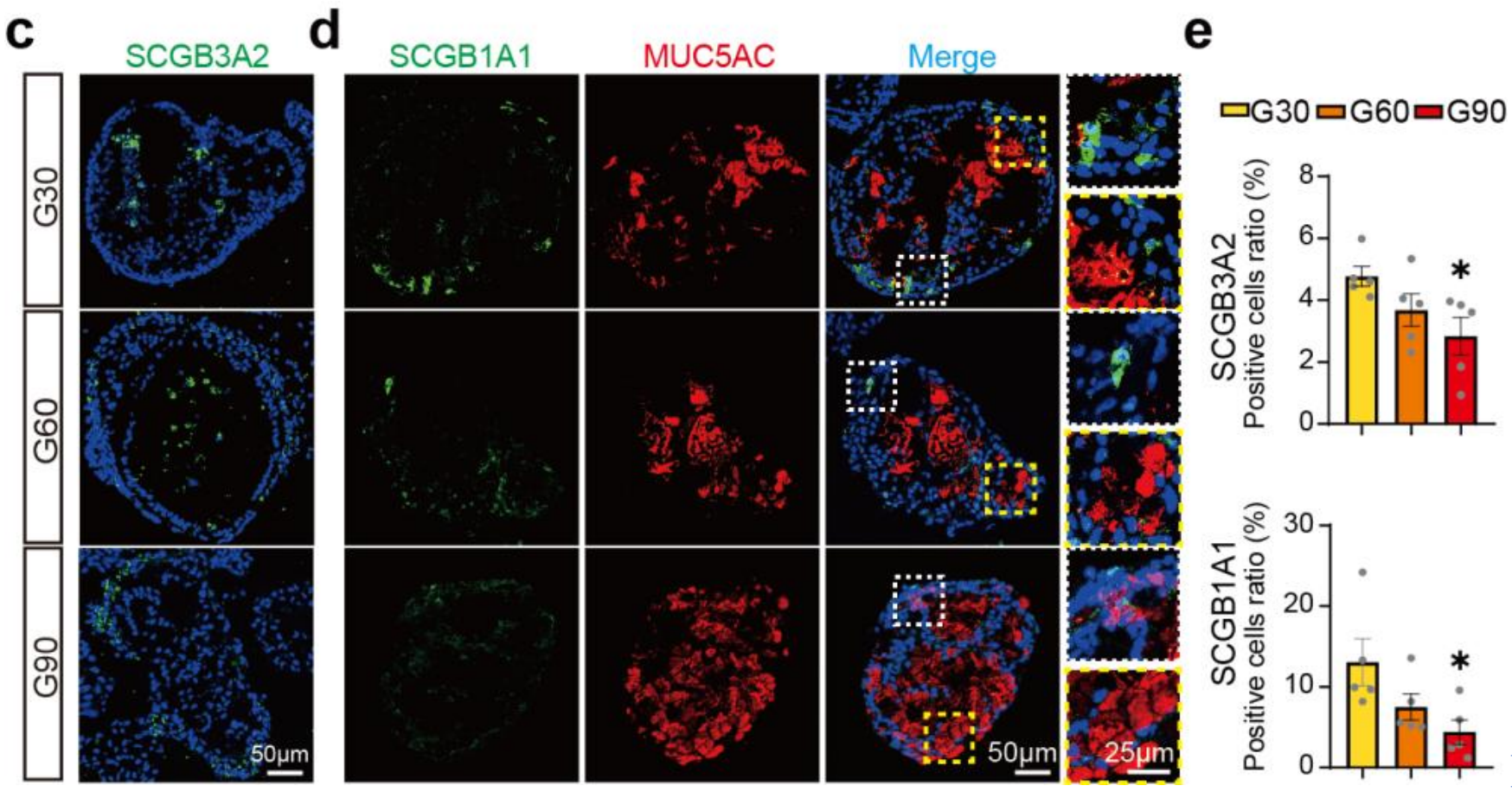

Fig. 2c, d, e

4. While focusing on ECM stiffness as a key regulator of epithelial cell fate, the authors may have to acknowledge that other aspects of the microenvironment, e.g. ECM remodeling, matrix topography etc, also play critical roles. The authors are suggested to briefly discuss these factors in the manuscript (e.g., Jones et al., 2024, Science; Courbot et al., 2025, NPJ Regenerative Medicine).

We thank the reviewer for the suggestion. To acknowledge these additional extracellular microenvironmental factors, we have expanded the "Discussion" section of the revised manuscript (page 15) and incorporated the suggested recent, high-impact references (Jones et al., Science, 2024)[42] (Courbot et al., NPJ Biol Phys Mech, 2025)[43] as follows:

"In addition to stiffness and composition, other microenvironmental properties, such as ECM remodeling, matrix topography and viscoelasticity, critically regulate lung epithelial fate and regeneration by altering stress relaxation, mechanotransduction, matrix topography and niche plasticity[59, 60]. Integrating tunable viscoelasticity and controlled degradability into hydrogels, together with 3D-printing and micropatterning, would more comprehensively recapitulate the native lung microenvironment."

5. The authors may discuss how hiPSC-derived region-specific lung organoids compare to adult stem cell-derived respiratory organoids in cellular composition and biological relevance. Suggested references: Chiu et al., 2022 Cell Discovery; Li et al., 2024 Nature Communications.

We thank the reviewer for the suggestion. We have added the following comparison between hiPSC-derived and adult stem cell-derived respiratory organoids to the "Discussion" section of the revised manuscript (page 16) that:

"Both hPSC- and lung tissue-specific adult stem cell (ASC)-derived respiratory organoids recapitulate key structural and functional properties of the respiratory epithelium[47]. Their cellular compositions are broadly similar, comprising four major airway epithelial cells (ciliated, goblet, club and basal cells) and two principal alveolar epithelial cell types (AT2 and AT1)[66]. ASC-derived respiratory organoids yield more mature and homogeneous epithelial cells with minimal off-target differentiation but are limited by tissue access. In contrast, hPSC-derived respiratory organoids provide an essentially unlimited cell source but are often immature with high heterogeneity and long differentiation timelines. "

6. The demonstration of region-specific SARS-CoV-2 infection highlights the physiological relevance of the lung organoids. The authors may consider extending the discussion by linking ECM stiffness-related pulmonary diseases, such as IPF and COPD.

We appreciate the reviewer's valuable suggestion. In response, we added a discussion of ECM stiffness-related disease adjacent to the discussion of hiPSC- and hASC-derived organoids, highlighting the potential of stiffness-tunable hydrogel *in vitro* model for future studies. We now state in the "Discussion" section of the revised manuscript (page 16) that:

"ECM stiffness is a central pathological feature of idiopathic pulmonary fibrosis (IPF) and chronic obstructive pulmonary disease (COPD), in which matrix remodeling alters tissue mechanics and actively drives epithelial cell behaviors[67]. In future studies, combining patient-derived ASC organoids with stiffness-tunable hydrogels will enable personalized *in vitro* models to explore how extracellular mechanics contribute to epithelial plasticity, injury responses and fibrosis progression in diseases such as IPF and COPD."

7. Minor issues
1) Line 54. This study discover.
2) Line 93. Please specify the stage for "budding morphogenesis in embryonic lungs."
3) Please clarify figure legends (e.g., arrows in Figures 2i and 3h) and define abbreviations (e.g., "LBs," "TJs") at first mention.
4) Line 131. Exhibited ◊ induced?

We thank the reviewer for spotting these. All suggested changes have been made:

1) Revised page 3 (original line 54): "This study discovers";

2) Revised page 4 (original line 93): "embryonic lung bud (E10.5–E12.5)";

3) We have clarified figure legends:

Fig. 2h: "TEM images of goblet cells showing secretory vesicles (blue arrows) in organoids.";

Fig. 2i: "TEM images of ciliated cells under G30, G60, and G90 in longitudinal sections (red arrow) and in transverse sections (green triangle) at high stiffness.";

Fig. 4h (original Fig. 3h): "TEM images demonstrating the presence of lamellar bodies (LBs) (red arrow) in AT2 cells, LBs with organized layers appear in G60 and G90.";

Fig. 4i (original Fig. 3i): "TEM images illustrating the tight junctions (TJs) (yellow arrow) in AT1 cells, the TJs become thinner with increased stiffness (G90).";

Fig. 5g: "White arrow indicates the cell with cytoplasmic YAP; red arrow indicates the cell with cytoplasm-to-nuclear translocated YAP; yellow arrow indicates the cell with nuclear YAP.";

Fig. 5h: "IF staining of YAP (green), TAZ (red), and AGER (white) in G30, G60, and G90; scale bars, 20 µm. White arrow indicates the cell with cytoplasmic YAP and nuclear TAZ; red arrow indicates the cell with nuclear YAP and nuclear TAZ."

4) In response to Reviewer 2's Minor Point 3, we compiled Rebuttal Table 1, which lists all abbreviations and their definitions. We respectfully refer our Reviewer to Rebuttal Table 1 (pages 24-25) for full details.

After professional English-language editing by Springer Nature Author Services (SNAS), the revised line page 6 (original line 131) has been revised to: "Immunofluorescence (IF) confirmed increased NKX2-1 expression in G90 LPCs with strong colocalization of SOX2 and SOX9 (Fig. 1d)."

References

1. Chiu MC, *et al.* A bipotential organoid model of respiratory epithelium recapitulates high infectivity of SARS-CoV-2 Omicron variant. *Cell Discov* **8**, 57 (2022).
2. Li X, *et al.* 3D Culture of Chondrocytes in Gelatin Hydrogels with Different Stiffness (2016).
3. Chansoria P, Asif S, Polkoff K, Chung J, Piedrahita JA, Shirwaiker RA. Characterizing the Effects of Synergistic Thermal and Photo-Cross-Linking during Biofabrication on the Structural and Functional Properties of Gelatin Methacryloyl (GelMA) Hydrogels. *ACS Biomater Sci Eng* **7**, 5175-5188 (2021).
4. Dye BR, *et al.* In vitro generation of human pluripotent stem cell derived lung organoids. *Elife* **4**, e05098 (2015).
5. Hein RFC, *et al.* Stable iPSC-derived NKX2-1+ lung bud tip progenitor organoids give rise to airway and alveolar cell types. *Development* **149**, dev200693 (2022).
6. Miller AJ, *et al.* Generation of lung organoids from human pluripotent stem cells in vitro. *Nat Protoc* **14**, 518-540 (2019).
7. Nallet C, *et al.* Prenatal quantification of human foetal lung and liver elasticities between 24 and 39 weeks of gestation using 2D shear wave elastography. *Eur Radiol* **32**, 5559-5567 (2022).
8. Guo T, He C, Venado A, Zhou Y. Extracellular Matrix Stiffness in Lung Health and Disease. *Compr Physiol* **12**, 3523-3558 (2022).
9. Baruffaldi D, Palmara G, Pirri C, Frascella F. 3D Cell Culture: Recent Development in Materials with Tunable Stiffness. *ACS Appl Bio Mater* **4**, 2233-2250 (2021).
10. Cadena MA, *et al.* A 3D Bioprinted Cortical Organoid Platform for Modeling Human Brain Development. *Adv Healthc Mater* **13**, e2401603 (2024).
11. Luque T, *et al.* Local micromechanical properties of decellularized lung scaffolds measured with atomic force microscopy. *Acta Biomaterialia* **9**, 6852-6859 (2013).
12. Li J, Wang Z, Chu Q, Jiang K, Li J, Tang N. The Strength of Mechanical Forces Determines the Differentiation of Alveolar Epithelial Cells. *Dev Cell* **44**, 297-312 e295 (2018).
13. Shiraishi K, *et al.* Biophysical forces mediated by respiration maintain lung alveolar epithelial cell fate. *Cell* **186**, 1478-1492 e1415 (2023).
14. Bammert MT, *et al.* JUNB O-GlcNAcylation-Mediated Promoter Accessibility of Metabolic Genes Modulates Distinct Epithelial Lineage in Pulmonary Fibrosis. *Adv Sci (Weinh)* **12**, e2406751 (2025).
15. Liu D, Jiang Q, Xu Z, Li L, Lyu G. Evaluating fetal lung development at various gestational weeks using two-dimensional shear wave elastography. *Quant Imaging Med Surg* **14**, 5373-5384 (2024).
16. Bennet TJ, Randhawa A, Hua J, Cheung KC. Airway-On-A-Chip: Designs and Applications for Lung

Repair and Disease. *Cells* **10**, 1602 (2021).

17. Liu Y, *et al.* Engineered Hydrogels for Organoid Models of Human Nonalcoholic Fatty Liver Disease. *Adv Sci (Weinh)* **12**, e17332 (2025).

18. He S, *et al.* Stiffness Restricts the Stemness of the Intestinal Stem Cells and Skews Their Differentiation Toward Goblet Cells. *Gastroenterology* **164**, 1137-1151 e1115 (2023).

19. Cruz-Acuna R, *et al.* Engineered hydrogel reveals contribution of matrix mechanics to esophageal adenocarcinoma and identifies matrix-activated therapeutic targets. *J Clin Invest* **133**, (2023).

20. Nguyen J, *et al.* Culture substrate stiffness impacts human myoblast contractility-dependent proliferation and nuclear envelope wrinkling. *J Cell Sci* **137**, (2024).

21. Brock J, Erhardt J, Eisler SA, Hörning M. Optimization of Mechanosensitive Cross-Talk between Matrix Stiffness and Protein Density: Independent Matrix Properties Regulate Spreading Dynamics of Myocytes. *Cells* **11**, (2022).

22. Blackford SJI, *et al.* RGD density along with substrate stiffness regulate hPSC hepatocyte functionality through YAP signalling. *Biomaterials* **293**, 121982 (2023).

23. Conway JRW, *et al.* Defined extracellular matrix compositions support stiffness-insensitive cell spreading and adhesion signaling. *Proc Natl Acad Sci U S A* **120**, e2304288120 (2023).

24. Indana D, Agarwal P, Bhutani N, Chaudhuri O. Viscoelasticity and Adhesion Signaling in Biomaterials Control Human Pluripotent Stem Cell Morphogenesis in 3D Culture. *Adv Mater* **33**, e2101966 (2021).

25. Budeus B, Kroepel C, Stasch LM, Klein D. Matrix-free human lung organoids derived from induced pluripotent stem cells to model lung injury. *Stem Cell Research & Therapy* **15**, 468 (2024).

26. Hawkins F, *et al.* Prospective isolation of NKX2-1-expressing human lung progenitors derived from pluripotent stem cells. *J Clin Invest* **127**, 2277-2294 (2017).

27. Tamai K, *et al.* iPSC-derived mesenchymal cells that support alveolar organoid development. *Cell Rep Methods* **2**, 100314 (2022).

28. He P, *et al.* A human fetal lung cell atlas uncovers proximal-distal gradients of differentiation and key regulators of epithelial fates. *Cell* **185**, 4841-4860.e4825 (2022).

29. Basil MC, *et al.* Human distal airways contain a multipotent secretory cell that can regenerate alveoli. *Nature* **604**, 120-126 (2022).

30. Nguyen HT, *et al.* Viral small T oncoproteins transform cells by alleviating hippo-pathway-mediated inhibition of the YAP proto-oncogene. *Cell Rep* **8**, 707-713 (2014).

31. Chen J, Wang X, He Q, Yang HC, Fogo AB, Harris RC. Inhibition of transcriptional coactivator YAP Impairs the expression and function of transcription factor WT1 in diabetic podocyte injury. *Kidney Int* **105**, 1200-1211 (2024).

32. Spencer AR, *et al.* Bioprinting of a Cell-Laden Conductive Hydrogel Composite. *ACS Appl Mater Interfaces* **11**, 30518-30533 (2019).

33. Hu W, Xie X, Xu J. Epimedium-Derived Exosome-Loaded GelMA Hydrogel Enhances MC3T3-E1 Osteogenesis via PI3K/Akt Pathway (2025).

34. Tabatabaei F, Moharamzadeh K, Tayebi L. Fibroblast encapsulation in gelatin methacryloyl (GelMA) versus collagen hydrogel as substrates for oral mucosa tissue engineering. *Journal of Oral Biology and Craniofacial Research* **10**, 573-577 (2020).

35. ISO10993-5:2009. *Biological evaluation of medical devices — Part 5: Tests for in vitro cytotoxicity.* ISO (International Organization for Standardization) (2009).

36. Hoffman ET, *et al.* Regional and disease specific human lung extracellular matrix composition.

Biomaterials **293**, 121960 (2023).

37. Golebiowska AA, Intravaia JT, Sathe VM, Kumbar SG, Nukavarapu SP. Decellularized extracellular matrix biomaterials for regenerative therapies: Advances, challenges and clinical prospects. *Bioactive Materials* **32**, 98-123 (2024).

38. Kuşoğlu A*, et al.* Different Decellularization Methods in Bovine Lung Tissue Reveals Distinct Biochemical Composition, Stiffness, and Viscoelasticity in Reconstituted Hydrogels. *ACS Appl Bio Mater* **6**, 793-805 (2023).

39. Guimarães CF, Gasperini L, Marques AP, Reis RL. The stiffness of living tissues and its implications for tissue engineering. *Nature Reviews Materials* **5**, 351-370 (2020).

40. Kadur Lakshminarasimha Murthy P*, et al.* Human distal lung maps and lineage hierarchies reveal a bipotent progenitor. *Nature* **604**, 111-119 (2022).

41. Tindle C*, et al.* Adult stem cell-derived complete lung organoid models emulate lung disease in COVID-19. *eLife* **10**, e66417 (2021).

42. Jones DL*, et al.* An injury-induced mesenchymal-epithelial cell niche coordinates regenerative responses in the lung. *Science* **386**, eado5561 (2024).

43. Courbot O, Elosegui-Artola A. The role of extracellular matrix viscoelasticity in development and disease. *NPJ Biol Phys Mech* **2**, 10 (2025).

SPRINGER NATURE
Author Services

Editing Certificate

This document certifies that the manuscript

Extracellular stiffness directs region-specific lung epithelial differentiation revealed by hPSC-derived lung organoids

prepared by the authors

Zhiying Liao, Hao Meng, Junjie Lv, Dong Wang, Hengrui Zhang, Runxi jiang, Ruihao Lan, Yu Chen, Jiaqi Sun, Zonghong Li, Xiangping Yang, Xuepeng Chen, Jincun Zhao, Tao Xu, Huisheng Liu

was edited for proper English language, grammar, punctuation, spelling, and overall style by one or more of the highly qualified English speaking editors at SNAS.

This certificate was issued on **September 10, 2025** and may be verified on the SNAS website using the verification code **7ED1-1C41-5304-28E6-1433** .

Neither the research content nor the authors' intentions were altered in any way during the editing process. Documents receiving this certification should be English-ready for publication; however, the author has the ability to accept or reject our suggestions and changes. To verify the final SNAS edited version, please visit our verification page at secure.authorservices.springernature.com/certificate/verify.
If you have any questions or concerns about this edited document, please contact SNAS at support@as.springernature.com.

SNAS provides a range of editing, translation, and manuscript services for researchers and publishers around the world.
For more information about our company, services, and partner discounts, please visit authorservices.springernature.com.

We are grateful for the reviewers' insightful comments and valuable suggestions that have helped us in making our manuscript stronger. As a result of addressing all the comments from the reviewers, we have modified a total of 48 figure panels (22 in the main figures and 26 in the supplementary figures), added a total of 20 new panels (14 to the main figures and 6 to the supplementary figures), revised 2 supplementary tables and 1 supplementary video, added 6 new supplementary videos, provided more detailed methods, expanded the results and removed irrelevant contents from the discussion.

We have addressed each of the concerns raised by the reviewers as follows:

1) We have specified extracellular matrix stiffness in the title.

2) We have provided additional physiological validation of region-specific hAWOs and hALOs and clarified the anatomical scope and relevance of the airway segments.

3) We have addressed the concern of RGD density and corrected the errors in the mechanical testing of our gradient stiffness hydrogel.

4) We have revised the discussion about the mechanical cues beyond mechanical stiffness in cell fate determination.

5) We have performed two additional scRNA-seq biological replicates and integrated them with the original datasets to reanalyze the data and regenerate the figures.

Please see below for our point-by-point response to the comments.

Reviewer #1 (Remarks to the Author):

The authors have addressed my concerns.

Thank you for your positive feedback. We are glad that our revisions have addressed your concerns.

Reviewer #2 (Remarks to the Author):

Thank you for your extensive responses to the previous round of comments. We still have several remaining questions:

Major comments:

1. Title
The revised title is not a complete or precise phrase. "Extracellular stiffness" is ambiguous; it should read "Extracellular matrix stiffness."

We thank the reviewer for pointing out this issue. As suggested, we have revised the title into:

"Extracellular matrix stiffness directs region-specific lung epithelial differentiation revealed by hPSC-derived lung organoids."

2. SARS-CoV-2 data as physiological validation
Using SARS-CoV-2 data to demonstrate physiological relevance remains unconvincing. Although the authors base this work on a finding that has been published, this approach is not widely adopted or well established for model validation. Please consider adding more physiologically relevant and established methods to substantiate the model.

We sincerely thank the reviewer for this valuable suggestion.

To further substantiate the physiological relevance of the model, we performed 1) mucociliary functional assays for hAWOs (new Fig. 3a-h, together with experiments included in the previous version: 2; Supplementary Videos 1-3); 2) forskolin induced swelling (FIS) (new Fig. 3i-l); 1) and 2) together are as established physiological assays for hAWOs. 3) Lamellar body maturation markers co-staining of ABCA3 and SFTPB (new Fig. 6b-d, together with experiments included in the previous version: 4) lipid trafficking assays (now new Fig. 6a) and 5) TEM imaging of lamillar body (LB, AT2) and intercellular junction (AT1) (now new Fig. 6h, i); 3), 4) and 5) together provide physiologically relevant validation of the hALOs model. We have described these in the "Results" section (page 7 and page 11) that:

"**Physiological Validation of Proximal-to-Distal hAWOs Specified in Stiff-to-Soft Hydrogels**

To validate the physiological relevance of the stiffness-guided, region-specific hAWOs, we performed established functional assays for mucociliary defense[42, 43] and epithelial ion and fluid transport capacity [9, 44]. Since the major differences among G30-, G60-, and G90-hAWOs were the distribution of ciliated cells and secretory lineages (e.g., goblet- and ciliated-enriched features in G90-hAWOs versus SCGB1A1/SCGB3A2-secretory cells enriched in G30-hAWOs), we assessed ciliary motility and mucociliary transport to evaluate cilia-related function and forskolin-induced swelling (FIS) which is related to secretory cell function.

Physiologically, motile cilia provide a critical airway defense barrier by rhythmically beating to propel mucus and trapped pathogens toward the pharynx. Our results indicated ciliary beat frequency (CBF) (Supplementary Videos 1-3 and Fig. 3a) was significantly increased in G90-AWOs (8.34 ± 0.35 Hz) compared with G30 (3.78 ± 0.16 Hz) and G60-AWOs (6.97 ± 0.15 Hz). The cilia on G90-hAWOs (5.43 ± 0.06 µm) and G60-hAWOs (4.93 ± 0.20 µm) were also significantly longer than those in G30 (3.15 ± 0.35 µm) (Fig 3b, c). Transmission electron microscopy (TEM) analysis was used to examine intracellular ultrastructures features of organelles. Ultrastructure of the cilia in G90 appeared longer and denser in longitudinal sections (red arrow, Fig. 3d), and exhibited a canonical 9+2 axonemal architecture (nine doublets surrounding a central pair), indicating the presence of motile, functionally mature cilia (green triangle, Fig. 3d). With increasing stiffness, the goblet cells in G90 displayed more numerous and larger mucus vesicles (blue triangle, Supplementary Fig. 4e). Together, cilia and goblet cells contribute to functional mucociliary clearance. Additionally, G90-hAWOs exhibited the largest ciliary beat amplitude (CBA) (4.17 ± 0.20 µm; Fig. 3e, f), likely reflecting their higher CBF and longer cilia (Fig. 3a-d). To measure mucociliary transport, fluorescent beads were tracked (Fig. 3g). Functionally, G90-hAWOs showed the highest particle clearance speed (2.74 ± 0.076) compared with G60-hAWOs (2.40 ± 0.031) and G30-hAWOs (2.06 ± 0.058) (Fig. 3h). Together, these data indicate that region-specific hAWOs demonstrate a more robust mucociliary clearance phenotype in G90-hAWOs which show greater coverage of motile cilia and goblet cells mimicking proximal airways, compared with the lower cilia coverage mimicking distal airways in G60- and G30-hAWOs.

Forskolin-induced swelling is widely used to assess cAMP-dependent anion or fluid transport and functional maturation of airway secretory epithelium. The hAWOs from G30, G60, and G90 all showed significant swelling in response to forskolin treatment after 24 hours (Fig. 3i, j) with 3.09-,

2.45- and 2.18-fold increases, respectively (Fig. 3k). The average swelling rates of G30-, G60- and G90-hAWOs were 0.603, 0.589, and 0.596-fold/h, respectively, in the first 2 hours, and 0.116, 0.095, 0.083-fold/h, respectively, over the subsequent 22 hours (Fig. 3l), indicating functional epithelial fluid secretion capacity. Although the early responses were comparable among three groups, swelling divergence became more evident over time, with G90-hAWOs displaying significantly less swelling in normalized area than G30-hAWOs. Taken together, by modulating ECM stiffness, we can obtain hAWOs with different airway epithelial cell compositions with proximal-like mucociliary defense properties versus more distal-small airway like FIS response, thereby providing region-specific-mimic hAWOs for modeling regional specific airway physiology and disease.

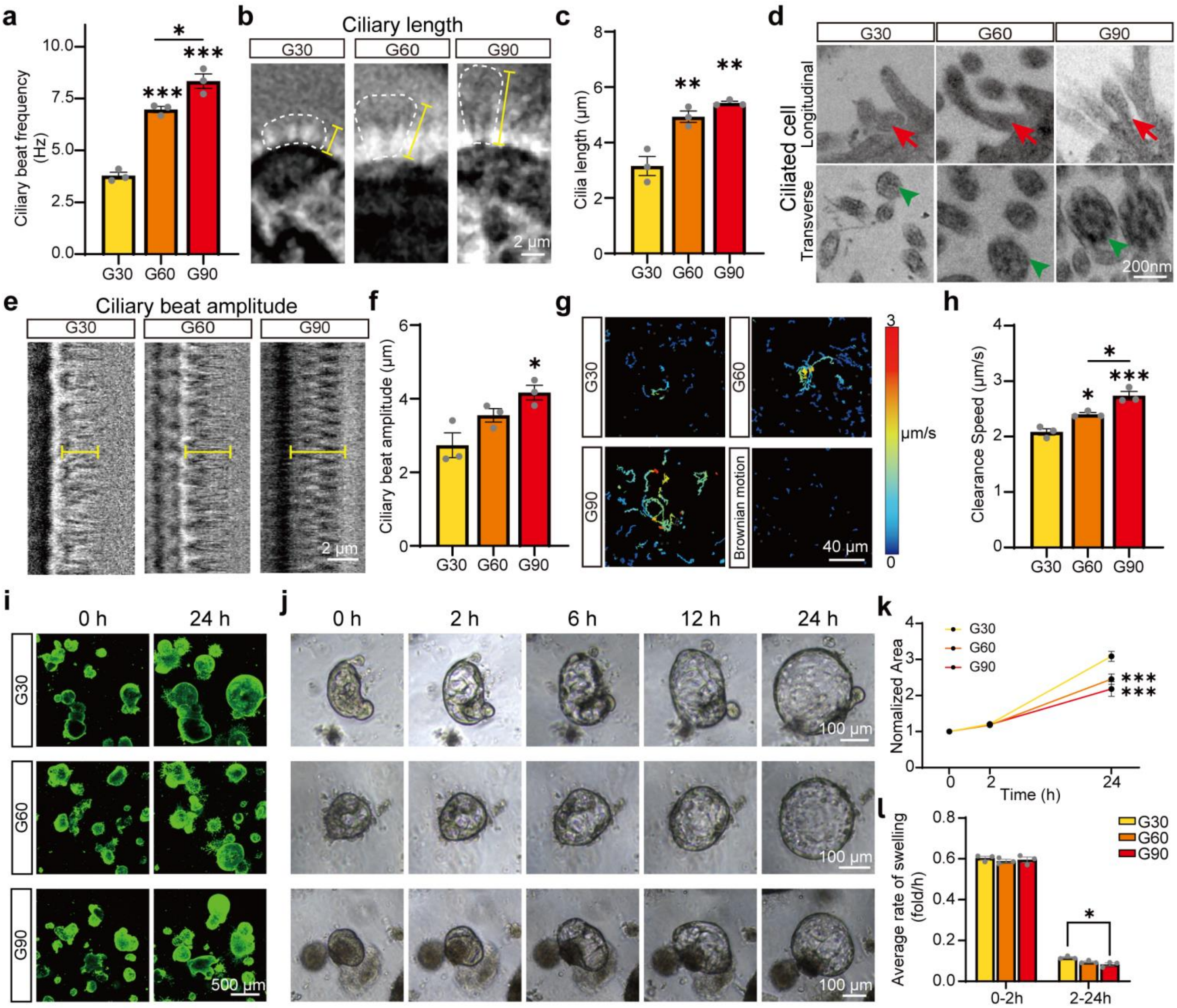

Figure 3. Region-specific differences in ciliary function and forskolin-induced swelling of hAWOs differentiated under varying ECM stiffness.

a Quantification of average ciliary beat frequency (CBF) in hAWOs. **b** Representative images of ciliary captured under bright field, white dashed box denotes the ciliary region, scale bar: 2 μm. **c** Quantification of ciliary lengths of G30-, G60- and G90-hAWOs. **d** TEM images of ciliated cells

under G30, G60, and G90 in longitudinal sections (red arrow) and in transverse sections (green triangle). **e** Analysis of ciliary beat amplitude (CBA) based on kymograph span (Scale bar: 2 μm). **f** Quantification of CBA of G30-, G60- and G90-hAWOs. **g** Stacked images of the fluorescent beads placed in the culture medium with hAWOs and Brownian motion of the beads without exposure to the hAWOs. The videos were recorded for 20 seconds. Color spectrum reflected time course. **h** The clearance speed of hAWOs calculated from at least 30 trajectories of the fluorescent beads per sample in each hAWOs with three independent replicates. **i** Fluorescence microscopy of live hAWOs (calcein green staining) at 0 h and 24 h after forskolin treatment. Scale bar: 500 μm. **j** Live imaging of hAWOs recorded at 0, 2, 6, 12, and 24 h after forskolin treatment. **k** Quantification of normalized organoid swelling area at 0, 2, and 24 h post-forskolin treatment. The bright-field area of each well at 0 h was set to 1. **l** The average rate of swelling was calculated for the first 2 hours and substitute 22 hours to reflect instant and long-term response.
Data are presented as mean ± SEM. Statistical analyses were performed using one-way ANOVA or two-way ANOVA with Tukey's multiple comparison test. *$p < 0.05$, **$p < 0.01$, ***$p < 0.001$.

"**Increased Stiffness Promotes hALO Functional Maturation and Epithelial Integrity**

Given that the key physiological function of the alveolus is surfactant production and respiration, we next performed established functional assays to evaluate AT2 surfactant maturation and AT1 epithelial integrity. Surfactant biogenesis in AT2 cells is tightly coupled to lipid handling and the endolysosomal-lamellar body (LB) axis, in which large amounts of phospholipids (e.g., DPPC) are synthesized, trafficked through acidic vesicular compartments, and packaged into LBs for secretion[47, 48]. β-BODIPY (neutral lipid) and LysoTracker (acidic endolysosomal compartments) staining are powerful for studying lipid droplets and lysosomal activity. We found that AT2 cells in G60 exhibited increased colocalization of β-BODIPY and LysoTracker, indicating enhanced surfactant metabolism (Fig. 6a). Furthermore, we further validated LB maturation using ABCA3, a transmembrane phospholipid transporter and a well-established marker of mature LBs. IF quantification demonstrated a higher proportion of ABCA3+ cells in G60 (51.04 ± 9.56%) and G90 (31.57 ± 0.76%) compared with G30 (15.75 ± 2.10%) (Fig. 6b, c). Moreover, ABCA3 and SFTPB co-staining revealed an increased fraction of ABCA3+SFTPB+ double-positive cells in G60, indicating more mature AT2 surfactant-associated function under intermediate-to-high stiffness (Fig. 6d). ......The integrity of the alveolar epithelial barrier is primarily maintained by intercellular junctions (eg. tight junctions) between alveolar epithelial cells48. AT1 in G90 displayed more elongated shapes and more tightly integrated intercellular junctions than those in G30 and G60 did (Fig. 6f, yellow arrow). The presence of elongated AT1-like morphology and well-formed intercellular junctions is morphologically consistent with functional barrier-forming alveolar epithelium.

Collectively, these results demonstrate that stiffness tuning enhanced surfactant biogenesis and LB

maturation in AT2-enriched hALOs (G60) and strengthened AT1-associated epithelial integrity in AT1-dominant hALOs (G90), supporting the generation of more *in vivo*-like mature alveolar organoids under higher mechanical cues. If the goal is to obtain more alveolar stem cells for transplantation or cell banking, G60 may represent the optimal stiffness, whereas if the model aims to study AT1 integrity or AT2-to-AT1 transdifferentiation, G90 would be a better choice.

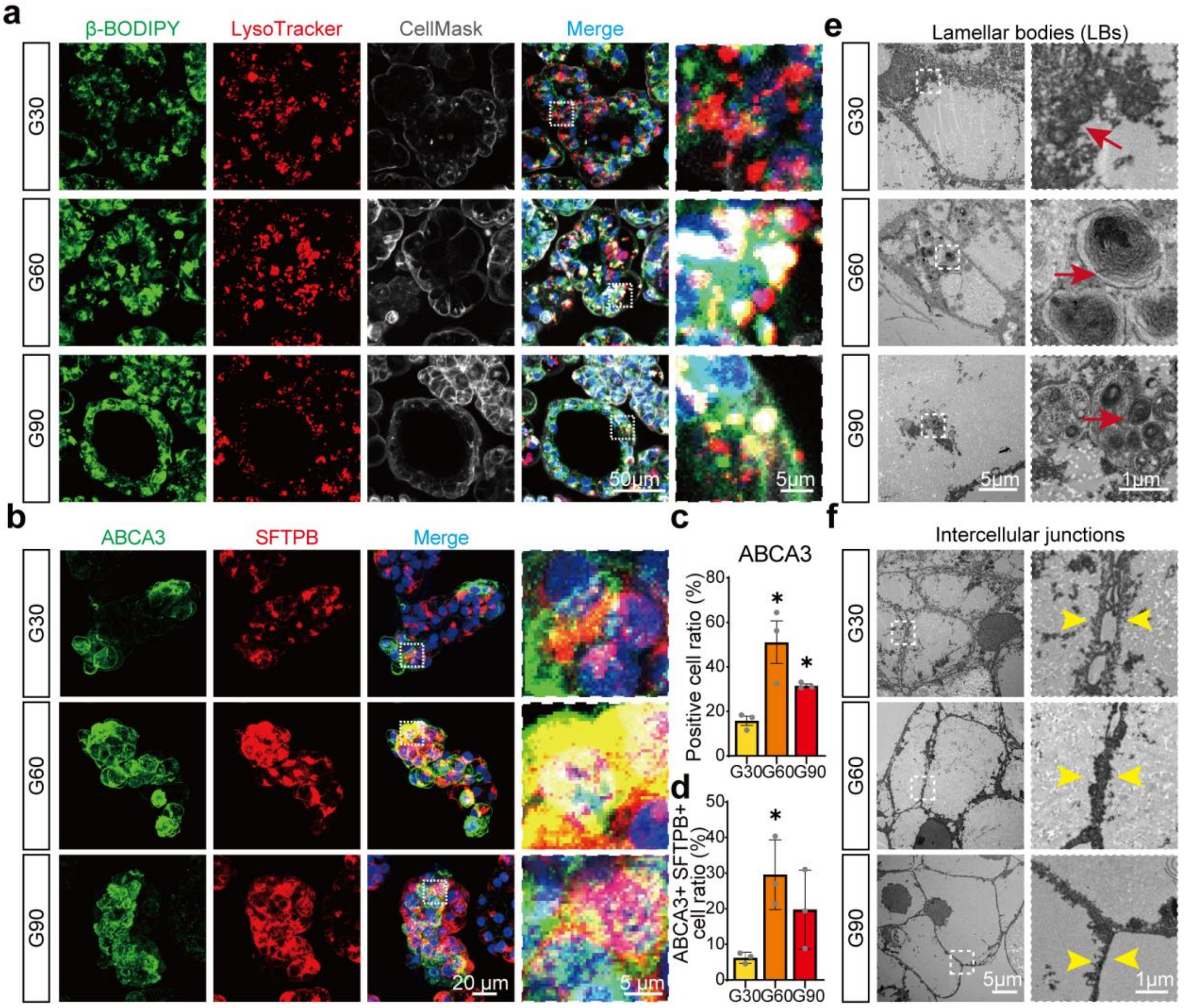

Figure 6. Functional maturation and epithelial integrity of hALOs under varying stiffness. a Live-cell confocal imaging of lipid metabolism and surfactant activity in hALOs cultured in G30, G60, and G90 hydrogels. β-BODIPY (green) labels neutral lipids, LysoTracker (red) indicates acidic organelles, and CellMask (white) labels the cell membrane. Merged images and magnified insets highlight colocalization of lipid droplets and lysosomes, indicating active lipid metabolism in AT2 cells under intermediate stiffness conditions. **b** IF staining of SFTPB and ABCA3, magnified insets highlight colocalization of $ABCA3^{+}SFTPB^{+}$. **c-d** Quantification of $ABCA3^{+}$ and $ABCA3^{+}SFTPB^{+}$ cell ratio, respectively. **e** TEM images demonstrating the presence of lamellar bodies (LBs) (red arrow) in AT2 cells, LBs with organized layers appear in G60 and G90. **f** TEM images illustrating the intercellular junction (yellow arrow) in AT1 cells, the intercellular space becomes smaller with increased stiffness.

*$p < 0.05$, analyzed by paired test or one-way ANOVA with Tukey's multiple comparisons test (n=3).

The experimental methods are detailed in the "Methods" section of the revised manuscript (Page 21)

that:

“Measurement of the ciliary beat frequency, ciliary beat amplitude and mucociliary transport

To analyze ciliary beat frequency (CBF), hAWOs (>30 days in hAWOs medium) were released from GelMA hydrogels (collagenase II as described “Differentiation of LPCs into human lung organoids (hAWOs or hALOs)” section) and transferred to glass-bottom confocal dishes. For measuring the CBF and CBA, 1,366 frames of bright-field images of the beating cilia were acquired at 130 frames per second (fps) on a high-speed confocal imaging system (Andor Dragonfly 200). CBF was calculated using a fast Fourier transform (FFT) based analysis[42]. Briefly, the regions of interest (ROIs) (2 × 2 μm) of each frame were selected using the Image J software program (National Institutes of Health). Then, the average brightness of each ROI and frame was calculated, followed by exporting and processing in R (v4.5.2) for FFT analysis. The ROIs of beating cilia by thresholding the CBF (3 and 35 Hz) were selected to minimize the effect of noise. At least three fields of view (FOVs) were recorded from each batch.

Ciliary length was measured using the freehand line tool in ImageJ by tracing individual cilia with clearly identifiable base and tip points[43]. For each batch, at least three randomly selected FOVs were analyzed, and at least ten individual cilia per FOV were measured. The average value was used for statistical analysis.

For the measurement of ciliary beat amplitude (CBA), individual cilia with clearly visible basal bodies and tips were first identified using ImageJ. Kymographs were then generated to visualize the spatiotemporal movement of the cilia. The displacement range of each beating cilium was manually traced on the kymographs, and the ciliary beat amplitude was defined as the maximum excursion distance between the base and tip positions during the beating cycle.

To assess mucociliary transport, fluorescent microspheres (Fluoresbrite®, 1 μm; Polysciences; 1:1000 dilution) were added to the culture medium. Fluorescence images were acquired using a high-speed spinning-disk confocal imaging system (Andor Dragonfly 200) equipped with a 20× oil-immersion objective. Videos were recorded at 20 fps, with 400 frames captured per acquisition. The trajectories and displacement of fluorescent beads driven by ciliary beating were automatically tracked using the TrackMate in ImageJ (Fiji). Particle transport velocity was calculated based on the

displacement of individual bead trajectories over time. Because ciliated regions in airway organoids are spatially localized rather than uniformly distributed across the entire field of view, particle trajectories were analyzed specifically within regions exhibiting coordinated ciliary beating. This approach ensured that the measured particle movement reflected active mucociliary transport rather than passive diffusion. Particles with extremely low velocities were considered to represent random Brownian diffusion and were excluded from the analysis. Brownian motion was used as a negative control for mucociliary transport analysis following previously described approaches [43]. For each experiment, more than five particle trajectories per organoid were analyzed to calculate the mucociliary transport index. During imaging, the samples were maintained in an incubator (37°C, 5% CO_2) equipped with the microscope.

Forskolin-Induced Swelling of Organoids

One day prior to the experiment, organoids were released from GelMA hydrogels as described above, resuspended in 20 µL of grwoth factor reduced Matrigel, and seeded into 48-well plates. After Matrigel solidification, 100 µL of AWOs culture medium (without 8Br-cAMP and IBMX) was added. For the swelling analysis, the organoids were incubated with 10 µM forskolin (Aladdin, Cat#66575-29-9) and 2 µM Calcein AM (Solarbio, Cat#CA1630) for 0-24 hours at 37°C, 5% CO_2. Images were acquired at 2 h, 6 h, 12 h, and 24 h using a Bright field microscope (Nikon, ECLIPSE Ts2-FL). The area of organoids was analyzed using ImageJ software, and normalized relative to the average area at 0 h (before treatment). Images were again analyzed at 2 and 24 hr post-forskolin addition and the ratio of the post-swelling area to the original area was calculated. At least 5 individual organoids were measured for each sample with 3 replicates.”

3. RGD density across GelMA with different methacrylation

The statement that RGD density is the same in G30, G60, and G90 is inaccurate. While the sequence-level RGD count may be similar, differing degrees of methacrylation alter the GelMA network structure and thus the degree of bioavailable/accessible RGD for cells.

We thank the reviewer for pointing out this issue.

In our study, the statement of "RGD density is the same" in G30, G60 and G90 was based on the previous publication (Li et al., Polymers, 2016)[1] that: "...The results indicated that the arginine content in Gel and GelMA macromers was almost the same, which suggests that the arginine should be not involved in the reaction even at a high DoF (Fig. 2). Therefore, the RGD density in all the GelMA hydrogels prepared from the three types of GelMA macromers should be kept at the same levels, which was important to exclude the influence of different density of active RGD motifs."

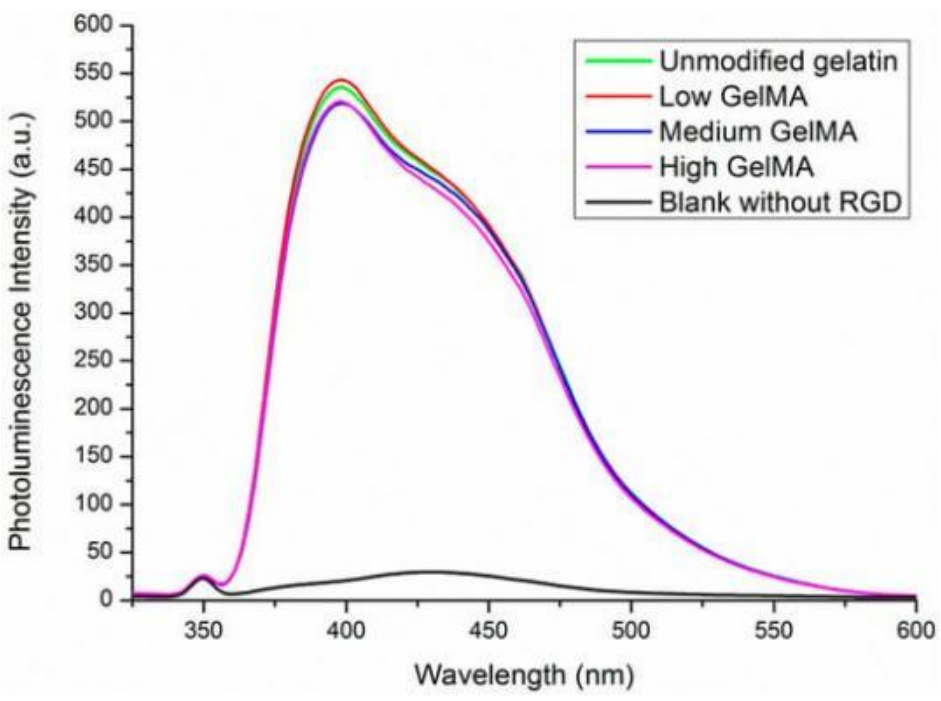

Fig. 2 from Li et al., Polymers, 2016

"The degree of functionalization (DoF, %, percentage of methacryloyl in their study) of low, medium and high GelMA is 25%, 50% and 90%, respectively."

Table 1. The DoF of Low GelMA, Medium GelMA, and High GelMA.

Sample	Feed Ratio of Gel (g)/MA (mL)	DoF (%)
Low GelMA	5/0.2	25.8 ± 0.7
Medium GelMA	5/1.0	52.5 ± 1.2
High GelMA	5/5.0	91.7 ± 1.4

Table 1 from Li et al., Polymers, 2016

We acknowledge the reviewer's concern that although the sequence-level ligand content may be similar, differences in hydrogel network structure and crosslinking can affect the bioavailability and accessibility of adhesive motifs to cells. To avoid misunderstanding, we have replaced "RGD density" by "GelMA concentration" and now described in the revised manuscript (page 7) that:

"The stiffness of the GelMA hydrogels was tuned exclusively by varying the degree of methacryloyl (MA) substitution but keep the GelMA concentration the same. ... Previous studies similarly reported no significant effect of stiffness on proliferation when GelMA concentration keeps the same[38]."

4. Thickness vs. stiffness inconsistency

You state thicker GelMA leads to higher stiffness; however, in the rebuttal Figure 1, panel i (2.5 mm) shows G90 at ~22–23 kPa, whereas panel l (5 mm) shows G90 at ~17–18 kPa. Please clarify this. It is unclear why the stiffness, an inherent property of a material, would change when measuring hydrogels of differing thicknesses. An explanation as to why these data changed is necessary.

We apologize for the error and have corrected it accordingly.

To determine whether sample thickness affected the measured stiffness of G90 hydrogels, we performed a time-dependent compression test on G90 hydrogels with thicknesses of 2.5 mm and 5.0 mm. As expected, the compressive modulus increased with increasing crosslinking time. However, no significant difference was observed between the two thicknesses at the tested time (Rebuttal Figure 2).

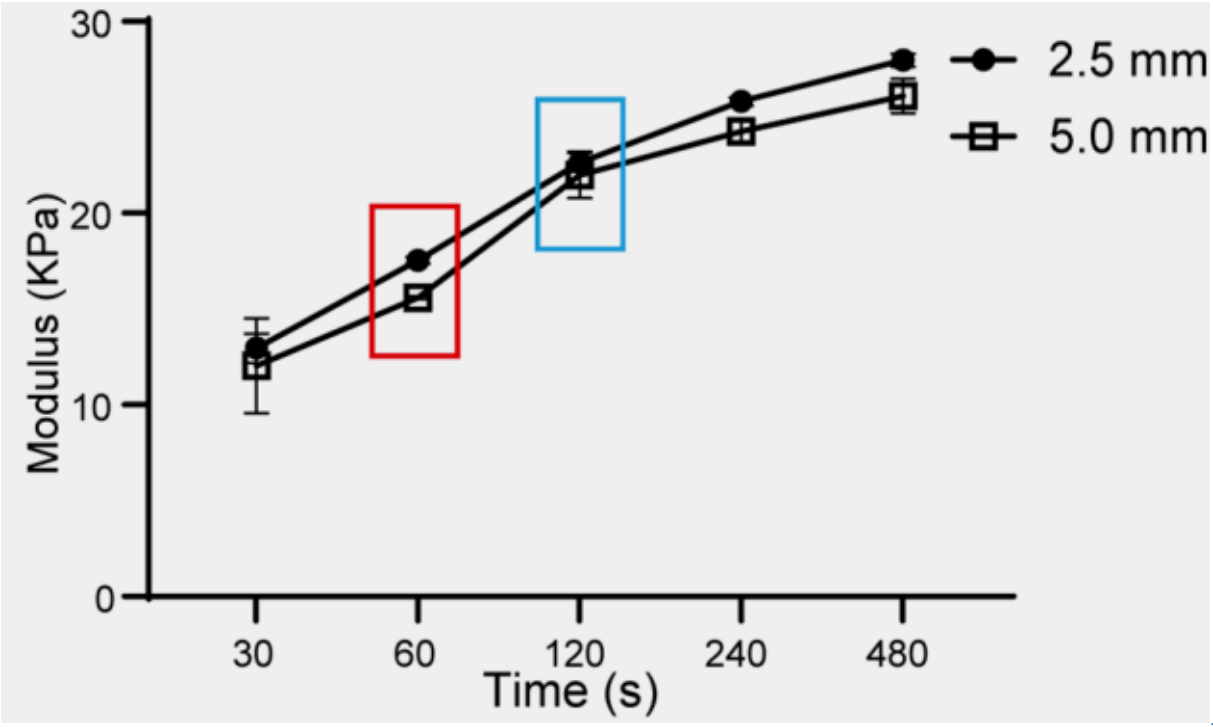

Rebuttal Figure 2

At 60 s of UV crosslinking, the Young's modulus was 15.60 ± 0.05 kPa for the 5.0 mm-G90 and 17.53 ± 0.10 kPa for the 2.5 mm-G90 (red box, P = 0.12). At 120 s, the compressive modulus reached 21.99 ± 0.70 kPa for the 5.0 mm-G90 and 22.65 ± 0.28 kPa for the 2.5 mm-G90 (blue box, P = 0.94). The experiments have been repeated three times. The G90 hydrogel (5.0 mm in thickness) crosslinked under UV light for 120 seconds consistently showed an average stiffness of approximately 22 kPa (Rebuttal Figure 2), which is different from panel l (5 mm) shows G90 at ~17–18 kPa (Rebuttal Figure 1*l*).

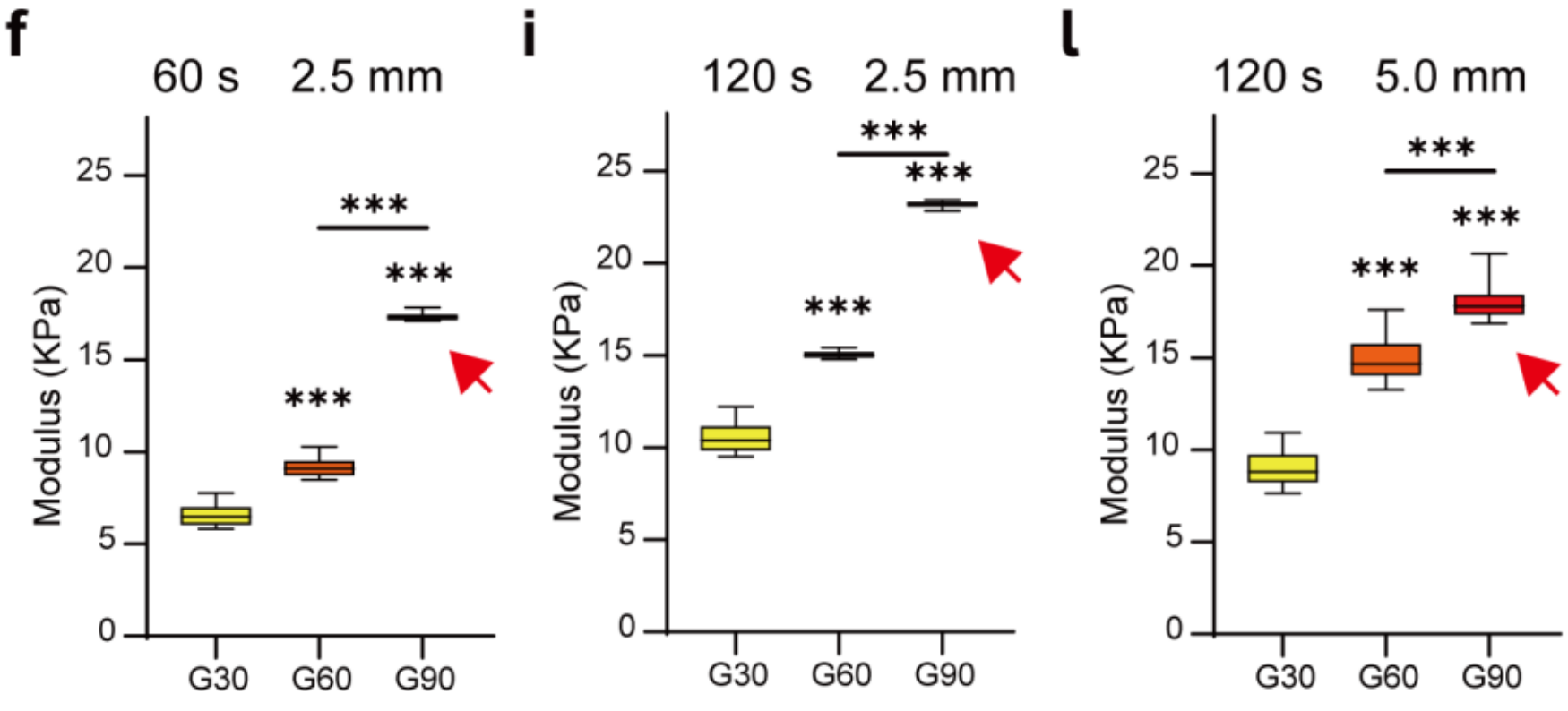

Rebuttal Figure 1

After revisiting the the original datasets and re-examined the experimental records, we found the G90 with a 5mm thickness had been inadvertently crosslinked for 60 s rather than 120 s. This explains why the modulus was lower than that of G90 hydrogel with 2.5 mm thickness crosslinked for 120 s (Rebuttal Figure 1i), while comparable to G90 hydrogel with 2.5 mm thickness and crosslinked for 60 s (Rebuttal Figure 1f).

After correction, these results indicate that there is no statistically significant difference in stiffness between the two thicknesses (2.5 mm *vs* 5 mm), which is consistent with the physical expectation that hydrogel stiffness should be independent of sample thickness.

5. Targeted airway region

Which small-airway compartment is the model intended to mimic? Note that cartilage is present in the trachea and bronchi but absent from bronchioles (small airways). Please clarify anatomical scope and relevance.

1) We thank the reviewer for raising this important question regarding the anatomical scope of our hAWOs model.

To benchmark *in vitro* hPSC induced airway organoids with native human fetal lung airway epithelium, Miller et al. (Miller et al., Dev Cell, 2020)[2] generated single-cell transcriptomic datasets from both airway organoids and developing human fetal lung epithelium including **trachea, small airway and distal lung (Fig. 3A, B)**. Comparative analysis revealed that *in vitro* derived organoids recapitulated molecular features of their fetal counterparts.

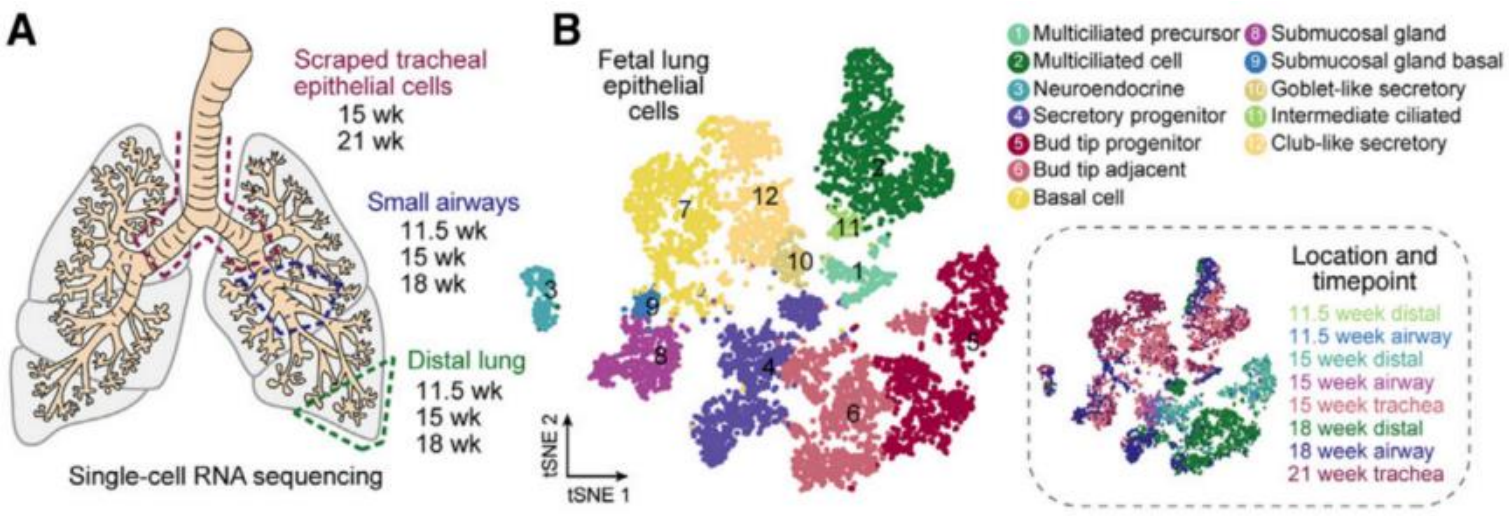

(Fig. 3A, B from Miller et al., Developmental Cell, 2020)

Following this strategy, we assessed the anatomical relevance of our stiffness-guided hAWOs by performing a reference-based mapping and annotation analysis using the developing human airway atlas from Miller et al and prepared Fig.S8 to answer this anatomical relevance of our model. Our results indicate that our model predominantly represents conducting airway epithelium spanning the trachea to bronchiolar (small airway) compartments. We now clarify the anatomical scope of our hAWOs model in the “Results” section (page 10) of the revised manuscript that:

"To benchmark anatomical relevance of our hAWOs cultured under various stiffness with native human fetal lung airway epithelium, we performed a comparative analysis referenced the datasets of the developing fetal airway atlas spanning the trachea, small airways and distal lung (Supplementary Fig. 8a)[46]. The reference map generated from trachea, small airway and distal lung regions anatomically defined fetal epithelial populations (Supplementary Fig. 8b). We then mapped our scRNA-seq data from hAWOs with different stiffness onto the referenced map. The integrated map of our stiffness-guided hAWOs indicated that the majority of cells correspond to epithelial populations of the trachea (35273 cells) and small airways (29457 cells), with only a minor fraction mapping to distal lung lineages (4403 cells) (Supplementary Fig. 8c). Finally, we quantitatively compared the predicted tissue position labels of organoids across different stiffness groups which also indicated more trachea-like G90-hAWOs, more small airway-like-G30-hAWOs (Supplementary Fig. 8d). Together, these results indicate that our ECM-stiffness-guided hAWOs primarily model the conducting airway epithelium spanning the trachea to small-airway regions."

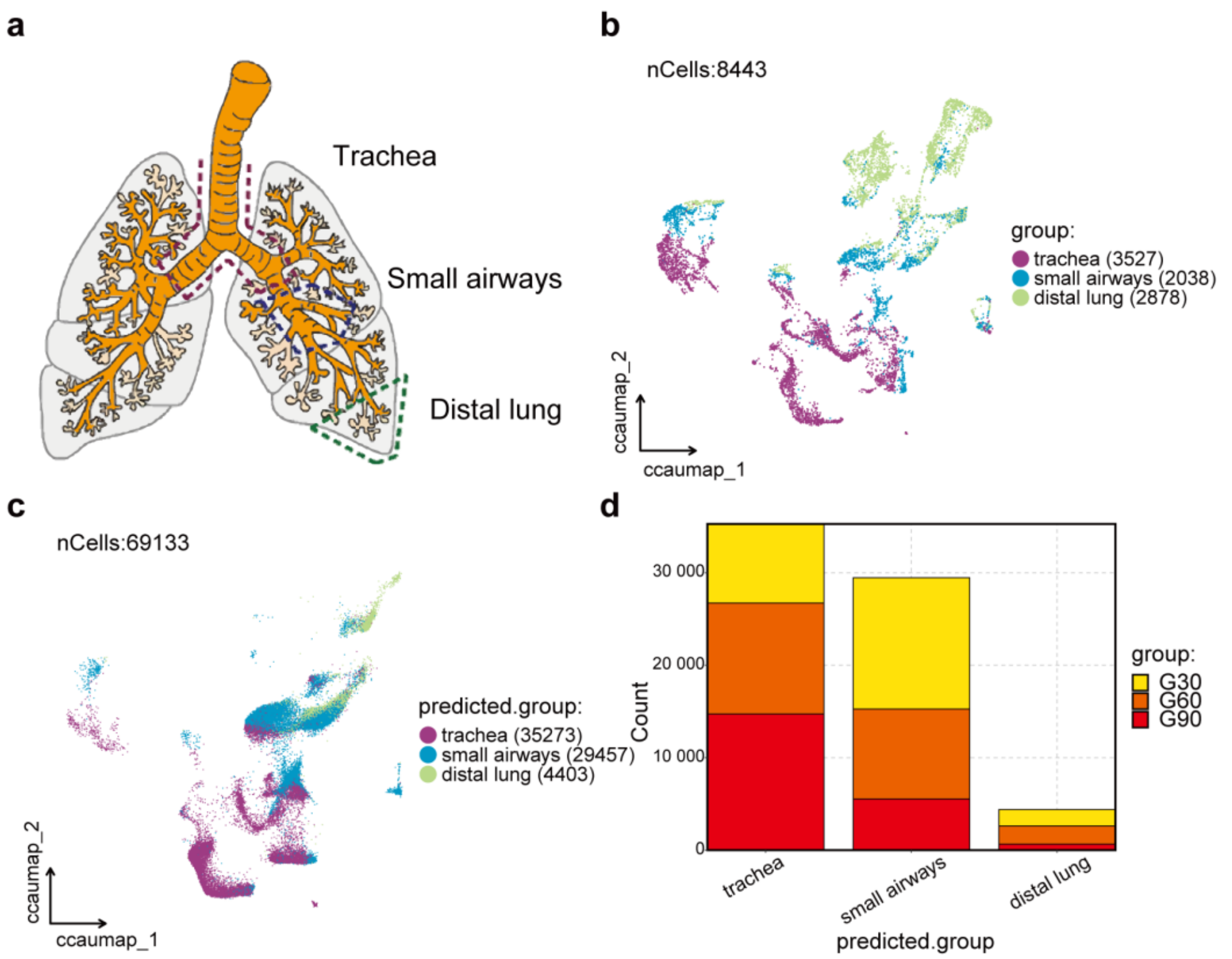

Supplementary Fig. 8. In vitro derived AWOs under stiff-to-soft hydrogels recapitulate molecular features of native fetal trachea and small airway cells.

a Anatomical diagram of the trachea, small airways and distal lung of the fetal regional airway epithelium. b UMAP of the re-processed data of E-MTAB-8221, labeled by original airway regions of trachea (purple), small airways (blue) and distal lung (green). c Integrated UMAP showing our stiffness-guided hAWOs projected on the predicted regions of the published fetal airway map. d Bar chart shows the numbers of epithelial cells from different stiffness-guided hAWOs across the predicted airway regions of trachea, small airways and distal lung.

However, we do not claim that our *in vitro* hAWOs are absolutely equivalent to any single *in vivo* anatomical compartment. As noted by Miller *et al.*, "the transcriptional signature of an *in vitro*-derived cell is unlikely to be absolutely equivalent to an in *vivo* cell type, because the *in vitro* and *in vivo* environments are inherently different." We therefore use this mapping analysis to define the closest anatomical relevance and regional bias of our model, rather than to claim exact one-to-one equivalence.

We now summarize this point in the "Discussion" section of the revised manuscript (page 16) that:

"Although mimetic ECM stiffness along the proximal-to-distal axis biases hAWOs toward distinct proximal-to-small-airway identities, modulation of ECM stiffness alone is not sufficient to fully replicate *in vivo* airway segment."

2) We appreciate reviewer's comment and acknowledge the cartilage distribution in airway is "present in the trachea and bronchi but absent from bronchioles (small airways)". We now depict in "Introduction" section (page 3) of the revised manuscript that:

"The conducting airway is a hierarchical, tree-like branching structure that extends from the proximal trachea to the distal bronchioles along the proximal–distal axis[13]. ...Mechanical rigidity **of trachea and bronchi** is reinforced by cartilage rings and smooth muscle layers, resulting in a thick and stiff ECM[13]. As the airway tree branches from larger conducting airways to smaller distal bronchioles, the ECM gradually becomes thinner and softer, accompanied by the loss of cartilage support[14]....."

We also corrected the illustration of schematic airway (Supplementary Fig. 14) as below:
The cartilage is present in the trachea and bronchi and absent from bronchiole.

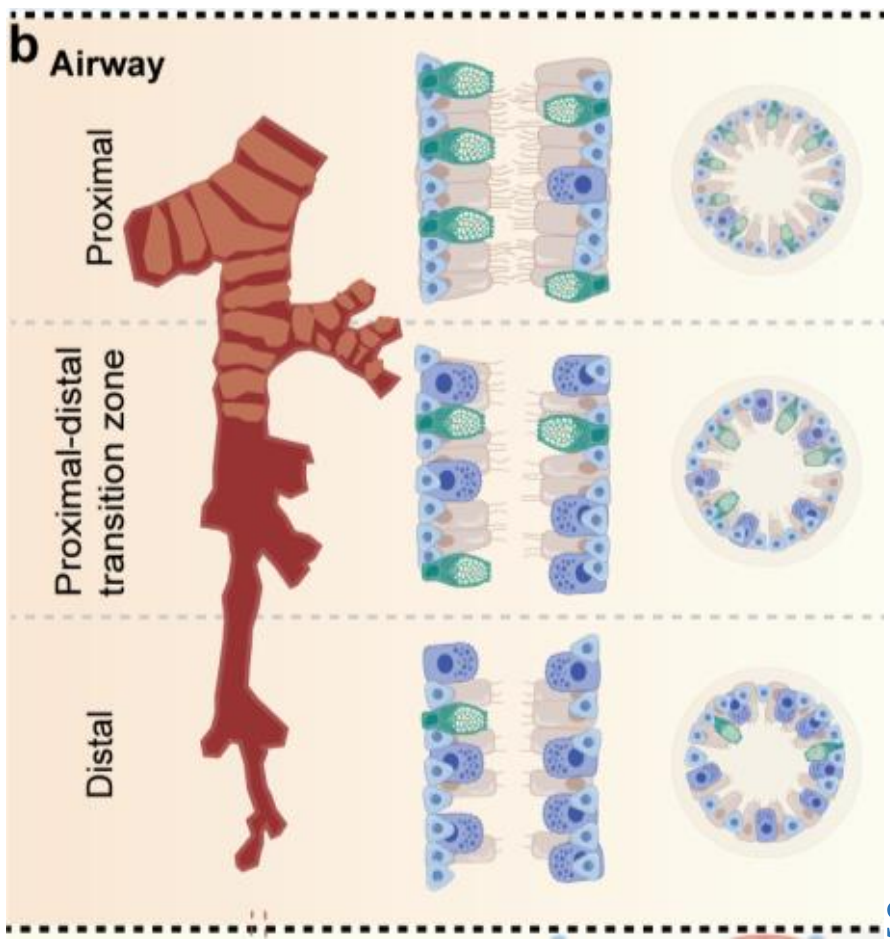

Supplementary Fig. 14b

6. Mechanical cues beyond stiffness

In your response to Question 6 (p.13), you note that elasticity and loading contribute to in vivo mechanosignaling in alveoli. However, the experimental design in this study did not incorporate elasticity/loading but only adjusted stiffness. Therefore the relevance of this response is not immediately obvious. This is not convincing for the conclusion and discussion.

We sincerely thank the reviewer for giving us the opportunity to clarify this point again and apologize that our previous response did not sufficiently address the concern. We acknowledge that stiffness is not equivalent to elasticity or mechanical loading, although all of them belong to mechanical forces. Our intention was not to treat these distinct mechanical inputs as the same, but rather to acknowledge that their downstream mechanotransduction pathways may partially overlap.

Previous studies on the role of mechanical forces in alveolar epithelial cell fates have shown that:

1) Respiratory force activates Hippo pathway defines AT1 fate:

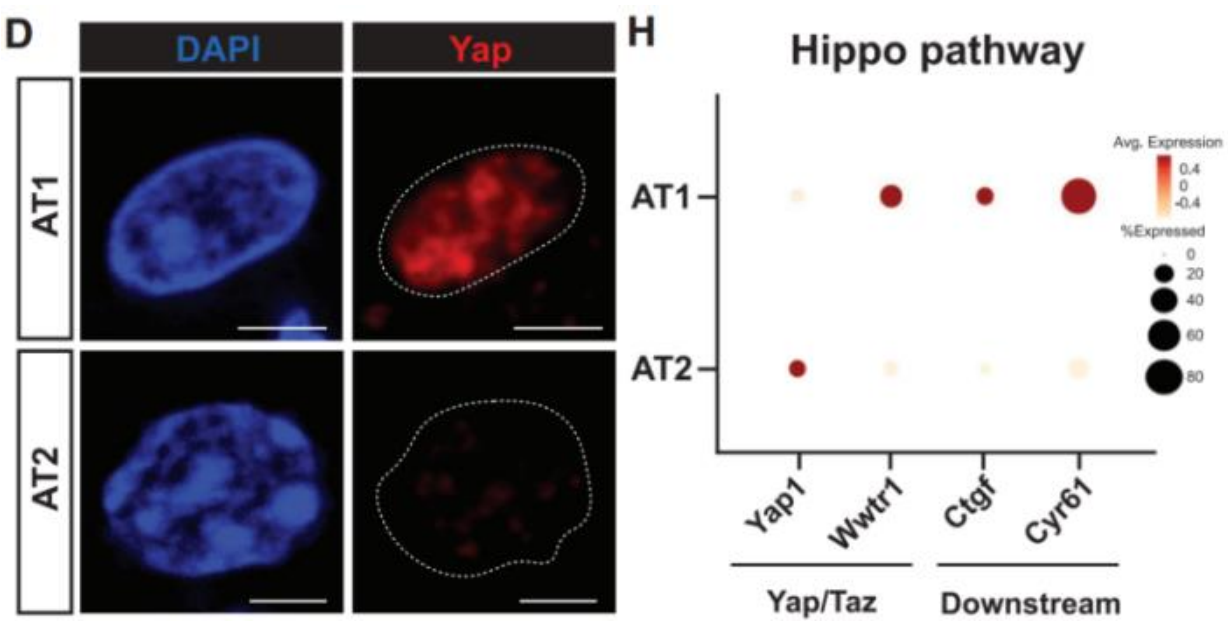

Fig. 1 D & H, Shiraishi et al., Cell, 2023

"Biophysical forces mediated by respiration is the key of AT1 generation with activation of YAP pathway (Fig. 1D and H) (Shiraishi et al., Cell, 2023)[3]." This result is consistent with our finding that ECM stiffness promotes AT1 cell fate through activation of YAP (Fig. 5f-j).

2) Nuclear YAP activation is sufficient to drive an AT1 transcriptomic shift:

"AT1^{YAP5SA} cells had higher frequency of expression of YAP downstream targets, ANKRD1, CYR61, and CTGF, consistent with forced overexpression of activated nuclear YAP (Figure 3H). Taken together, the results suggest that overexpression of nuclear YAP drives a transcriptomic shift from iAT2 to iAT1-like cells in a cell-autonomous manner (Burgess et al., Cell Stem Cell, 2024)[4] ."

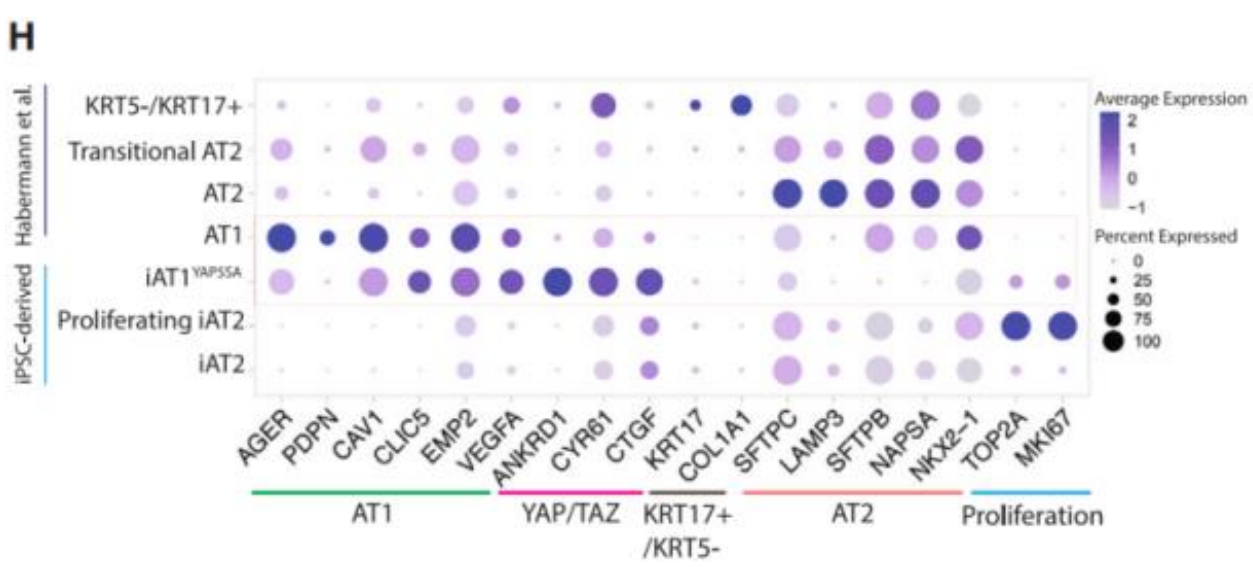

Fig. 3H, Burgess et al., Cell Stem Cell, 2024

This result is consistent with our finding that addition of the small molecules (TRULI) of nuclear YAP activator significantly increased the expression of AT1 related markers independent of ECM

stiffness stimulation (Fig. 7n, q, r).

3) Taken together, different mechanical cues, including ECM stiffness, elasticity and mechanical loading, all of these may act distinct upstream inputs, but the downstream mechanotransduction pathways often overlap, such as FAK, Hippo, integrin signaling (Kindberg et al., Curr Opin Cell Biol, 2020)[5]. In our study, the most prominent mechanism associated with ECM stiffness directed AT2 and AT1 cell fate specification was enrichment of Hippo pathway, which is consistent with previous publications showing that respiratory forces or other mechanical stimuli can direct alveolar epithelial cell fate through similar downstream signaling pathways.

We have revised the related discussion to avoid any unintended impression that stiffness is equivalent to elasticity or mechanical loading. We now revised the description in the "Introduction" section of the revised manuscript (Page 4) that:

"After birth, biomechanical forces generated by respiratory motion activate Hippo-YAP/TAZ signaling pathway for maintaining AT1 cell identity, whereas the loss of biophysical forces can drive AT1 reprogramming toward AT2[18, 19]. An appropriate level of ECM elasticity is also critical for secondary septation (the formation of mature alveoli) and coordination with respiratory motion through regulation of downstream pathways, among which YAP/TAZ mediated mechanical tension-induced pulmonary alveolar regeneration[20]. Overexpression of activated nuclear YAP is sufficient to drive transcriptomic shift of iAT2 to iAT1-like cells, without ECM mechanical forces involvement[21]. Although mechanical force including ECM stiffness, elasticity, loading, all of these may act differently, the underlying mechanism may overlap (e.g. FAK, Hippo, integrin signaling pathways)[22]."

We have summarized in the discussion section (page 17) that:" Our study indicates that ECM stiffness modulates lung development by regulating the Hippo pathway, which in turn regulates the specification of lung progenitors and the differentiation of airway and alveolar epithelial cell fates (Fig. 1, 3 and 5)." to support the mechanical stiffness regulates epithelial cell fates primarily through Hippo signaling pathway.

7. The discussion points relating to cortical organoids for assessing optimal neural development do not provide substantive support for the points related to this manuscript. Similarly, pleura-to-alveolar septa stiffness gradients refer to gradients within the alveolar compartment, and thus are difficult to align with the current thesis that there is an altered response to stiffness between the airway and alveolar compartments.

We sincerely thank the reviewer for this critical comment.

Our intention in citing the cortical organoids study (Cadena et al., Adv Healthc Mater, 2024)[6] was to highlight a broader concept that "organoid differentiation may not always be optimal at the soft matrix within the physiological range of the native tissue. In Cadena et al.'s study:

1) They found cortical organoids showed improved development in relatively higher stiffness (from 7-9 kPa) compared with lower stiffness (from 0.1-3 kPa), despite the reported "stiffness range of 0.1 to 10 kPa of the developing human fetal cortex."

Similarly, lung ECM stiffness ranges from 0.5-15 kPa and the reported fetal lung stiffness is around 3.68-5 kPa [27, 68]. We prepared GelMA hydrogel with the stiffness range of 2.99 to 11.31 kPa and found alveolar epithelial differentiation and maturation were enhanced under the intermediate-to-high stiffness (6.22 to 11.31 kPa). We have depicted in the discussion (page 17) that: "GelMA hydrogels (G30, G60 and G90) with stiffness ranging from 2.99 kPa to 11.31 kPa (Supplementary Fig. 1c), covering reported lung regional ECM values (0.5-15 kPa) and fetal lung stiffness (3.68-5 kPa)[27,68]" "distal alveolar cells did not favor soft hydrogel as we proposed. Instead, intermediate-to-high stiffness (6.22-11.31kPa) enhanced alveolar cells maturation and AT2-to-AT1 transition (Fig. 4)."

2) To explain the reason why cortical organoids prefer the scaffold with the higher stiffness, they discussed: "the stiffness of the developing skull (1-50 MPa) (Cadena et al., Adv Healthc Mater, 2024)[6] is orders of magnitude higher than that of the brain ECM, but little is known how this physical constraint influences neural developmental processes."

With regard to the discussion of external mechanical constraints, lung parenchyma is enclosed by a visceral pleura layer with MPa-scale tensile mechanics (Ramirez et al., Acta Biomaterialia, 2024)[7]. Therefore, our original intention was to raise the possibility that lung epithelial development may occur under multiscale mechanical influences, including tissue-level constraints beyond the local ECM. To avoid confusion, we have rephrased our discussion in the revised manuscript (page 17) that:

"A previous study reported that cortical organoids developed better in relatively stiffer scaffolds (7-9 kPa) than in softer scaffolds (0.1-3 kPa). The physical constraints imposed by the developing skull with orders of magnitude higher stiffness (1-50 MPa), may provide tissue-level constraints beyond the local ECM[69]. Similarly, the lung parenchyma is surrounded by the visceral pleura, whose tensile mechanics are orders of magnitude higher than those of the local epithelial ECM[41]. Therefore, lung

epithelial development may occur under multiscale mechanical influences, including tissue-level constraints beyond the local basement membrane and interstitial ECM.”

3)We acknowledge the gradient stiffness from proximal-to-distal airway is different from pleura-to-alveolar septa stiffness gradients. Our previous wording may unintentionally conflated these two spatial framework. To avoid the confusion, we have removed the description of “pleura-to-alveolar septa stiffness gradients” in our revised manuscript.

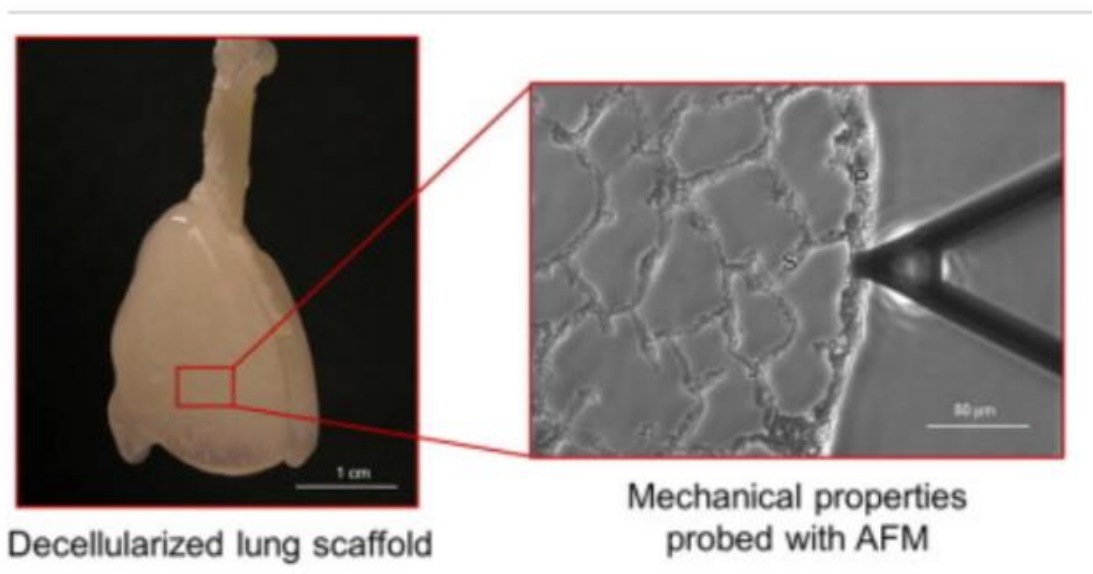

From the literature, Luque et al. (Luque et al., Acta Biomaterialia, 2013)[8] used AFM to identify local stiffness in alveolar wall segments, alveolar wall junctions and pleura regions, with the gradient stiffness ranging from 5.59 to 15.76 kPa.

	Alveolar segments	Alveolar junctions	Pleura
A (kPa)	5.59±3.39*	6.79±3.88*	15.76±13.70

Graphic abstract and table 1 from Luque et al., Acta Biomaterialia, 2013

We referenced Luque’s study to show that the intermediate-to-high stiffness (6.22 to 11.31 kPa) located between this range of the native lung alveolar compartment. We now describe in the “Discussion” section of the revised manuscript that:

“Luque et al used AFM to measure the local stiffness of lung ECM in different regions, including alveolar wall segments, alveolar wall junctions and pleura regions, with the stiffness incresed from 5.59 to 15.76 kPa[70]. Our results indicated that alveolar epithelial cells differentiated better under intermediate-to-high stiffness, which fall within the stiffness range reported for alveolar regions *in vivo*.”

8. scRNA-seq replication

With only one biological replicate, the scRNA-seq evidence is insufficient to draw any conclusions.

We thank the reviewer for pointing out this major methodological issue.

We agree that three biological replicates are important for drawing reliable conclusions. In response to the reviewer’s concern and to further strengthen the reproducibility and reliability of our data, we performed two additional independent biological replicates of the scRNA-seq experiments for both hAWOs and hALOs under three varying stiffness (G30, G60 and G90). We then integrated the three

replicates for reanalyze the data and regenerated the corresponding figures. Comparative analysis of these independent datasets confirmed consistent results, including similar distributions of epithelial subpopulations and reproducible trends in region-specific marker expression across stiffness conditions. Accordingly, we have revised and updated the Results, Methods, Figure panels and Figure legends with the new data and regenerated figures.

In the results, we have updated cell numbers in both the figure panels and the main text after integrating the three biological replicates of hAWOs under varying stiffness (page 9), as follows:

“Three independent biological replicates from each stiffness group (G30, G60 and G90) with a total of 71,179 cells were analyzed, and the datasets were integrated for scRNA-seq analysis and figure generation (Supplementary Fig. 6a).”

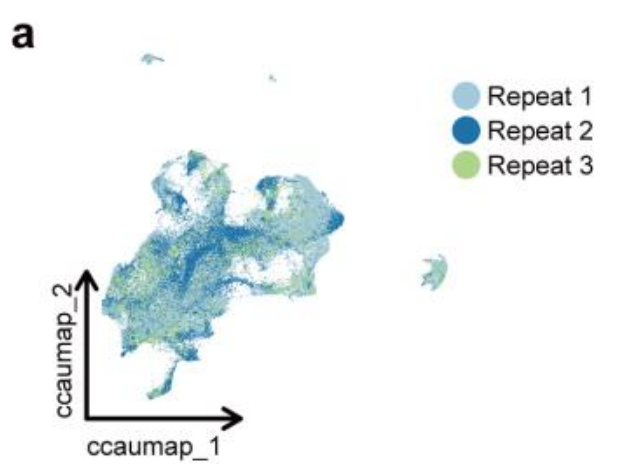

Supplementary Fig. 6 **a** UMAP of integrated scRNA-seq data from three independent biological replicates for each stiffness group, colored by replicate identity, showing consistent integration across replicates.

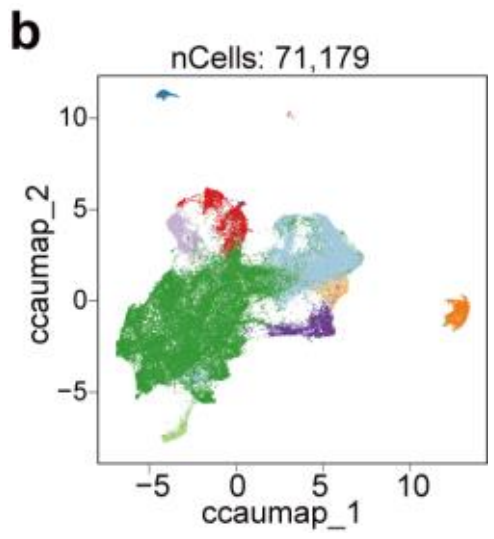

Fig. 4 **b** Uniform Manifold Approximation and Projection (UMAP) visualization of 71,179 cells from hAWOs differentiated under G30, G60 and G90 across three biological replicates, grouped into 10 distinct clusters based on curated cell type markers.

We also integrated the three independent biological replicates of hALOs (page 13), as follows:

“Among the total of 26,643 cells profiled from three independent biological replicates for each stiffness group (G30, G60 and G90),...”

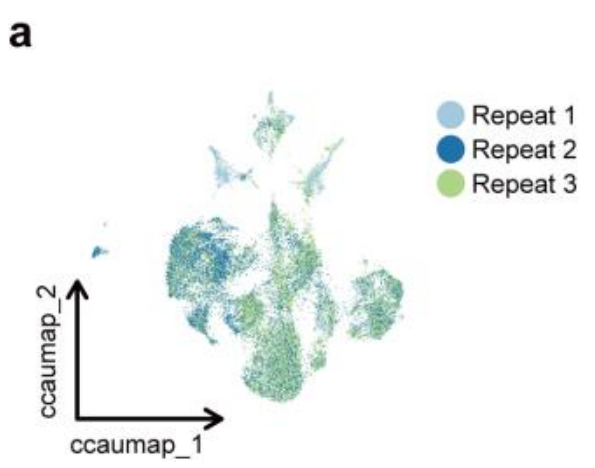

Supplementary Fig. 11 **a** UMAP of integrated scRNA-seq data from three independent biological replicates or each stiffness group, colored by replicate identity, showing consistent integration across replicates.

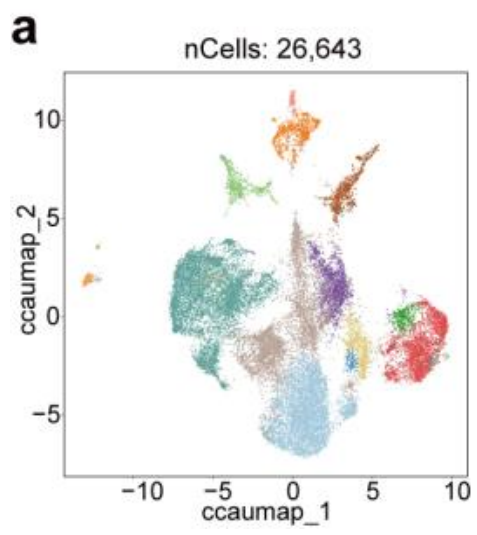

Fig. 7 **a** UMAP visualization of 26,643 cells from hALOs differentiated under G30, G60 and G90 across three independent biological replicates, showing 16 annotated cell types based on curated markers.

The main figures 4 (b-i) and 7 (a-f, k-m); Supplementary figures 6, 7, 11 and 12 of scRNA-seq have been updated with the new figure panel regenerated from the 3 biological replicates.

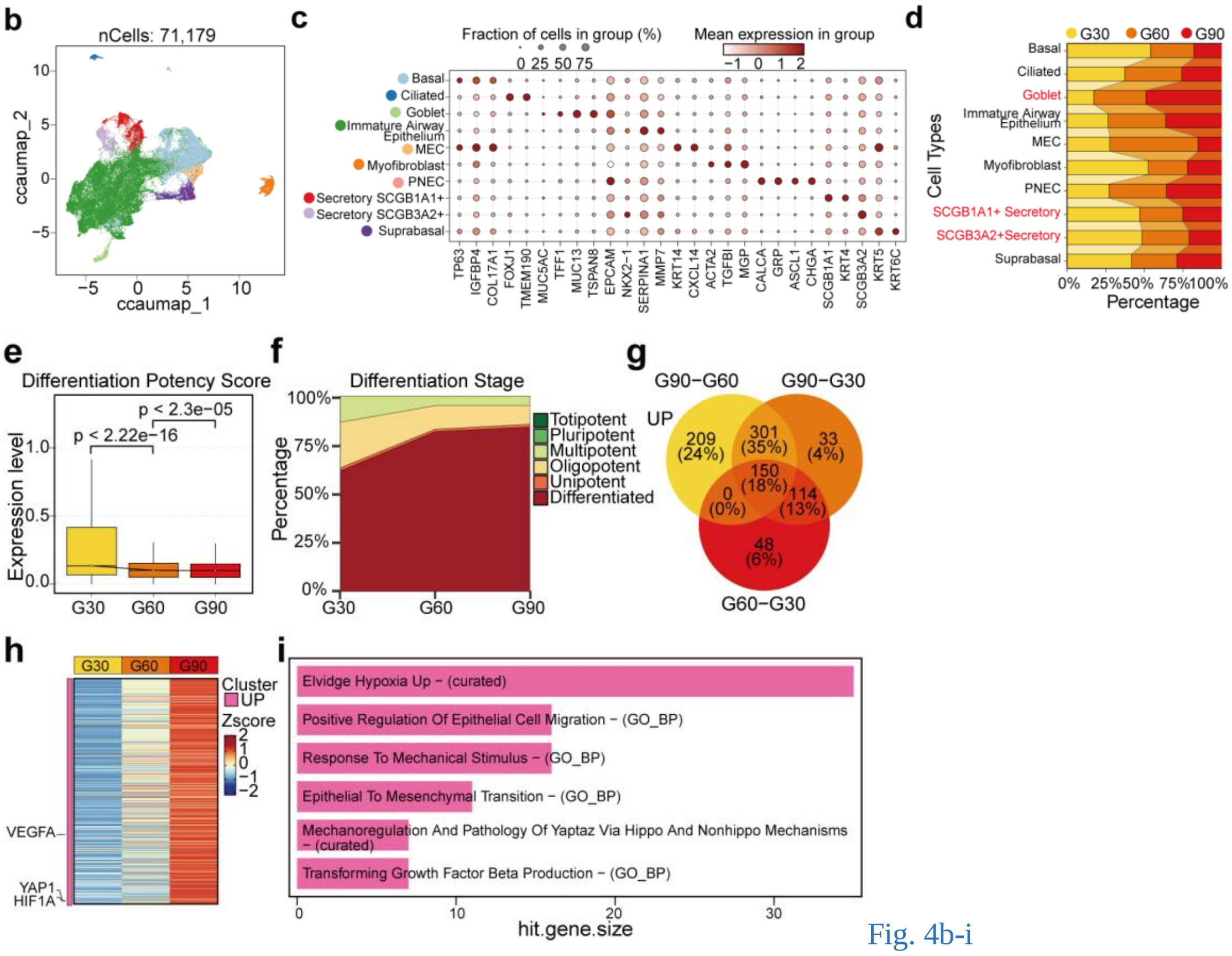

Fig. 4b-i

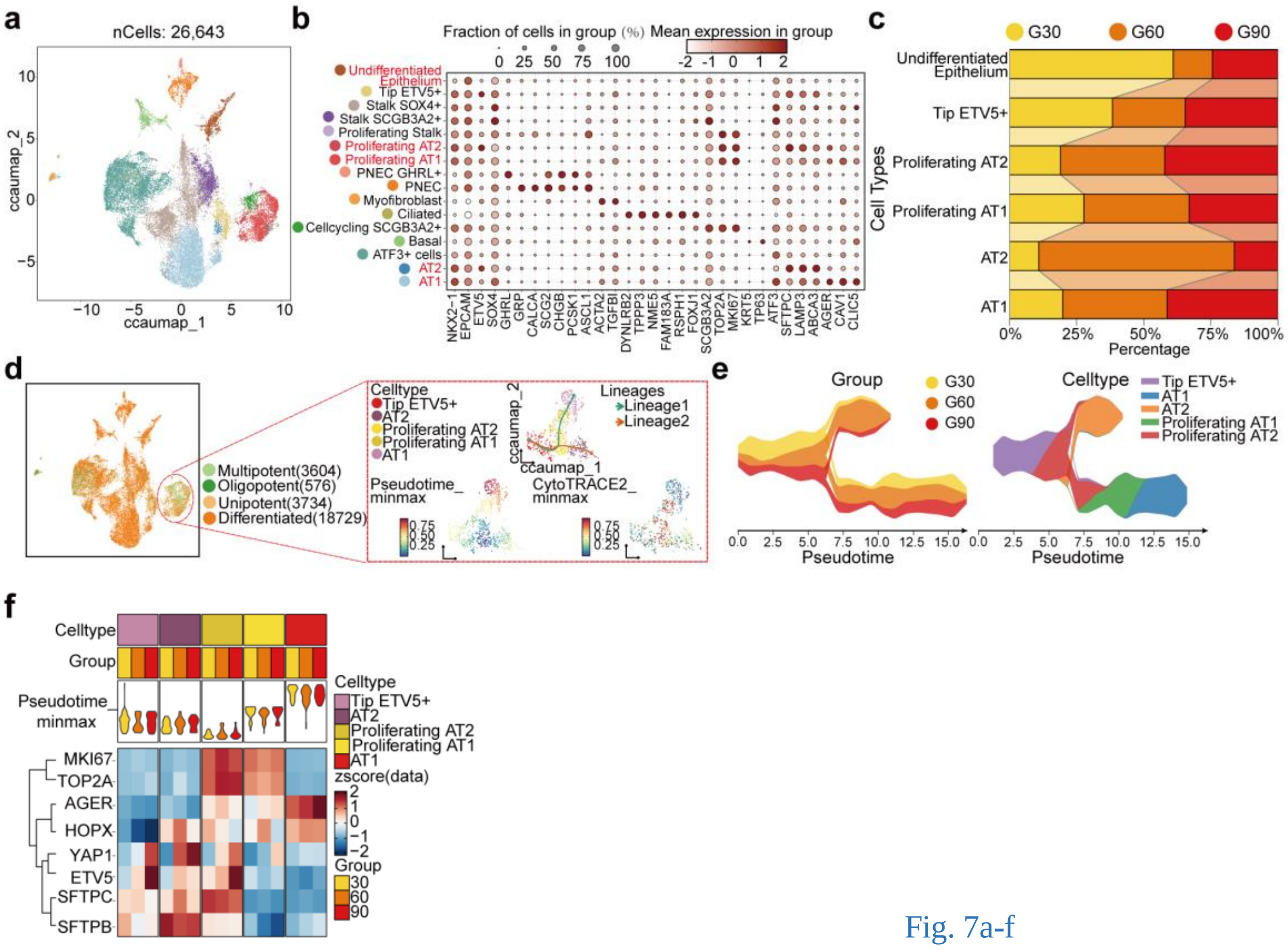

Fig. 7a-f

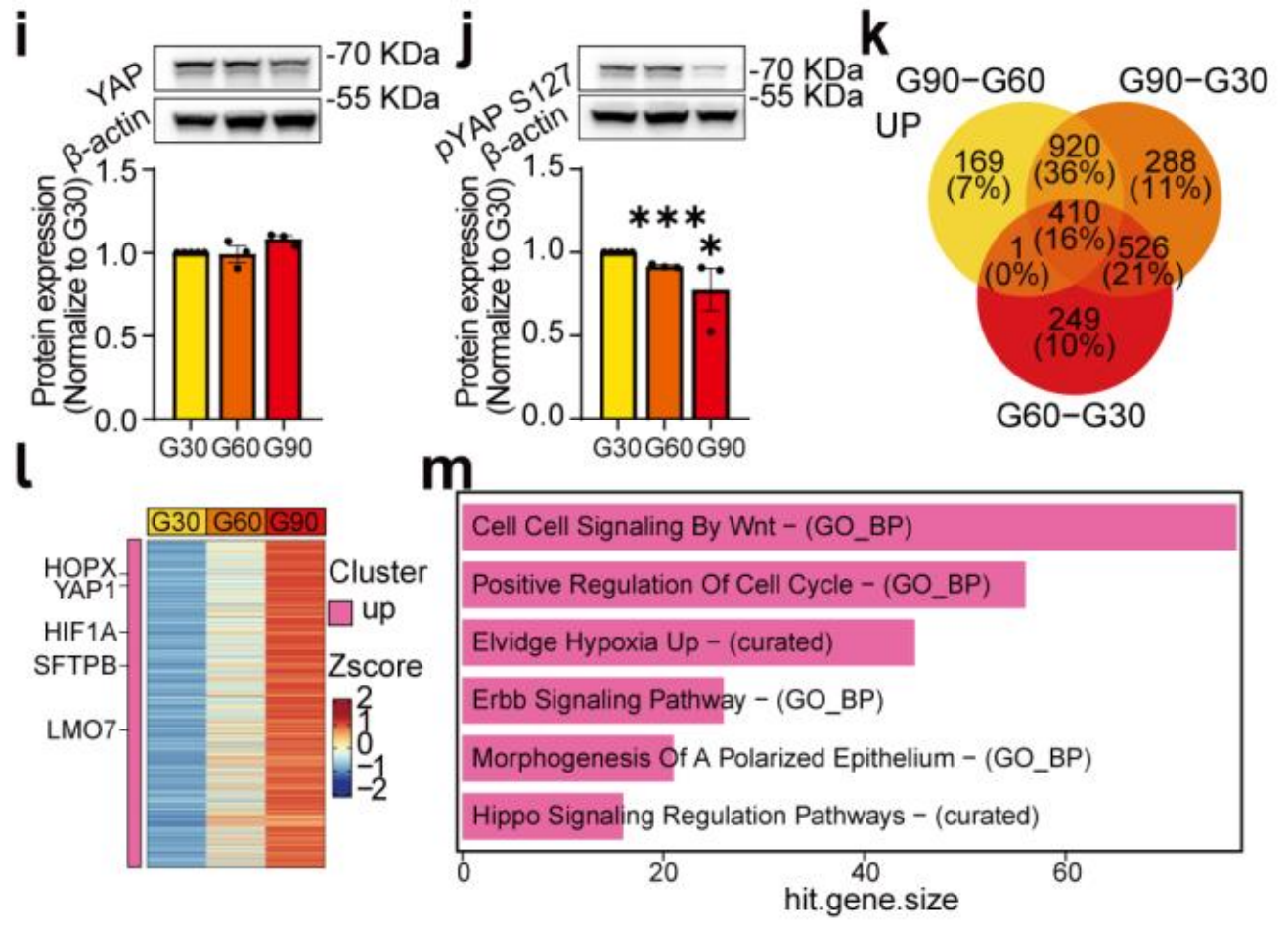

Fig. 7k-m

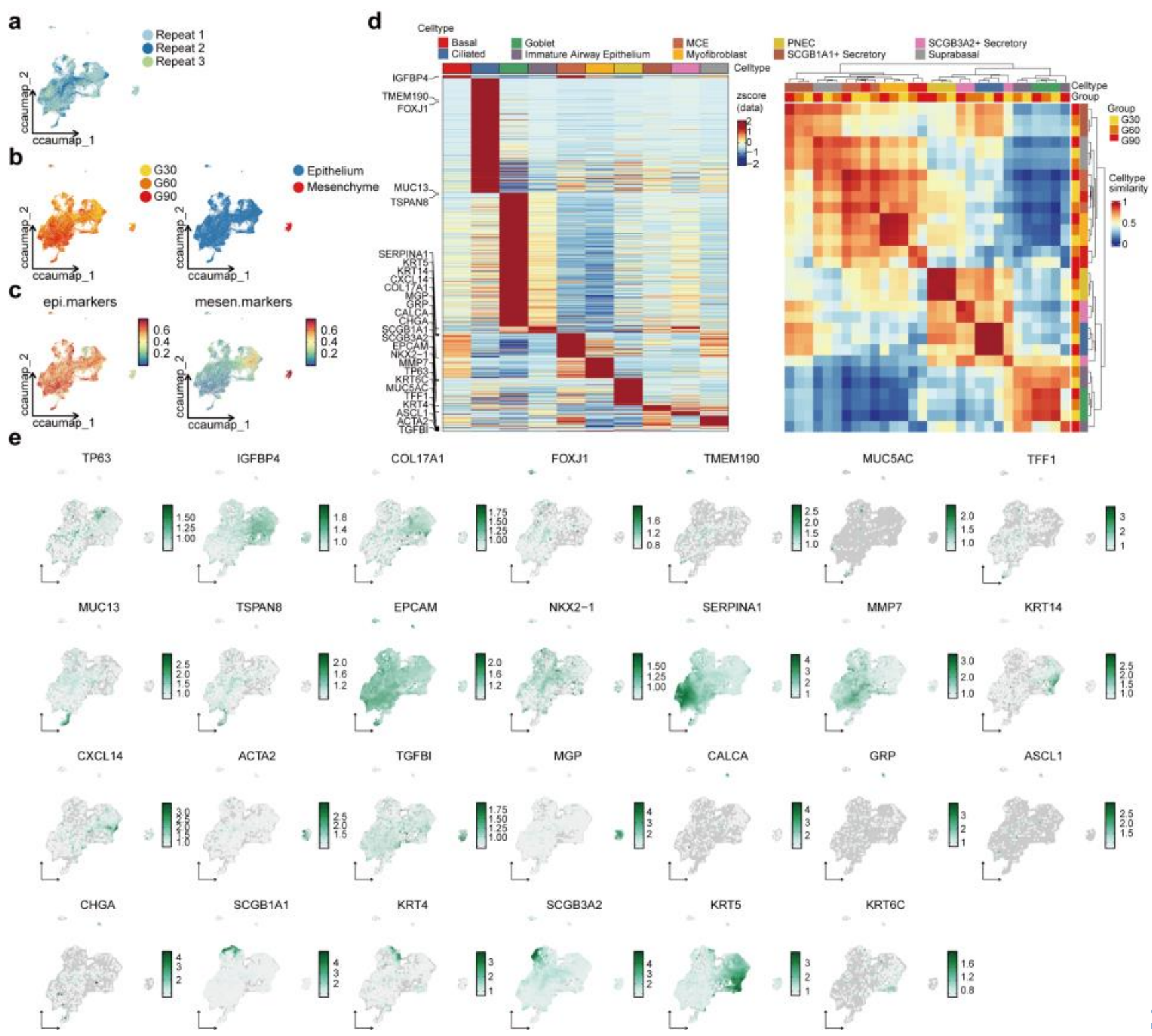

Supplementary Fig. 6

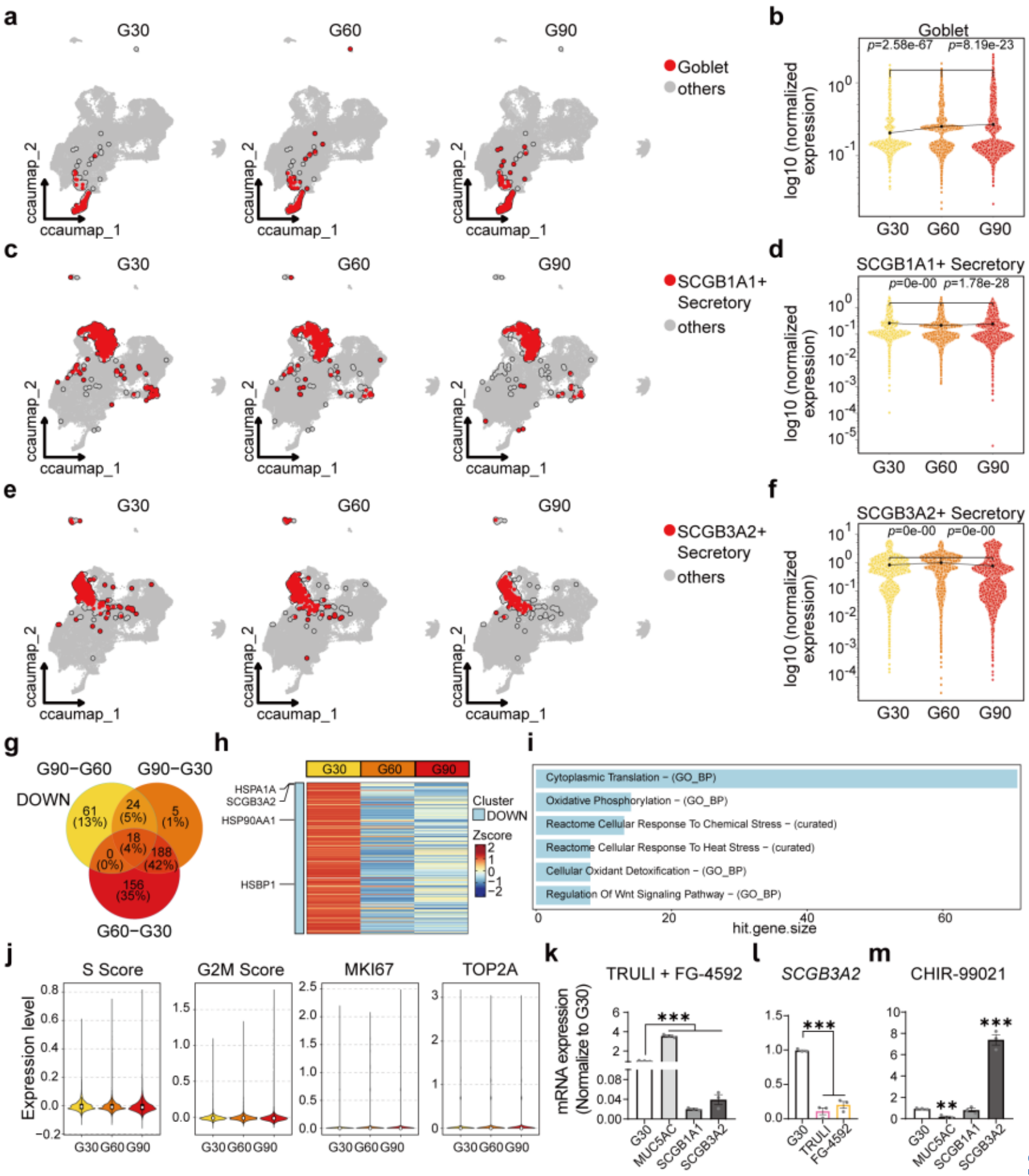

Supplementary Fig. 7a-j

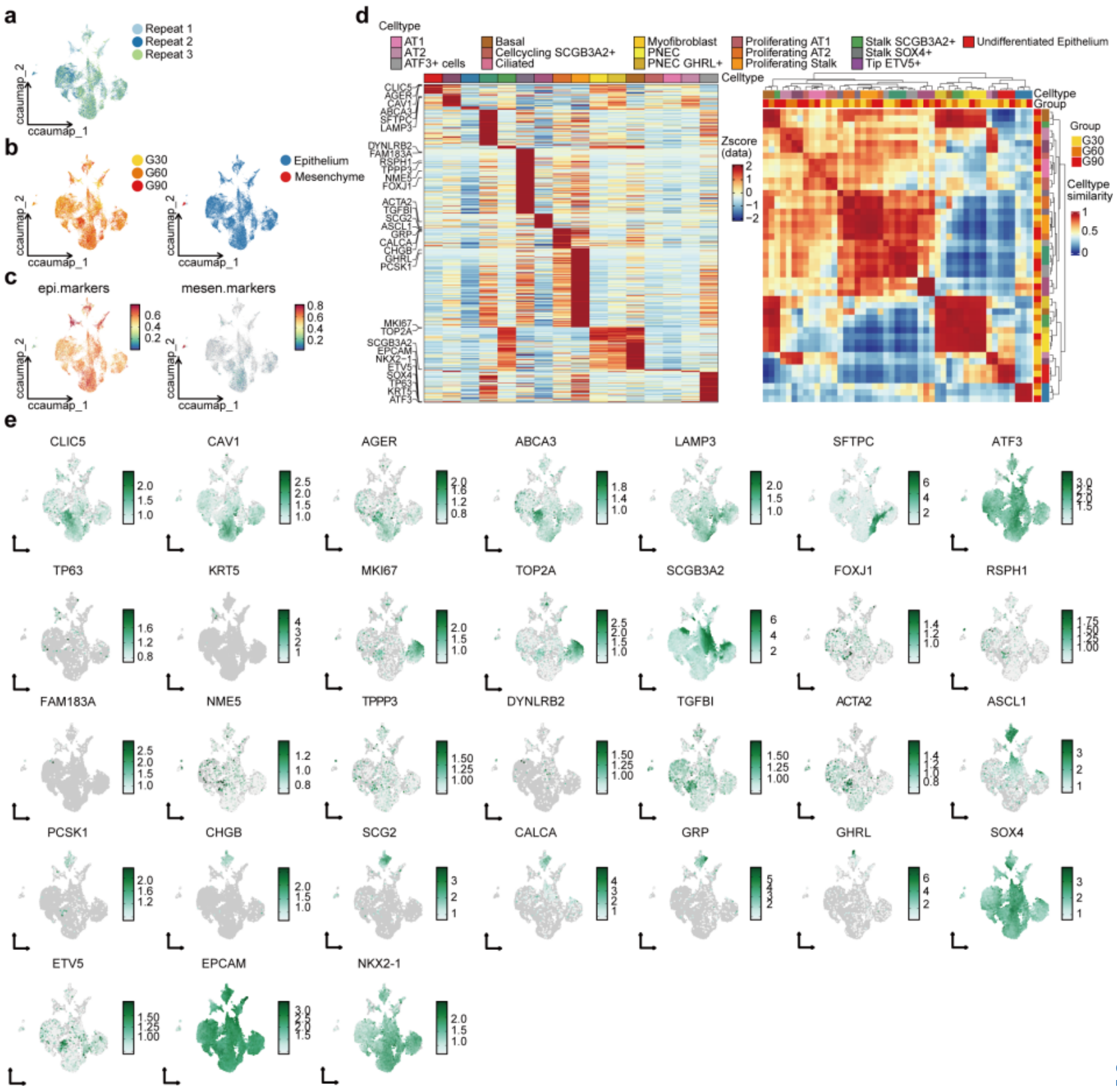

Supplementary Fig.11

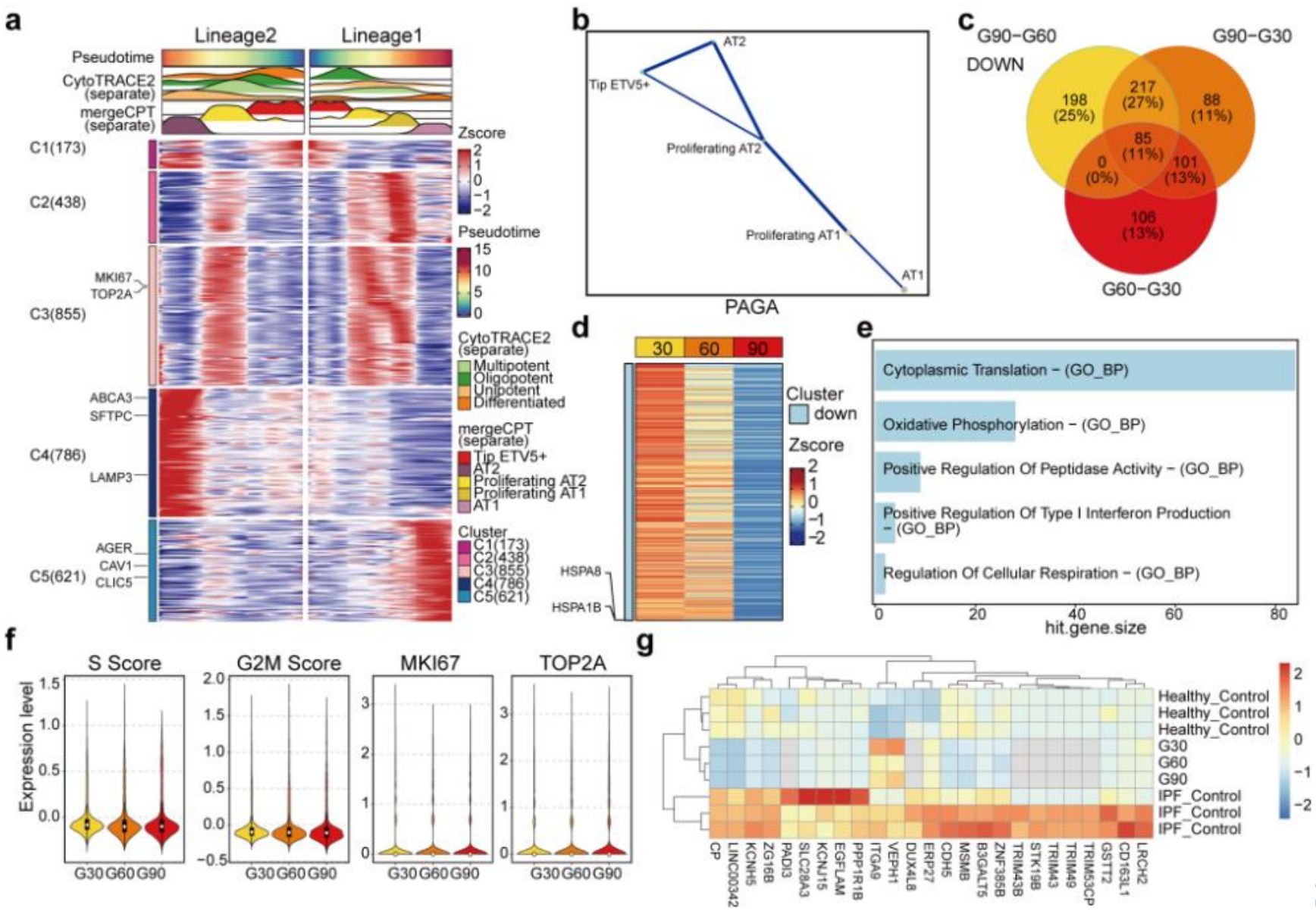

Supplementary fig.12

Meanwhile, we have updated the “Methods” section to clearly describe we included three biological replications (page 23) as following:

“For scRNA-seq, **three** biological replicates were included for each stiffness conditions (G30, G60, G90)......The raw sequencing reads for hAWOs (**3 biological replicates** for each stiffness).....The raw sequencing reads for hALOs (**3 biological replicates** for each stiffness) ”

Correspondingly, we have added more details in the “Supplementary Methods” of the revised Supplementary Information (page 30) that:

“Reads from three biological replicates of hAWOs and the three biological replicates of hALOs were each mapped to the GRCh38 genome.”

Minor comments

1. In the Discussion, the sentence regarding fetal and neonatal lungs (“…alveoli continue to mature postnatally under respiratory forces…”) should be removed, as the study focuses on region-specific effects and this statement is not directly relevant.

Thank you for pointing out this issue. We removed this sentence as Reviewer suggested.

2. Please remove or revise the sentence in the discussion:
“However, the development and maturation of the alveolar epithelium (air exchange under the respiratory system) are not simply better, and the external force seems to play an essential role in signaling pathway that determine epithelial fates.” As currently phrased, it is unclear and insufficiently relevant to the model used.

We appreciate the reviewer’s valuable suggestion. We have rephrased this sentence as follows (Page 18):

"However, the development and maturation of the alveolar epithelium require external mechanical force, which seem to play an essential role in promoting alvolar epithelial development."

Please also refer to our response to Question 7 of the major comments for more details.

Reviewer #3 (Remarks to the Author):

I co-reviewed this manuscript with one of the reviewers who provided the listed reports. This is part of the Nature Communications initiative to facilitate training in peer review and to provide appropriate recognition for Early Career Researchers who co-review manuscripts.

We thank Reviewer #3 for the time and effort devoted to the evaluation of our manuscript.

Reviewer #4 (Remarks to the Author):

The authors have adequately addressed my questions and revised the manuscript accordingly.

We sincerely thank the reviewer for the positive evaluation and are pleased that the revised manuscript has adequately addressed the concerns raised.

References

1. Li X, *et al.* 3D Culture of Chondrocytes in Gelatin Hydrogels with Different Stiffness. *Polymers* **8**, 269 (2016).
2. Miller AJ, *et al.* In Vitro and In Vivo Development of the Human Airway at Single-Cell Resolution. *Dev Cell* **53**, 117-128 e116 (2020).
3. Shiraishi K, *et al.* Biophysical forces mediated by respiration maintain lung alveolar epithelial cell fate. *Cell* **186**, 1478-1492 e1415 (2023).
4. Burgess CL, *et al.* Generation of human alveolar epithelial type I cells from pluripotent stem cells. *Cell Stem Cell* **31**, 657-675.e658 (2024).
5. Kindberg A, Hu JK, Bush JO. Forced to communicate: Integration of mechanical and biochemical signaling in morphogenesis. *Curr Opin Cell Biol* **66**, 59-68 (2020).
6. Cadena MA, *et al.* A 3D Bioprinted Cortical Organoid Platform for Modeling Human Brain Development. *Adv Healthc Mater* **13**, e2401603 (2024).
7. Ramirez GO, Mariano CA, Carter D, Eskandari M. Visceral pleura mechanics: Characterization of human, pig, and rat lung material properties. *Acta Biomaterialia* **189**, 388-398 (2024).
8. Luque T, *et al.* Local micromechanical properties of decellularized lung scaffolds measured with atomic force microscopy. *Acta Biomaterialia* **9**, 6852-6859 (2013).

Reviewer #5 (Remarks to the Author):

In my opinion, the authors have very thoroughly and comprehensively addressed Reviewer 2's stated concerns, and should be commended for the diligence and care in conducting new experiments and revising the wording of the manuscript to support the claims made.

We sincerely thank Reviewer 5 for the positive comments and recognition of our efforts. We are also grateful to Reviewer 2 for the constructive comments, which have helped us substantially improve the manuscript.
