# Supplementary material for: Extracellular matrix stiffness directs region-specific lung epithelial differentiation revealed by hPSC-derived lung organoids
Source: Nat Commun. 2026 Jul 17;17:8757. doi: 10.1038/s41467-026-75663-9 (PMC13493954; doi:10.1038/s41467-026-75663-9)
Supplement: Supplementary file 3 [file 41467_2026_75663_MOESM1_ESM.pdf]

Supplementary Information

Extracellular matrix stiffness directs region-specific lung epithelial differentiation revealed by hPSC-derived lung organoids

Zhiying Liao [#], Hao Meng [#*], Junjie Lv [#], Dong Wang, Hengrui Zhang, Runxi Jiang, Ruihao Lan, Yu Chen, Jiaqi Sun, Zonghong Li, Xiangping Yang, Xuepeng Chen [*], Jincun Zhao [*], Tao Xu [*], Huisheng Liu [*]

This file includes:

Supplementary Figures 1 to 14

Supplementary Tables 1 to 3

Supplementary Methods

Supplementary References

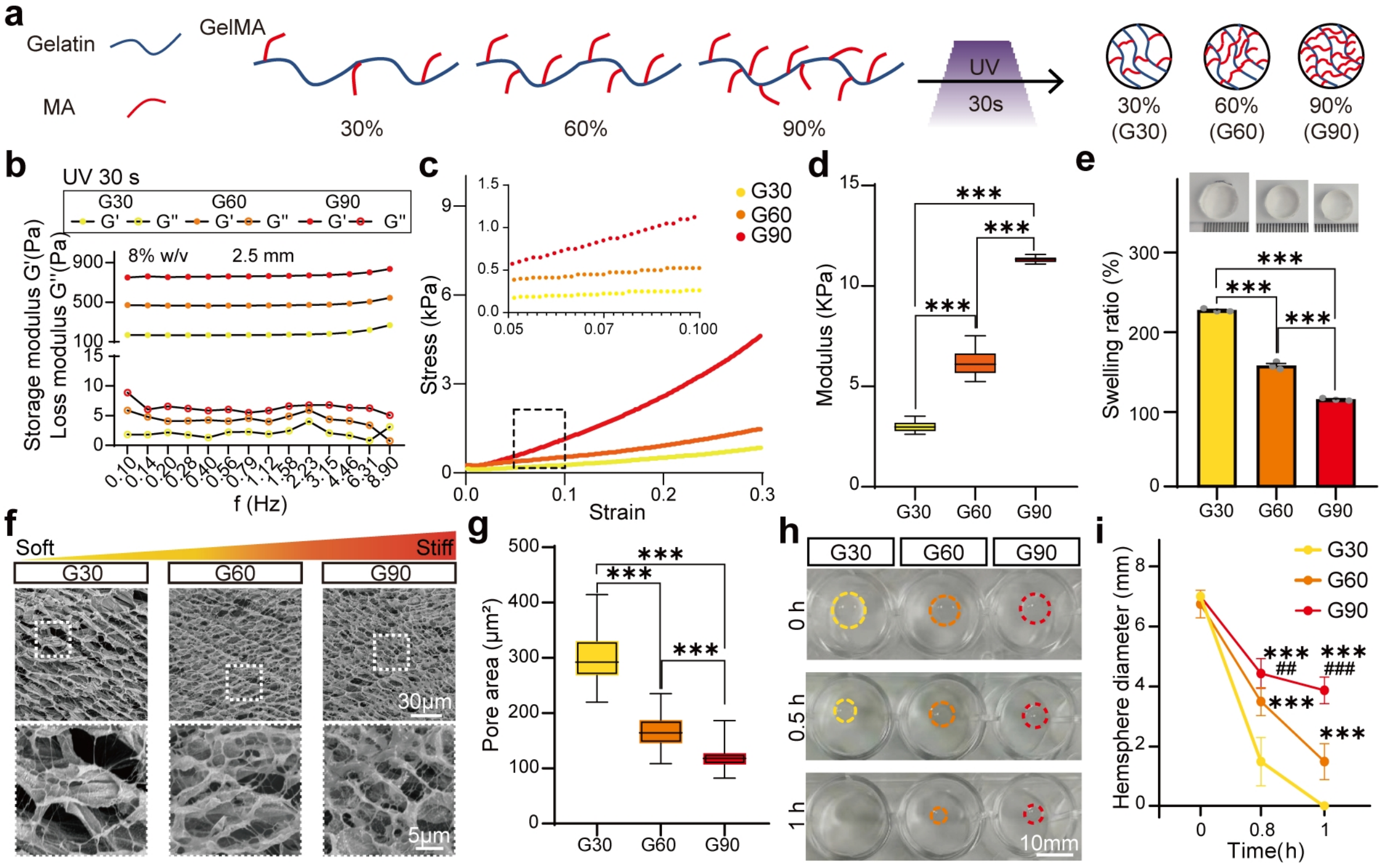

Supplementary Fig. 1. Preparation and characterization of stiffness-tunable GelMA hydrogels.

a Schematic showing GelMA hydrogel synthesis with varying degrees of methacrylation (MA) (30%, 60%, 90%) to produce G30, G60 and G90 hydrogels by UV crosslinking. Long blue curve is Gelatin; short red curve represents MA. **b** Frequency-dependent dynamic rheology measurements of storage modulus (G') and loss modulus (G'') for G30, G60 and G90 hydrogels. **c** Representative stress-strain curves of GelMA hydrogels. The inset magnifies the strain range of 0.050–0.100, highlighting the initial part where the stress and strain exhibit a linear increase. **d** Elastic modulus of G30, G60 and G90 hydrogels. Higher MA yields significantly stiffer hydrogels. $P < 0.001$, < 0.001, < 0.001. **e** Swelling ratios of G30, G60 and G90 hydrogels. Insets show representative images of hydrogels post-swelling. $P < 0.001$, < 0.001, < 0.001. **f** Representative scanning electron microscopy (SEM) images of hydrogels cross-section pore structures under varying stiffness ($n = 3$ independent biological replicates). **g** Quantification of pore area for G30, G60 and G90 hydrogels, showing a significant reduction in pore size with increasing stiffness. $P < 0.001$, < 0.001, < 0.001. **h** Degradation testing of hydrogels immersed in pH 7.4 PBS with 20 µg/mL collagenase type II. The hydrogel droplets were taken photos over time, showing size reduction due to degradation. Dashed circles indicate hydrogel boundaries. **i** Quantification of hydrogel hemisphere diameters during degradation. Higher stiffness (G90) leads to slower degradation compared to softer hydrogels (G30). $p = 0.0042$, < 0.001, < 0.001 (0.8 h); $P < 0.001$, < 0.001, < 0.001 (1 h). Yellow, orange, and red denote G30, G60, and G90 stiffness groups, respectively.

Statistical significance is indicated by * indicating comparison to G30, and # denoting comparisons to G60. Data are presented as mean ± SEM, with individual dots representing values from at least 3 replicates in 3 independent biological experiments. *P* values were calculated using one-way ANOVA (**d**, **e**, **g**) and two-way ANOVA (**i**) with Tukey's multiple comparison test. ** *and* ## $P < 0.01$, *** and ### $P < 0.001$.

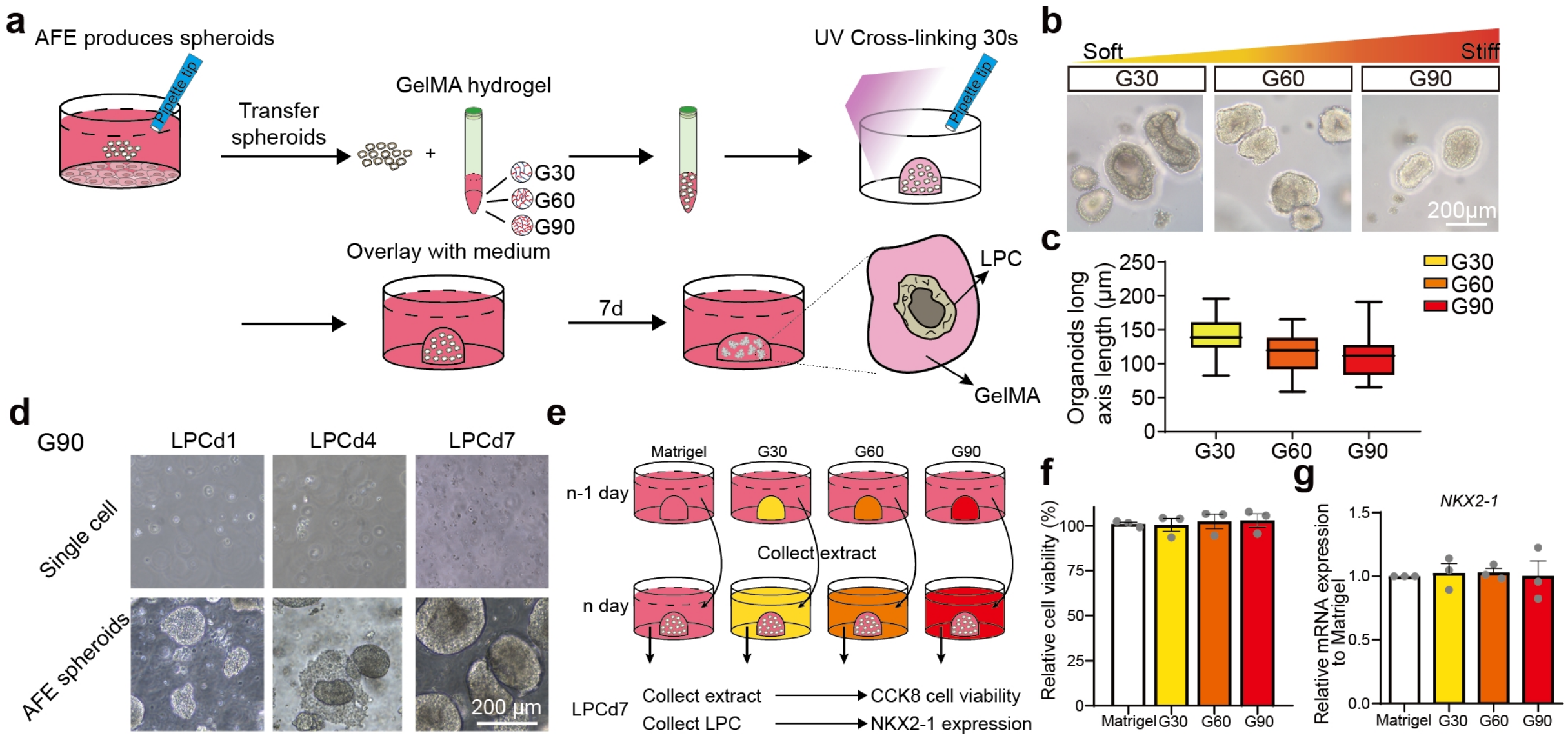

Supplementary Fig. 2. Hydrogel stiffness does not affect lumenized LPCs formation from AFE spheroids.

a Schematic illustrating the embedding AFE spheroids in GelMA hydrogels of different stiffness (G30, G60, G90), followed by UV crosslinking to solidify the droplets and culture in LPC medium for 7 days. **b** Representative bright-field images showing LPC differentiate in varying stiffness hydrogels appear epithelium structure with lumen inside. Scale bar, 200 μm (*n* = 3 independent biological replicates). **c** Quantification of LPC long axis length across G30, G60, and G90, showing no significant differences. **d** Under high stiffness (G90), AFE spheroids differentiate into LPCs over time (LPCd1, LPCd4, LPCd7), whilst single cells could not grow. Scale bar, 200 μm (*n* = 3 independent biological replicates). **e** Experimental design for hydrogel extract assay. Extract media from Matrigel, G30, G60 and G90 cultures were collected every other day and applied to LPC. in cell viability and differentiation. The extracts and LPCs are collected on LPC-d7 for CCK8 cell viability and *NKX2-1* gene expression assay. **f** Relative cell viability (CCK8) indicates extracts from G30, G60, and G90 yield cell viability comparable to the control. **g** qPCR of *NKX2-1* gene expression shows hydrogel extracts have no effect on LPC differentiation.

Data are presented as mean ± SEM, with individual dots representing values from at least 3 replicates in 3 independent biological experiments. *P* values were calculated using one-way ANOVA with Tukey's multiple comparison test (**c**, **f** , **g**).

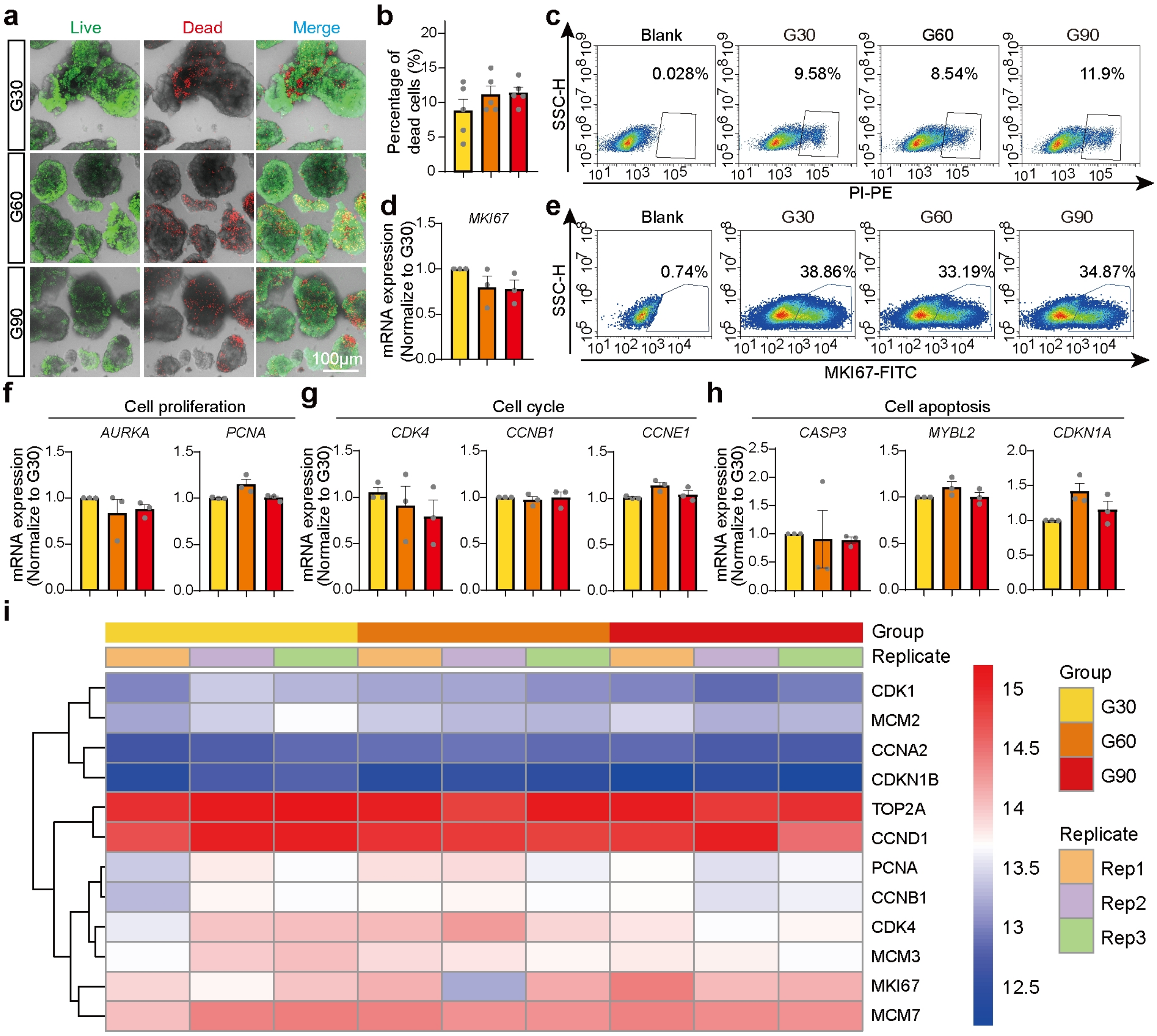

Supplementary Fig. 3. Hydrogel stiffness does not affect cell proliferation, cell cycle and apoptosis.

a Representative live (green) and dead (red) staining of LPCs in G30, G60, and G90 hydrogels (n = 3 independent biological replicates). **b** Quantification of the percentage of dead cells in different stiffness conditions, showing no significant variation. **c** Representative flow cytometry plots for propidium iodide (PI) staining, assessing cell viability in LPCs cultured under varying stiffness hydrogels. **d** qPCR analysis of *MKI67* mRNA expression, indicating no significant differences in cell proliferation among three groups. **e** Flow cytometry analysis of MKI67-positive LPCs showing similar proliferation rates across G30, G60, and G90 hydrogels. **f** qPCR analysis of proliferation-related genes (*AURKA* and *PCNA*) showing no stiffness-dependent changes. **g** qPCR analysis of cell cycle-related genes (*CDK4*, *CCNB1*, and *CCNE1*) showing comparable expression levels across stiffness groups. **h** qPCR analysis of apoptosis-related genes (*CASP3*, *MYBL2*, and *CDKN1A*) demonstrating no significant induction of apoptosis under varying stiffness. **i** Bulk RNA-seq heatmap of cell-cycle and proliferation genes shows no differences among hydrogel stiffness groups (G30, G60 and G90).

Data are presented as mean ± SEM, with distinct dots representing individual values from 3 independent biological experiments. Statistical comparisons were performed using one-way ANOVA with Tukey's multiple comparison test (**b**, **d**, **f**–**h**).

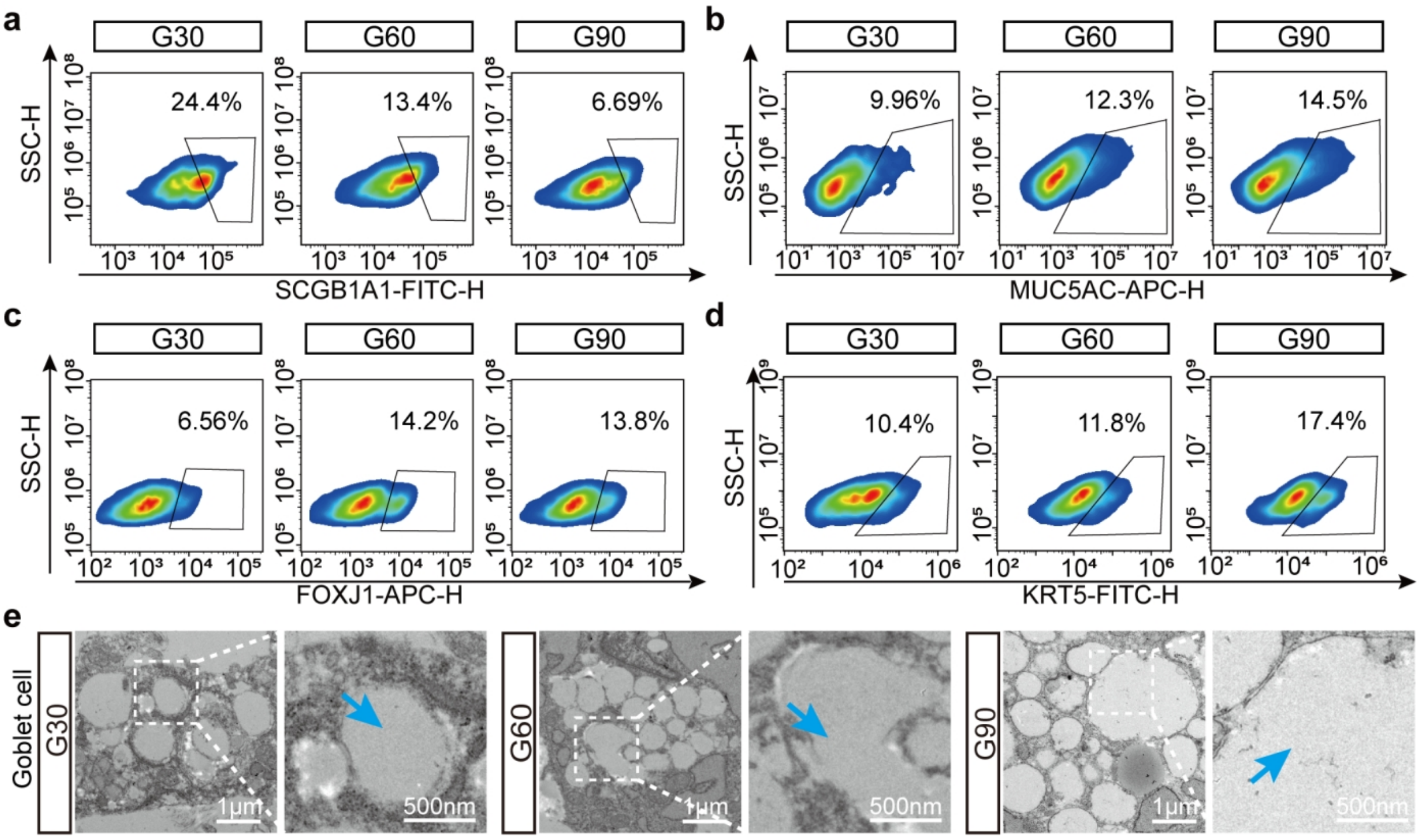

Supplementary Fig. 4. Flow cytometry analysis and TEM of hAWOs under varying stiffness.

a SCGB1A1 (secretory cells) shows reduced expression with increasing stiffness. **b** MUC5AC (goblet cells) exhibits a stiffness-dependent increase, particularly in G90. **c** FOXJ1 (ciliated cells) shows modest upregulation under G60 and G90. **d** KRT5 (basal cells) is significantly upregulated in G90 hydrogel. **e** TEM images of goblet cells showing secretory vesicles (blue arrows) in organoids.

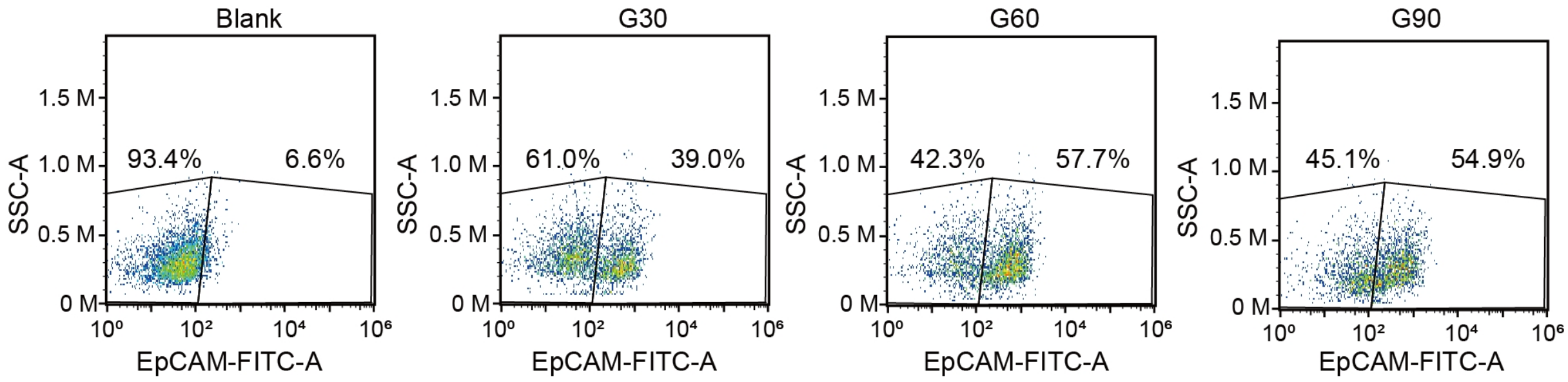

Supplementary Fig. 5. Flow cytometric isolation of EpCAM$^+$ epithelial cells from hAWOs prior to scRNA-seq.

Representative SSC-A vs. FITC-A_EpCAM plots for the unstained control (Blank) and for organoids cultured in GelMA hydrogels of increasing stiffness (G30, G60, G90). Gating thresholds were set using the Blank control. Numbers indicate the proportion (%) of EpCAM$^-$ (left gate) and EpCAM$^+$ (right gate) cells among total cells. The EpCAM$^+$ cells were collected for scRNA-seq.

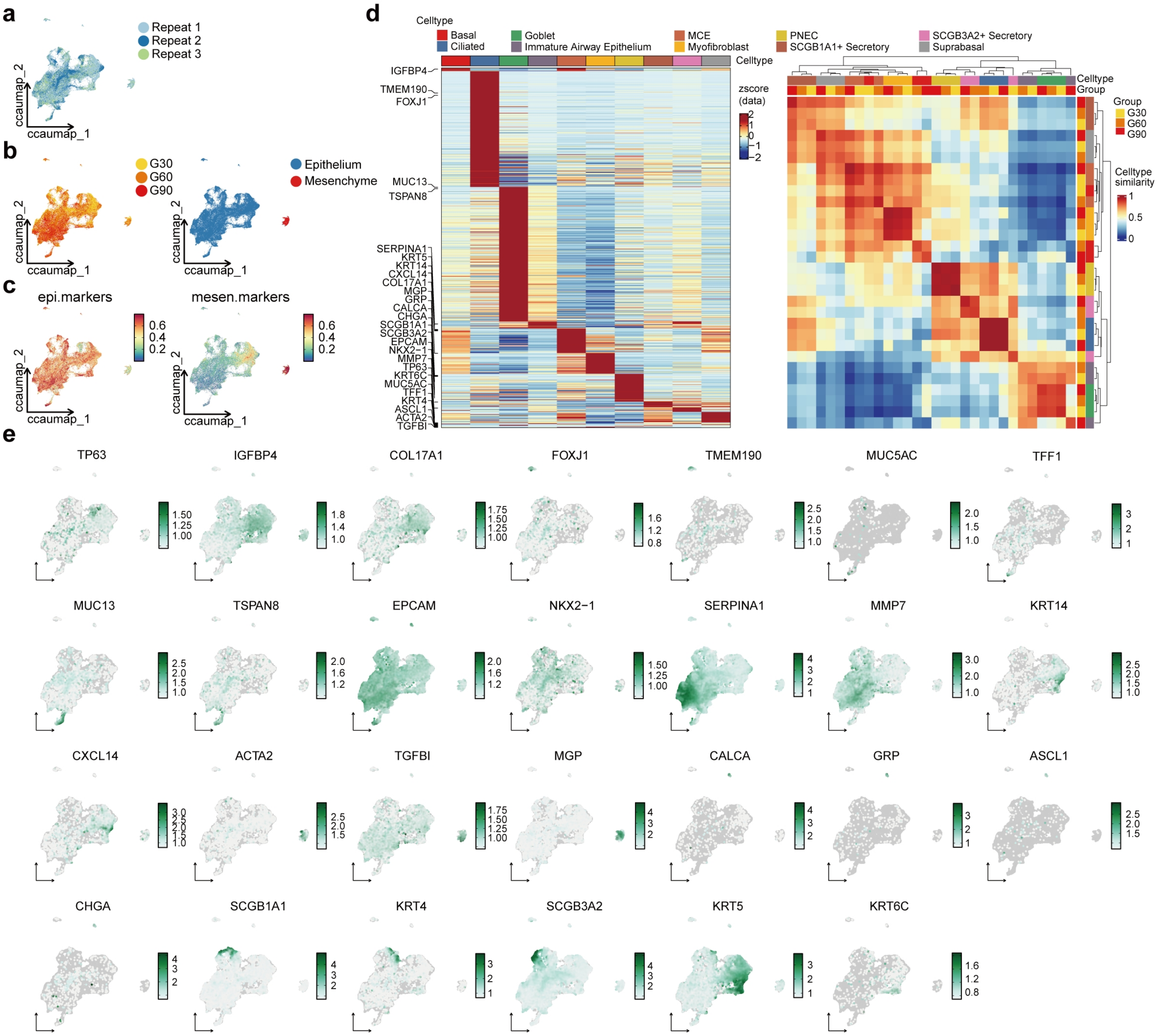

Supplementary Fig. 6. scRNA-seq of EpCAM$^+$ hAWOs defines airway lineages and cluster identities across stiffness conditions.

a UMAP of integrated scRNA-seq data from three independent biological replicates for each stiffness group, colored by replicate identity, showing consistent integration across replicates. **b** UMAP of integrated scRNA-seq data, showing cell clustering by stiffness (G30, G60, G90) and annotated major cell types (mesenchyme and epithelium). **c** Feature plots highlighting mesenchymal and epithelial cell marker expression across UMAP clusters. **d** Heatmap of canonical markers genes expression across cell types demonstrating the specificity (left) and consistency (right) of genes expression profiles across stiffness. **e** Feature plots showing normalized expression of representative markers for each airway epithelial cell population across the UMAP. A complete list of marker genes used for annotation is provided in Supplementary Table 2.

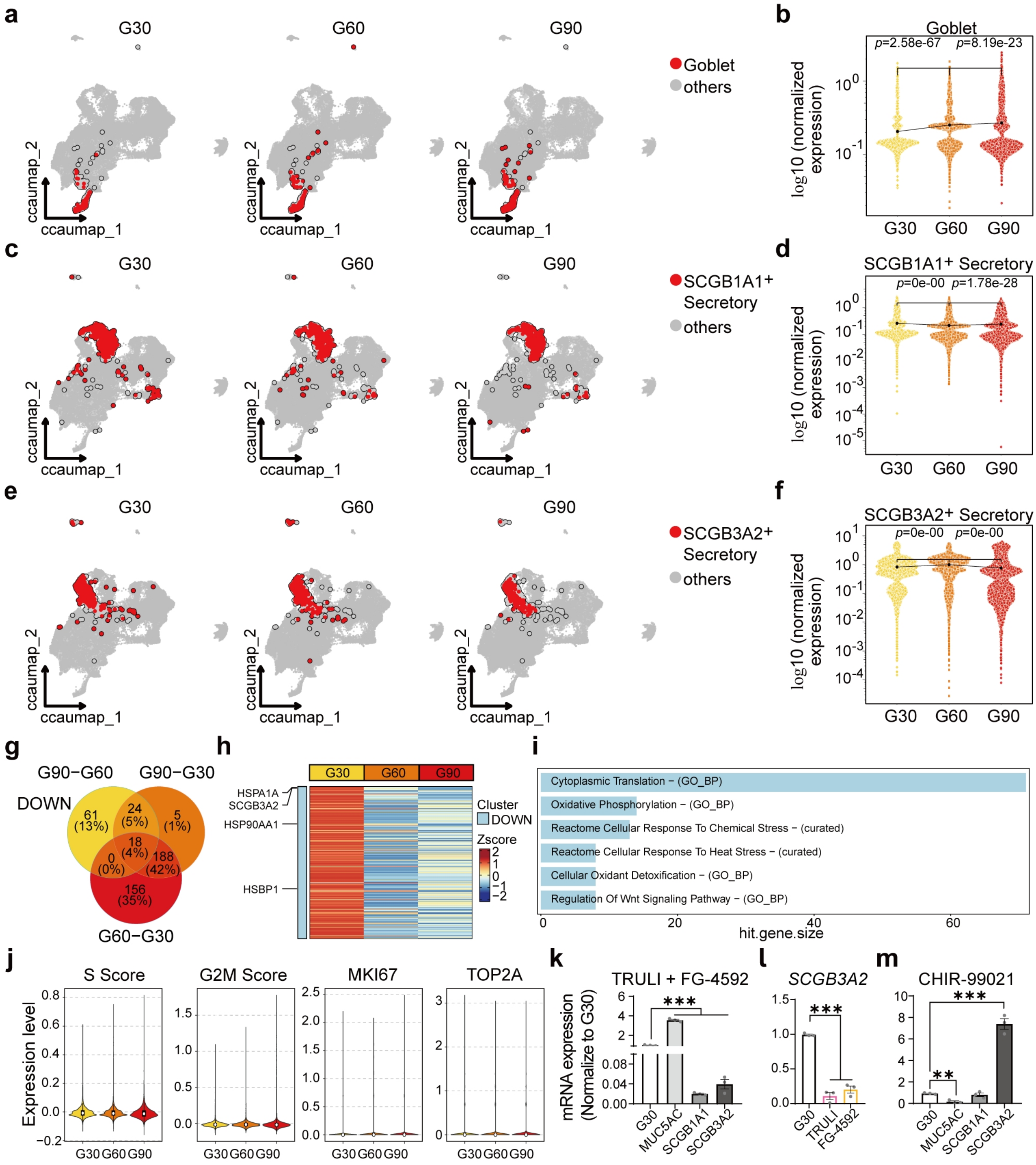

Supplementary Fig. 7. Secretory lineage distribution and stiffness responsive pathways in EpCAM⁺ hAWOs revealed by scRNA-seq and small molecules validation.

a–f UMAPs and corresponding violin plots for goblet cells **(a, b)**, SCGB1A1⁺ secretory cells **(c, d)** and SCGB3A2⁺ secretory cells **(e, f)** across G30, G60 and G90**. g** Venn diagram of downregulated genes from pairwise comparisons (G90-G60, G90-G30, G60-G30), highlighting shared stiffness-responsive genes. **h** Heatmap of 230 downregulated differentially expressed genes (DEGs). **i** Pathway enrichment of downregulated genes, including cytoplasmic translation, oxidative phosphorylation, Wnt signaling regulation, and cellular stress response. **j** Cell cycle scores (S Score, G2M Score) and proliferation markers (*MKI67*, *TOP2A*) are comparable across G30, G60 and G90. **k** qPCR in hAWOs treated with TRULI and FG-4592 shows increased *MUC5AC* and decreased expressions of *SCGB1A1* and *SCGB3A2*. $P < 0.001$, < 0.001, < 0.001. **l** In G30 organoids, TRULI and FG-4592 treatment significantly reduces

SCGB3A2 expression. $P < 0.001$, < 0.001. **m** CHIR-99021 treatment of G30 increases *SCGB3A2* expression but decreases *MUC5AC* expression. $p = 0.001$, $P < 0.001$.
Data are presented as mean ± SEM, $^{**}P < 0.01$, $^{***}P < 0.001$, analyzed by unpaired t test, one-way ANOVA with Dunnett's multiple comparison test (**k**–**m**) (*n*=3 independent biological repeats).

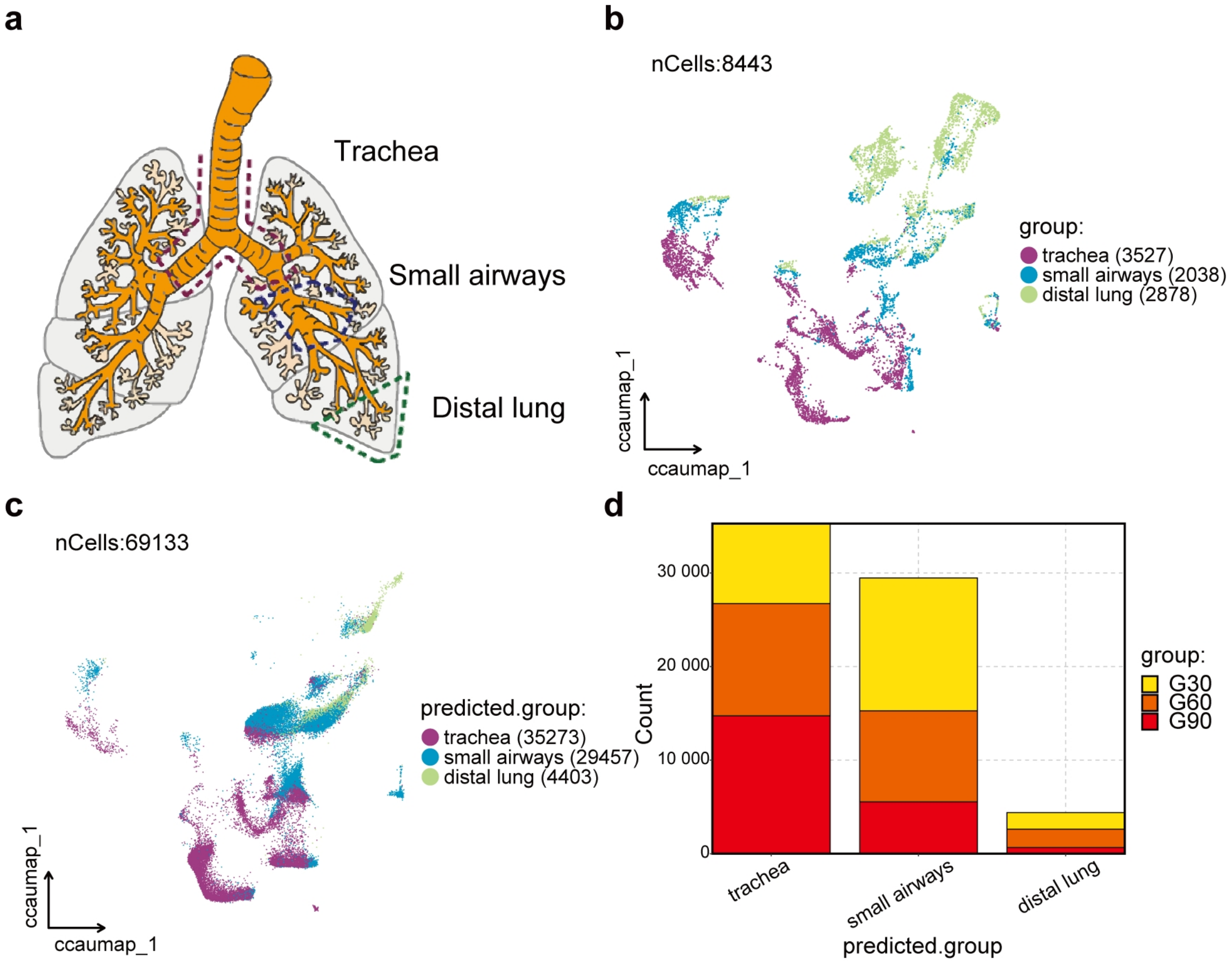

Supplementary Fig. 8. *In vitro* derived AWOs under stiff-to-soft hydrogels recapitulate molecular features of native fetal trachea and small airway cells.

a Anatomical diagram of the trachea, small airways and distal lung of the fetal regional airway epithelium. **b** UMAP of the re-processed data of E-MTAB-8221, labeled by original airway regions of trachea (purple), small airways (blue) and distal lung (green). **c** Integrated UMAP showing our stiffness-guided hAWOs projected on the predicted regions of the published fetal airway map. **d** Bar chart shows the numbers of epithelial cells from different stiffness-guided hAWOs across the predicted airway regions of trachea, small airways and distal lung.

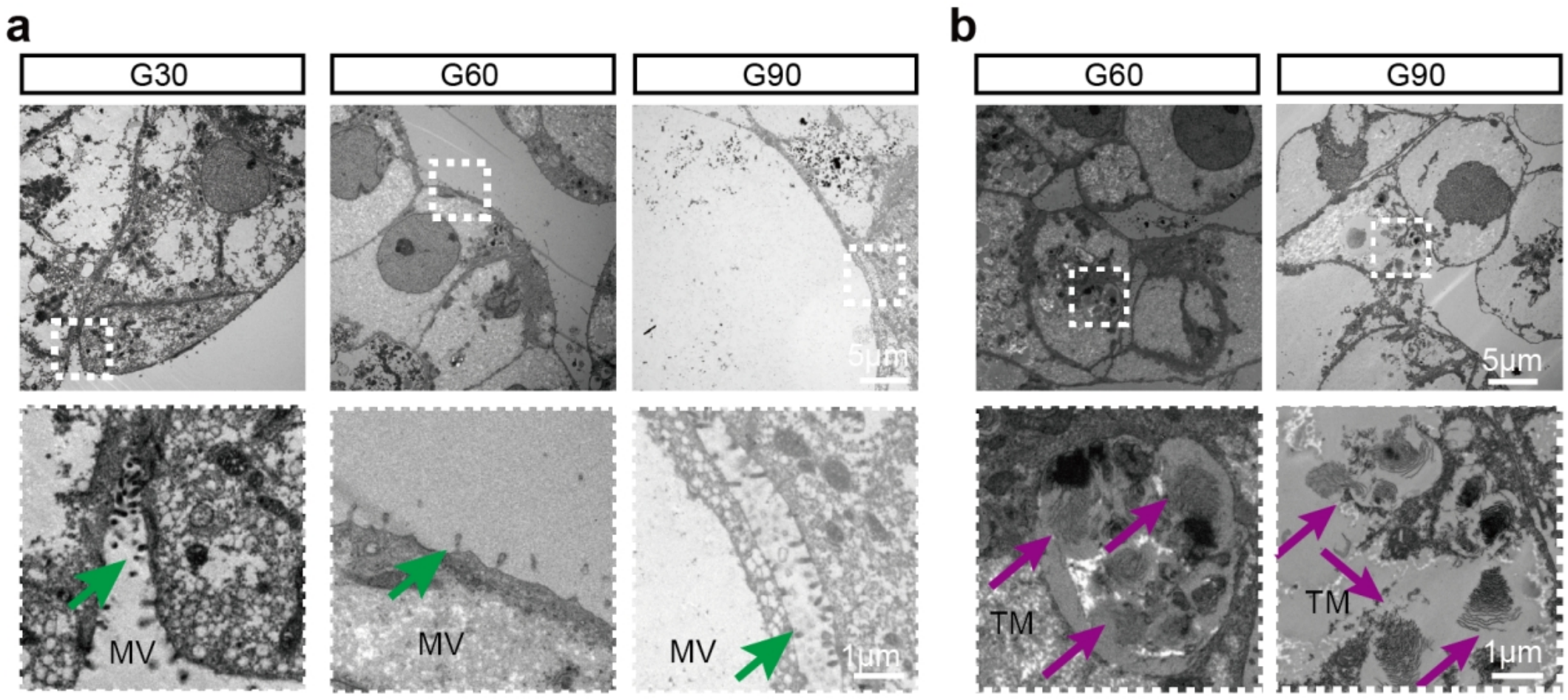

Supplementary Fig. 9. TEM images of microvilli and tubular myelin in mature AT2 cells.

a Representative TEM images showing microvilli (MV, green arrows) on the surface of AT2 cells in hALOs under G30, G60, and G90, showing no stiffness-dependent changes (n = 3 independent biological replicates). **b** TEM images displaying tubular myelin (TM, purple arrow), a key marker of AT2 surfactant secretion, was observed in G60 and G90 hALOs but not in G30.

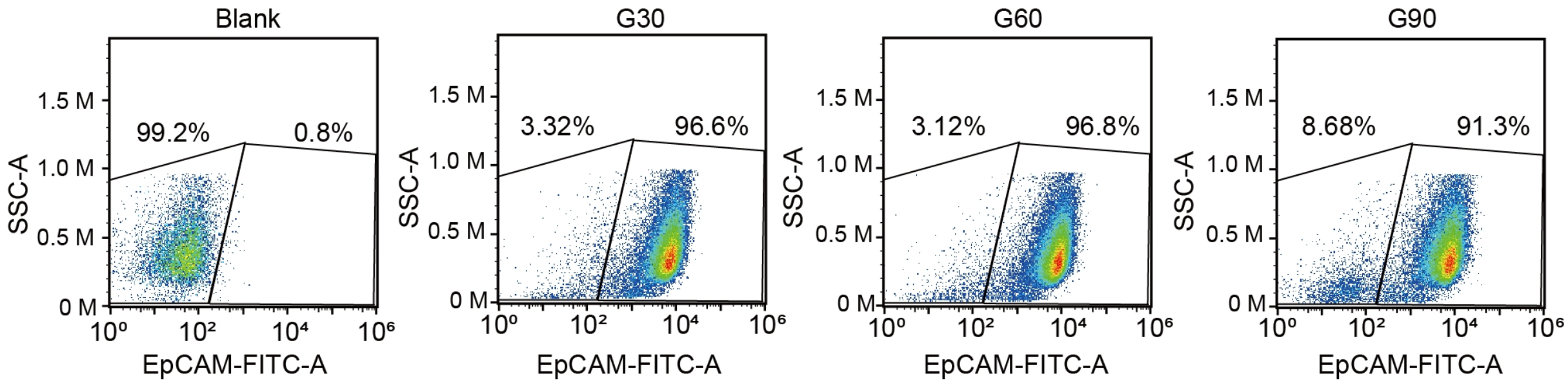

Supplementary Fig. 10. Flow cytometric isolation of EpCAM$^+$ epithelial cells from hALOs prior to scRNA-seq.

Representative SSC-A vs. FITC-A_EpCAM plots for the unstained control (Blank) and for hALOs cultured in GelMA hydrogels of increasing stiffness (G30, G60, G90). Gating thresholds were set using the Blank control. Numbers indicate the proportion (%) of EpCAM$^-$ (left gate) and EpCAM$^+$ (right gate) cells among total cells. The EpCAM$^+$ cells were collected for scRNA-seq.

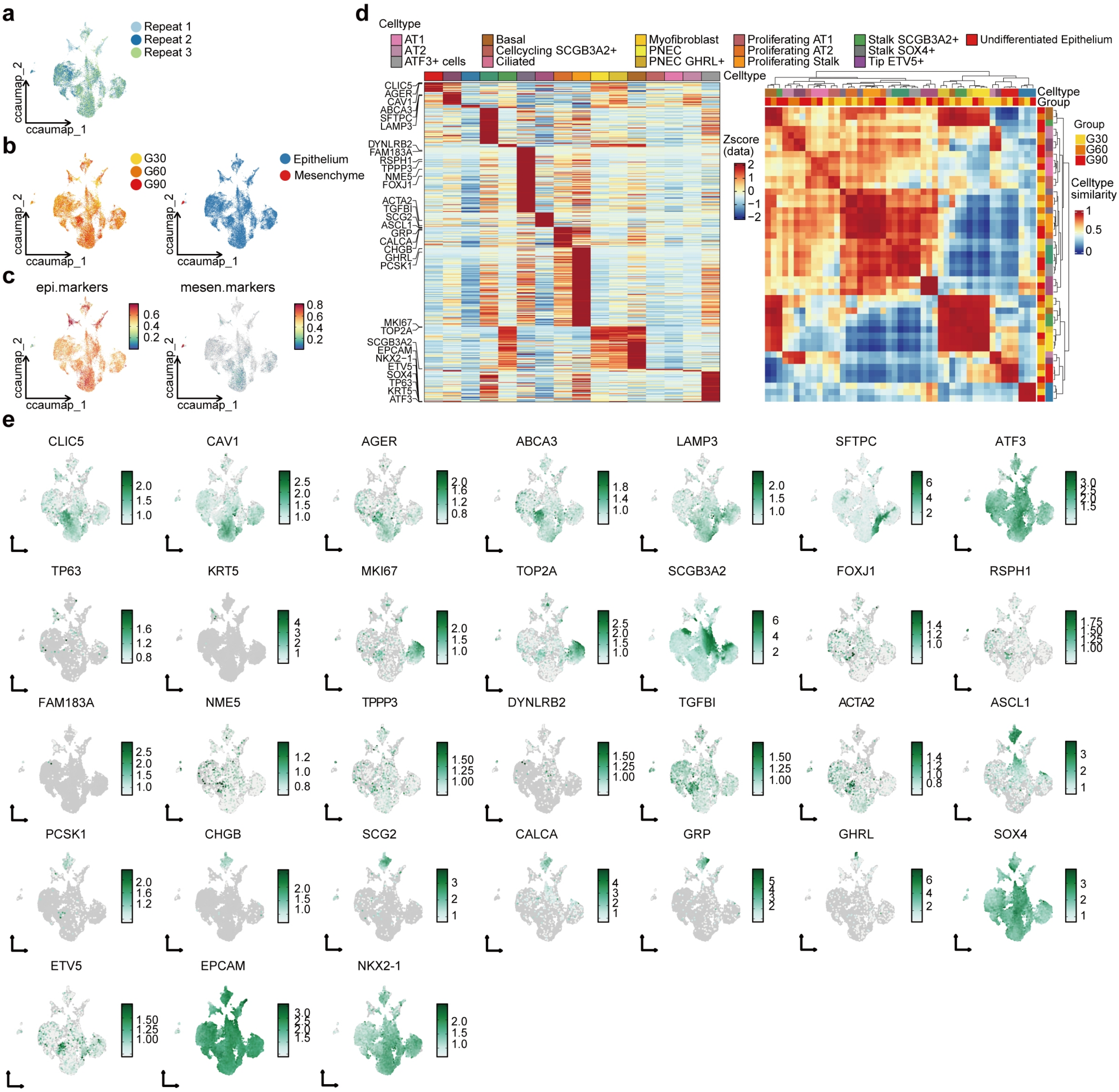

Supplementary Fig. 11. scRNA-seq of EpCAM$^+$ hALOs defines alveolar lineages and cluster identities across stiffness conditions.

a UMAP of integrated scRNA-seq data from three independent biological replicates or each stiffness group, colored by replicate identity, showing consistent integration across replicates. **b** UMAP of integrated scRNA-seq data, showing cell clustering by stiffness (G30, G60, G90) and annotated major cell types (mesenchyme and epithelium). **c** Feature plots highlighting mesenchymal and epithelial cell marker expression across UMAP clusters. **d** Heatmap of canonical markers genes' expression across cell types demonstrating the specificity (left) and consistency (right) of genes expression profiles across stiffness. **e** Feature plots showing normalized expression of representative markers for each alveolar epithelial cell population across the UMAP. A complete list of marker genes used for annotation is provided in Supplementary Table 3.

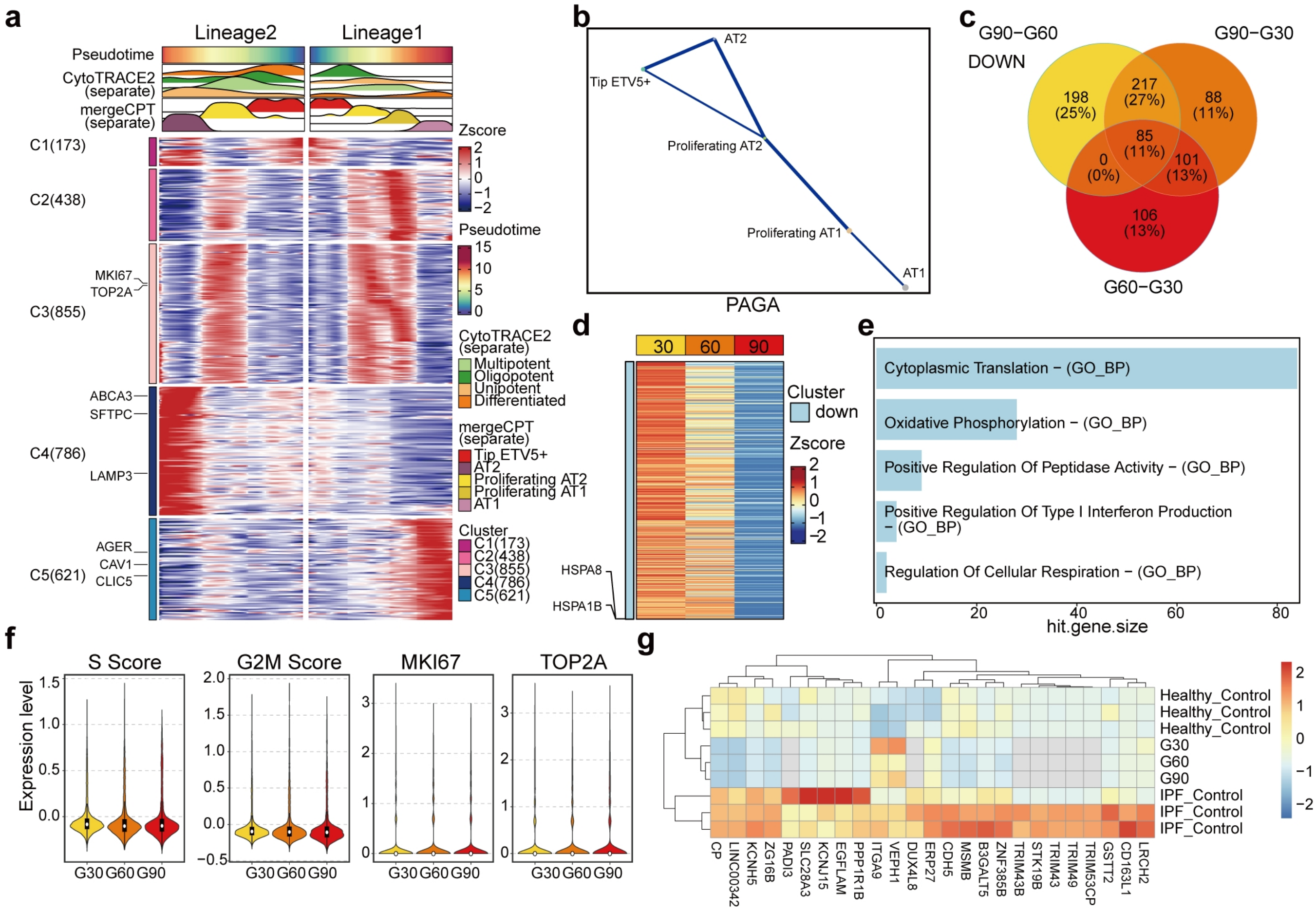

Supplementary Fig. 12. scRNA-seq elucidates ECM stiffness-dependent alveolar differentiation in hALOs.

a Pseudotime heatmap depicting gene expression changes along the bifurcation lineages (Lineage 1 and Lineage 2) of hALOs differentiation. The right panel represents AT1 lineage, including AT1-differentiation related genes such as *AGER*, *CAV1* and *CLIC5*, whilst the left panel represents AT2-lineage of genes such as *ABCA3*, *SFTPC* and *LAMP3*. **b** Partition-based graph abstraction (PAGA) trajectory analysis illustrating the differentiation relationships among AT1, proliferating AT1, proliferating AT2, AT2, and tip ETV5^{+} cells populations. The edge thickness denotes connectivity strength. **c** Venn diagram of downregulated DEGs (G90 vs. G60, G90 vs. G30, G60 vs. G90) across stiffness variations, identifying shared and stiffness-specific genes. The central overlapped region contains 85 genes consistently decreased with increased stiffness. **d** Heatmap of shared down-regulated gene sets across stiffness groups. **e** GO-BP enrichment for the downregulated pathways. **f** Violin plots of cell cycle scores (S Score, G2M Score) and proliferation markers (*MKI67*, *TOP2A*) showing comparable levels across G30, G60 and G90. **g** Heatmap of idiopathic pulmonary fibrosis (IPF)-associated genes comparing hALOs (G30, G60, G90) with healthy-control and IPF-control lung samples demonstrating gene-expression profiles that cluster more closely with healthy controls than IPF controls.

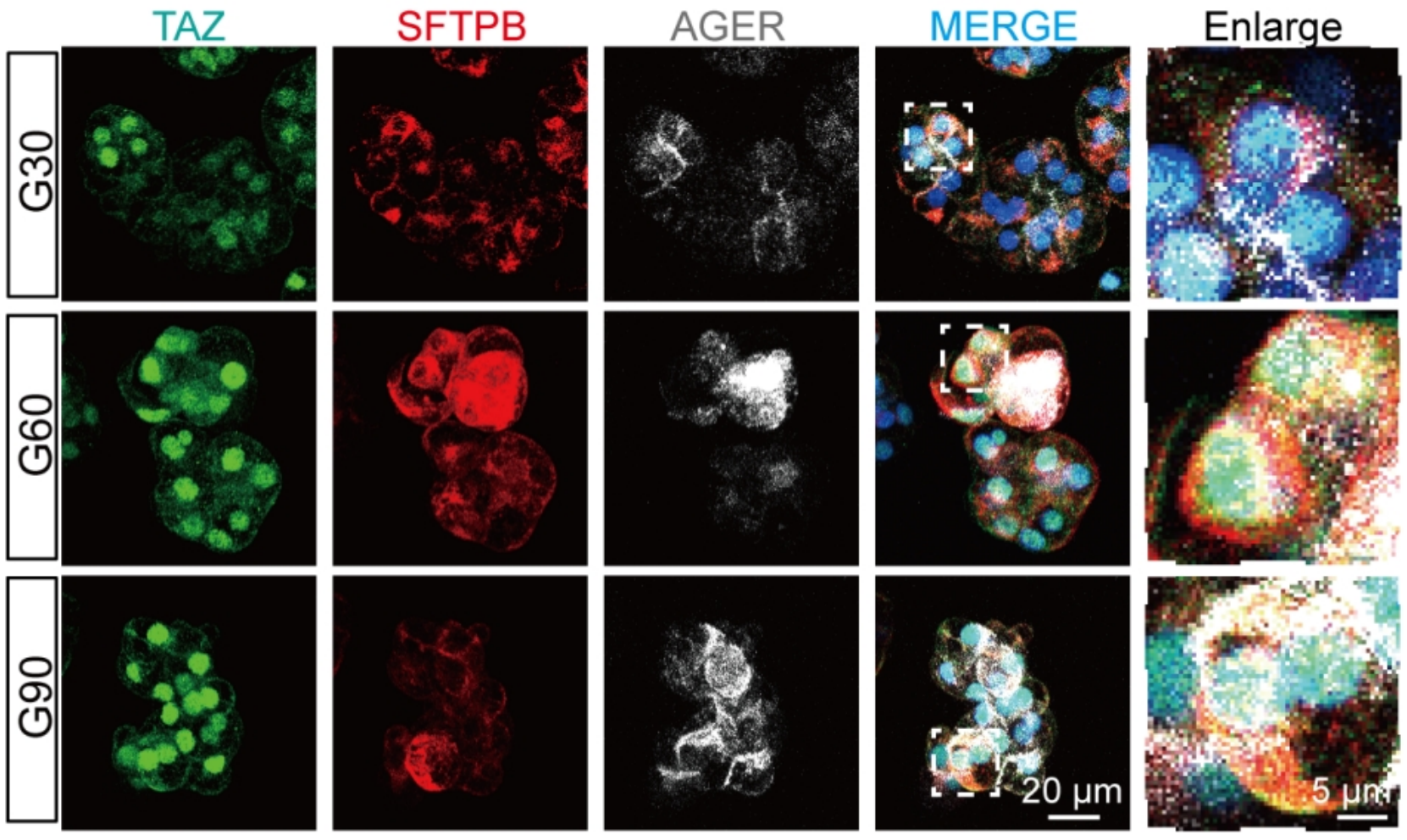

Supplementary Fig. 13. TAZ localization in hALOs across hydrogel stiffness.
Representative confocal images of hALOs cultured in GelMA hydrogels of increasing stiffness (G30, G60, G90) stained for TAZ (green), SFTPB (red; AT2 marker) and AGER (white/gray; AT1 marker). MERGE panels include DAPI (blue). Dashed boxes indicate regions shown at higher magnification at right. Scale bars: 20 µm (overview) and 5 µm (zoom). *n* = 3 independent biological replicates.

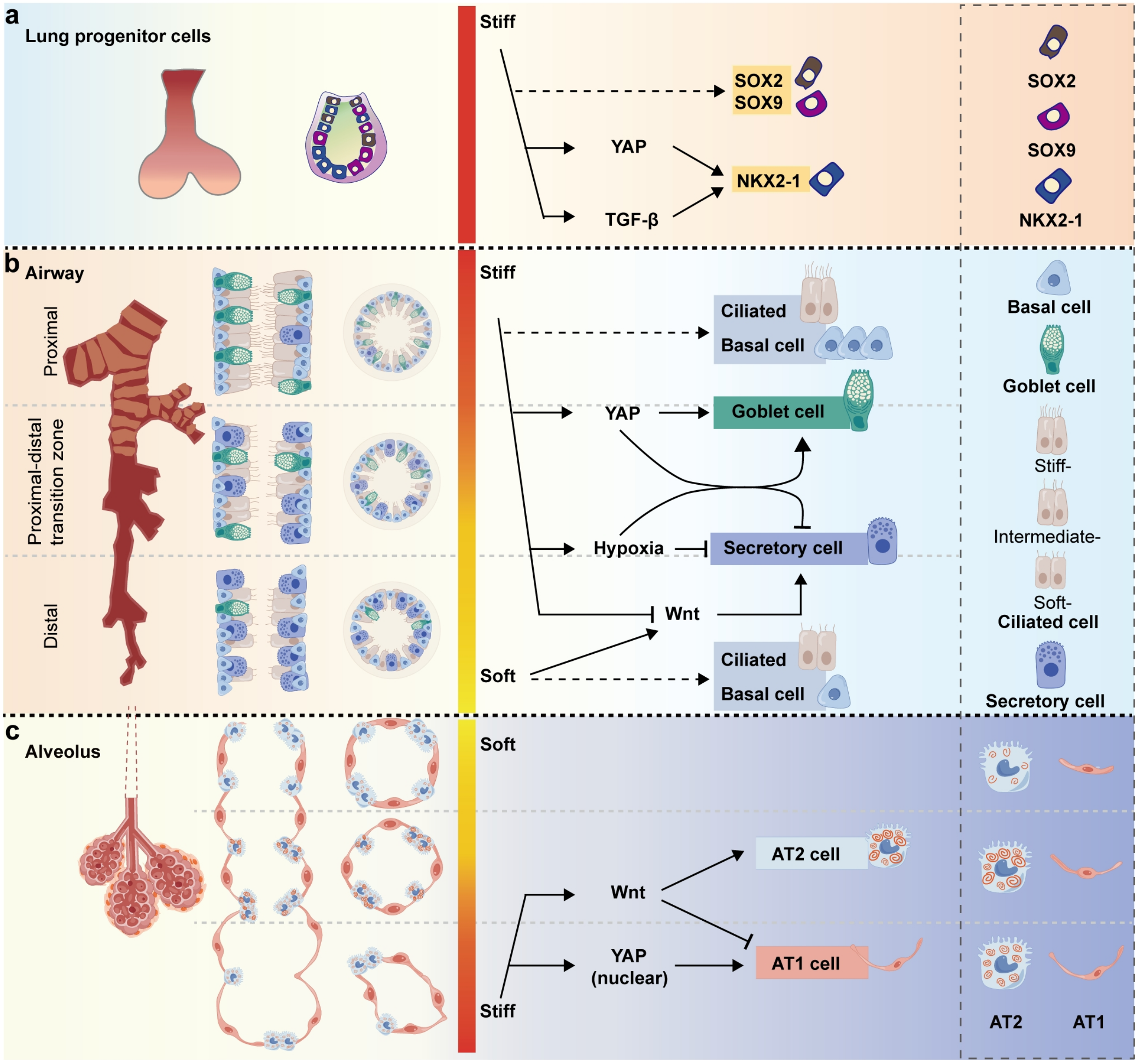

Supplementary Fig. 14. Schematic of ECM stiffness regulates lung epithelial cell fate during lung development. This schematic illustrates how ECM stiffness influences epithelial lung differentiation and cell fate across distinct lung regions. The diagram integrates regional lung morphology and cell composition (left), the stiffness-mediated regulatory mechanisms (middle), and cell type illustrations (right).

a Lung progenitor cells: High ECM stiffness enhances the expression of key LPC markers, including NKX2-1, SOX9, and SOX2. In particular, NKX2-1+ lung progenitors are upregulated via activation of the YAP and TGF-β signaling pathways. Enhancing NKX2-1 expression is critical for lung lineage specification. **b** Airway: Proximal airway cells, including goblet, ciliated, and basal cells, with ciliated cells exhibit longer and denser cilia. Proximal-to-distal transitional zone features more secretory cells, fewer goblet cells, and ciliated cells with shorter and less abundant cilia. Distal airway is predominantly composed of secretory cells, alongside ciliated cells with sparse cilia and seldom goblet cells. Stiffness-mediated regulation in stem cell-derived lung organoid models recapitulates these region-specific cell compositions observed *in vivo*. Mechanistically, high stiffness promotes the differentiation of goblet and secretory cells primarily through YAP (Hippo) and hypoxia signaling, while low stiffness supports the emergence of secretory cells and promotes secretory cells differentiation via activation of the Wnt signaling pathway. **c** Alveolus**:** Increased ECM stiffness drives AT2 cell maturation, characterized by mature lamellar bodies. Further stiffening induces the transition of AT2 to AT1 cells, resulting in AT1 cells adopting a thinner and more elongated morphology.

The Wnt signaling pathway promotes AT2 cell differentiation and maturation while inhibiting AT1 cell generation. In contrast, nuclear translocation of YAP and its upregulation in response to stiffness facilitate AT1 cell differentiation.

Supplementary Table 1. Primers used in the present study

Gene	Forward (5'-3')	Reverse (5'-3')
NKX2-1	CGGCATGAACATGAGCGGCAT	GCCGACAGGTACTTCTGTTGCTTG
SOX2	GCTTAGCCTCGTCGATGAAC	AACCCCAAGATGCACAACTC
SOX9	GACTACACCGACCACCAGAACTCC	CTGAGCTCGGCGTTGTG
PAX8	TGCCTCACAACTCCATCAGA	CAGGTCTACGATGCGCTG
CDX2	GGGCTCTCTGAGAGGCAGGT	GGTGACGGTGGGGTTTAGCA
GATA4	GCGGTGCTTCCAGCAACTCCA	GACATCGCACTGACTGAGAACG
MUC5AC	ACCAATGCTCTGTATCCTTCCC	GTTTGGGTGGAGTAAGCCACA
SCGB1A1	TTCAGCGTGTCATCGAAACCC	ACAGTGAGCTTTGGGCTATTTTT
SCGB3A2	GGCTAAGGAAGTGTGTAAATGAGC	CCATCCACCTCCGCTCTTTATC
ID2	GACAGCAAAGCACTGTGTGG	TCAGCACTTAAAAGATTCCGTG
ABCA3	CAGTGGACGGGCATACATCA	CCTTCAGCTGGGCGTAGAAA
SFTPB	TGCCTGGACCACCTCATCCTTG	GTCCTCACACTCTTGGCATAGG
SFTPC	AGCAAAGAGGTCCTGATGGA	CGATAAGAAGGCGTTTCAGG
PDPN	AGGAGAGCAACAACTCAACGGGAA	TTCTGCCAGGACCCAGAGC
HOPX	GCCTTTCCGAGGAGGAGAC	TCTGTGACGGATCTGCACTC
AGER	GCCACTGGTGCTGAAGTGTA	TGGTCTCCTTTCCATTCCTG
SARS-CoV-2 NP	CTGCAGATTTGGATGATTTCTCC	CCTTGTGTGGTCTGCATGAGTTTAG
MKI67	GAAAGAGTGGCAACCTGCCTTC	GCACCAAGTTTTACTACATCTGCC
AURKA	GCAACCAGTGTACCTCATCCTG	AAGTCTTCCAAAGCCCACTGCC
PCNA	ACACTAAGGGCCGAAGATAACG	ACAGCATCTCCAATATGGCTGA
CDK4	CTTTGGCAGCTGGTCACATGG	CTCAGATCAAGGGAGACCCTCAC
CCNB1	GACCTGTGTCAGGCTTTCTCTG	GGTATTTTGGTCTGACTGCTTGC
CCNE1	TGGATGTTGACTGCCTTGAA	TCCCCGTCTCCCTTATAACC
CASP3	GGCATGGAGAACACTGAAAAC	GCGAATCTGTTTCTTTGCATG
MYBL2	CACCAGAAACGAGCCTGCCTTA	CTCAGGTCACACCAAGCATCAG
CDKN1A	CGATGGAACTTCGACTTTGTCA	GCACAAGGGTACAAGACAGTG
GAPDH	TGCACCACCAACTGCTTAGC	GGCATGGACTGTGGTCATGAG

Supplementary Table 2. List of marker genes used for hAWOs cell type annotation

Cell type	Markers
Basal	EpCAM, TP63, IGFBP4, COL17A1
Ciliated	EpCAM, FOXJ1, TMEM190
Goblet	EpCAM, MUC5AC, TFF1, MUC13, TSPAN8
Immature Airway Epithelium	EpCAM, NKX2-1, SERPINA1, MMP7
MEC	EpCAM, KRT14, CXCL14
Myofibroblast	ACTA2, TGFBI, MGP
PNEC	EpCAM, CALCA, GRP, ASCL1, CHGA
$SCGB1A1^{+}$ Secretory	EpCAM, SCGB1A1, KRT4
$SCGB3A2^{+}$ Secretory	EpCAM, SCGB3A2
Suprabasal	EpCAM, KRT5, KRT6C

Supplementary Table 3. List of marker genes used for hALOs cell type annotation

Cell type	Markers
Undifferentiated Epithelium	EpCAM, NKX2-1
Tip ETV5^{+}	EpCAM, ETV5, NKX2-1
Stalk SOX4^{+}	EpCAM, SOX4
Stalk SCGB3A2^{+}	EpCAM, SCGB3A2
Proliferating stalk	EpCAM, MKI67, TOP2A
Proliferating AT2	EpCAM, MKI67, TOP2A, ABCA3, LAMP3, SFTPC
Proliferating AT1	EpCAM, MKI67, TOP2A, CLIC5, CAV1, AGER
PCNA GHRL^{+}	EpCAM, ASCL1, PCSK1, CHGB, SCG2, GHRL
PCNA	EpCAM, ASCL1, PCSK1, CHGB, SCG2, CALCA, GRP
Myofibroblast	TGFBI, ACTA2
Ciliated	EpCAM, FOXJ1, RSPH1, FAM183A, NME5, TPPP3, DYNLRB2
Cellcycling SCGB3A2^{+}	EpCAM, MKI67, TOP2A, SCGB3A2
Basal	EpCAM, TP63, KRT5
ATF3^{+} cells	EpCAM, ATF3
AT2	EpCAM, ABCA3, LAMP3, SFTPC
AT1	EpCAM, CLIC5, CAV1, AGER

Supplementary Methods:

1. Preparation and characterization of GelMA hydrogel with various stiffness

1.1 Preparation of GelMA hydrogel with various stiffness

GelMA hydrogels with varying stiffness levels were prepared by altering the methacrylation (MA) substitution degree while maintaining a constant precursor concentration (8% w/v) and crosslinking time (30 s). GelMA kits with substitution degrees of 30%, 60%, and 90% were obtained from Engineering for Life Co. (EFL, Cat#EFL-GM-30, Cat#EFL-GM-60, Cat#EFL-GM-90). Hydrogel was prepared following the manufacturer's protocol. Briefly, 8% w/v GelMA precursor was prepared by adding 0.25% w/v lithium phenyl-2,4,6-trimethylbenzoylphosphinate (LAP) photoinitiator to GelMA powder. To prevent solidification, the hydrogel precursor was kept at a 37 °C heat plate and shielded from light until ready for hydrogel preparation.

1.2 Rheological Analysis

For sample preparation, GelMA precursor solutions were injected into PDMS molds (2.5 mm thickness) sandwiched between glass coverslips. To facilitate detachment, coverslips were pretreated with an anti-adherence rinsing solution (STEMCELL, Cat#7010), then rinsed with DPBS (Gibco, Cat#14190250). GelMA precursor was crosslinked under UV light for 30 s. The hydrogel was carefully detached with a spatula and equilibrated in DPBS overnight. Prior to testing, it was punched into 10 mm diameter cylinders using a biopsy punch. The storage modulus (G') and loss modulus (G'') of hydrogels were measured by small-strain oscillatory shear measurements on a rheometer (Anton Paar, Cat#MCR 302e). The mechanical response of the hydrogel sandwiched between the parallel plates of the rheometer was recorded by performing frequency sweep (0.1–50 Hz) measurements in a 1% constant strain mode at room temperature with an angular rate of 5 rad/s.

1.3 Determination of compression modulus

To evaluate the impact of MA grafting ratios on hydrogel stiffness, uniaxial compression testing was conducted. Cross-linked hydrogels of varying stiffness were prepared following "1.2 sample preparation" in the main text and tested using a biological force instrument (EFL, Cat#T5600) equipped with a 5 N load cell. Each construct (n = 3 per group) was compressed at a strain rate of 1 mm/min with a preload of 0.01 N. The compression modulus was calculated from the slope of the stress-strain curve in the range of 0.05–0.1 mm strain.

1.4 Swelling ratio analysis

GelMA hydrogels were incubated in 1 mL of deionized water at 37°C for 120 minutes to reach equilibrium swelling. The swollen hydrogels were weighed, and the swelling ratio was calculated using the following formula:

$$\text{Swelling Ratio} = \frac{w_t - w_0}{w_0} \times 100\%$$

Where W_t is the weight of the hydrogel after 120 minutes and W_0 is the dry weight of the hydrogel measured after lyophilization.

1.5 Scanning electron microscopy (SEM) and pore size analysis

To assess pore size, GelMA hydrogels of varying stiffness were frozen in liquid nitrogen, lyophilized (HUACHEN LAB, Cat#LGJ-12) overnight. The dried hydrogel was cutted to expose the cross-section and sputter-coated with gold (Quorum, Cat#Q150r S Plus). SEM imaging was captured using a field-emission scanning electron microscope (ZEISS, Cat#GeminiSEM 500). Images were analyzed using ImageJ software, and pore sizes were calculated by measuring and averaging the areas of at least 15 pores per sample.

1.6 Degradation testing

GelMA hydrogels were crosslinked in 24-well plates, and the initial hydrogel diameter was measured. Samples were incubated in 500 μL of 20 μg/mL collagenase type II at 37°C. Hydrogels degradation was assessed by measuring the diameter of the hydrogel for up to 1 hour. Images of the samples were captured and diameter was measured for degradation analysis.

2. Differentiation of hESCs into lung organoids

hESCs at approximately 85% confluence were dissociated into single-cell suspensions using Accutase (Stem Cell, Cat#7920) and reseeded at a density of 1.6×10^5 cells/mL onto 24-well plates pre-coated with Matrigel (Corning, Cat#354277). 10 μM Y-27632 (MCE, Cat#HY-10071) was added to mTeSR1 medium on the first day. Once the cells reached 70–80% confluence, differentiation into lung organoids was initiated by sequential administration of specific growth factors as described below.

2.1 Definitive endoderm (DE) medium[1, 2, 3]

DE basal medium was prepared by composition of MCDB131 (Thermo Fisher, Cat#10372019) with 1.8 mg/mL glucose (Sigma, Cat#G7528-1 kg), 1.5 mg/mL sodium bicarbonate (Sigma, Cat#6014-500 g), 0.4% bovine serum albumin (BSA, Proliant, Cat#69700), 1% Penicillin/Streptomycin (Pen/Strep) (Thermo Fisher, Cat#15140122), 1% GlutaMAX (Thermo Fisher, Cat#35050061) and was stored at 4℃ before use. 100 ng/mL Activin A (StemCell, Cat#78001) and CHIR-99021 (MCE, Cat#HY-10182) with decreasing concentration of 3, 0.1 and 0 μM for 3 days were added to DE basal medium on the day of use.

2.2 Anterior foregut (AFE) medium[4, 5]

The AFE basal medium was prepared by addition of Advanced DMEM/F12 medium (Thermo Fisher, Cat#12634010) with 2% B27 supplement (Thermo Fisher, Cat#17504044), 1% N2 (Thermo Fisher, Cat#17502048), 1% GlutaMAX, 1% Pen/Strep, 1% HEPES (Thermo Fisher, Cat#15630080) and stored at 4°C before use. 1 μM SAG (MCE, Cat#HY-12848), 10 μM SB-431542 (MCE, Cat#HY-10431), 200 ng/mL Noggin (MCE, Cat#HY-P7051A), 500 ng/mL FGF4 (MCE, Cat#HY-P7014) and 2 μM CHIR-99021 were added to the AFE basal medium and the medium was refreshed daily for 3 to 5 days, depending on the formation of self-assemble spheroids.

2.3 Lung progenitor cell (LPC) medium[5]

LPC basal medium is the same with AFE basal medium. 10 μM DAPT (MCE, Cat#HY-13027), 20 ng/mL BMP4 (MCE, Cat#HY-P7007), 10 ng/mL FGF7 (MCE, Cat#HY-P70597), 10 ng/mL FGF10 (MCE, Cat#HY-P70695), 0.5 μM retinoic acid (Sigma, Cat#R2625), 3 μM CHIR-99021 were added to LPC basal medium and LPC medium was refreshed every other day for 7 days.

2.4 Human airway organoids (hAWOs) medium[6]

hAWOs basal medium was prepared by supplementation of DMEM/F12 medium (Thermo Fisher, Cat#10565018) with 1% B27 supplement, 0.25% BSA, 0.1% ITS-X (Thermo Fisher, Cat#51500056), and 1% Pen/Strep. 50 nM dexamethasone (MCE, Cat#HY-14648), 100 nM 8-Br-cAMP (MCE, Cat#HY-12318), 100 nM 3-isobutyl-1-methylxanthine (MCE, Cat#HY-12318), 10 ng/mL FGF7 were added before using. The hAWOs medium was refreshed on Monday morning, Wednesday afternoon and Friday afternoon every week for 35 to 45 days.

2.5 Human alveolar organoids (hALOs) medium

To obtain hALOs medium, hAWOs medium was supplemented with 3 μM CHIR-99021 and 10 μM SB-431542. The hALOs medium was refreshed Monday morning, Wednesday afternoon and Friday afternoon every week for 35–45 days.

3. Biocompatibility testing of GelMA extracts according to ISO 10993-5

To determine whether the residual uncrosslinked hydrogel components could affect cell behavior independently of stiffness, the cell viability and differentiation efficiency exposed to hydrogel extracts were assessed following ISO 10993-5[7] with minor modification. Briefly, 25 μL droplets of Materigel (control) and GelMA (G30, G60 and G90) were polymerized without cells (3 repeats per condition), overlaid with 550 μL LPC culture medium, and incubated at 37°Cfor 24 h to generate conditioned medium (hydrogel “extracts”). The next day, AFE

spheroids were embedded in growth factor reduced Matrigel (Corning, Cat#354230) and polymerized at 37°C for 10 min. The 24 h extracts (Matrigel, G30, G60 and G90) were applied to the Matrigel-embedded AFE spheroids. Media (extracts or control) were replaced every other day. After 7 days, 20 μL of CCK8 (MCE, Cat#HY-K0301) was added per well and incubated for 2 h at 37°C. The absorbance was measured at 450 nm using a microplate reader. Relative cell viability was calculated as below:

$$\text{Relative cell viability (\%)} = (As-Ab)/(Ac-Ab)\times 100\%$$

Where As is the samples absorbance, Ac is the control absorbance, Ab is the blank (without cells) absorbance.

In parallel, LPCs were lysed and total mRNA were extracted for *NKX2-1* expression analysis (qPCR).

4. Live/dead staining assay

The viability of LPCs encapsulated in hydrogels was evaluated using a Calcein-AM/propidium iodide (PI) Live/Dead Cell Viability Assay Kit (Solarbio, Cat#CA1630). Live/Dead staining was performed on day 7 LPCs following previous published protocol[8] with minor modification. Live cells were labeled with Calcein-AM and dead cells with PI. Briefly, LPCs were released from GelMA using collagenase Type II, then incubated with Calcein-AM (2 μM) and PI (4 μM) premixed in DPBS at 37 °C for 30 min, protected from light. After staining, samples were washed three times with DPBS, transferred to glass bottom cell culture dish (NST, Cat#801002) and imaged using an inverted fluorescence microscope (Carl Zeiss LSM 980).

5. Immunofluorescence (IF) staining[9, 10]

After releasing the organoids from the hydrogel, hAWOs were rinsed with DPBS twice and subsequently fixed in 4% w/v paraformaldehyde (PFA) (Sangon Biotech, Cat#E672002-0500) for 30 minutes at 18-25 °C. Following fixation, organoids were washed three times with DPBS and dehydrated in 30% sucrose at 4 °C until they settled at the bottom of the centrifuge tube. The organoids were then embedded in Cryo Embedding Medium (Biosharp, Cat#BL557A), flash-frozen in liquid nitrogen, and stored at −80 °C. Frozen organoids were sectioned into 10 μm slices using a cryomicrotome (Minux® FS800, RWD Life science Co., LTD) and mounted on adhesive slides for long-term storage at −20 °C. Before staining, sections were briefly fixed again in 4% PFA, permeabilized, and blocked for 30–60 minutes in blocking buffer (Beyotime, Cat#P0260). Primary antibodies diluted in antibody diluent (Beyotime, Cat#P0103) were applied and incubated overnight at 4 °C. Secondary antibodies and Hoechst

33342 (Thermo Scientific, Cat#875756-97-1) were added for 1 h at room temperature. Finally, stained sample were mounted using anti-fading mounting medium (ACMEC, Cat#AS2100). LPCs and hALOs with small size used whole mounting without cryosection. Briefly, the samples were collected and precipitated at the bottom of a 15 mL centrifuge tube, then fixed in 4% PFA and stained following the staining protocol described above.

6. Flow cytometry and fluorescence-activated cell sorting (FACS)

Organoids were dissociated into single cells using TrypLE (Thermo Fisher, Cat#12605028) at 37°C for 5–15 minutes, with gentle pipetting every 6 minutes to minimize cell clumping. An equal volume of DMEM/F-12 medium supplemented with 10 μM Y-27632 was added to terminate the digestion. Single cells were centrifuged at 300 × *g* for 3 minutes.

For flow cytometry analysis, single cells were fixed in 4% PFA for 30 minutes. Cells were permeabilized, blocked, and stained with primary and secondary antibodies according to the IF staining protocol. Stained cells were resuspended in sorting buffer (1 mM EDTA + 1% BSA in PBS) and passed through a 35 μm cell strainer (BD Falcon, Cat#352235) into FACS tubes and protecting from light. The stained single cells were ran and analyzed using Agilent Novocyte Advanteon (Agilent Technologies).

For FACS, the dissociation protocol of organoids was the same as above except all steps were performed on ice to preserve cell viability. After digestion, cells were incubated with EpCAM antibody (Biolegend, Cat#369810) on ice for 30 minutes. Single cells with $EpCAM^{+}$ were sorted using Sony MA900 sorter (Cat#LE-MA900FP) and collected in sorting buffer with Y-27632. Data were processed with NovoExpress (Agilent Novocyte Advanteon) and FlowJo_V10 software.

7. Transmission Electron Microscopy (TEM)[6]

Organoids were collected and fixed in 2.5% glutaraldehyde for 1 hour at room temperature, then transferred into fresh 2.5% glutaraldehyde for preservation at 4℃. Fixed samples were washed in 0.1 M PBS (pH = 7.4) for 15 minutes per wash, repeating this process four times. The samples were transferred to 1% osmium tetroxide for 1.5 hours at room temperature (performed in a fume hood due to osmium toxicity). After washing with PBS for 3 times, samples were sequentially dehydrated in graded ethanol solutions (50%, 70%, 80%, 90%, and 100% × 2) for 5 minutes each, followed by dehydration in acetone.

Samples were infiltrated with a 1:1 mixture of acetone and 812 resin for 2–4 hours at room temperature, and then embedded in fresh 812 resin, polymerization at 40°C for 24 hours, and

subsequently at 60°C for 12 hours. Ultrathin sections (80–100 nm) were sectioned using an ultramicrotome, mounted on grids, and stained with uranyl acetate and lead citrate, and imaged using a TEM (Thermo Fisher Scientific, Talos L120C G2).

8. Viral RNA isolation and quantification

To isolate viral RNA from infected lung organoids, SARS-CoV-2 infected organoids were lysed in 600 μL of RLT Plus buffer (QIAGEN, Cat#1053393) supplemented with 1% β-mercaptoethanol (Gibco, Cat#21985023). The lysate was mixed thoroughly with a pipette until complete lysis was achieved. The lysate was then stored at −20°C or −80°C for subsequent RNA isolation using the QIAGEN RNeasy Plus Mini Kit, following the manufacturer's instructions (QIAGEN, Cat#74106).

Viral RNA from the supernatant was isolated by combining 140 μL of supernatant with 560 μL of AVL buffer (QIAGEN, Cat#19089). The mixture was thoroughly vortexed and stored at −20°C until processing with the QIAGEN Viral RNA Mini Kit (QIAGEN, Cat#52906) according to the manufacturer's protocol. cDNA synthesis was performed with equal amounts of RNA from each sample using the Maxima H Minus First Strand cDNA Synthesis Kit (Thermo Fisher, Cat#EP0753). DNA copy numbers were calculated using the following formula for genome equivalents:

$$\text{DNA Copies} = \frac{\text{DNA amount (g)} \times 6.022 \times 10^{23}\ \text{(copies/mol)}}{\text{Length (bp)} \times 660\ \text{(g/mol/dp)}}$$

where DNA amount is 68.8 ng/μL, 6.022×10^{23} is Avogadro's number, length vector + insert is the transcript length in nucleotides (4049 bp), and 660 is the average molecular weight of a single nucleotide (estimated at 660 Da). This calculation enabled the determination of DNA copy numbers for subsequent analysis.

9. Single-cell RNA sequencing (scRNA-seq) of organoids and analysis

9.1 Preparation of Single-cell Suspensions

Lung organoids cultured under different stiffness conditions for 49 days were dissociated into single-cell suspensions using TrypLE (Thermo Fisher, Cat#12605028). The suspensions were passed through a 70 μm cell strainer (BD Falcon, Cat#352235) into FACS tubes, ensuring a single-cell yield of over 90%. For hAWOs, epithelial cell-positive populations were pre-sorted using a flow cytometer, following previously described Section 6 "Flow cytometry and fluorescence-activated cell sorting (FACS)". Cell viability and concentration were assessed using Trypan Blue Stain (Invitrogen, Cat#15250061) with the Countess™ 3 Automated Cell

Counter (Life Technologies). Final cell concentrations were adjusted to 1000 cells/μL with a viability of ≥80%.

9.2 Library Construction and Sequencing for hAWOs

hAWOs were sequenced on the C4 platform by Geneplus Gene Technology. Single-cell suspensions were processed using the DNBelab™ C Series High-throughput scRNA Library Kit (MGI, Cat#940-001818-00). Libraries were constructed following the manufacturer's instructions, including cDNA synthesis, size selection, end repair, A-tailing, adapter ligation, index library amplification (PCR), and library circularization of the sequencing library to build a library of 3' transcripts. Purification and quantification was performed using the Qubit ssDNA Assay Kit (Thermo Fisher) and Qsep100 (Bioptic). Sequencing was conducted on the DNBelab™ C4/TaiM4 platform with paired-end reads on a DNBSEQ-T7 sequencer.

9.3 Library Construction and Sequencing for hALOs

hALOs were sequenced on the 10x Genomics platform by Annoroad Gene Technology. Single-cell suspensions were combined with Gel Beads and oil droplets in separate channels of Chromium Chip G (10x Genomics, Cat#1000121) to generate Gel Bead-In-Emulsions (GEMs) via microfluidics. Following the manufacturer's protocol, mRNA was reverse-transcribed into complementary DNA (cDNA) and subsequently amplified into double-stranded cDNA. A-tailing was performed, followed by ligation of sequencing adapters, including the Read2 primer. The resulting libraries incorporated P5 and P7 adapters and dual indices, enabling compatibility with Illumina sequencing platforms. The quality-controlled libraries were sequenced on an Illumina platform by Annoroad Gene Technology, ensuring high-quality data acquisition for downstream analysis.

9.4 scRNA-seq data pre-processing and quality control

Raw sequencing data was processed with the DNBelab™ C Series Single-cell RNA Analysis Report V2.1.1 (hAWOs) and CellRanger v8.0.0 (hALOs) using default parameters. Reads from three biological replicates of hAWOs and the three biological replicates of hALOs were each mapped to the GRCh38 genome. Low-quality cells were excluded based on a cutoff of fewer than 500 genes or 1000 UMIs. Doublets were predicted using DoubletFinder (v2.0.4)[11], DoubletDetection (v4.2)[12], scDblFinder (v1.18.0)[13], and Scrublet (v0.2.3)[14], and cells flagged by at least two methods were excluded. Gruffi (v1.5.5)[15] was used to remove stressed cells of organoids with default parameters. All statistical analysis for violin charts and box charts is performed using 'wilcox.test (alternative=" two-sided ")' in the R package "stats".

9.5 Normalizing, integrating, clustering and annotation

Downstream analysis was performed using Seurat (v5.1.0)[16]. After quality control, gene-cell count matrices for hAWOs (G30, G60, G90) and hALOs (G30, G60, G90) were merged separately using the 'Seurat::merge()' function. Data normalization and batch correction were conducted with 'SCTransform()'. In detail, we applied method = "glmGamPoi" to improve modeling of gene expression variability. All samples were integrated simultaneously, allowing Seurat to internally identify shared features and compute a unified model based on Pearson residuals. To eliminate the batch effects among the 3 replicates, we used Seurat's IntegrateLayers (method = CCAIntegration) to integrate the data, following by constructing a KNN graph with 'FindNeighbors()'. Unsupervised clustering was performed using the Louvain algorithm of 'FindClusters()' with resolutions from 0.1 to 2.0, and low dimensionality visualization was conducted via 'RunUMAP()'. To avoid the potential influence of low quality cells and technical noise, we further performed subclustering on each cluster with resolution from 1.0 to 2.0, clusters with valuable canonical features were remained while clusters who have very low nUMIs/nGenes, or very high score of mitochondrial genes were excluded in the later analysis.

Manual annotation was performed using curated cell type markers[17, 18, 19]. Clusters were categorized into two main groups: Epithelium and Mesenchyme. For both hAWOs and hALOs scRNA data, epithelial cells constituted the largest proportion, displaying substantial heterogeneity within their cluster. Then we re-annotated each main group through unsupervised high-resolution clustering and cell type enrichment analysis via 'AddModuleScore()'. A total of 10 cell types were identified for hAWOs and 16 for hALOs referenced previous literatures[18, 20]. MetaNeighbor[21] correlation analysis using 'MetaNeighborUS()' confirmed cell type replicability across stiffness conditions.

9.6 Stiffness-mediated differentially expressed genes (DEG) analysis

DEG analysis was performed using the 'FindAllMarkers()' function in Seurat, calculating fold change and performing Wilcoxon Rank Sum tests. DEG sets for comparisons (G30 vs. G60, G60 vs. G90, G30 vs. G90) were intersected to identify genes consistently upregulated or downregulated with increasing stiffness.

9.7 Absolute differentiation potential score for organoid cells

CytoTRACE2 (v1.0.0)[22] was employed to predict cellular potency categories and absolute developmental potential based on normalized gene counts. Default parameters were applied using 'cytotrace2(full_model=T)' to calculate absolute differentiation potential scores.

9.8 Prediction of the physiological relevance of organoids with varying stiffness to the human fetal airway

To assess the cellular composition and physiological relevance of our hAWOs cultured under different stiffness conditions, we performed a reference-based mapping and annotation analysis using a public scRNA-seq dataset of the developing human airway[23]. This reference dataset provided a comprehensive transcriptional atlas of human fetal trachea, airway and distal lung during different developmental stages ranging from 11.5 weeks to 21 weeks.

First, the public reference dataset was processed and an integrated reference was constructed following standard procedures of Seurat. Our integrated query dataset was then queried onto the reference, we identified anchors between the query and the reference using the `FindTransferAnchors()`, and then used the `TransferData()` to classify each cell in our query data. This function leverages the anchor correspondences to infer tissue position labels from the reference, providing a predicted identity (e.g., trachea, airway and distal) and a corresponding prediction score for every query cell. For visualization, we optionally projected the query cells onto the reference UMAP structure using the `MapQuery()`. Finally, we quantitatively compared the predicted tissue position labels of organoids across different stiffness groups.

9.9 Alveolar trajectory inference analysis

To reconstruct alveolar developmental trajectories, cells from tip ETV5^{+}, AT2, proliferating AT2, AT1, and proliferating AT1 clusters were re-annotated. To avoid the bias in cell abundance caused by the sample cell quantity of different stiffness, we took the cell type with the smallest number as the reference and conducted equal random sampling for each of the remaining cell types of alveolar cells. This ensured that the prerequisite for the subsequent trajectory analysis was the balance and comparability of the number of each cell type. Trajectory inference was initially performed using Slingshot[24] based on the recomputed UMAP embedding, and then utilized STREAM (v1.1)[25] to reconstruct gene expression matrix in a structure called the principal graph, which is a set of curves that naturally describe the cell-pseudotime, trajectories, and branching points. In detail, 'seed_elastic_principal_graph (clustering="kmeans", n_clusters=12, use_vis=True)' was initially applied to construct the principal graph based on recomputed UMAP embedding, and then 'elastic_principal_graph (epg_alpha=0.02, epg_mu=0.1, epg_lambda=0.01)' was applied to smooth the trajectory. Two trajectories were identified: AT1 lineage and the AT2 lineage. Pseudotime-correlated genes were visualized using 'SCP::GroupHeatmap()'. PAGA analysis[26, 27] was used to verify trajectory robustness, showing consistency with STREAM-derived trajectories.

Supplementary References:

1. Meng H*, et al.* FGF7 enhances the expression of ACE2 in human islet organoids aggravating SARS-CoV-2 infection. *Signal Transduct Target Ther* **9**, 104 (2024).
2. Pagliuca FW*, et al.* Generation of functional human pancreatic beta cells in vitro. *Cell* **159**, 428-439 (2014).
3. Velazco-Cruz L*, et al.* Acquisition of Dynamic Function in Human Stem Cell-Derived beta Cells. *Stem Cell Reports* **12**, 351-365 (2019).
4. Hein RFC*, et al.* Stable iPSC-derived NKX2-1+ lung bud tip progenitor organoids give rise to airway and alveolar cell types. *Development* **149**, dev200693 (2022).
5. Miller AJ*, et al.* Generation of lung organoids from human pluripotent stem cells in vitro. *Nat Protoc* **14**, 518-540 (2019).
6. Pei R*, et al.* Host metabolism dysregulation and cell tropism identification in human airway and alveolar organoids upon SARS-CoV-2 infection. *Protein & Cell* **12**, 717-733 (2021).
7. ISO10993-5:2009. Biological evaluation of medical devices — Part 5: Tests for in vitro cytotoxicity.). ISO (International Organization for Standardization) (2009).
8. Xia M*, et al.* Varying mechanical forces drive sensory epithelium formation. *Sci Adv* **9**, eadf2664 (2023).
9. Nason-Tomaszewski CE, Thomas EE, Matera DL, Baker BM, Shikanov A. Extracellular matrix-templating fibrous hydrogels promote ovarian tissue remodeling and oocyte growth. *Bioactive Materials* **32**, 292-303 (2024).
10. McCauley KB, Hawkins F, Serra M, Thomas DC, Jacob A, Kotton DN. Efficient Derivation of Functional Human Airway Epithelium from Pluripotent Stem Cells via Temporal Regulation of Wnt Signaling. *Cell Stem Cell* **20**, 844-857 e846 (2017).
11. McGinnis CS, Murrow LM, Gartner ZJ. DoubletFinder: Doublet Detection in Single-Cell RNA Sequencing Data Using Artificial Nearest Neighbors. *Cell Systems* **8**, 329-337.e324 (2019).
12. Park J*, et al.* Cell segmentation-free inference of cell types from in situ transcriptomics data. *Nature Communications* **12**, 3545 (2021).
13. Germain P-L, Lun A, Macnair W, Robinson MD. Doublet identification in single-cell sequencing data using scDblFinder. *F1000Research* **10**, 979 (2021).
14. Wolock SL, Lopez R, Klein AM. Scrublet: Computational Identification of Cell Doublets in Single-Cell Transcriptomic Data. *Cell Systems* **8**, 281-291.e289 (2019).
15. Vértesy Á*, et al.* Gruffi: an algorithm for computational removal of stressed cells from brain organoid transcriptomic datasets. *The EMBO Journal* **41**, e111118 (2022).
16. Hao Y*, et al.* Dictionary learning for integrative, multimodal and scalable single-cell analysis. *Nature Biotechnology* **42**, 293-304 (2024).
17. Cao S*, et al.* Single-cell RNA sequencing reveals the developmental program underlying proximal-distal patterning of the human lung at the embryonic stage. *Cell Res* **33**, 421-433 (2023).
18. He P*, et al.* A human fetal lung cell atlas uncovers proximal-distal gradients of differentiation and key regulators of epithelial fates. *Cell* **185**, 4841-4860.e4825 (2022).
19. Travaglini KJ*, et al.* A molecular cell atlas of the human lung from single-cell RNA sequencing. *Nature* **587**, 619-625 (2020).
20. Basil MC*, et al.* Human distal airways contain a mulipotent secretory cell that can regenerate alveoli. *Nature* **604**, 120-126 (2022).
21. Crow M, Paul A, Ballouz S, Huang ZJ, Gillis J. Characterizing the replicability of cell types defined by single cell RNA-sequencing data using MetaNeighbor. *Nature Communications* **9**, 884 (2018).

22. Kang M*, et al.* Improved reconstruction of single-cell developmental potential with CytoTRACE 2. *Nature Methods* **22**, 2258-2263 (2025).
23. Miller AJ*, et al.* In Vitro and In Vivo Development of the Human Airway at Single-Cell Resolution. *Dev Cell* **53**, 117-128 e116 (2020).
24. Street K*, et al.* Slingshot: cell lineage and pseudotime inference for single-cell transcriptomics. *BMC Genomics* **19**, 477 (2018).
25. Chen H*, et al.* Single-cell trajectories reconstruction, exploration and mapping of omics data with STREAM. *Nature Communications* **10**, 1903 (2019).
26. Wolf FA*, et al.* PAGA: graph abstraction reconciles clustering with trajectory inference through a topology preserving map of single cells. *Genome Biology* **20**, 59 (2019).
27. Kadur Lakshminarasimha Murthy P*, et al.* Human distal lung maps and lineage hierarchies reveal a bipotent progenitor. *Nature* **604**, 111-119 (2022).
