# Supplementary material for: Extracellular matrix stiffness directs region-specific lung epithelial differentiation revealed by hPSC-derived lung organoids
Source: Nat Commun. 2026 Jul 17;17:8757. doi: 10.1038/s41467-026-75663-9 (PMC13493954; doi:10.1038/s41467-026-75663-9)
Supplement: Supplementary file 4 [file 41467_2026_75663_MOESM2_ESM.pdf]

# Description of Additional Supplementary Files

**File name: Supplementary Data 1**
Description: Detailed information for primary and secondary antibodies used in this study, including sources, catalog numbers and dilution ratios.

**File name: Supplementary Movie 1**
Description: Representative ciliary beating in hAWOs differentiated under the G30 stiffness condition.

**File name: Supplementary Movie 2**
Description: Representative ciliary beating in hAWOs differentiated under the G60 stiffness condition.

**File name: Supplementary Movie 3**
Description: Representative ciliary beating in hAWOs differentiated under the G90 stiffness condition.

**File name: Supplementary Movie 4**
Description: Clearance of fluorescent microbeads driven by ciliary beating in the G30 group.

**File name: Supplementary Movie 5**
Description: Clearance of fluorescent microbeads driven by ciliary beating in the G60 group.

**File name: Supplementary Movie 6**
Description: Clearance of fluorescent microbeads driven by ciliary beating in the G90 group.
